# Supplementary figures and images for: Pharmacological CDK4/6 inhibition promotes vulnerability to lysosomotropic agents in breast cancer (part 1 of 2)
Source: EMBO J. 2025 Feb 10;44(7):1921–42. doi: 10.1038/s44318-025-00371-x (PMC11961731; doi:10.1038/s44318-025-00371-x)

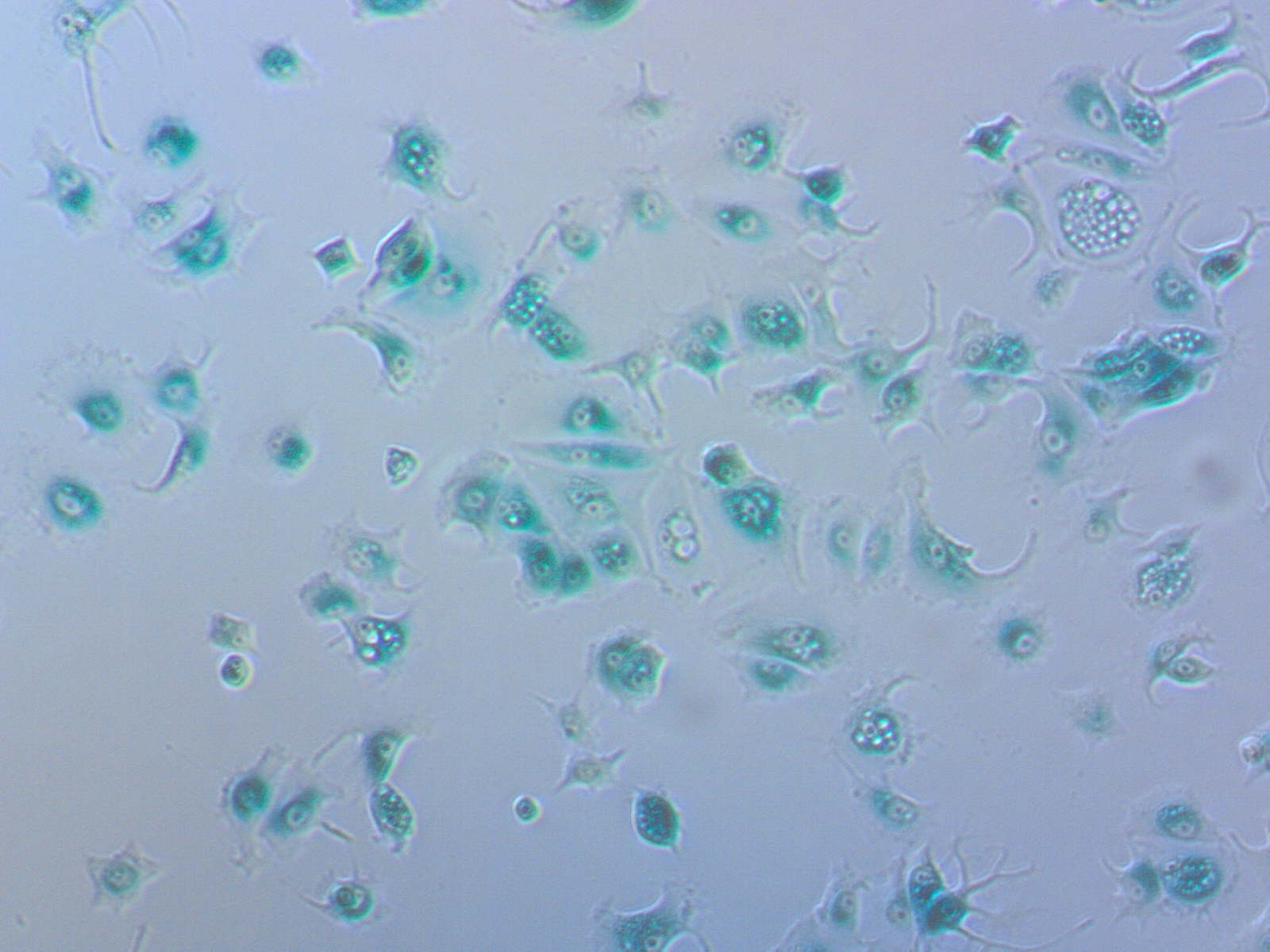

Supplement: Supplementary file 2 — Source data Fig. 1 [file 44318_2025_371_MOESM2_ESM.zip › SourceData_Figure 1/1A/ABEMA-2.jpg]

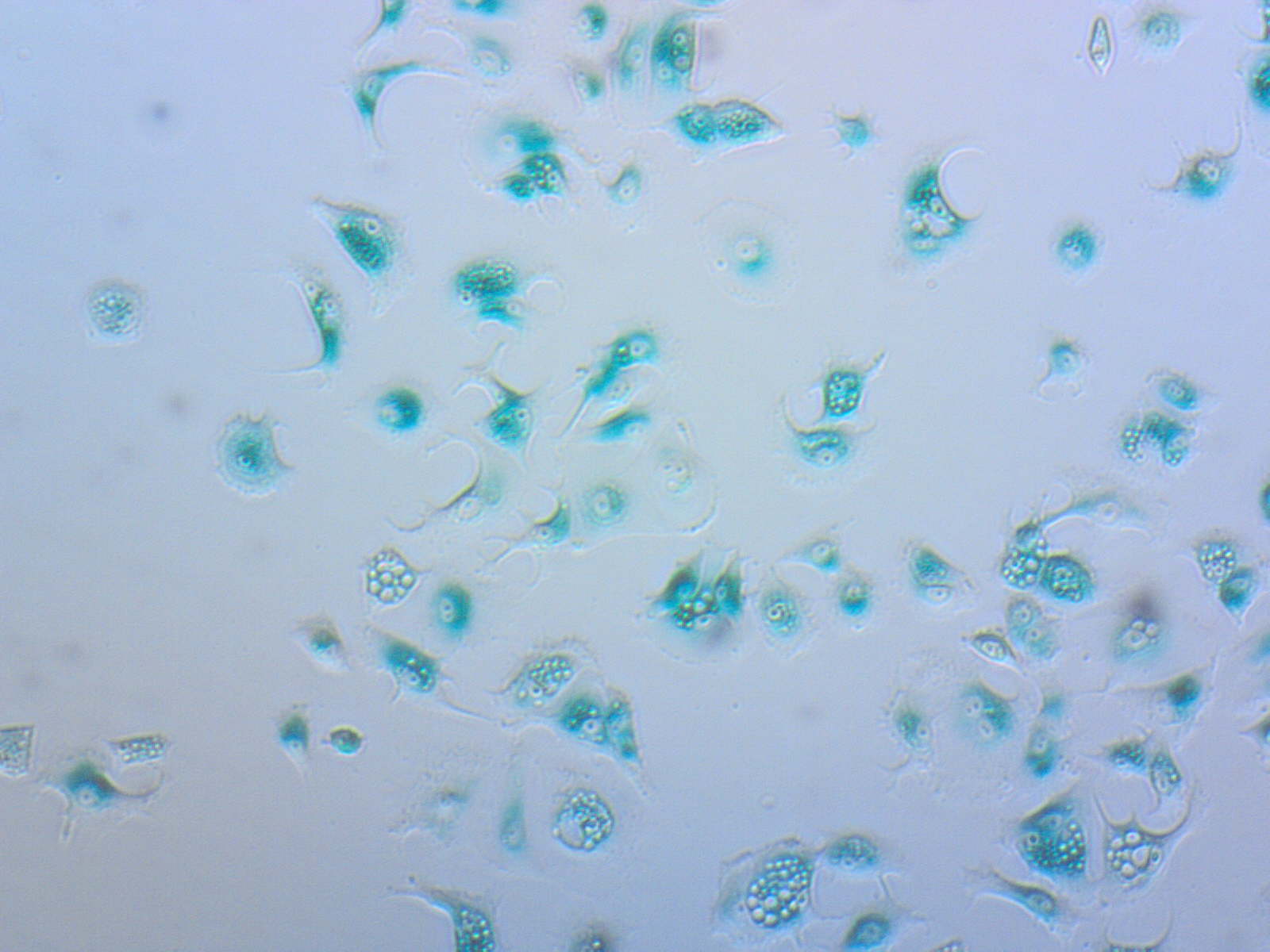

Supplement: Supplementary file 2 — Source data Fig. 1 [file 44318_2025_371_MOESM2_ESM.zip › SourceData_Figure 1/1A/ABEMA-3.jpg]

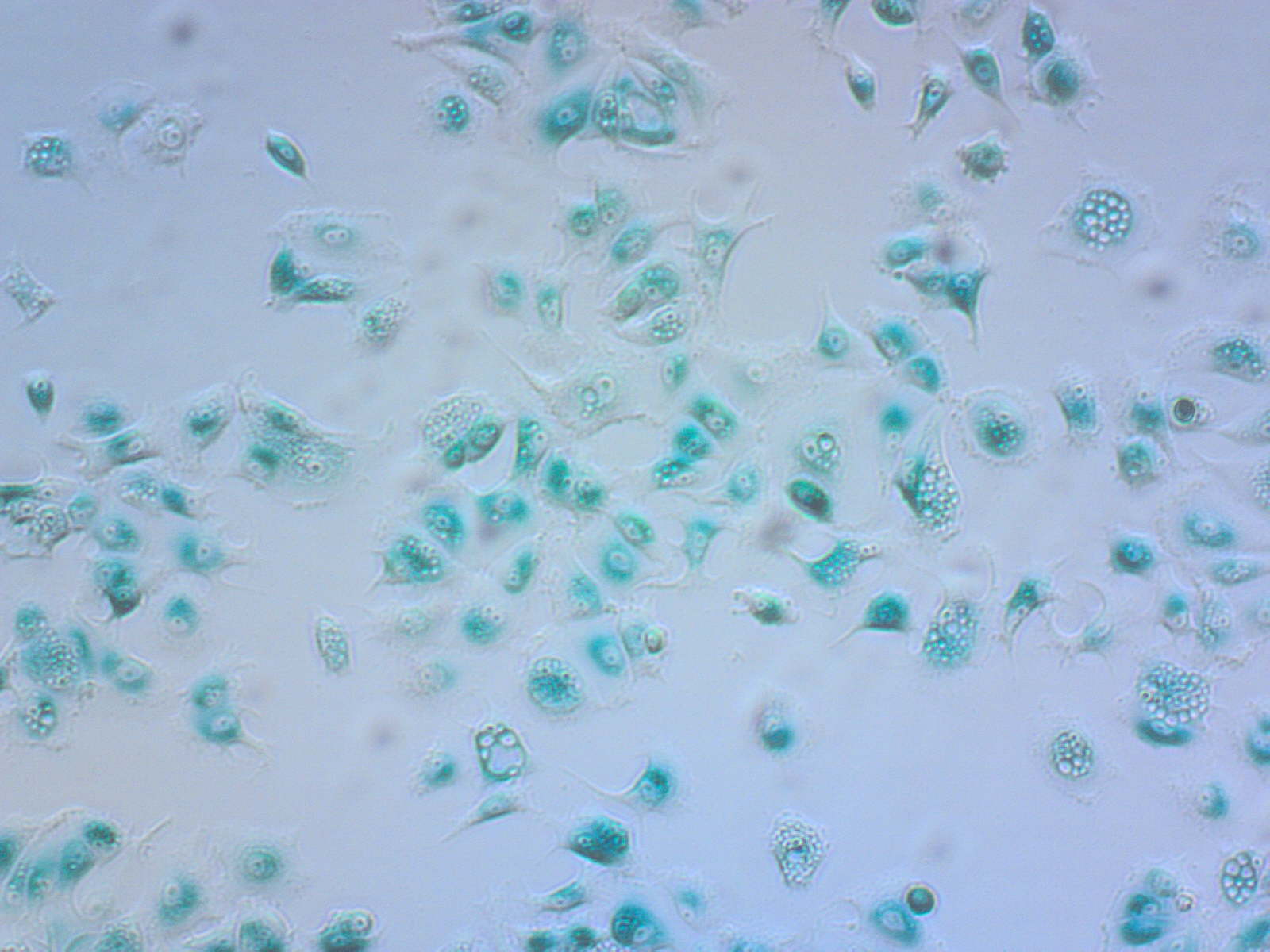

Supplement: Supplementary file 2 — Source data Fig. 1 [file 44318_2025_371_MOESM2_ESM.zip › SourceData_Figure 1/1A/ABEMA-1.jpg]

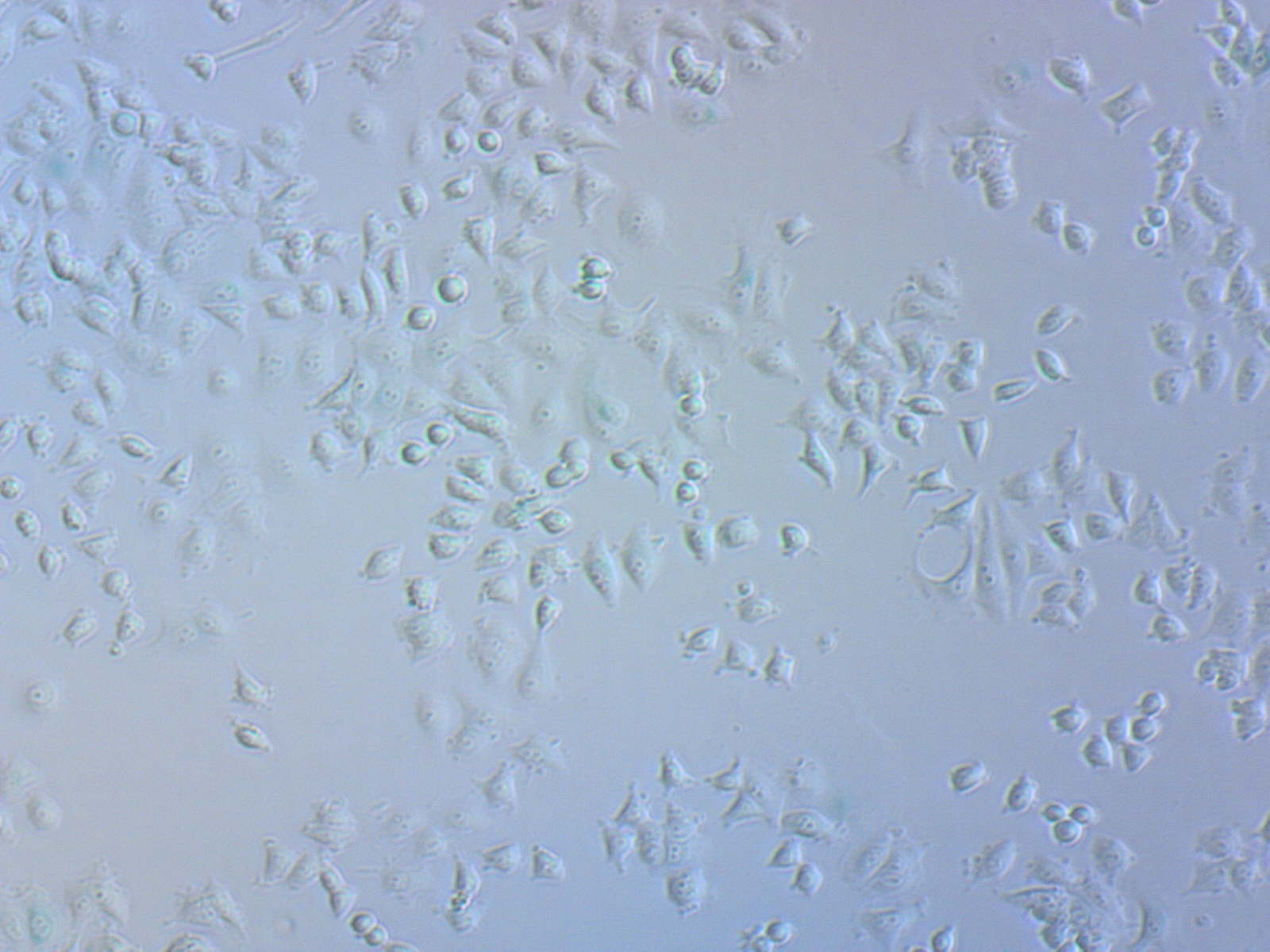

Supplement: Supplementary file 2 — Source data Fig. 1 [file 44318_2025_371_MOESM2_ESM.zip › SourceData_Figure 1/1A/VEH-1.jpg]

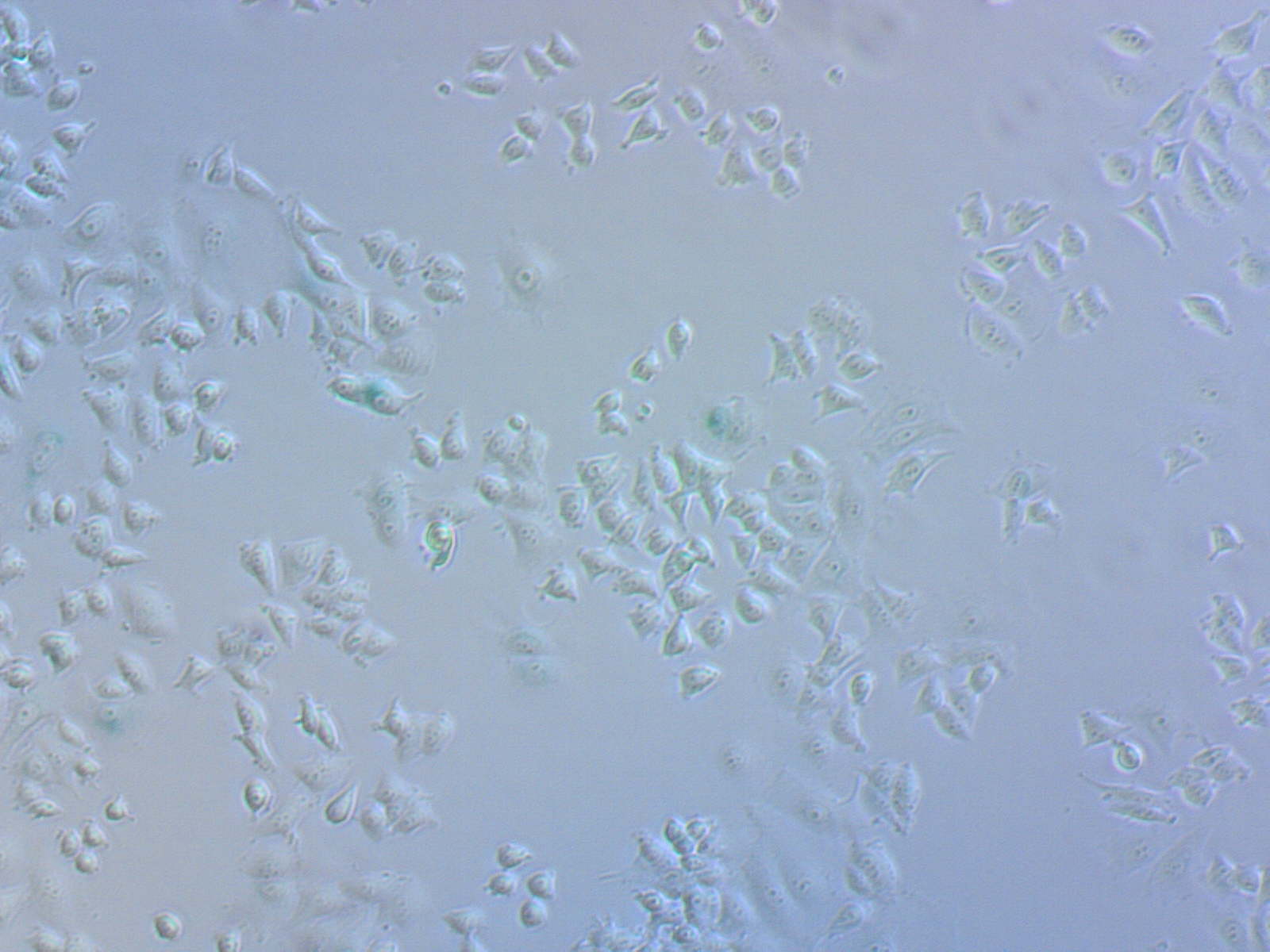

Supplement: Supplementary file 2 — Source data Fig. 1 [file 44318_2025_371_MOESM2_ESM.zip › SourceData_Figure 1/1A/VEH-2.jpg]

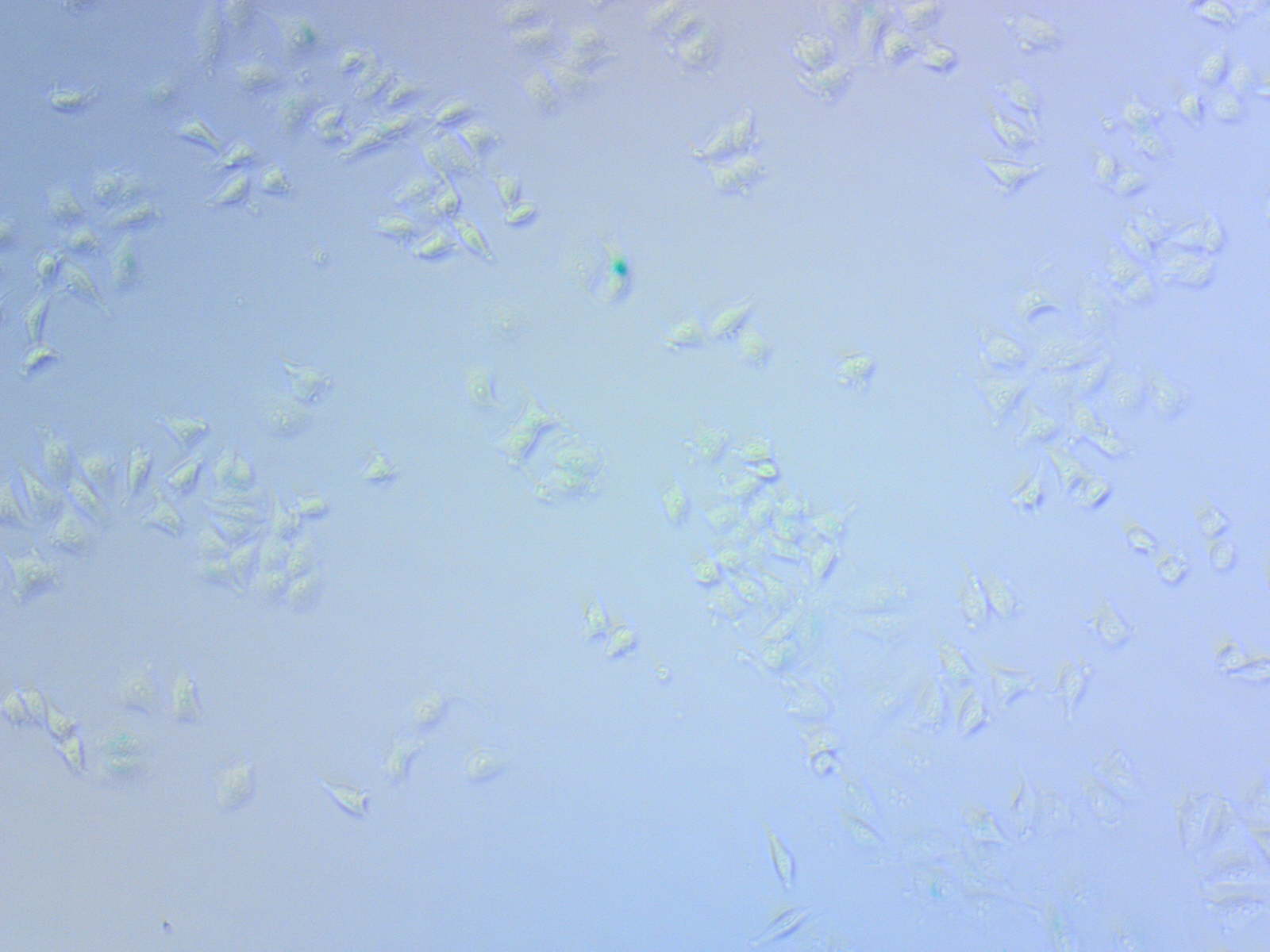

Supplement: Supplementary file 2 — Source data Fig. 1 [file 44318_2025_371_MOESM2_ESM.zip › SourceData_Figure 1/1A/VEH-3.jpg]

## Slide 1
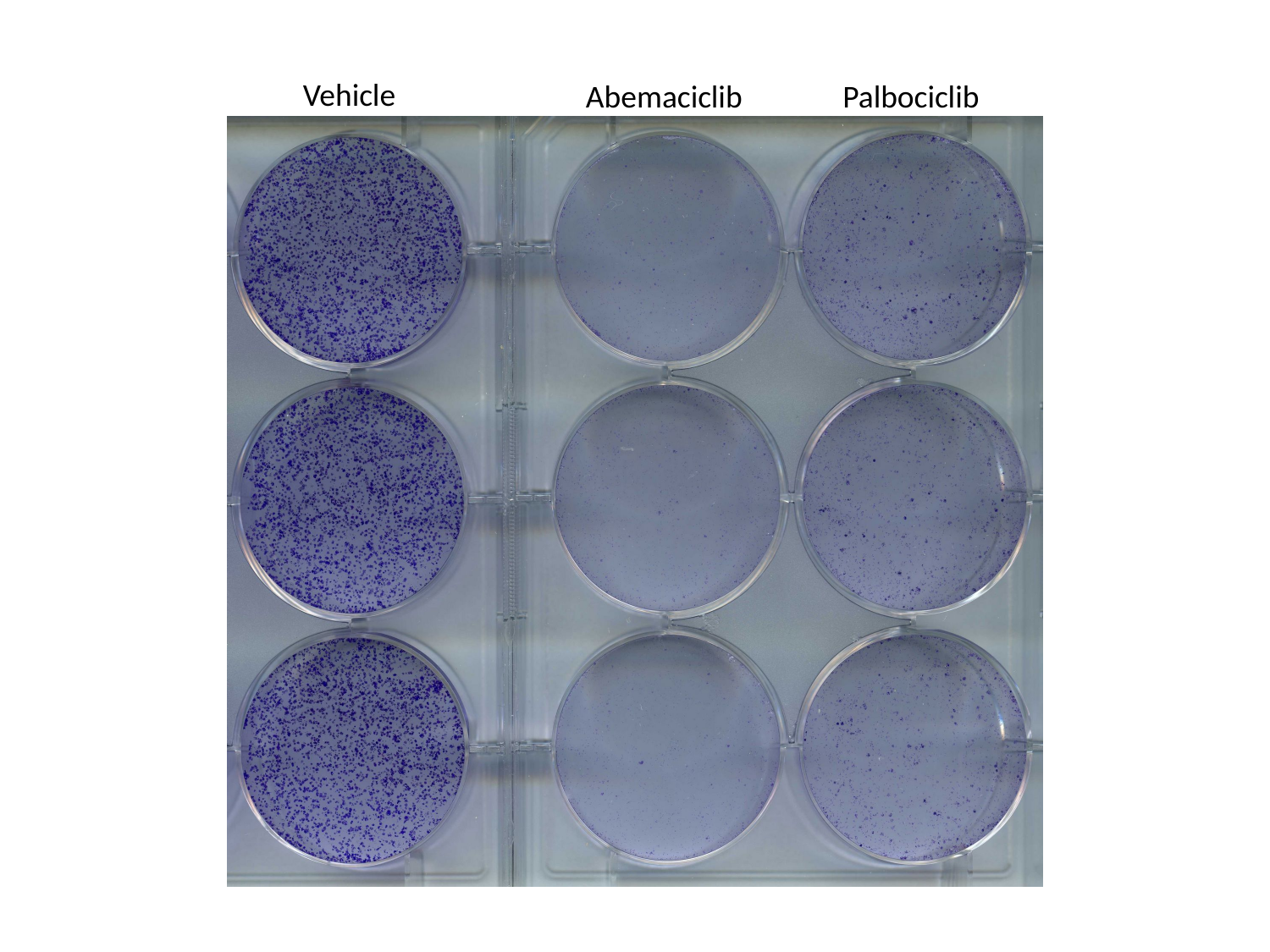

Vehicle
Abemaciclib
Palbociclib

Supplement: Supplementary file 2 — Source data Fig. 1 [file 44318_2025_371_MOESM2_ESM.zip › SourceData_Figure 1/1B/MCF7.pptx]

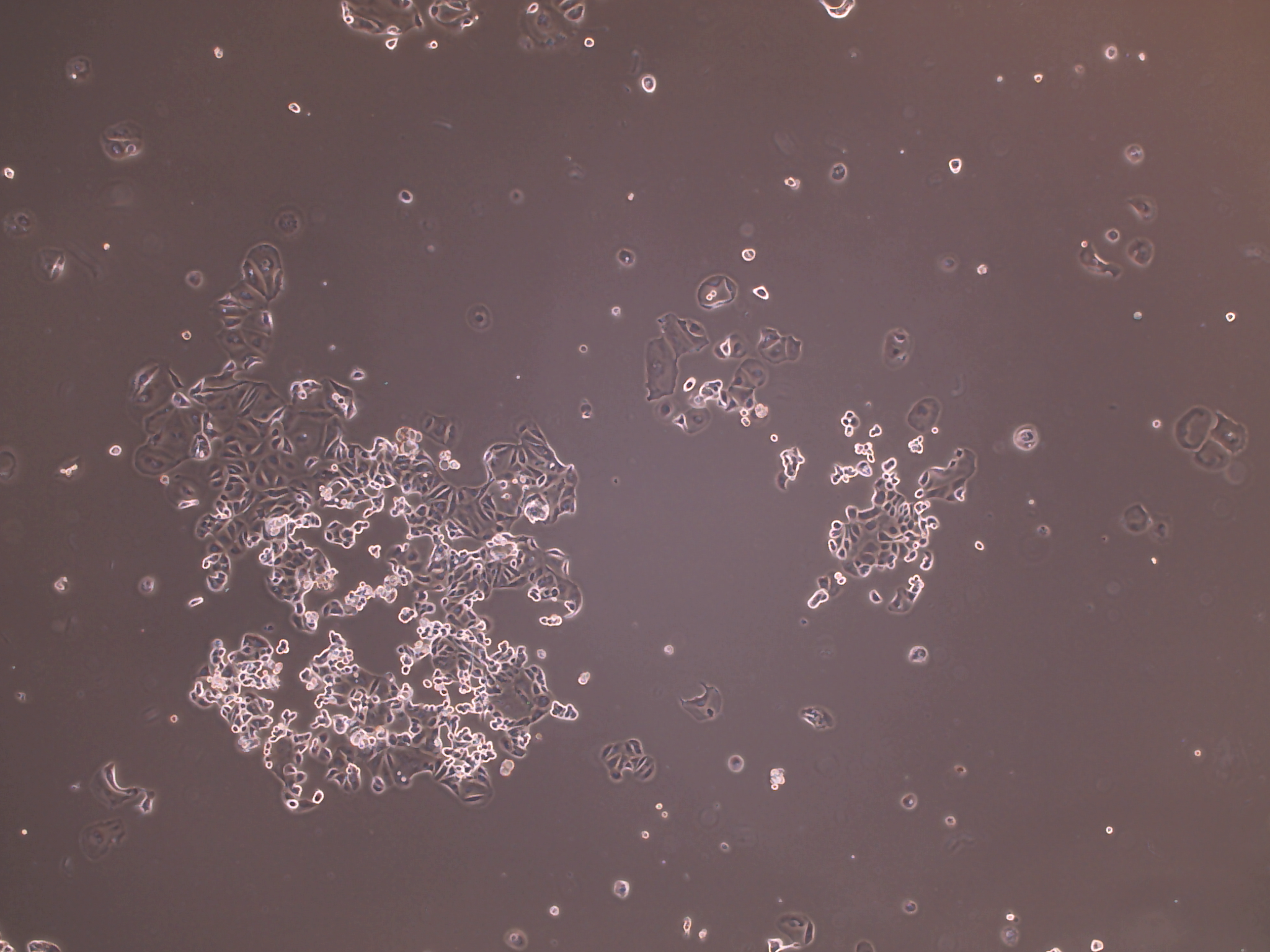

Supplement: Supplementary file 2 — Source data Fig. 1 [file 44318_2025_371_MOESM2_ESM.zip › SourceData_Figure 1/1D/mcf7 30dpt-2.tif]

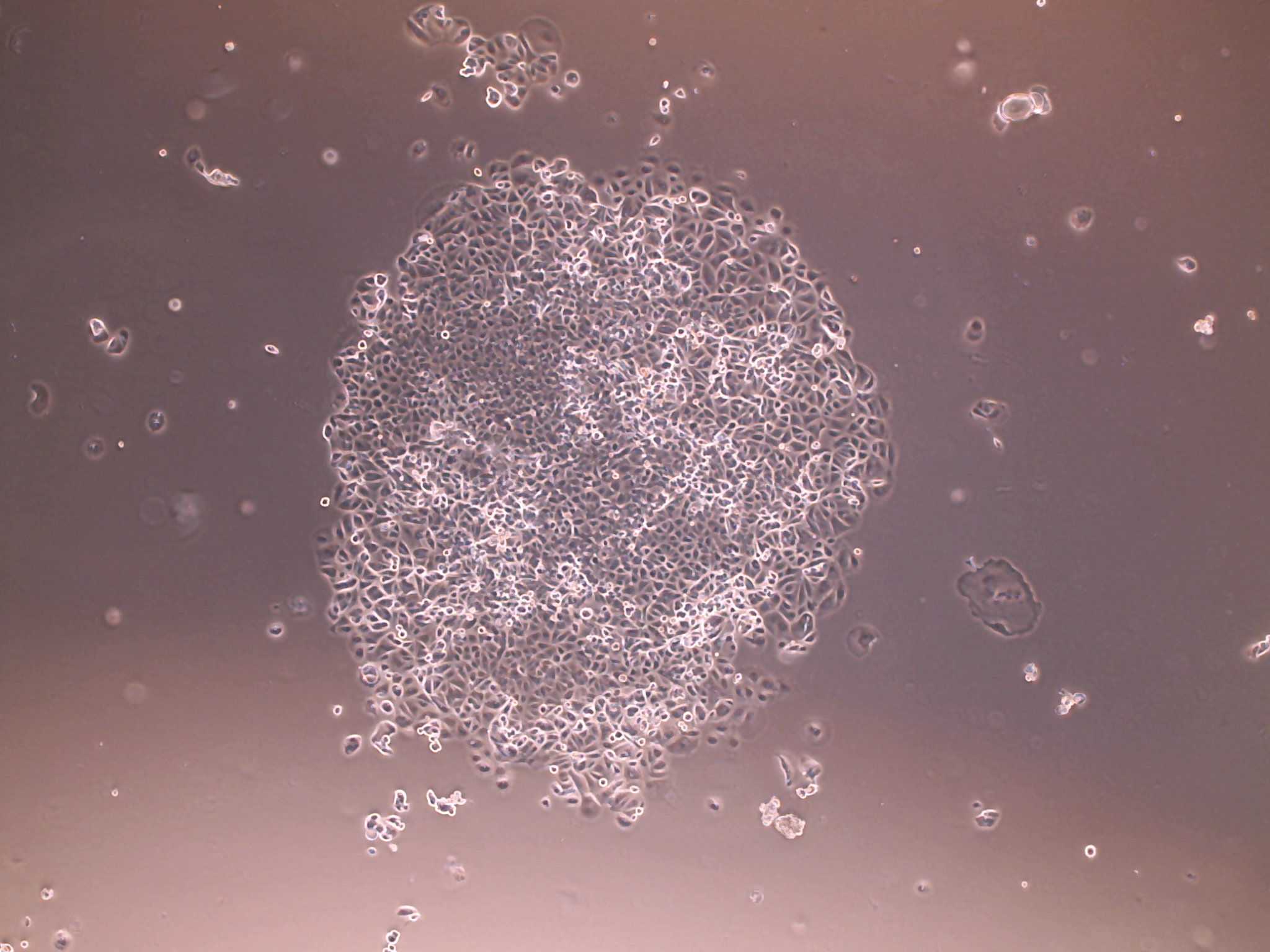

Supplement: Supplementary file 2 — Source data Fig. 1 [file 44318_2025_371_MOESM2_ESM.zip › SourceData_Figure 1/1D/mcf7 30dpt-3.tif]

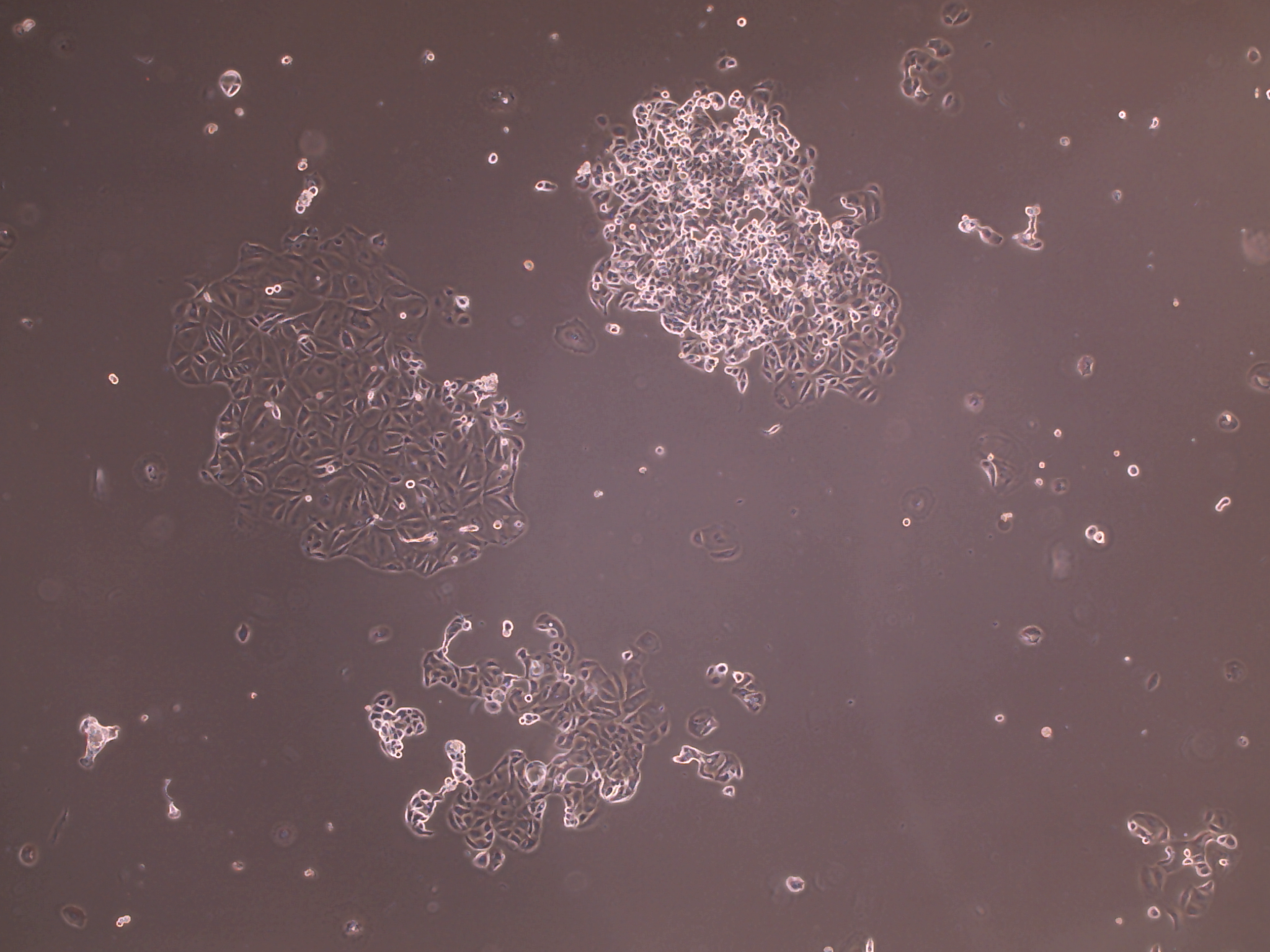

Supplement: Supplementary file 2 — Source data Fig. 1 [file 44318_2025_371_MOESM2_ESM.zip › SourceData_Figure 1/1D/mcf7 30dpt-1.tif]

1  $\mu$ m Abemaciclib  $\rightarrow$

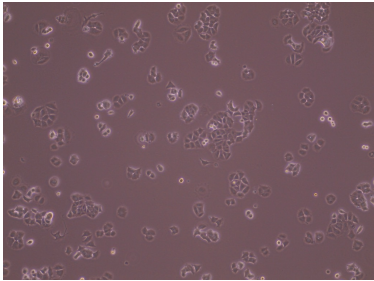

Day 0

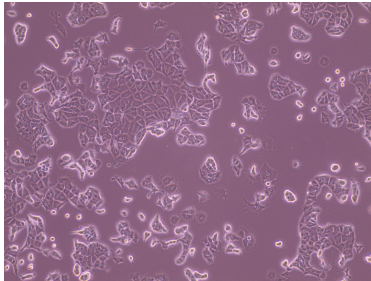

Day 2

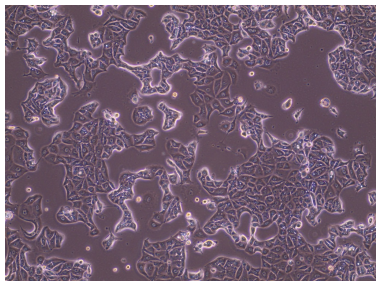

Day 5

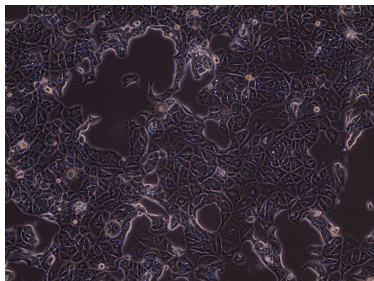

Day 9

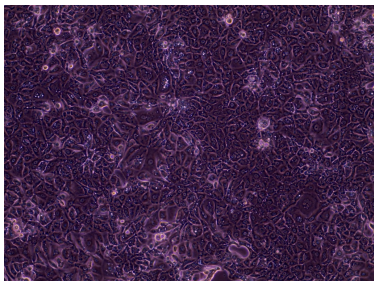

Day 12

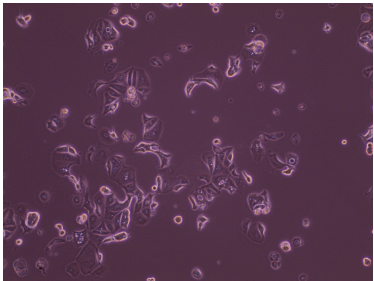

Day 20  
(Split\*)

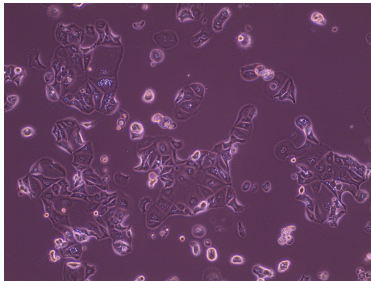

Day 27

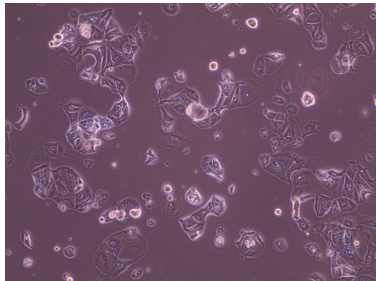

Day 30

Supplement: Supplementary file 2 — Source data Fig. 1 [file 44318_2025_371_MOESM2_ESM.zip › SourceData_Figure 1/1D/mcf7_During abemaciclib treatment.pdf]

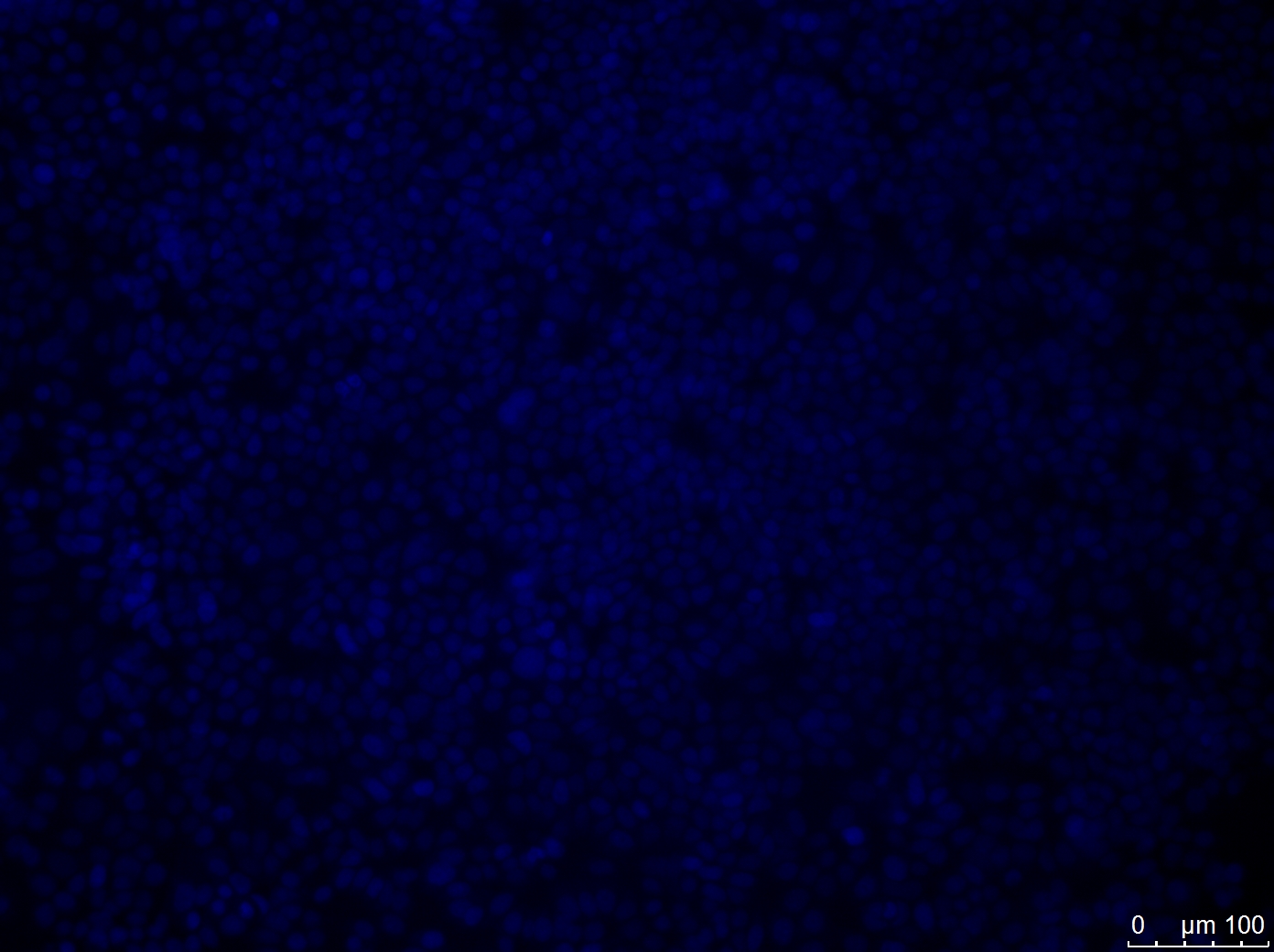

Supplement: Supplementary file 2 — Source data Fig. 1 [file 44318_2025_371_MOESM2_ESM.zip › SourceData_Figure 1/1C/Experiment_MCF7 VEH-1 DAPI.jpg]

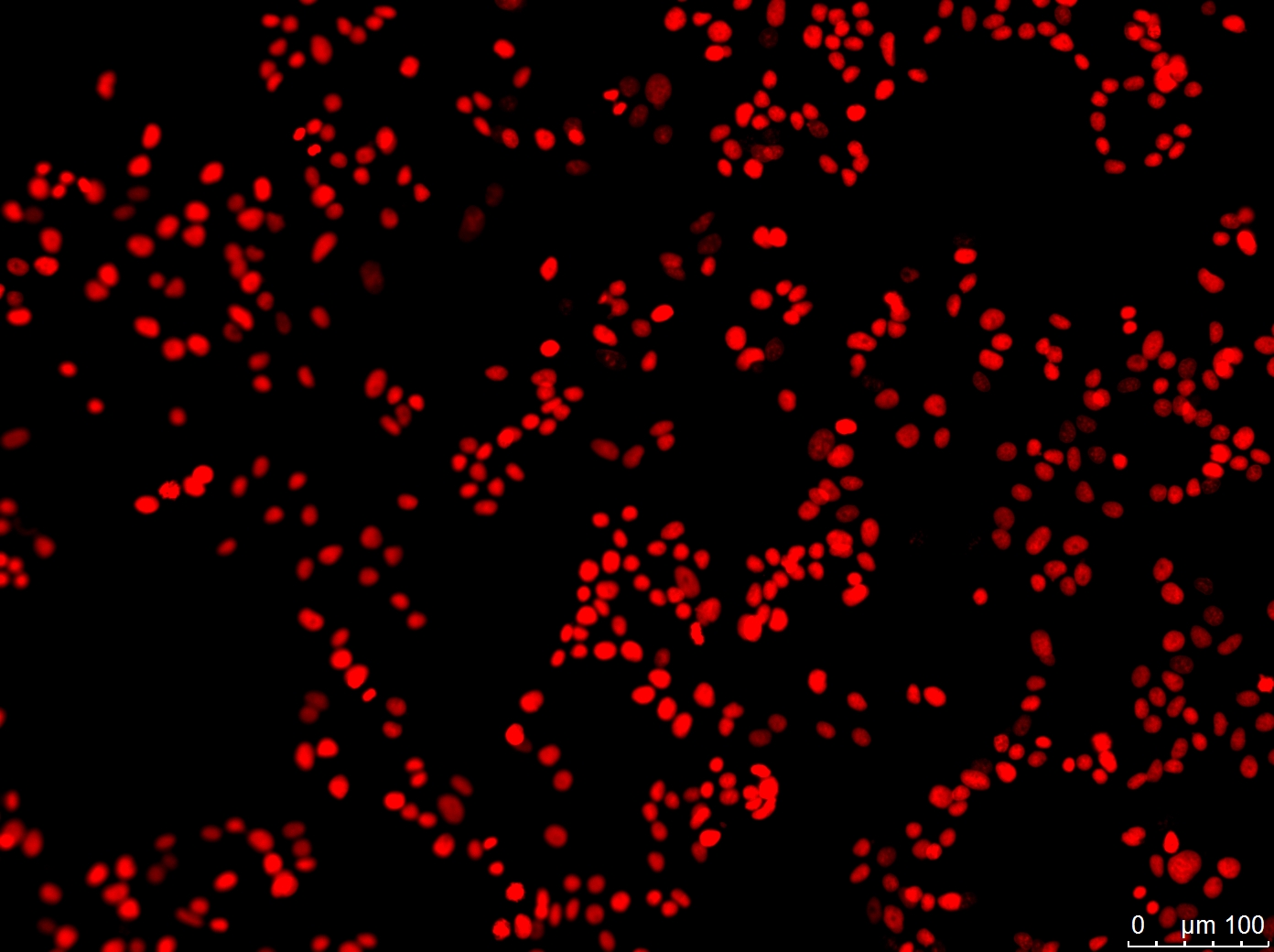

Supplement: Supplementary file 2 — Source data Fig. 1 [file 44318_2025_371_MOESM2_ESM.zip › SourceData_Figure 1/1C/Experiment_MCF7 VEH-3 EdU.jpg]

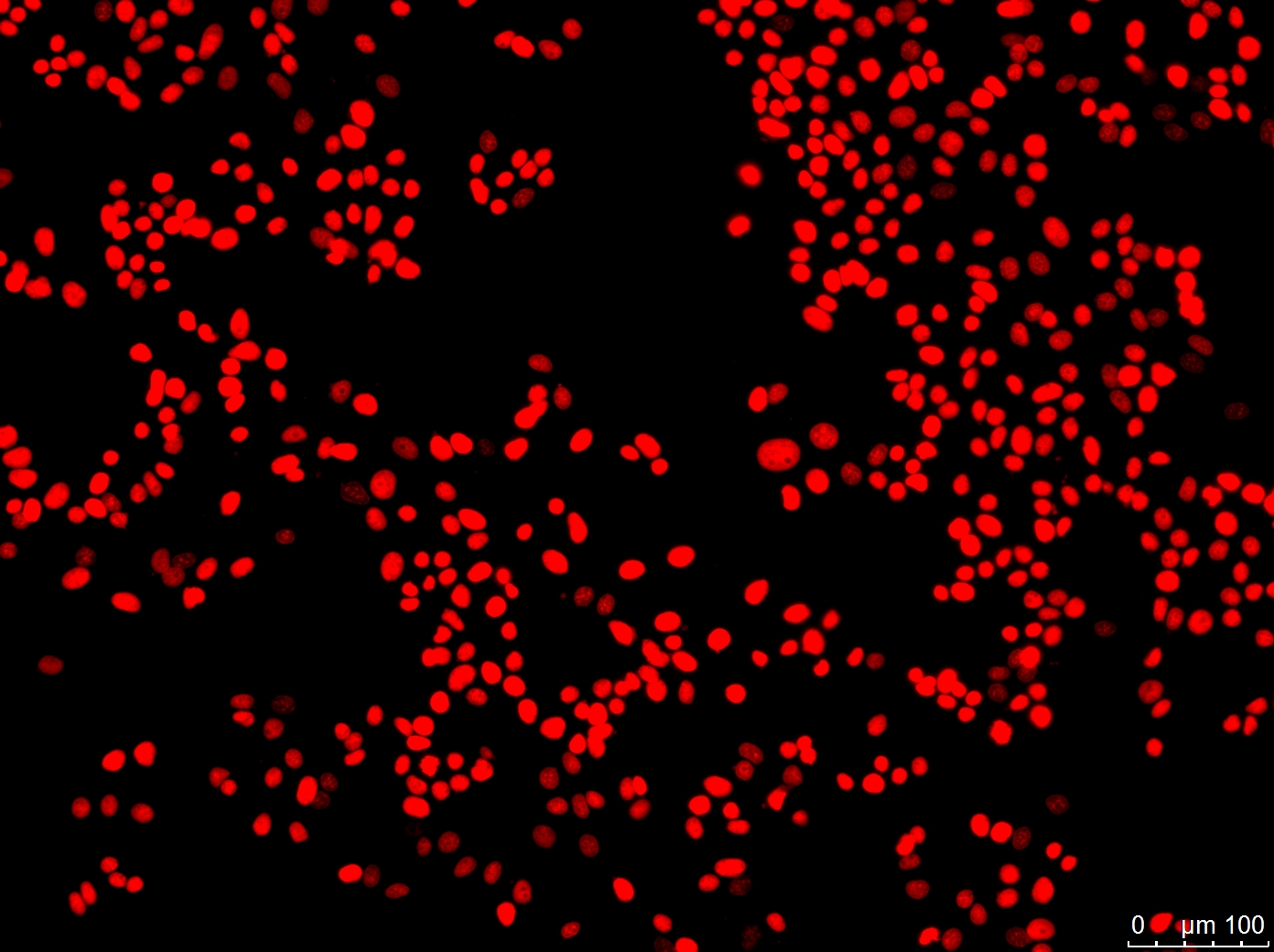

Supplement: Supplementary file 2 — Source data Fig. 1 [file 44318_2025_371_MOESM2_ESM.zip › SourceData_Figure 1/1C/Experiment_MCF7 VEH-2 EdU.jpg]

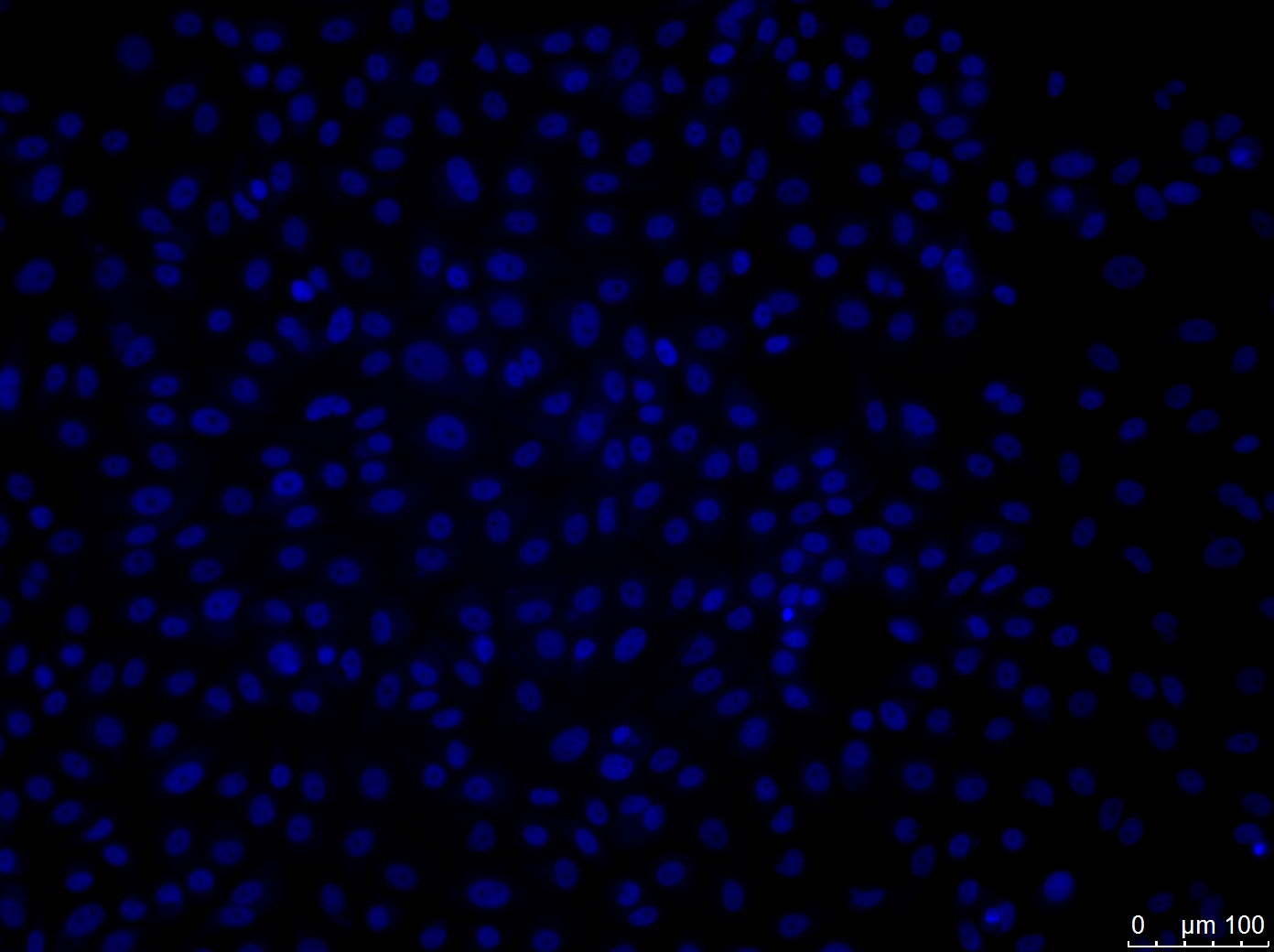

Supplement: Supplementary file 2 — Source data Fig. 1 [file 44318_2025_371_MOESM2_ESM.zip › SourceData_Figure 1/1C/Experiment_MCF7 ABEMA-1 DAPI.jpg]

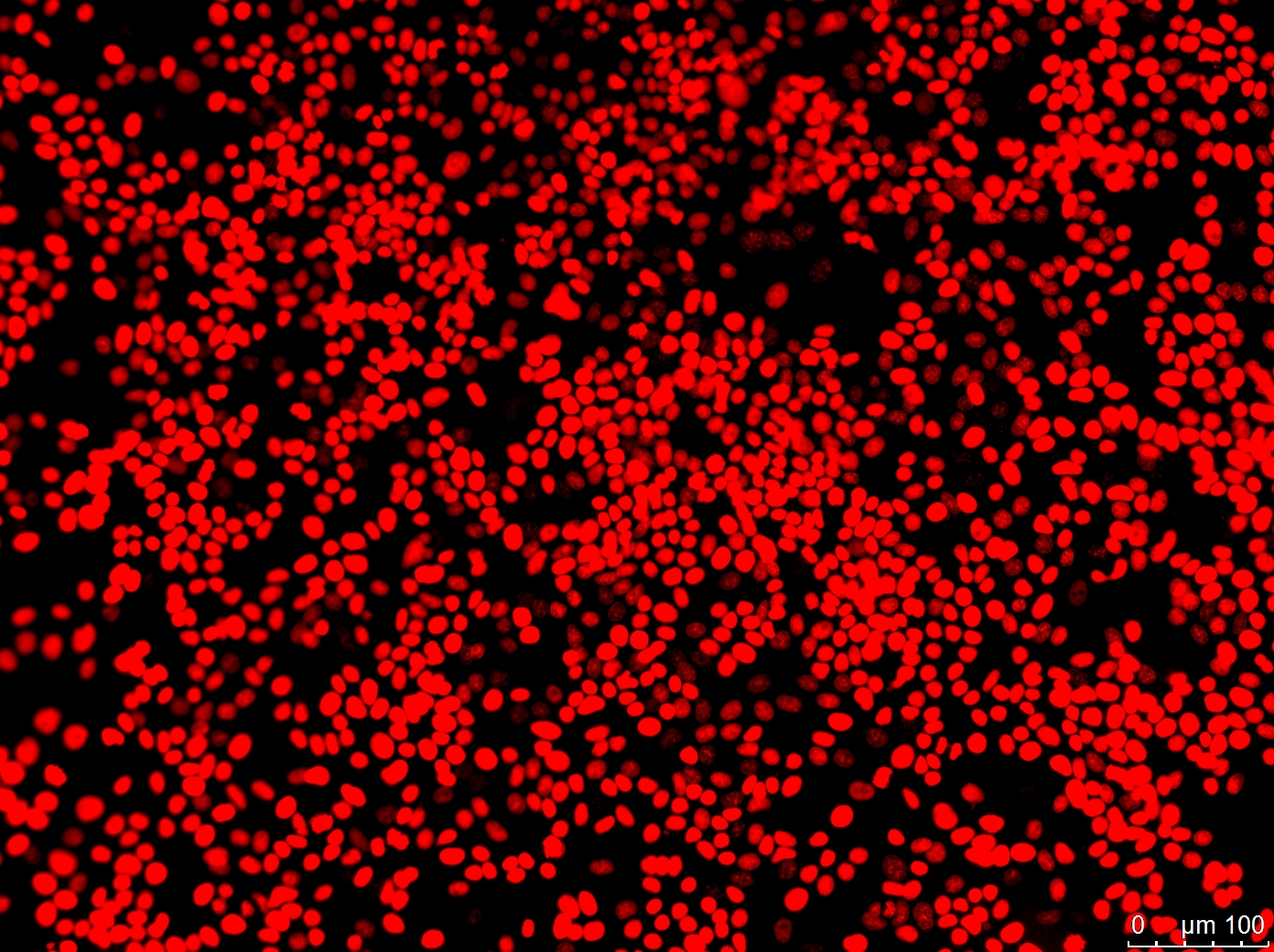

Supplement: Supplementary file 2 — Source data Fig. 1 [file 44318_2025_371_MOESM2_ESM.zip › SourceData_Figure 1/1C/Experiment_MCF7 VEH-1 EdU.jpg]

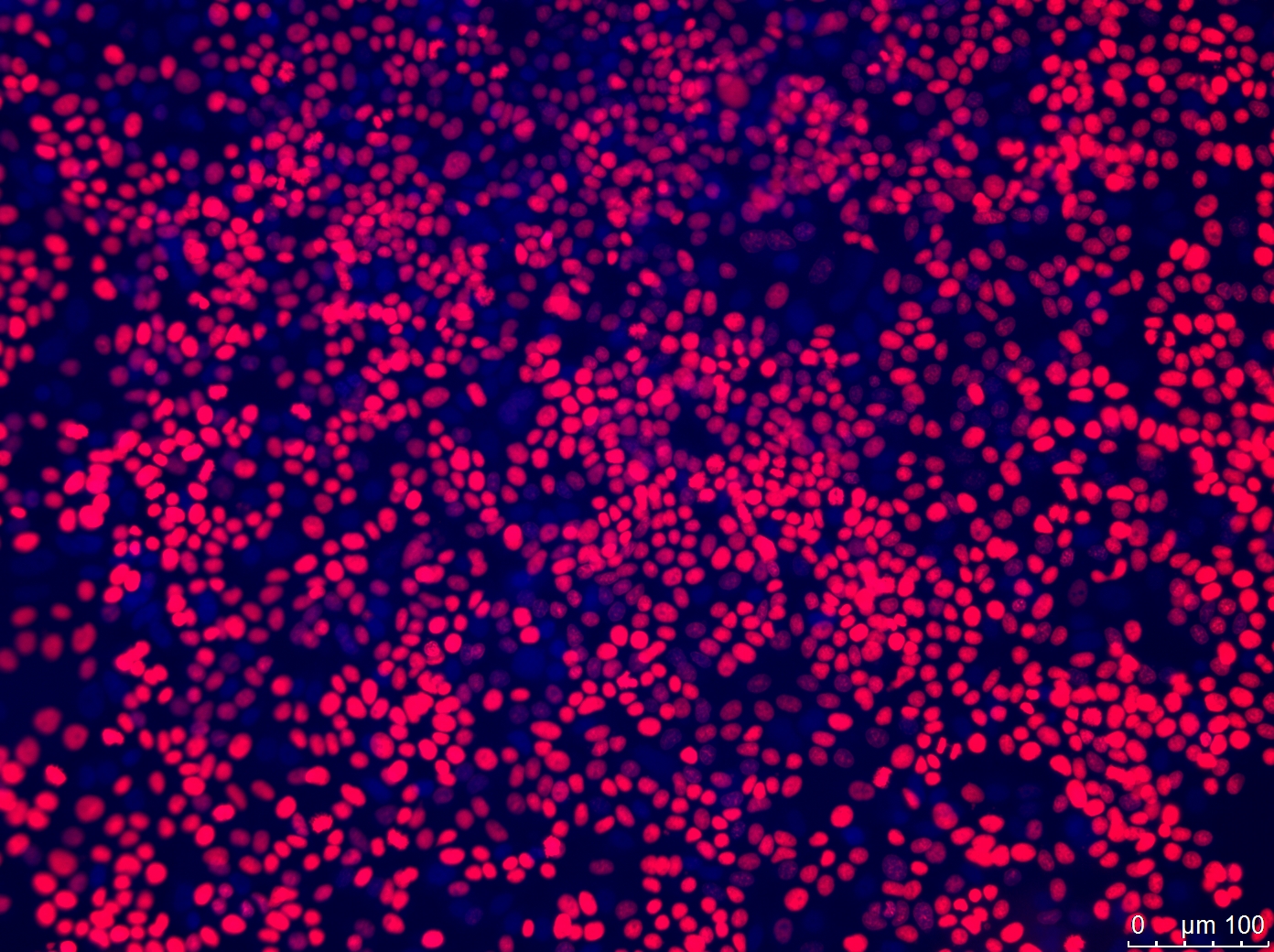

Supplement: Supplementary file 2 — Source data Fig. 1 [file 44318_2025_371_MOESM2_ESM.zip › SourceData_Figure 1/1C/Experiment_MCF7 VEH-1.jpg]

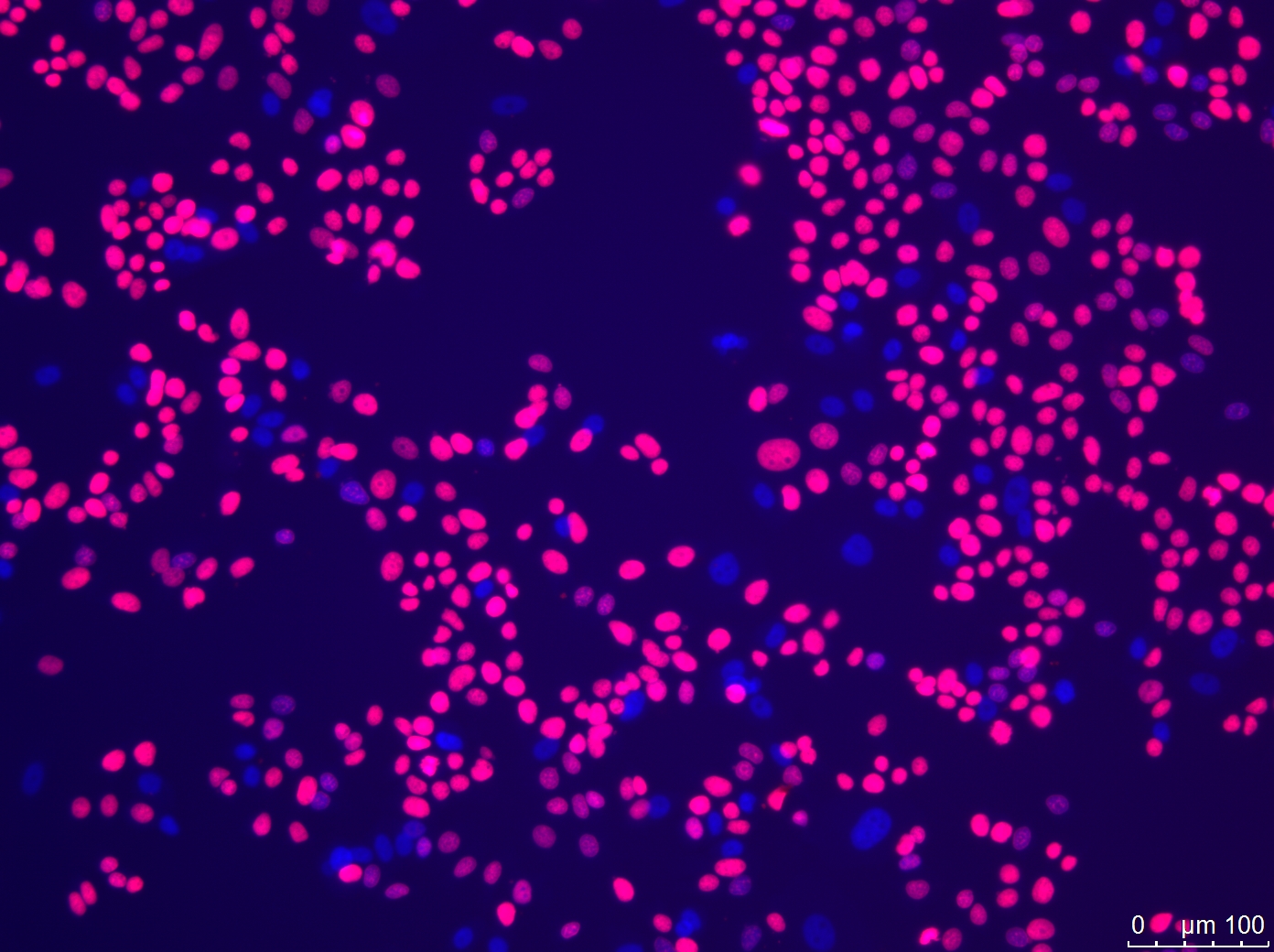

Supplement: Supplementary file 2 — Source data Fig. 1 [file 44318_2025_371_MOESM2_ESM.zip › SourceData_Figure 1/1C/Experiment_MCF7 VEH-2.jpg]

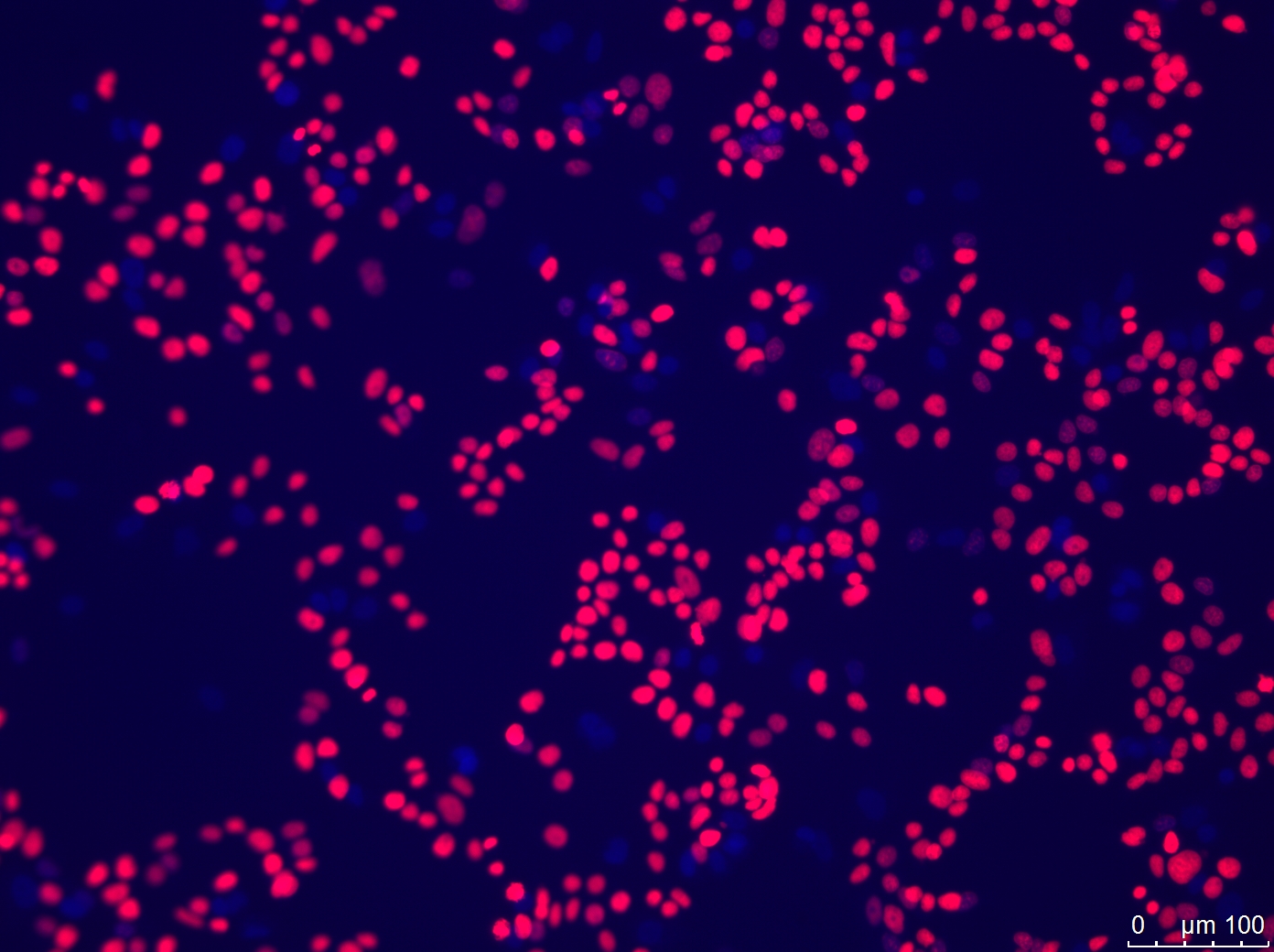

Supplement: Supplementary file 2 — Source data Fig. 1 [file 44318_2025_371_MOESM2_ESM.zip › SourceData_Figure 1/1C/Experiment_MCF7 VEH-3.jpg]

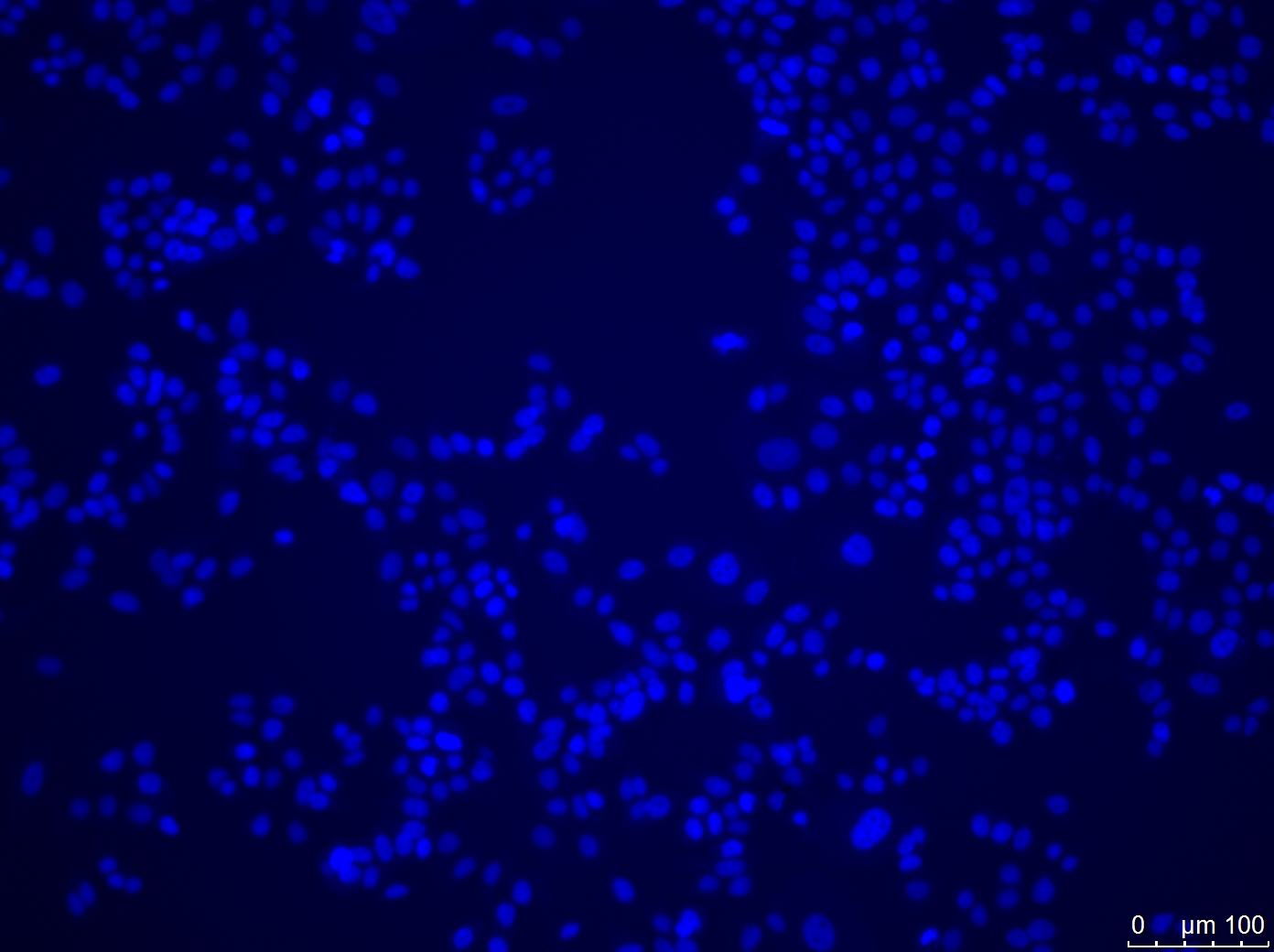

Supplement: Supplementary file 2 — Source data Fig. 1 [file 44318_2025_371_MOESM2_ESM.zip › SourceData_Figure 1/1C/Experiment_MCF7 VEH-2 DAPI.jpg]

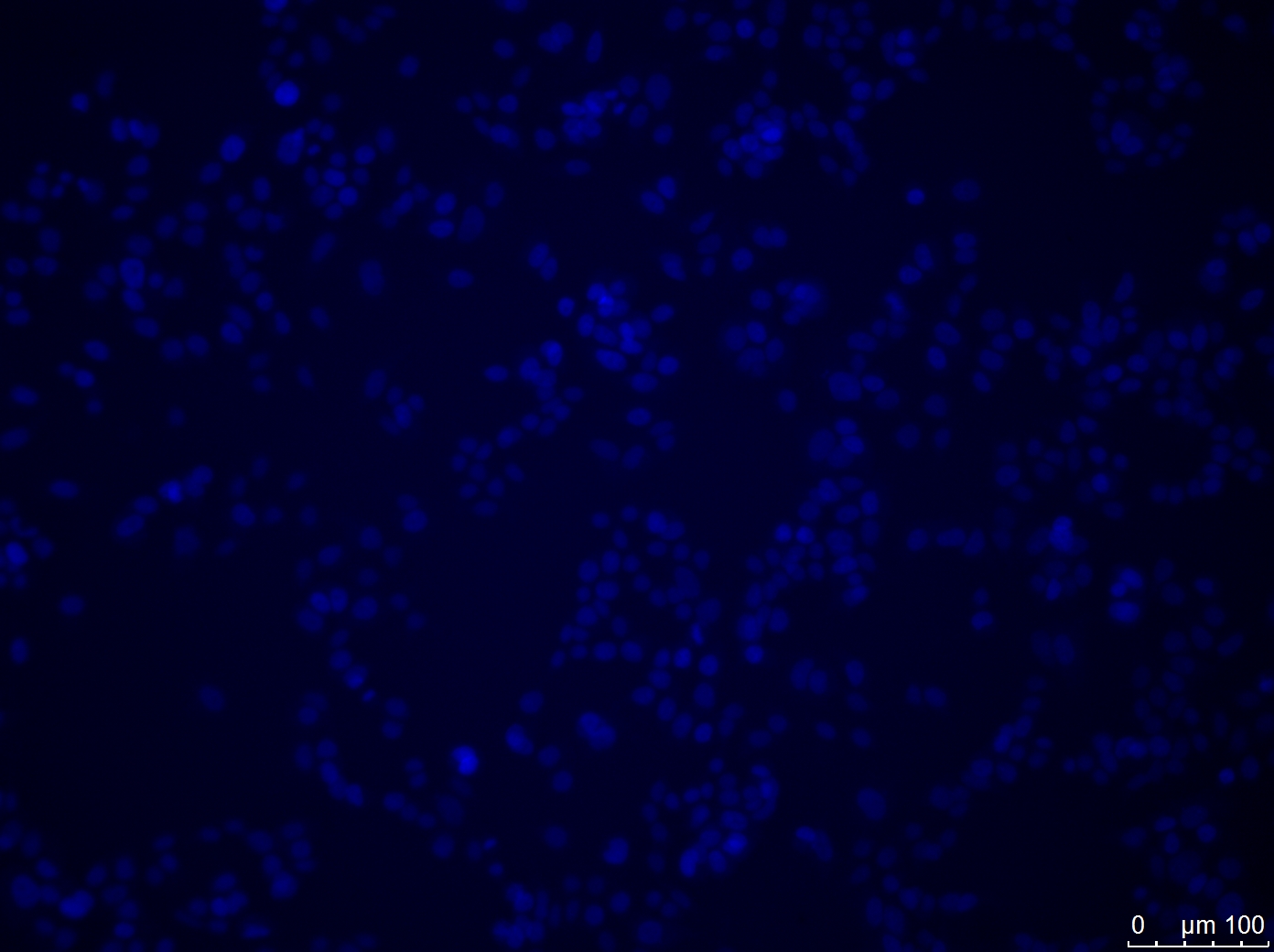

Supplement: Supplementary file 2 — Source data Fig. 1 [file 44318_2025_371_MOESM2_ESM.zip › SourceData_Figure 1/1C/Experiment_MCF7 VEH-3 DAPI.jpg]

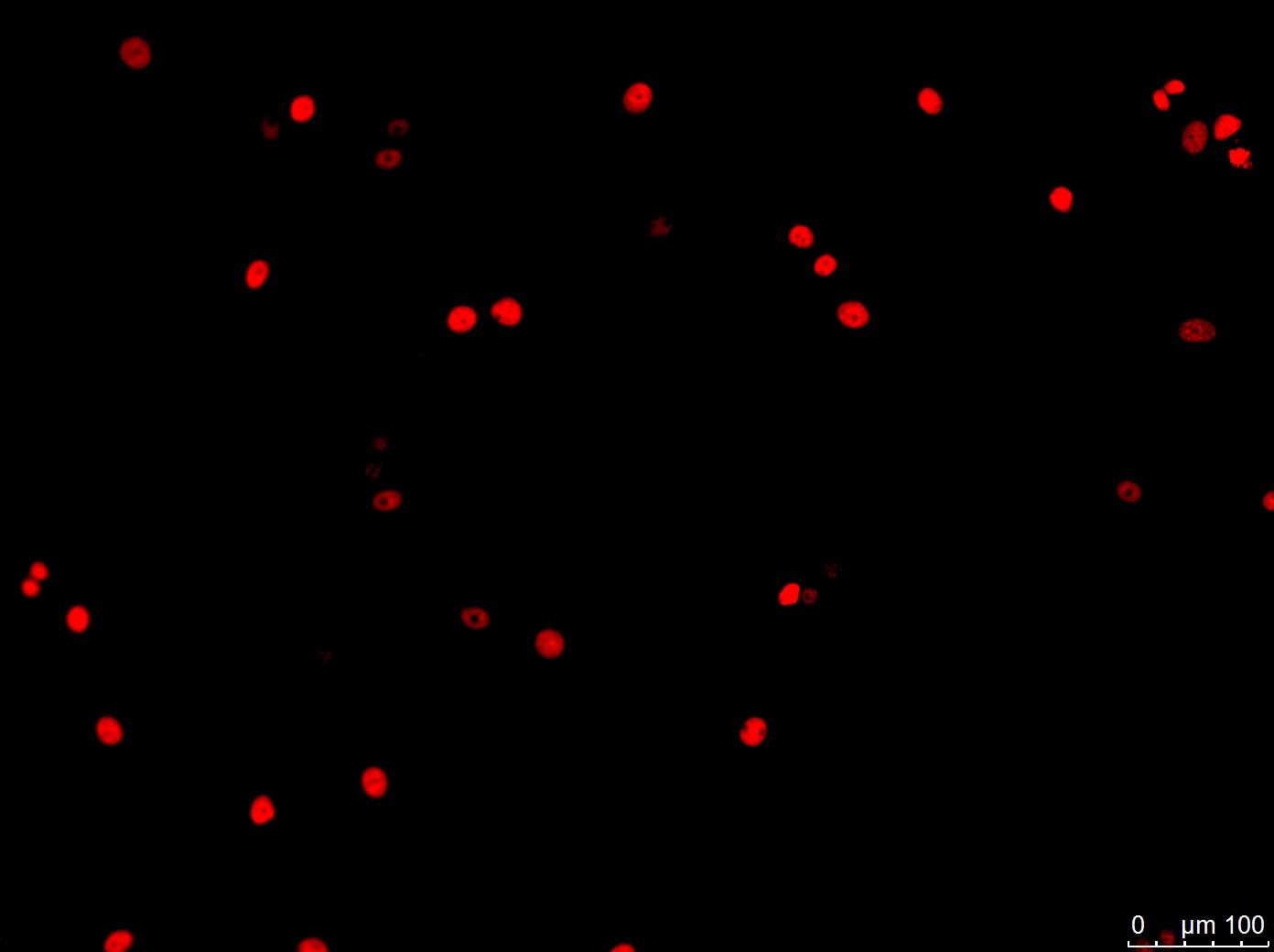

Supplement: Supplementary file 2 — Source data Fig. 1 [file 44318_2025_371_MOESM2_ESM.zip › SourceData_Figure 1/1C/Experiment_MCF7 ABEMA-1 EdU.jpg]

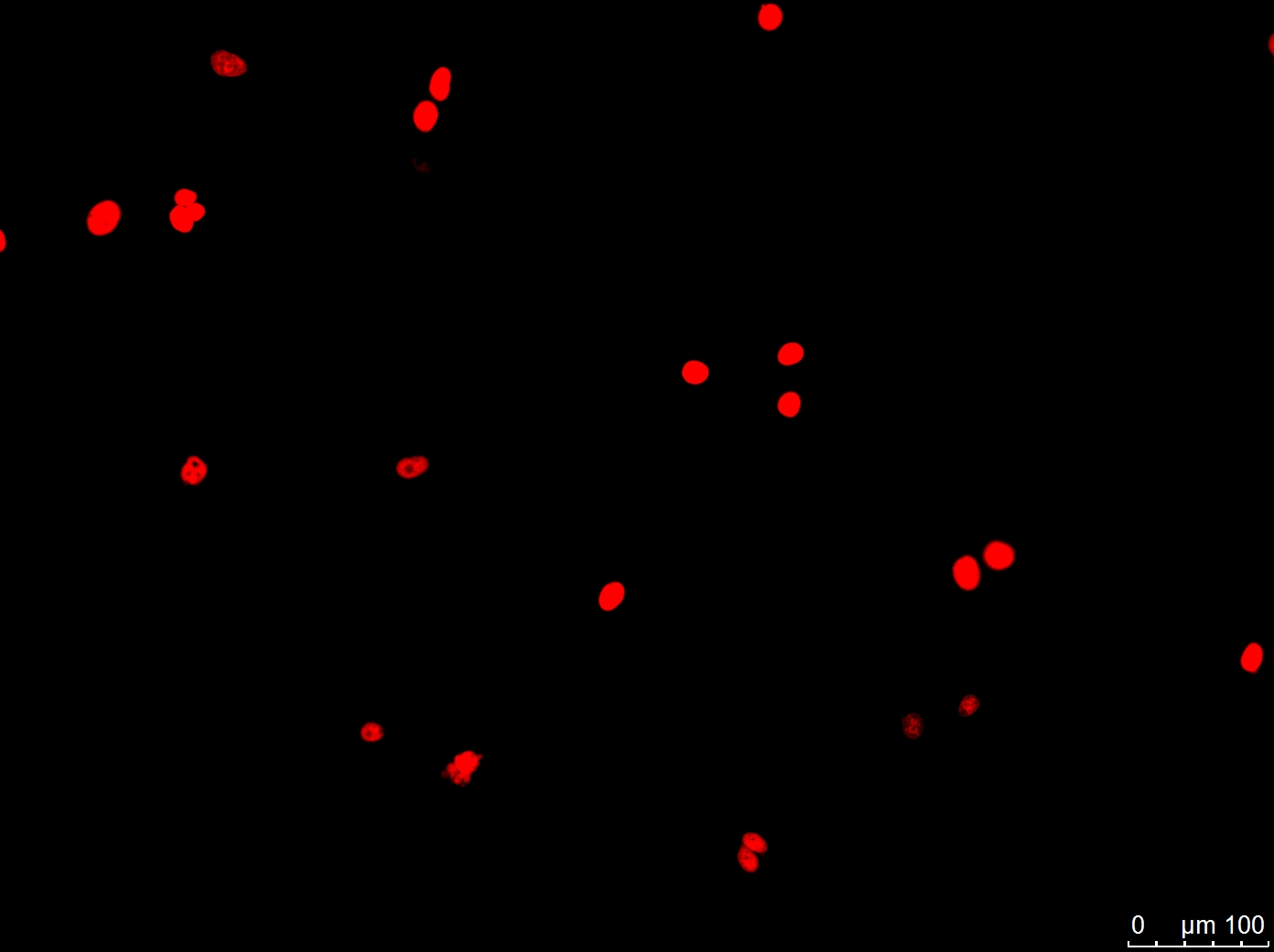

Supplement: Supplementary file 2 — Source data Fig. 1 [file 44318_2025_371_MOESM2_ESM.zip › SourceData_Figure 1/1C/Experiment_MCF7 ABEMA-3 EdU.jpg]

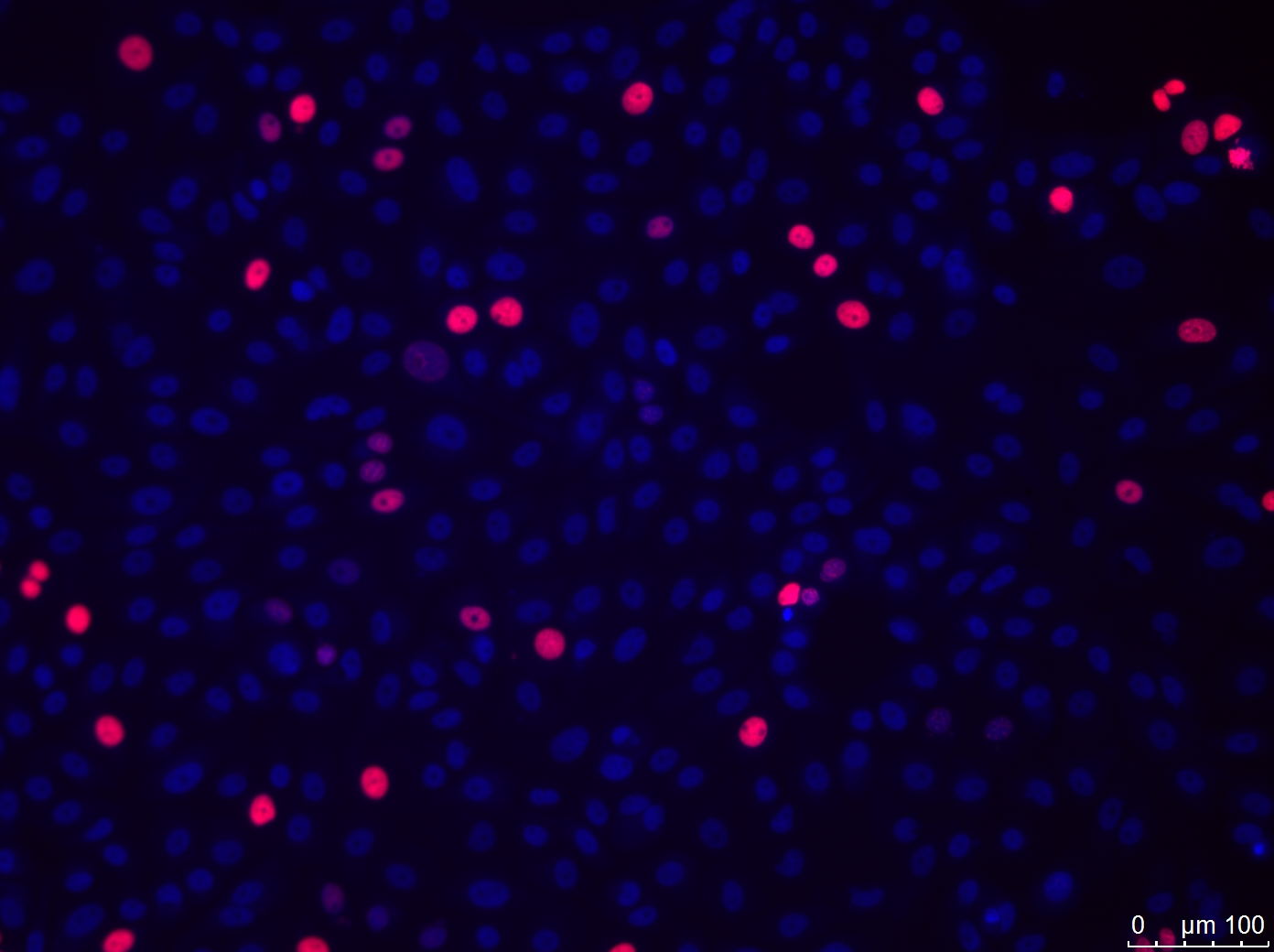

Supplement: Supplementary file 2 — Source data Fig. 1 [file 44318_2025_371_MOESM2_ESM.zip › SourceData_Figure 1/1C/Experiment_MCF7 ABEMA-1.jpg]

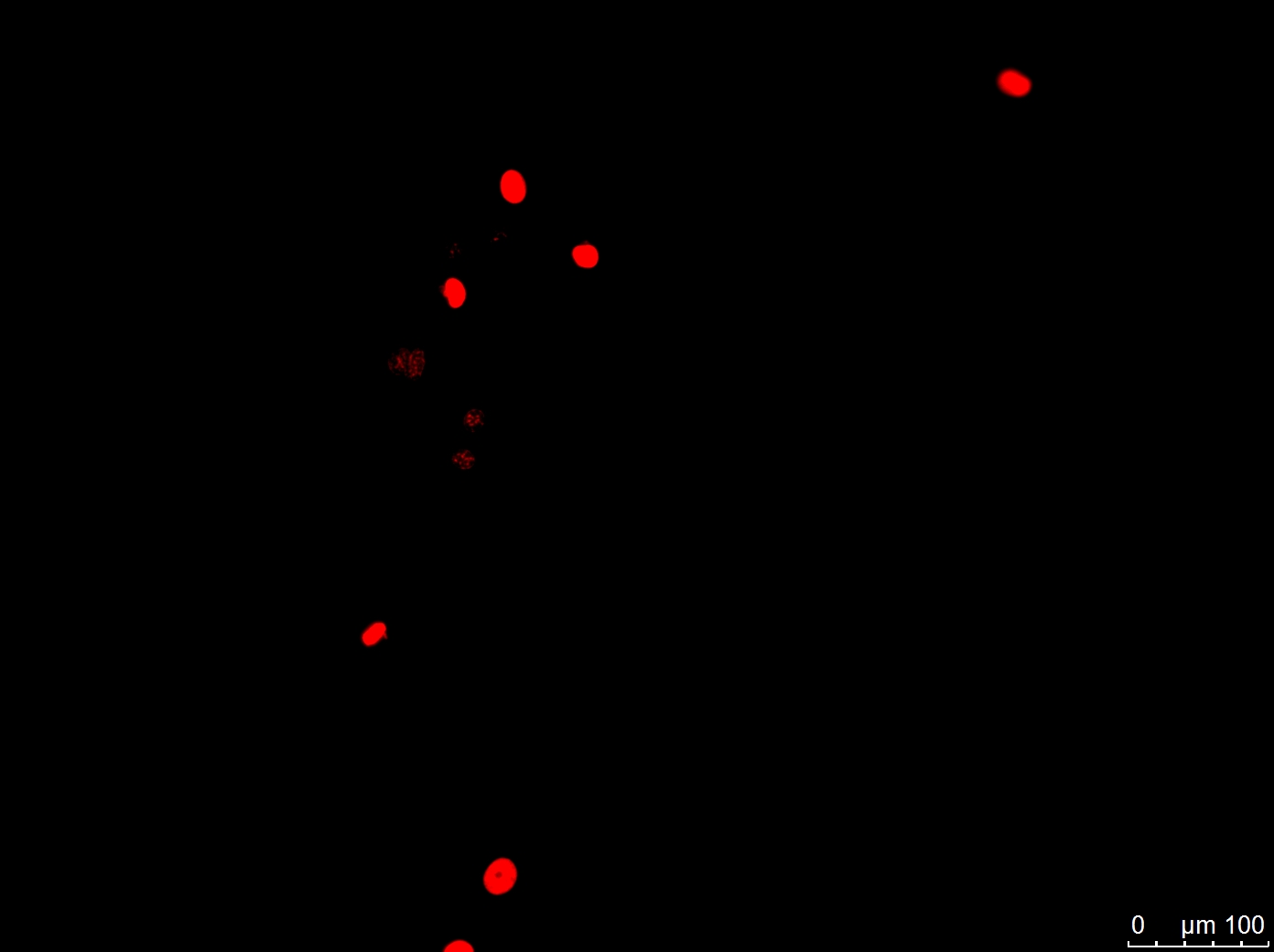

Supplement: Supplementary file 2 — Source data Fig. 1 [file 44318_2025_371_MOESM2_ESM.zip › SourceData_Figure 1/1C/Experiment_MCF7 ABEMA-2 EDU.jpg]

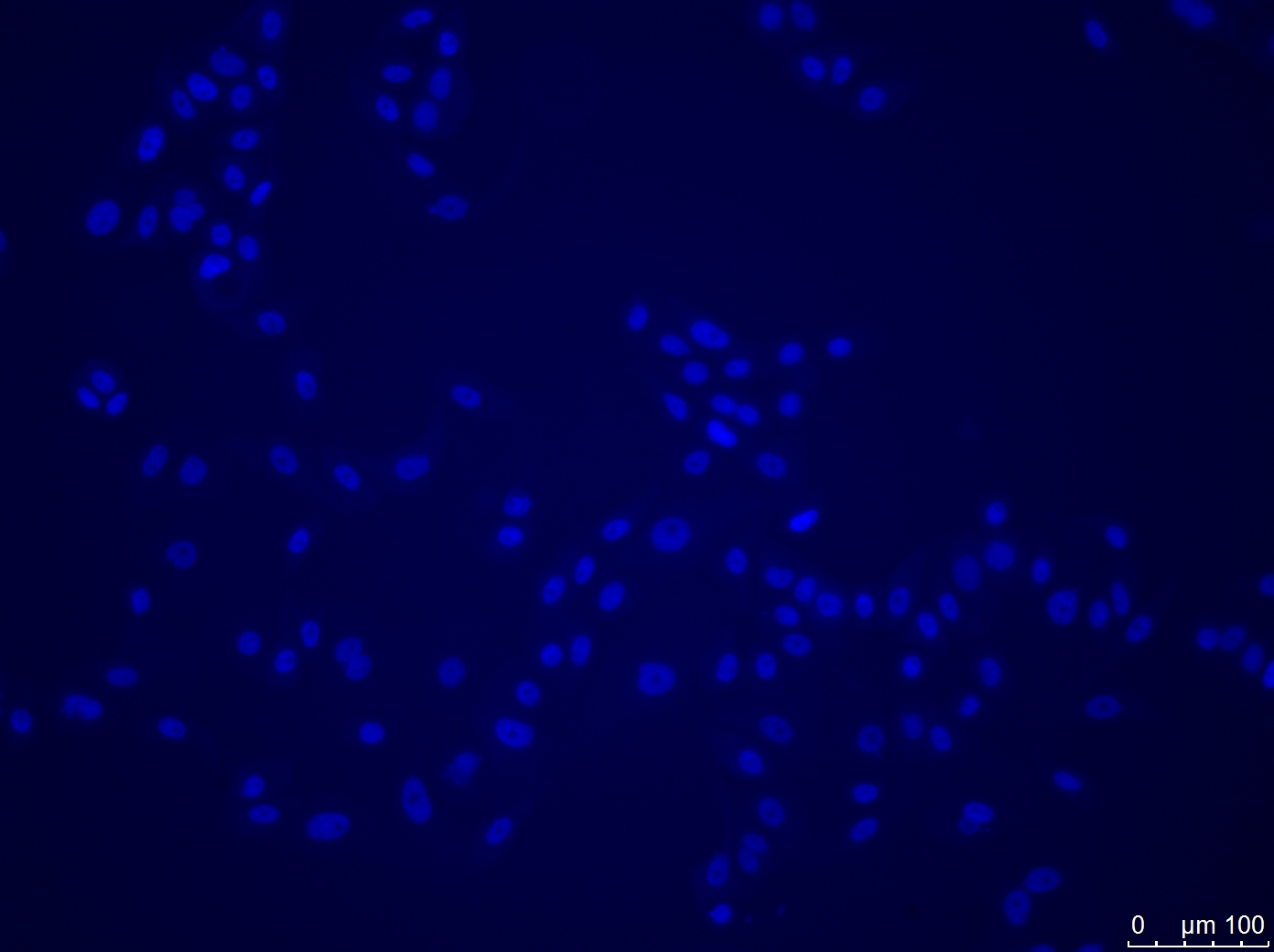

Supplement: Supplementary file 2 — Source data Fig. 1 [file 44318_2025_371_MOESM2_ESM.zip › SourceData_Figure 1/1C/Experiment_MCF7 ABEMA-3 DAPI.jpg]

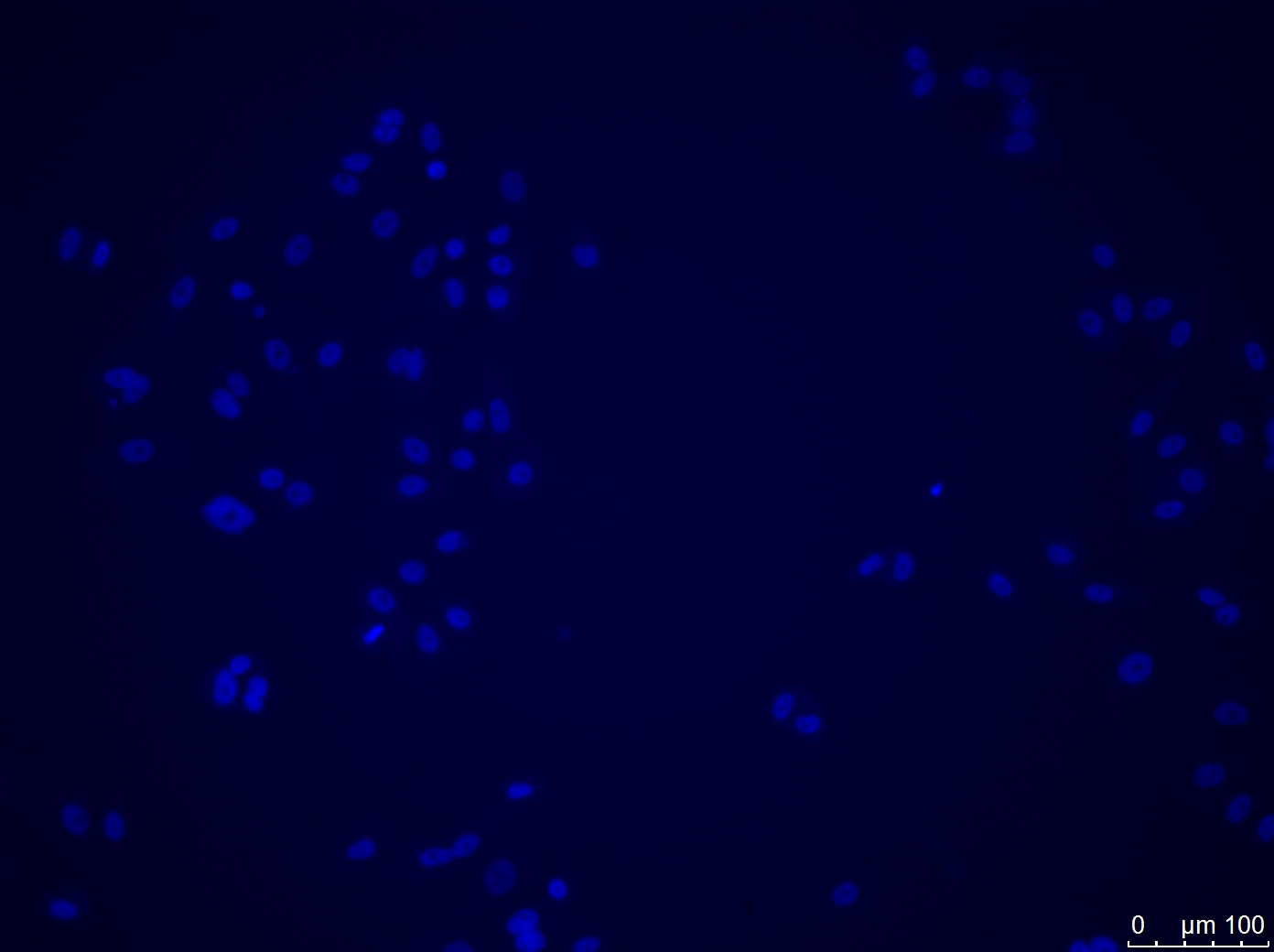

Supplement: Supplementary file 2 — Source data Fig. 1 [file 44318_2025_371_MOESM2_ESM.zip › SourceData_Figure 1/1C/Experiment_MCF7 ABEMA-2 DAPI.jpg]

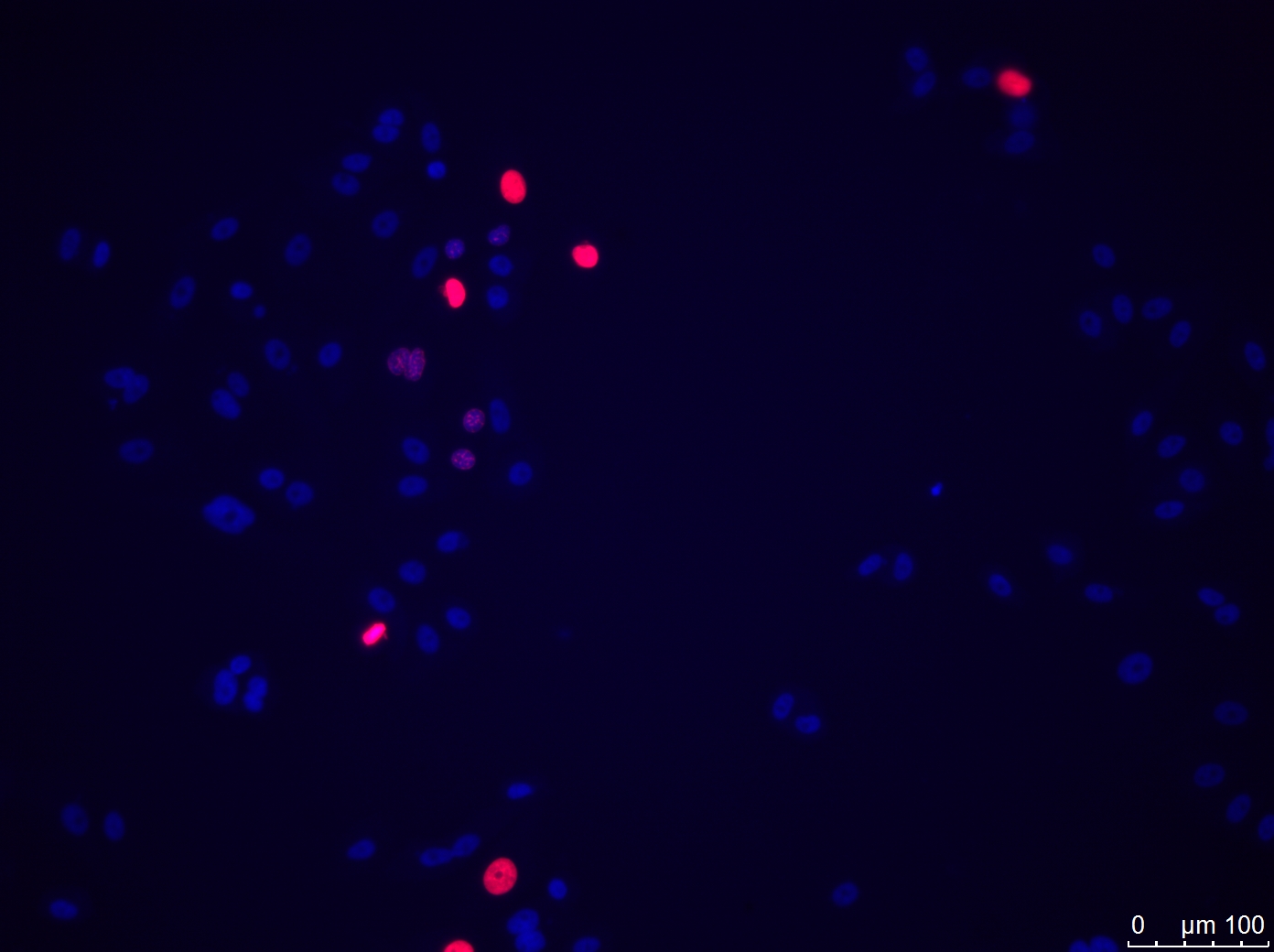

Supplement: Supplementary file 2 — Source data Fig. 1 [file 44318_2025_371_MOESM2_ESM.zip › SourceData_Figure 1/1C/Experiment_MCF7 ABEMA-2.jpg]

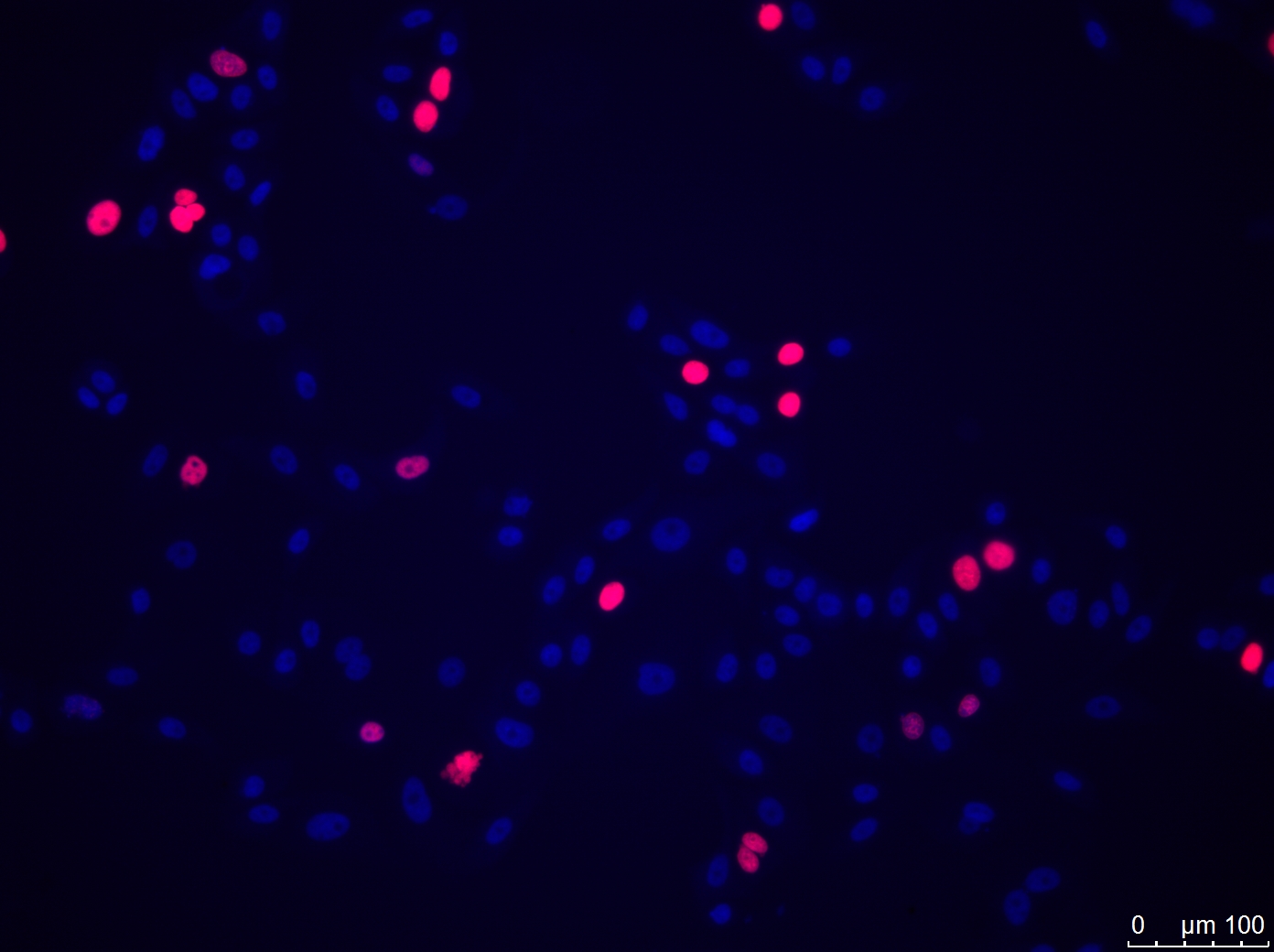

Supplement: Supplementary file 2 — Source data Fig. 1 [file 44318_2025_371_MOESM2_ESM.zip › SourceData_Figure 1/1C/Experiment_MCF7 ABEMA-3.jpg]

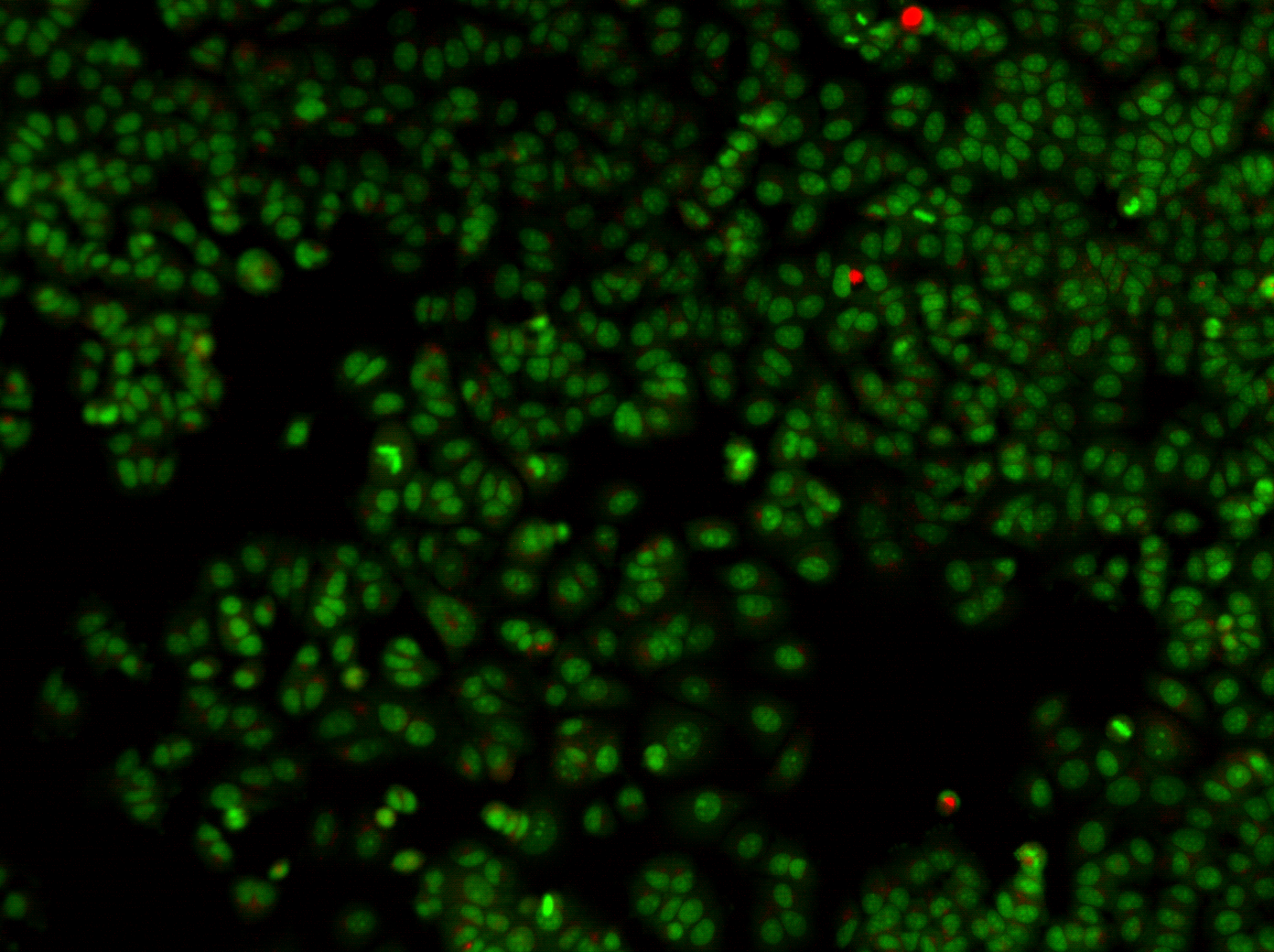

Supplement: Supplementary file 3 — Source data Fig. 2 [file 44318_2025_371_MOESM3_ESM.zip › SourceData_Figure 2/2D/Experiment.lif_Overlay-MCF7 control.tif]

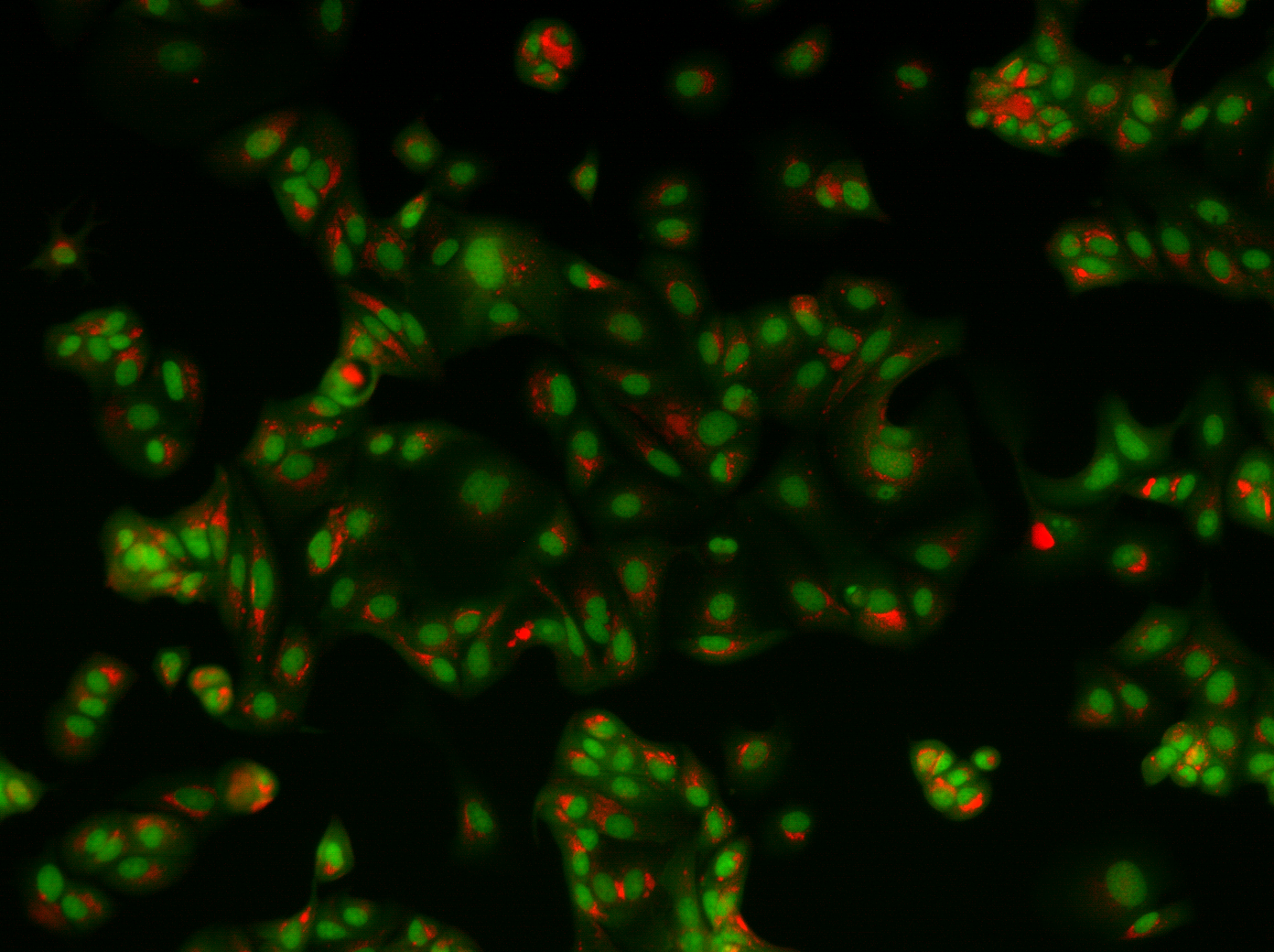

Supplement: Supplementary file 3 — Source data Fig. 2 [file 44318_2025_371_MOESM3_ESM.zip › SourceData_Figure 2/2D/Experiment.lif_Overlay-MCF7 Abema.tif]

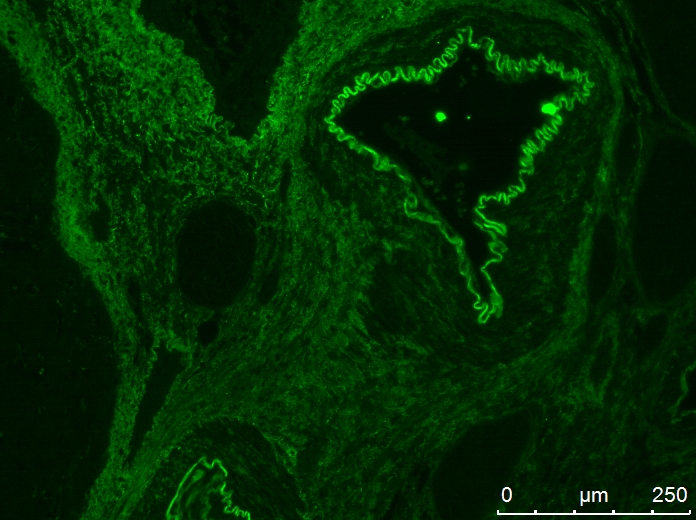

Supplement: Supplementary file 3 — Source data Fig. 2 [file 44318_2025_371_MOESM3_ESM.zip › SourceData_Figure 2/2M/Experiment_Post-2-1 LAMP1_22.5%.jpg]

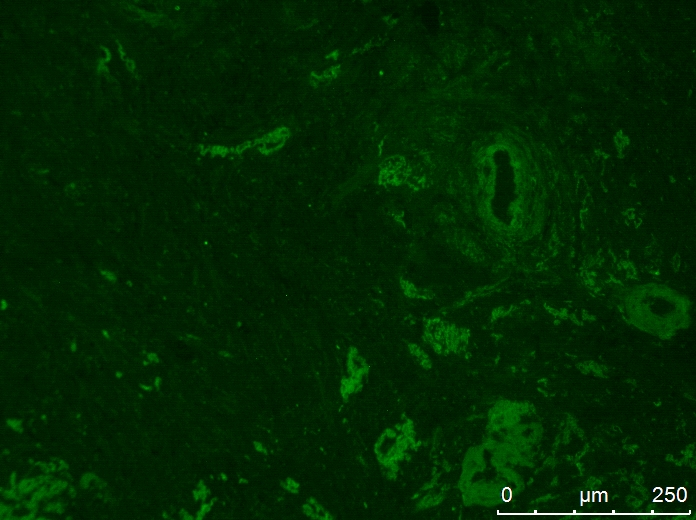

Supplement: Supplementary file 3 — Source data Fig. 2 [file 44318_2025_371_MOESM3_ESM.zip › SourceData_Figure 2/2M/Experiment_Pre-3-1 LAMP1_10%.jpg]

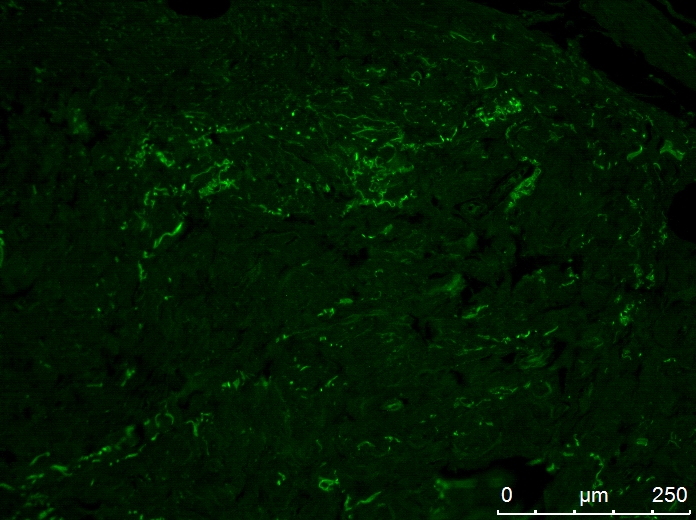

Supplement: Supplementary file 3 — Source data Fig. 2 [file 44318_2025_371_MOESM3_ESM.zip › SourceData_Figure 2/2M/Experiment_Post-1-2 LAMP1_5.406%.jpg]

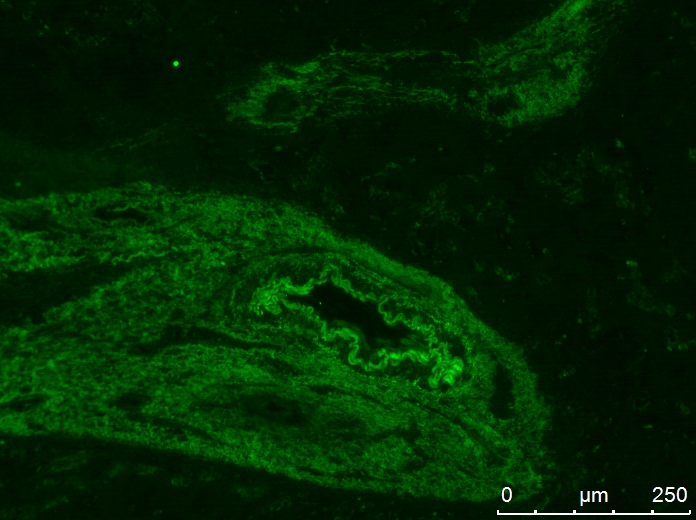

Supplement: Supplementary file 3 — Source data Fig. 2 [file 44318_2025_371_MOESM3_ESM.zip › SourceData_Figure 2/2M/Experiment_Post-3-1 LAMP1_24.8%.jpg]

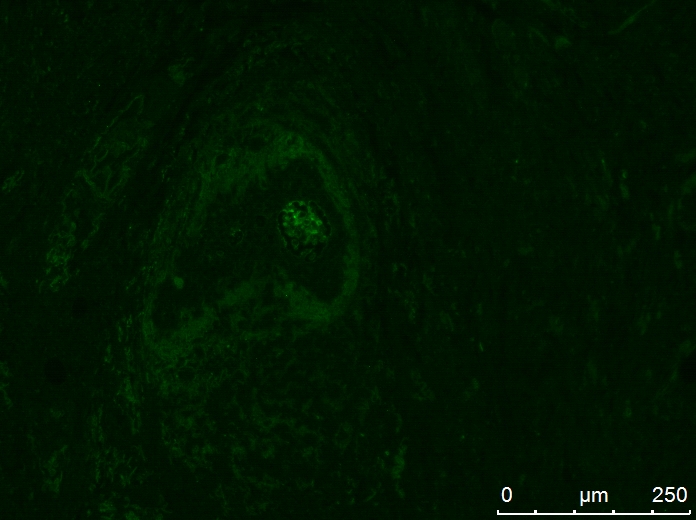

Supplement: Supplementary file 3 — Source data Fig. 2 [file 44318_2025_371_MOESM3_ESM.zip › SourceData_Figure 2/2M/Experiment_Pre-2-2 LAMP1_0.522%.jpg]

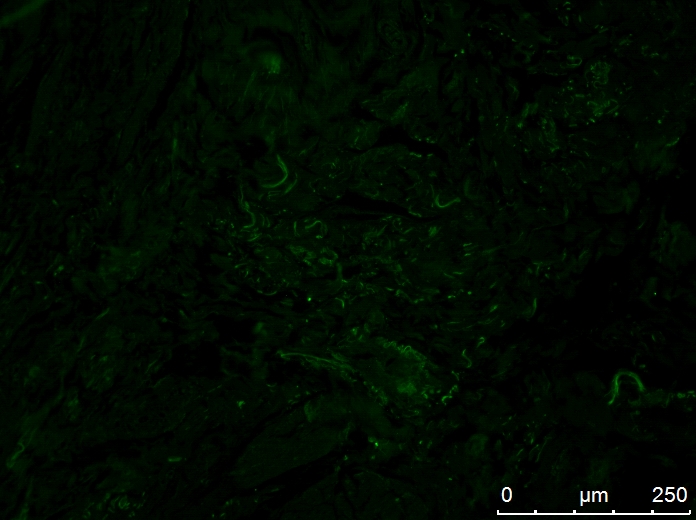

Supplement: Supplementary file 3 — Source data Fig. 2 [file 44318_2025_371_MOESM3_ESM.zip › SourceData_Figure 2/2M/Experiment_Post-1-3 LAMP1.jpg]

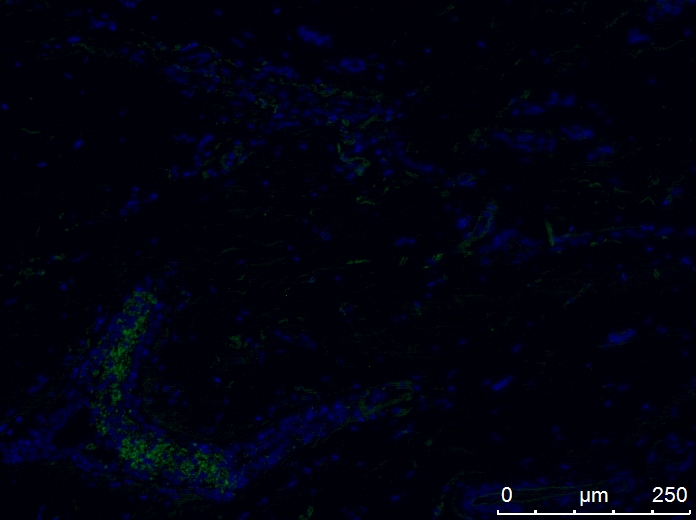

Supplement: Supplementary file 3 — Source data Fig. 2 [file 44318_2025_371_MOESM3_ESM.zip › SourceData_Figure 2/2M/Experiment_Post-1-1.jpg]

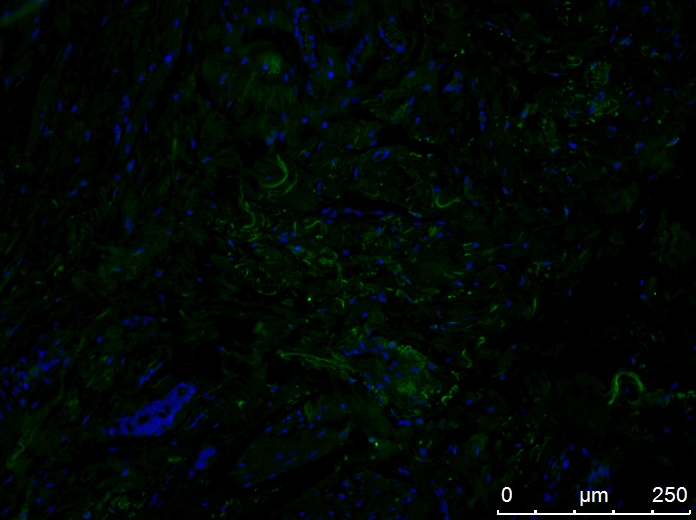

Supplement: Supplementary file 3 — Source data Fig. 2 [file 44318_2025_371_MOESM3_ESM.zip › SourceData_Figure 2/2M/Experiment_Post-1-3.jpg]

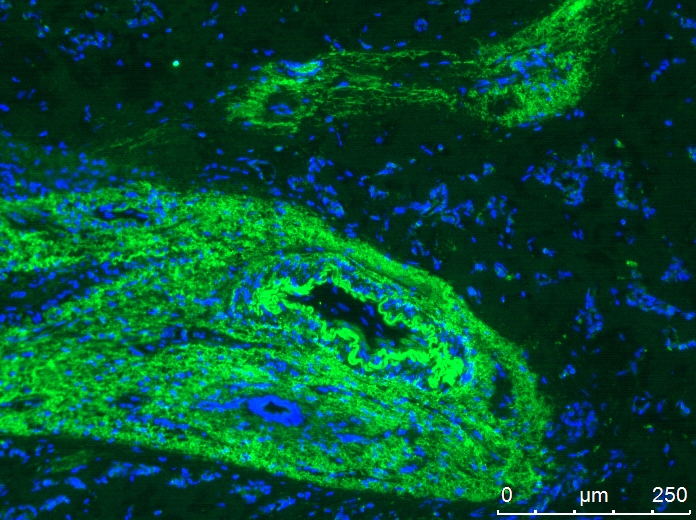

Supplement: Supplementary file 3 — Source data Fig. 2 [file 44318_2025_371_MOESM3_ESM.zip › SourceData_Figure 2/2M/Experiment_Post-3-1.jpg]

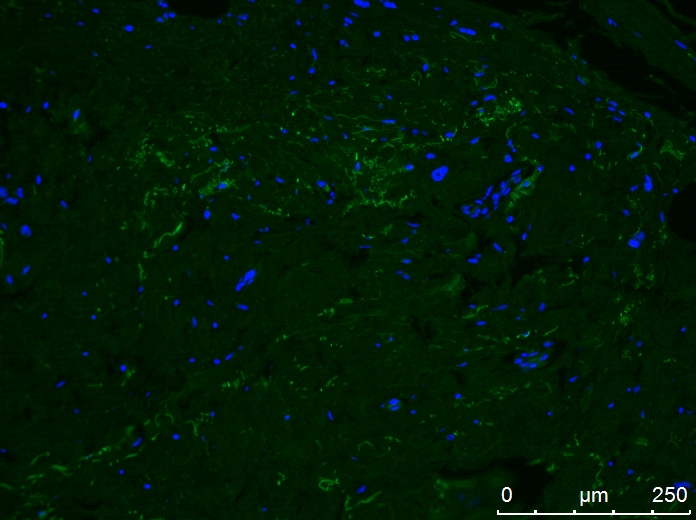

Supplement: Supplementary file 3 — Source data Fig. 2 [file 44318_2025_371_MOESM3_ESM.zip › SourceData_Figure 2/2M/Experiment_Post-1-2.jpg]

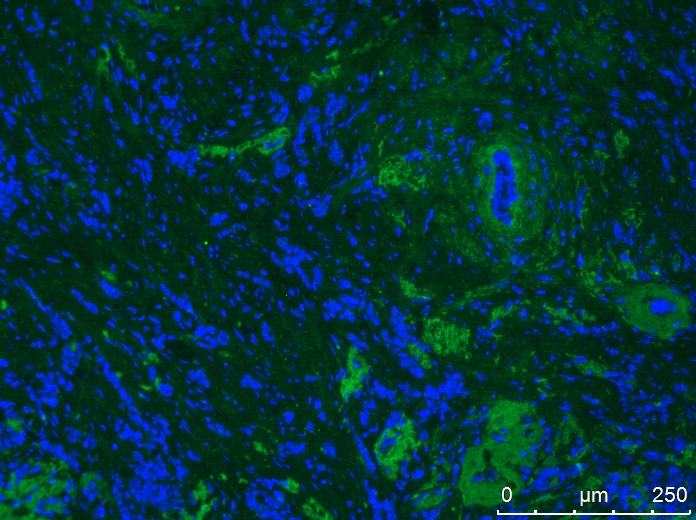

Supplement: Supplementary file 3 — Source data Fig. 2 [file 44318_2025_371_MOESM3_ESM.zip › SourceData_Figure 2/2M/Experiment_Pre-3-1.jpg]

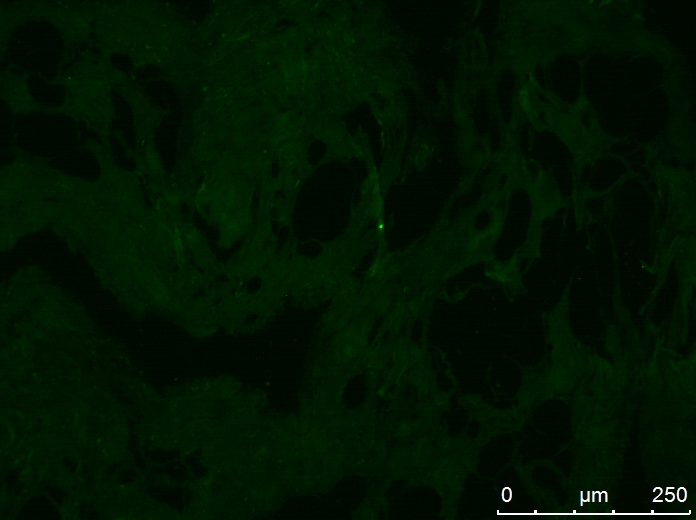

Supplement: Supplementary file 3 — Source data Fig. 2 [file 44318_2025_371_MOESM3_ESM.zip › SourceData_Figure 2/2M/Experiment_Pre-1-1 LAMP1_0.29%.jpg]

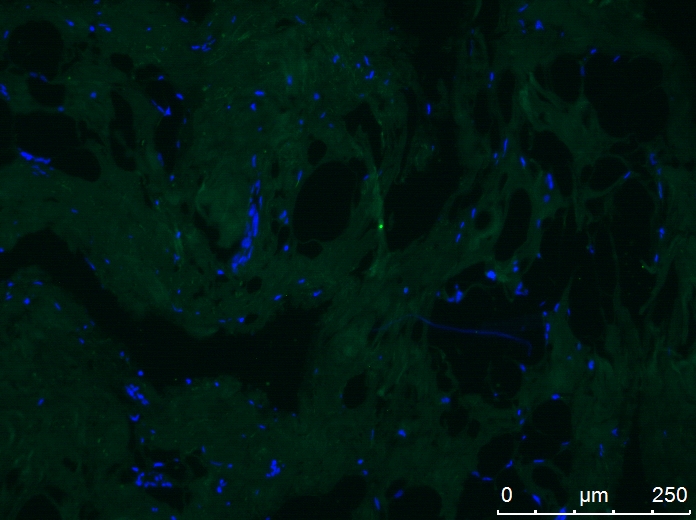

Supplement: Supplementary file 3 — Source data Fig. 2 [file 44318_2025_371_MOESM3_ESM.zip › SourceData_Figure 2/2M/Experiment_Pre-1-1.jpg]

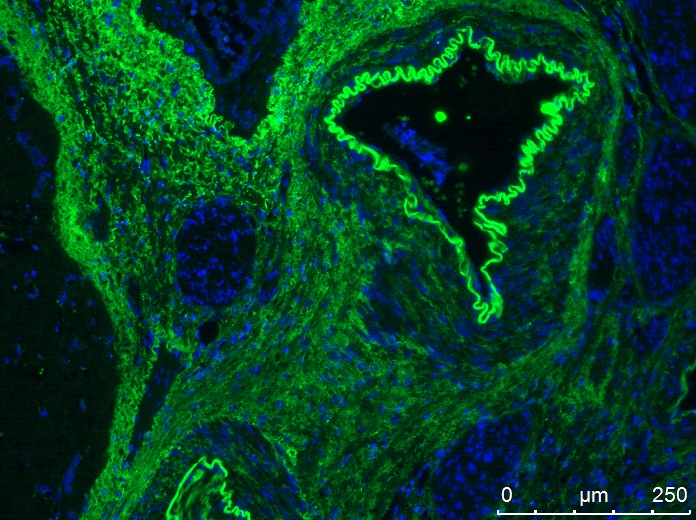

Supplement: Supplementary file 3 — Source data Fig. 2 [file 44318_2025_371_MOESM3_ESM.zip › SourceData_Figure 2/2M/Experiment_Post-2-1.jpg]

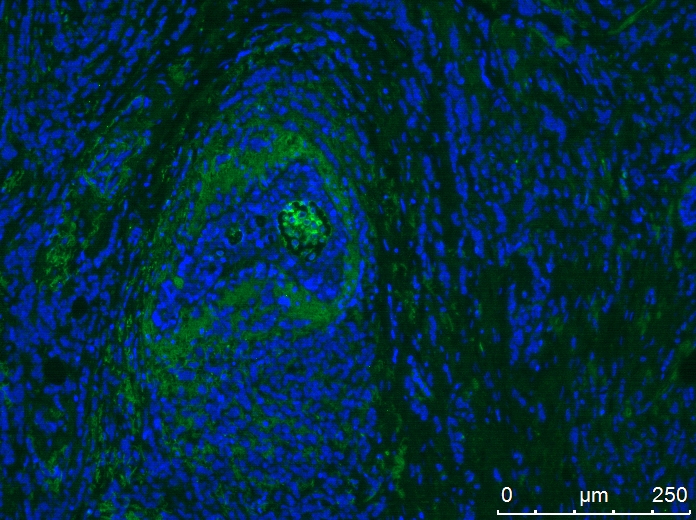

Supplement: Supplementary file 3 — Source data Fig. 2 [file 44318_2025_371_MOESM3_ESM.zip › SourceData_Figure 2/2M/Experiment_Pre-2-2.jpg]

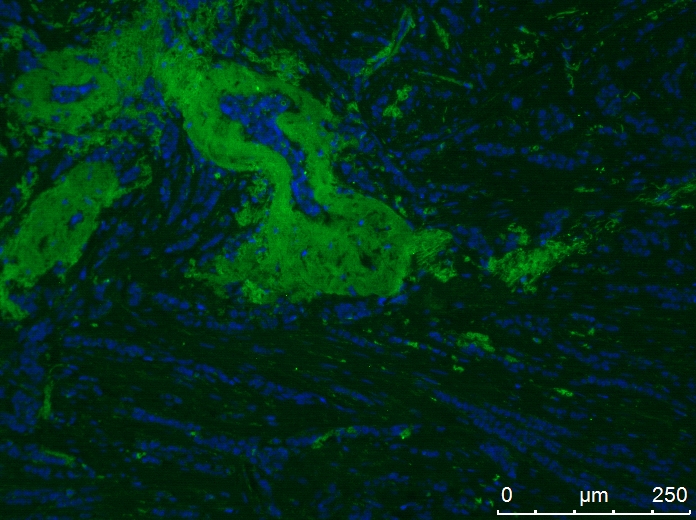

Supplement: Supplementary file 3 — Source data Fig. 2 [file 44318_2025_371_MOESM3_ESM.zip › SourceData_Figure 2/2M/Experiment_Pre-2-1.jpg]

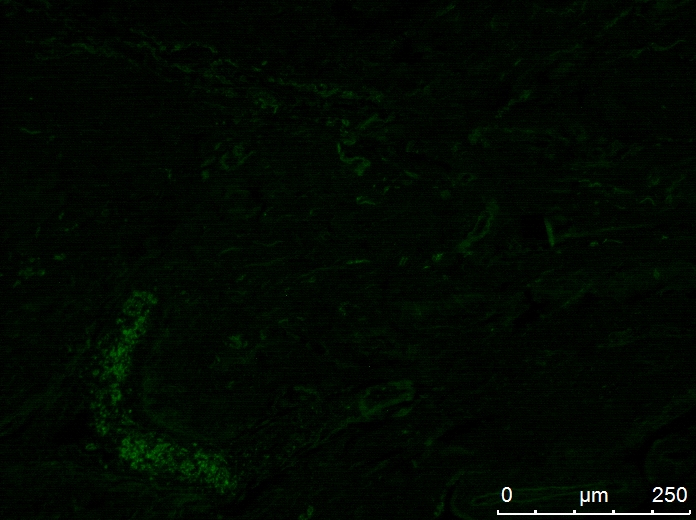

Supplement: Supplementary file 3 — Source data Fig. 2 [file 44318_2025_371_MOESM3_ESM.zip › SourceData_Figure 2/2M/Experiment_Post-1-1 LAMP1.jpg]

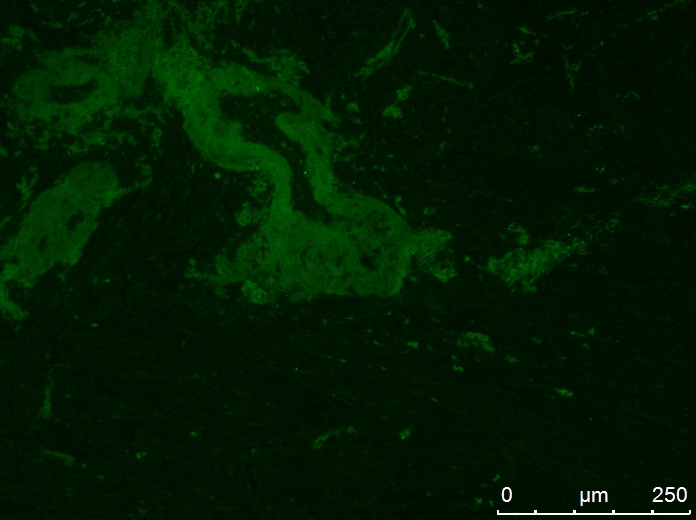

Supplement: Supplementary file 3 — Source data Fig. 2 [file 44318_2025_371_MOESM3_ESM.zip › SourceData_Figure 2/2M/Experiment_Pre-2-1 LAMP1.jpg]

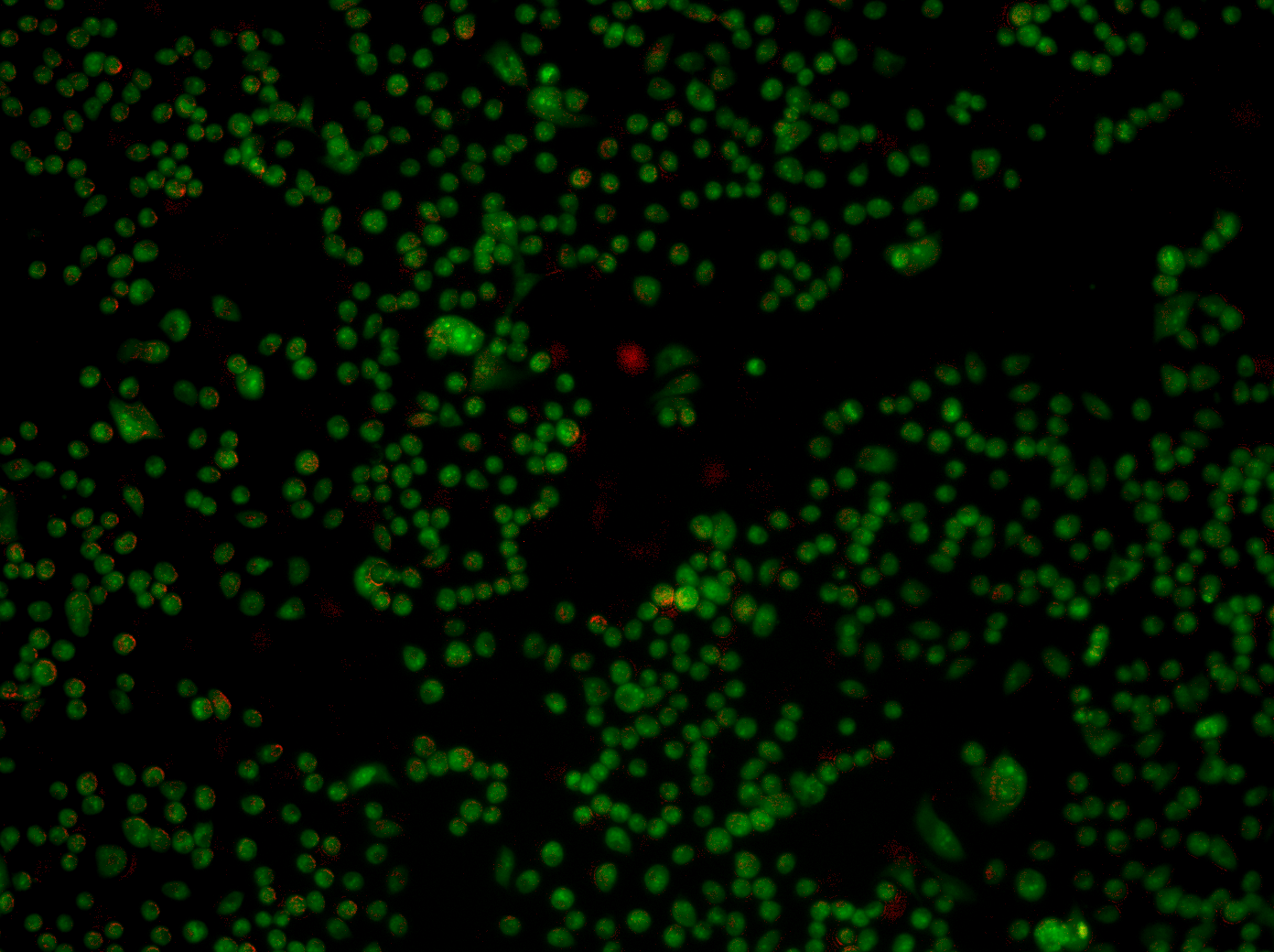

Supplement: Supplementary file 3 — Source data Fig. 2 [file 44318_2025_371_MOESM3_ESM.zip › SourceData_Figure 2/2K/T47D VEH.tif]

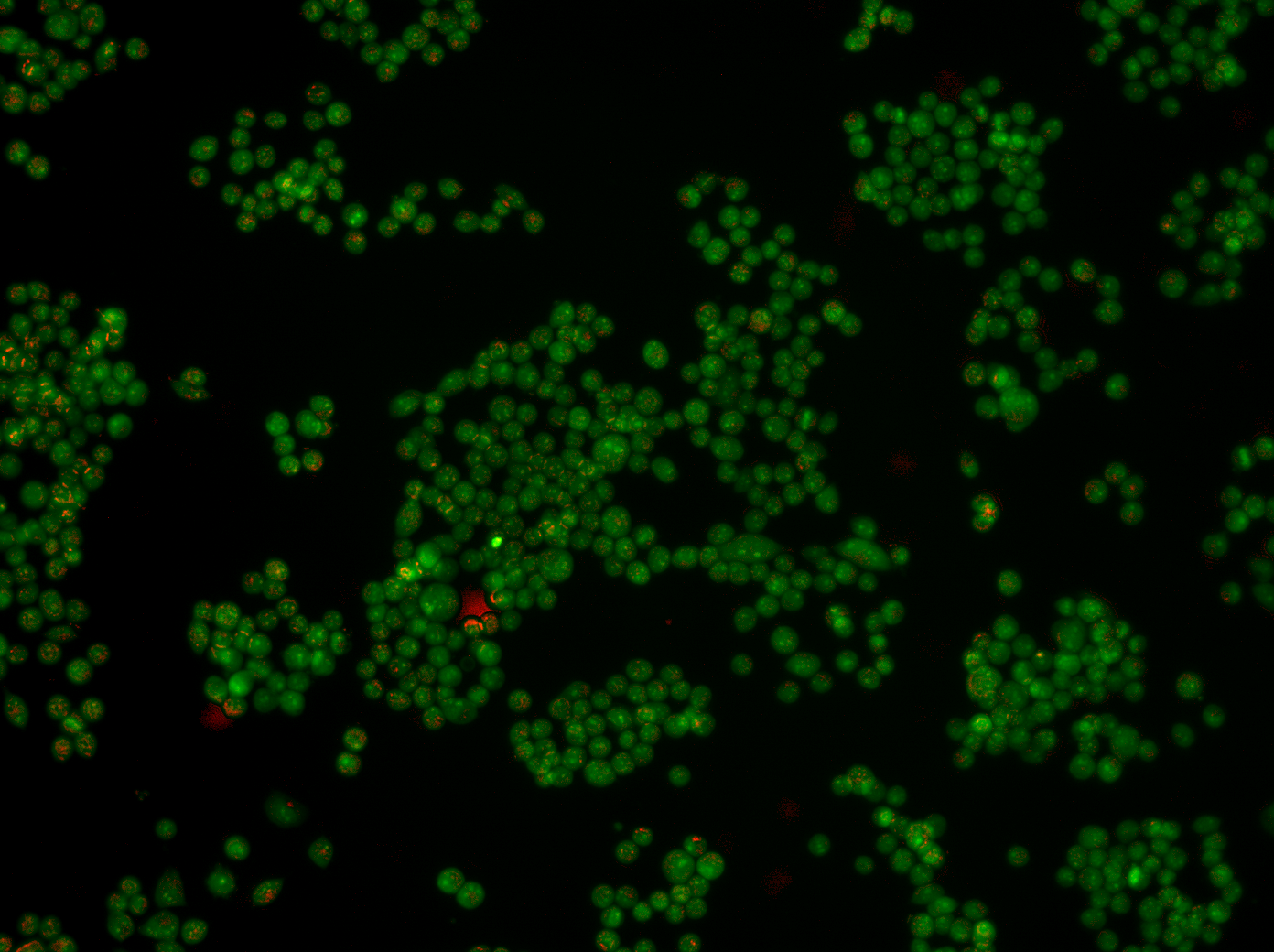

Supplement: Supplementary file 3 — Source data Fig. 2 [file 44318_2025_371_MOESM3_ESM.zip › SourceData_Figure 2/2K/BT474 VEH.tif]

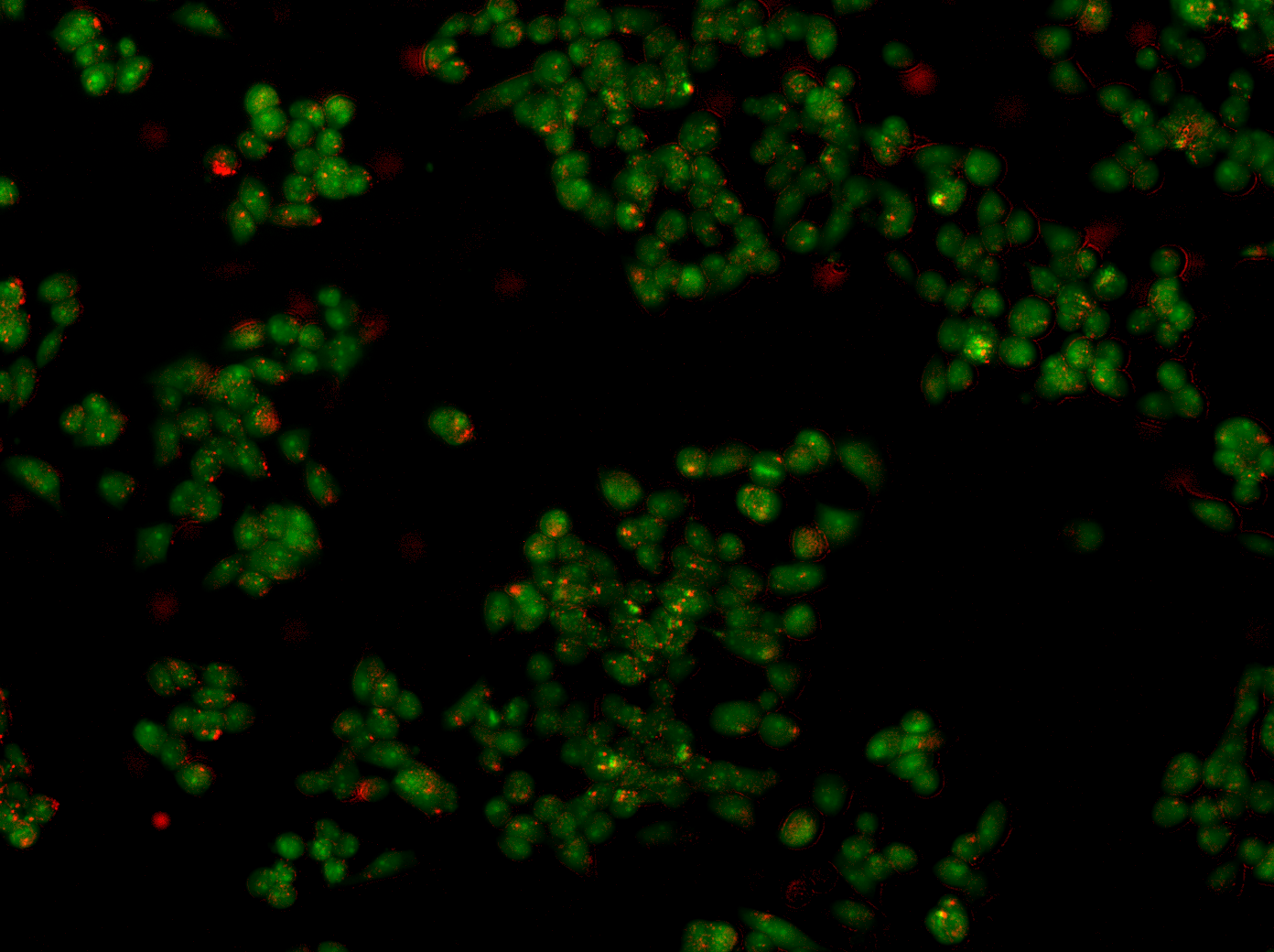

Supplement: Supplementary file 3 — Source data Fig. 2 [file 44318_2025_371_MOESM3_ESM.zip › SourceData_Figure 2/2K/BT474 ABEMA.tif]

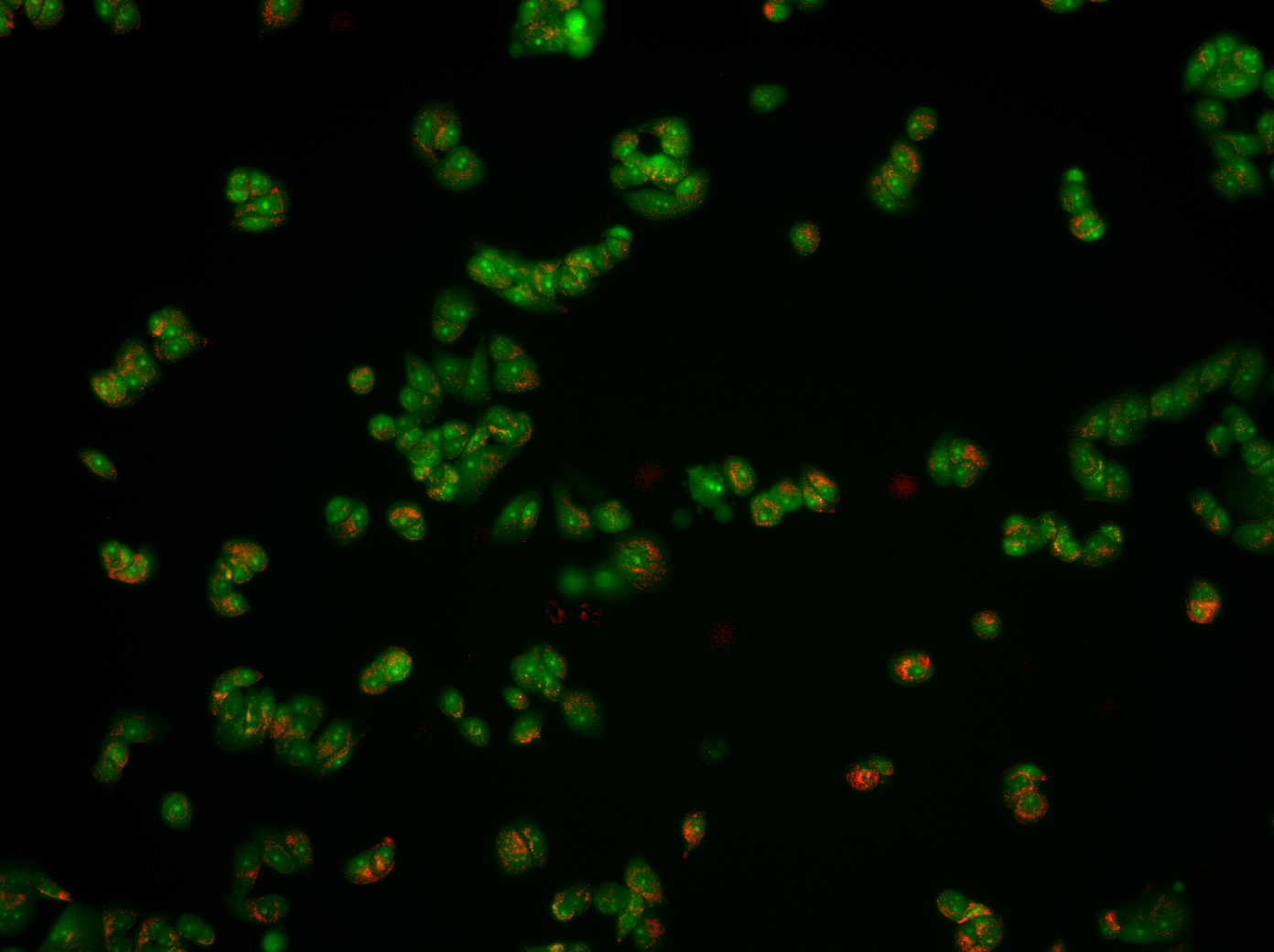

Supplement: Supplementary file 3 — Source data Fig. 2 [file 44318_2025_371_MOESM3_ESM.zip › SourceData_Figure 2/2K/T47D ABEMA.tif]

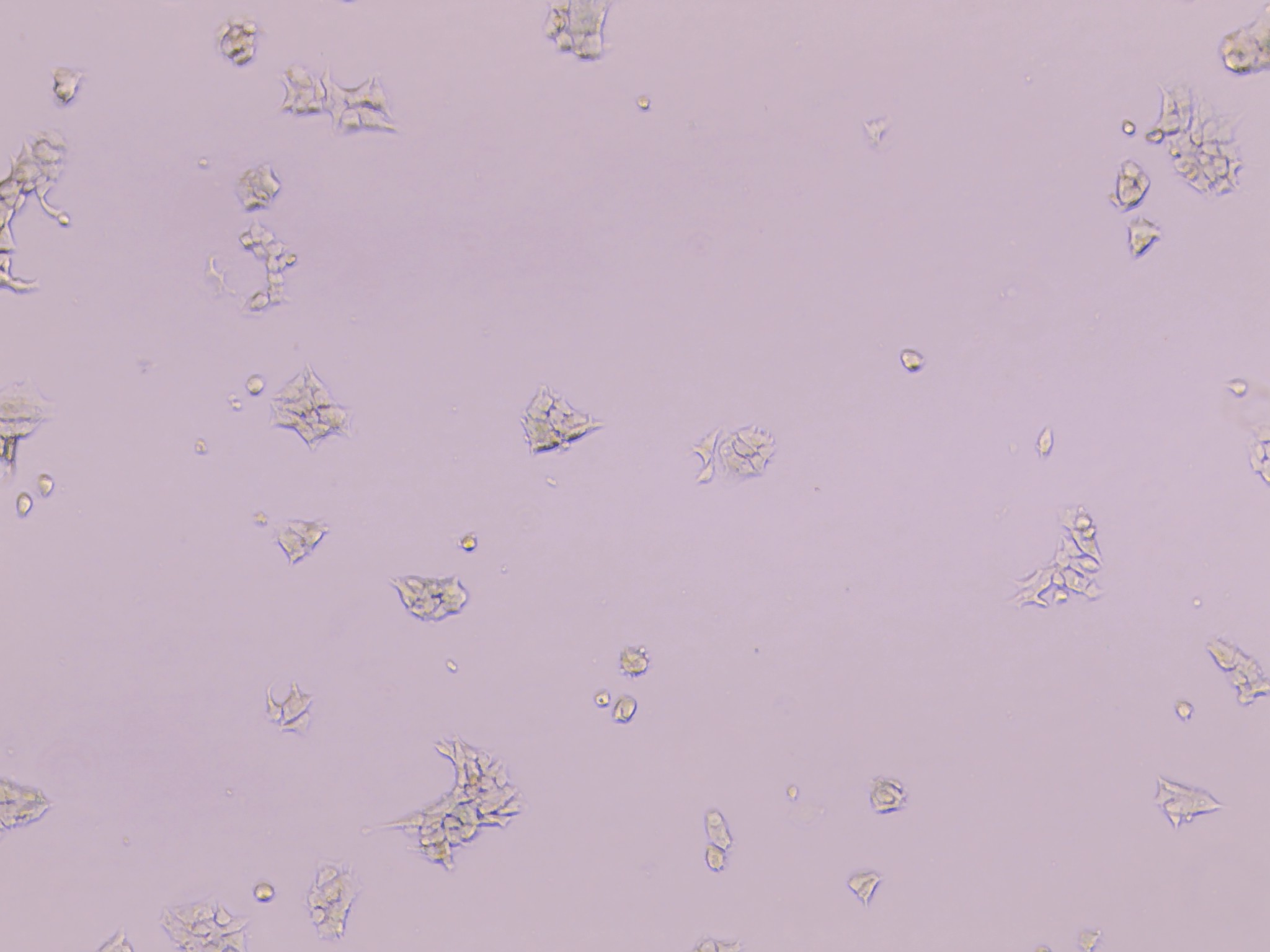

Supplement: Supplementary file 3 — Source data Fig. 2 [file 44318_2025_371_MOESM3_ESM.zip › SourceData_Figure 2/2J/bt747/bt747 abema 10215.jpg]

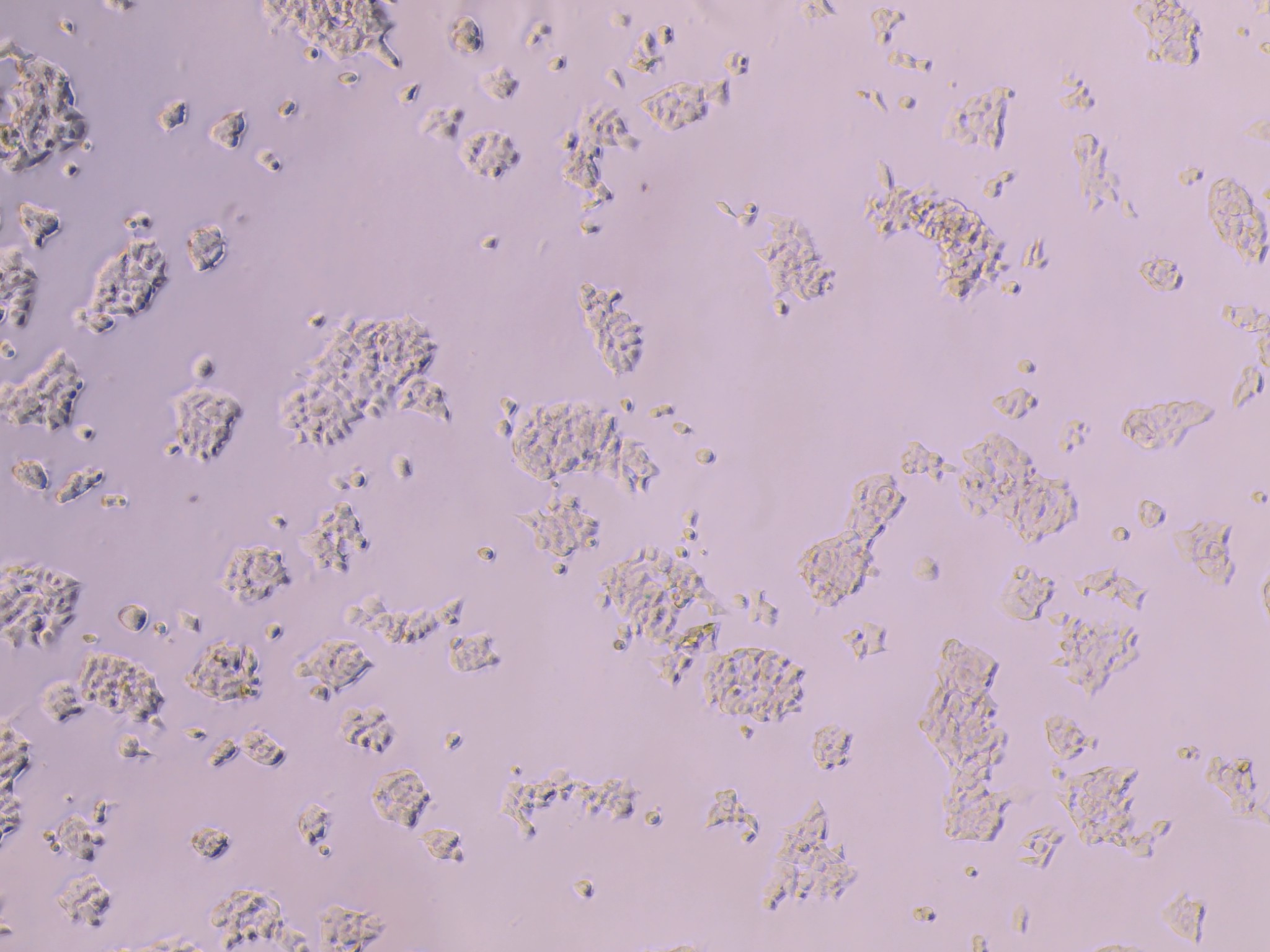

Supplement: Supplementary file 3 — Source data Fig. 2 [file 44318_2025_371_MOESM3_ESM.zip › SourceData_Figure 2/2J/bt747/bt747 ctrl 10216.jpg]

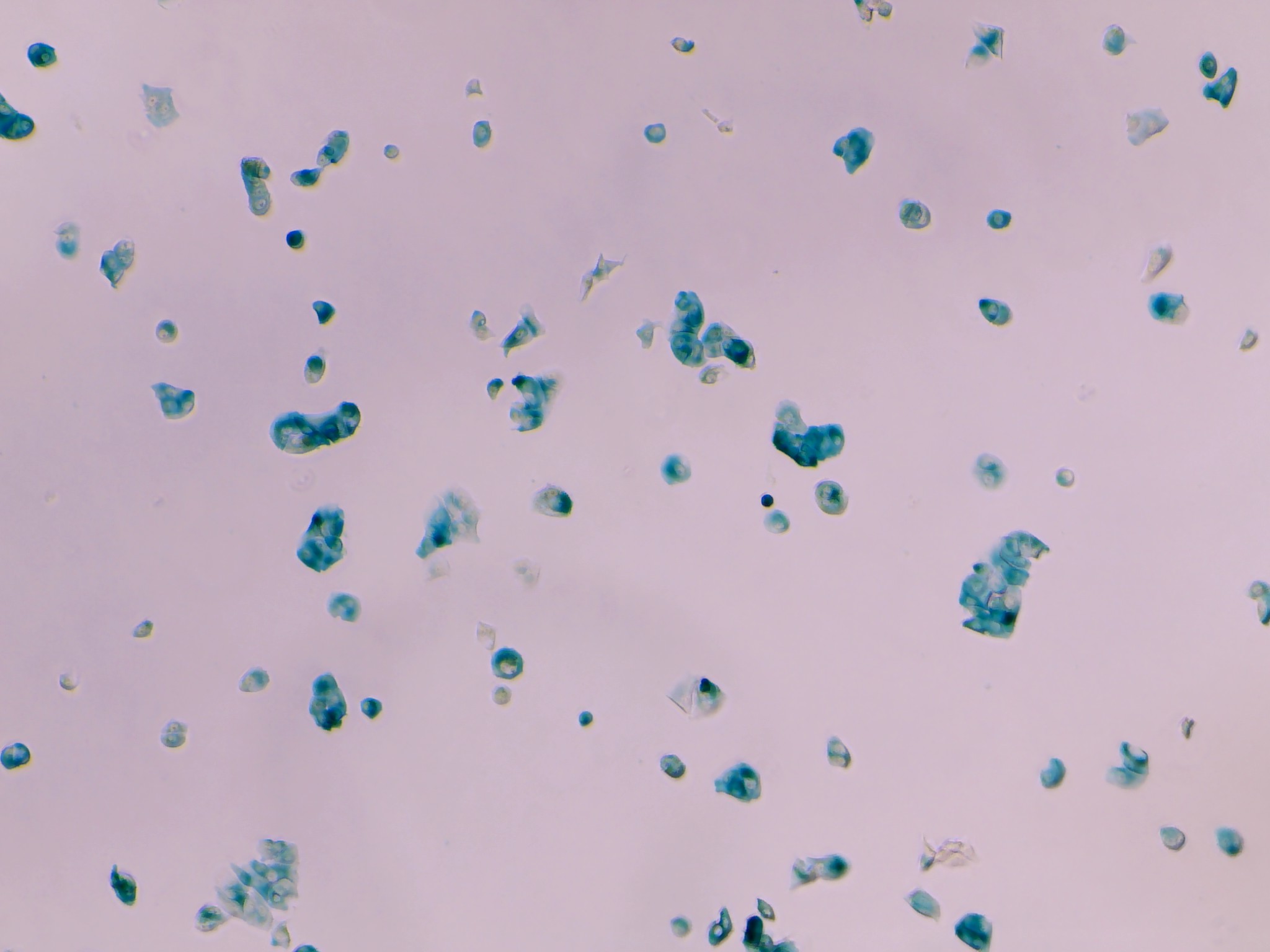

Supplement: Supplementary file 3 — Source data Fig. 2 [file 44318_2025_371_MOESM3_ESM.zip › SourceData_Figure 2/2J/t47d/n=1/t47d abema 30211.jpg]

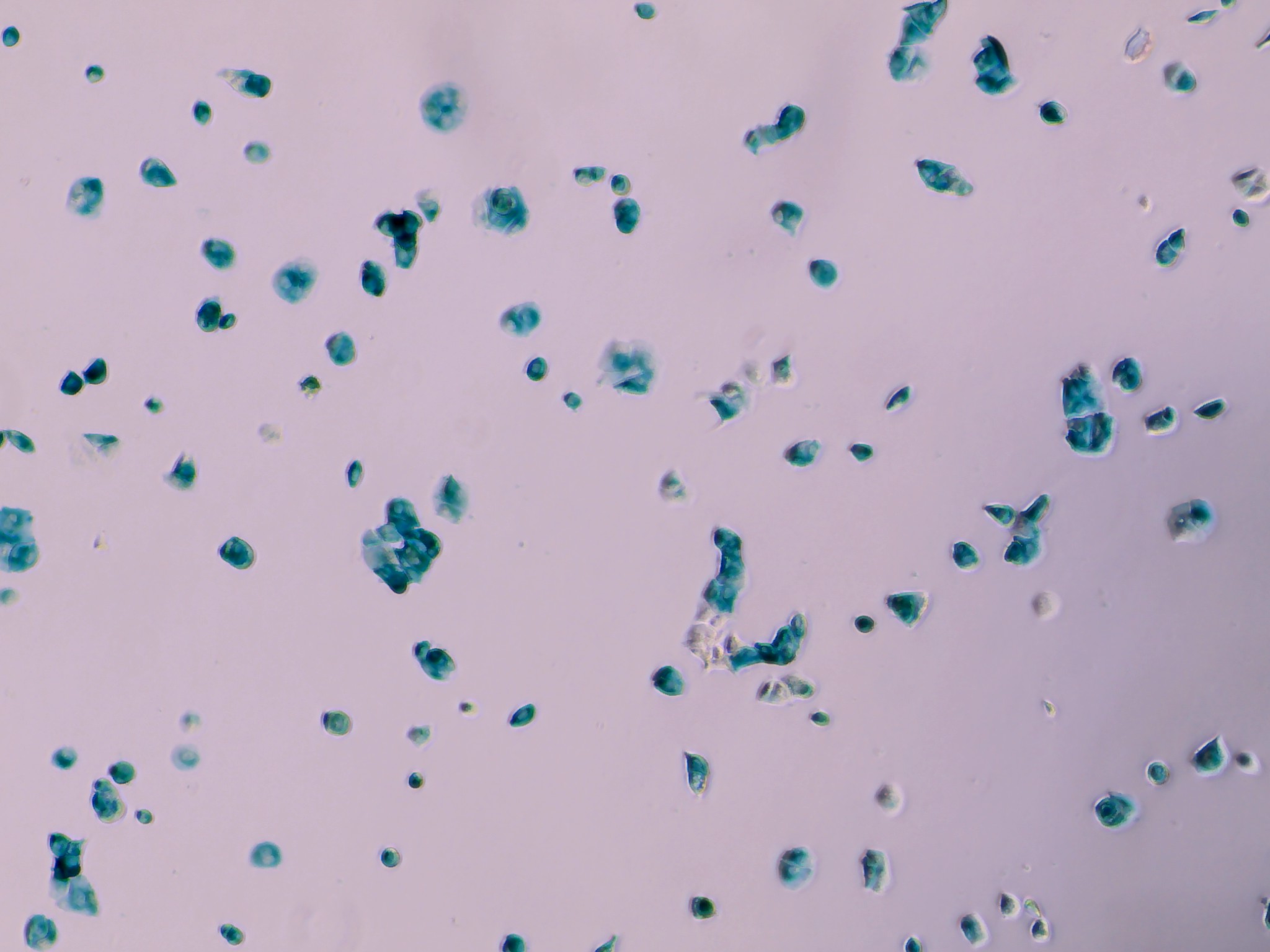

Supplement: Supplementary file 3 — Source data Fig. 2 [file 44318_2025_371_MOESM3_ESM.zip › SourceData_Figure 2/2J/t47d/n=1/t47d abema 20210.jpg]

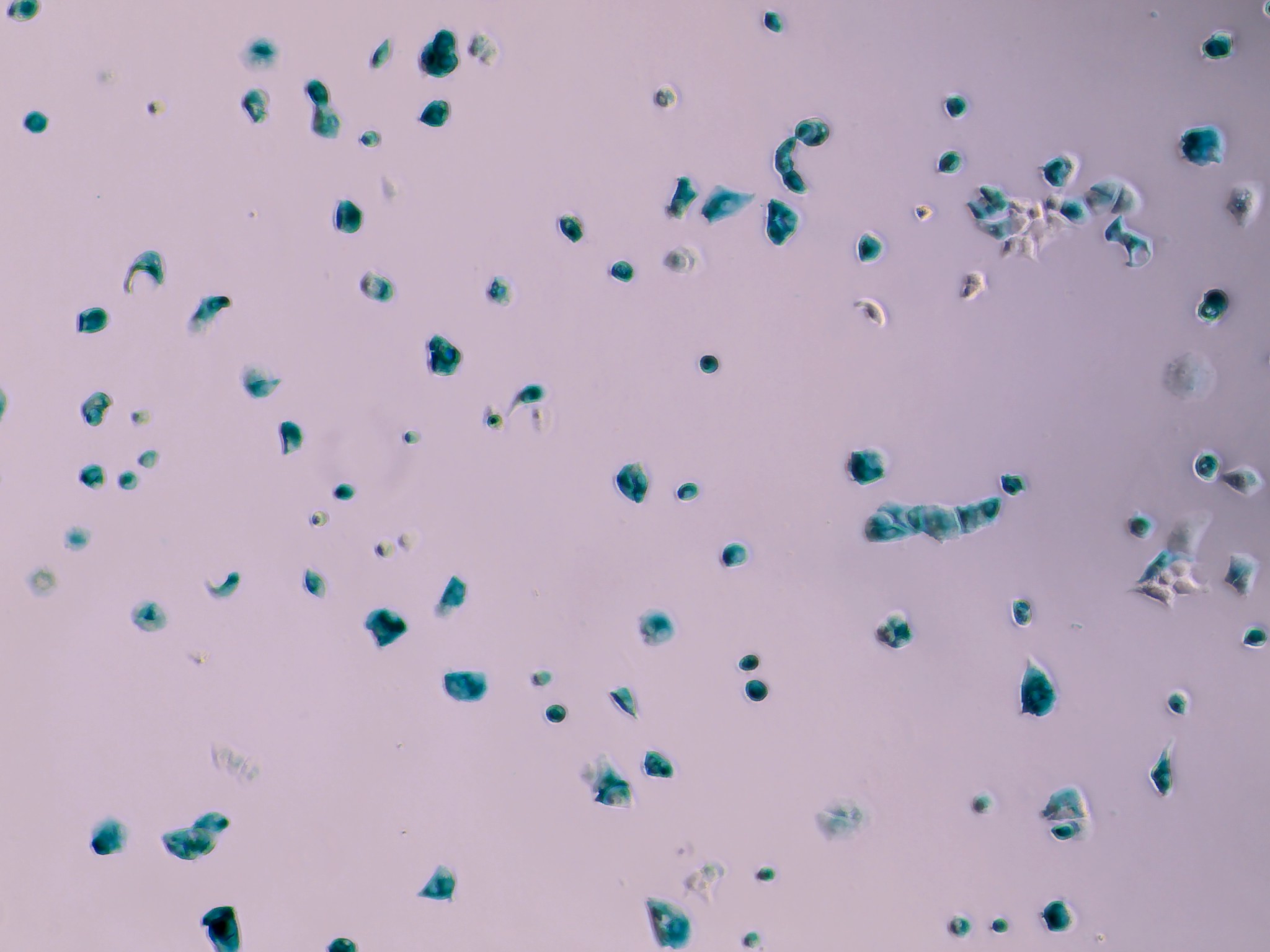

Supplement: Supplementary file 3 — Source data Fig. 2 [file 44318_2025_371_MOESM3_ESM.zip › SourceData_Figure 2/2J/t47d/n=1/t47d abema 60214.jpg]

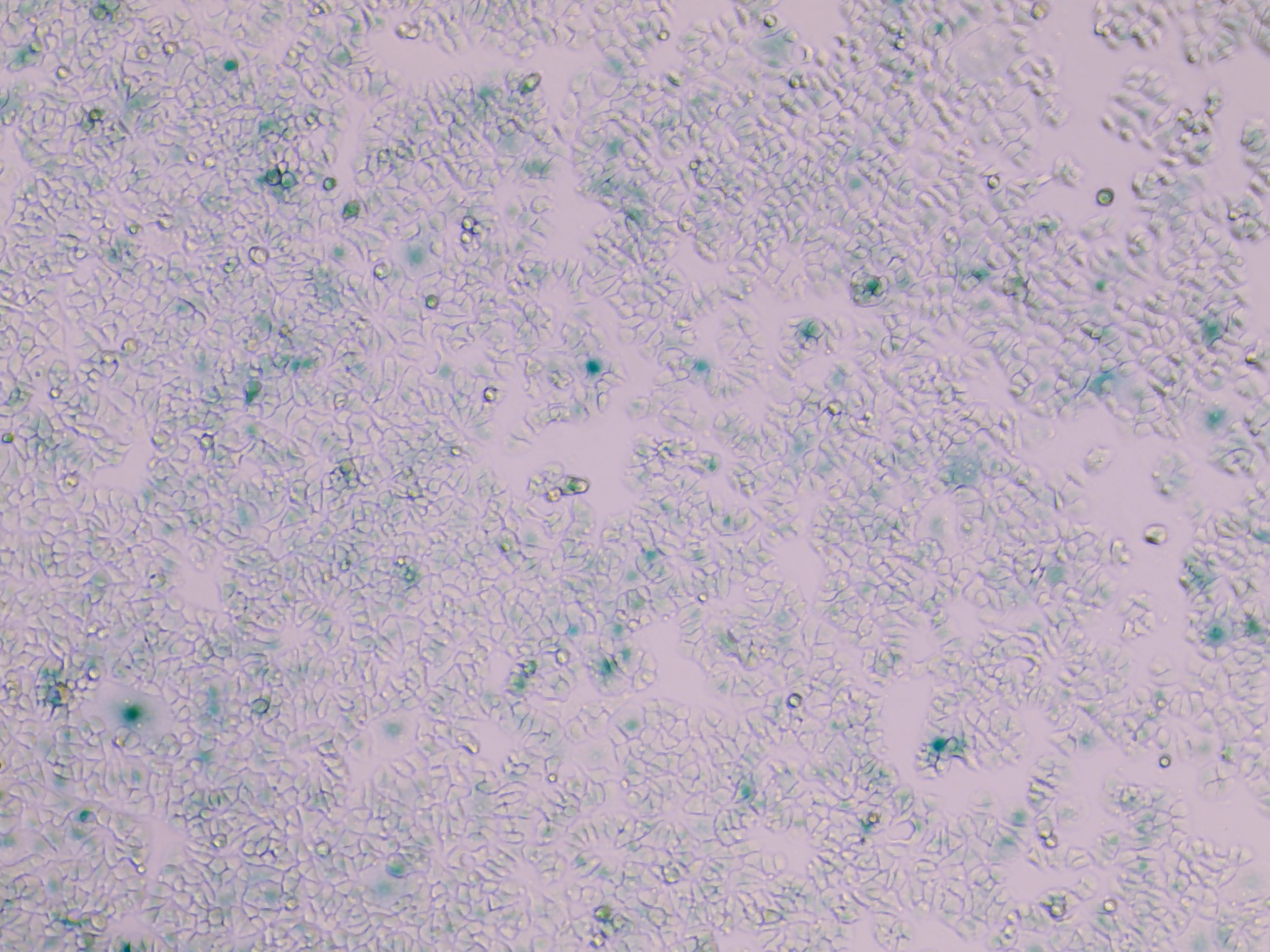

Supplement: Supplementary file 3 — Source data Fig. 2 [file 44318_2025_371_MOESM3_ESM.zip › SourceData_Figure 2/2J/t47d/n=1/t47d ctrl 20206.jpg]

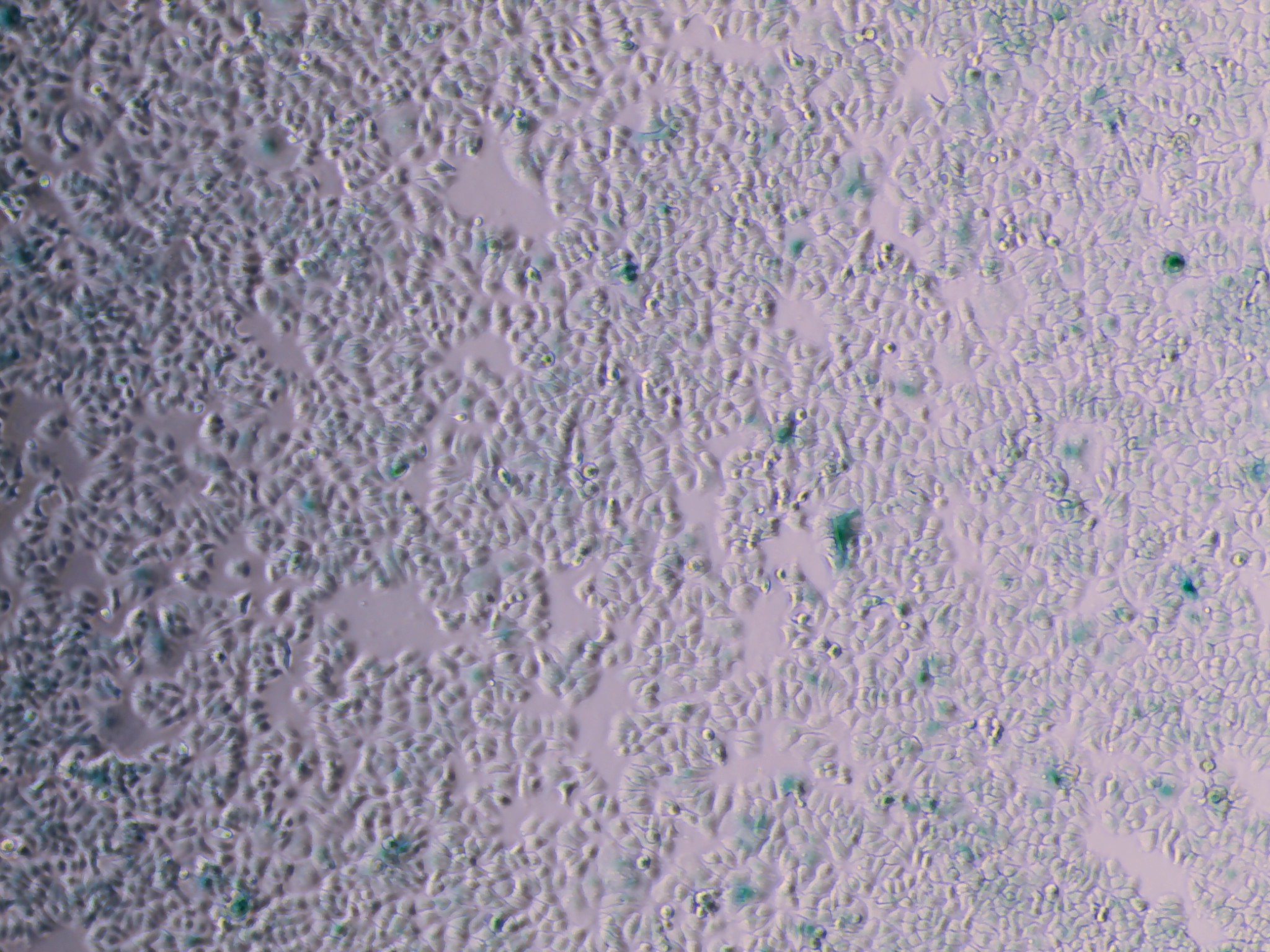

Supplement: Supplementary file 3 — Source data Fig. 2 [file 44318_2025_371_MOESM3_ESM.zip › SourceData_Figure 2/2J/t47d/n=1/t47d ctrl 30207.jpg]

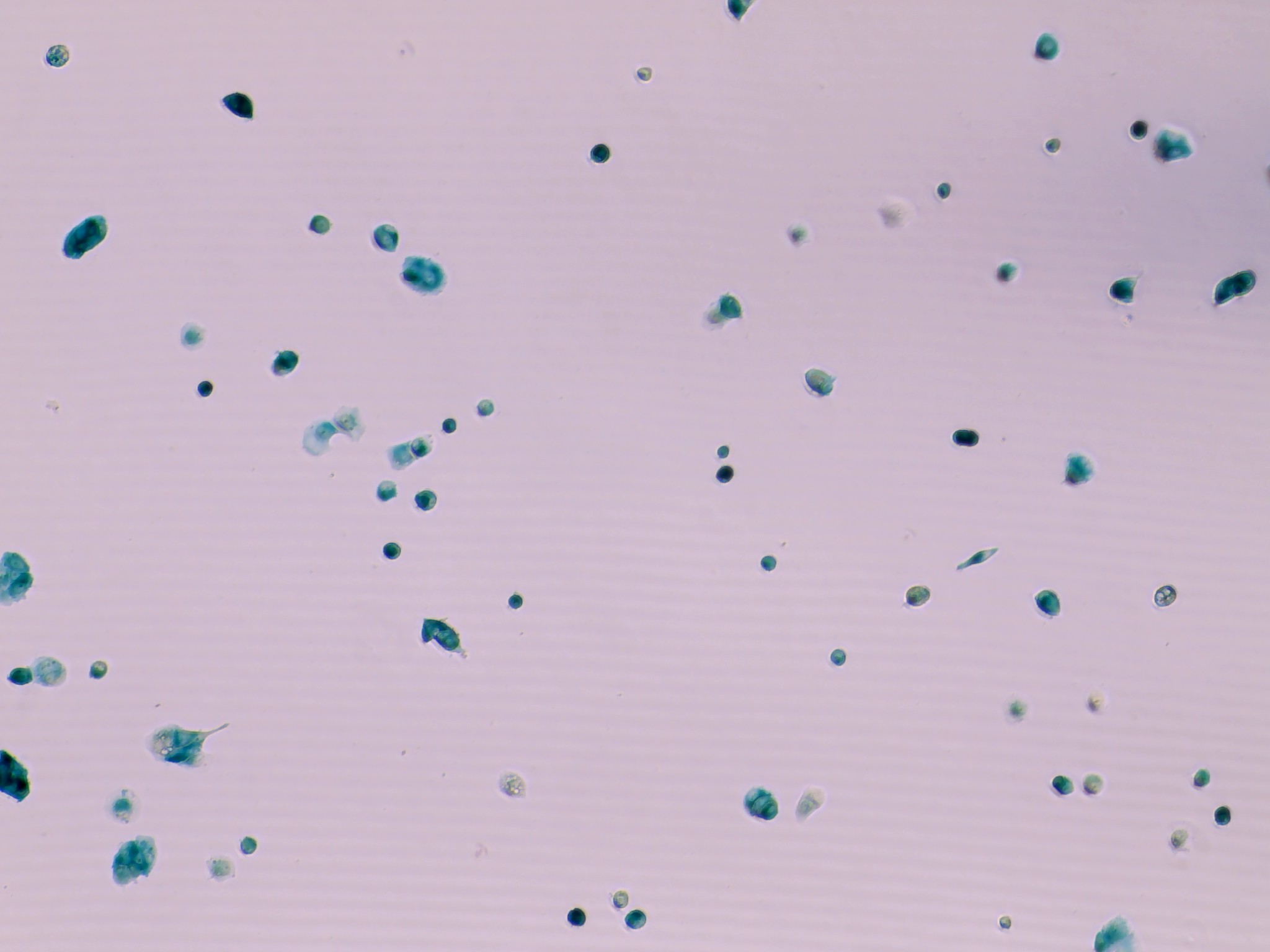

Supplement: Supplementary file 3 — Source data Fig. 2 [file 44318_2025_371_MOESM3_ESM.zip › SourceData_Figure 2/2J/t47d/n=1/t47d abema 40212.jpg]

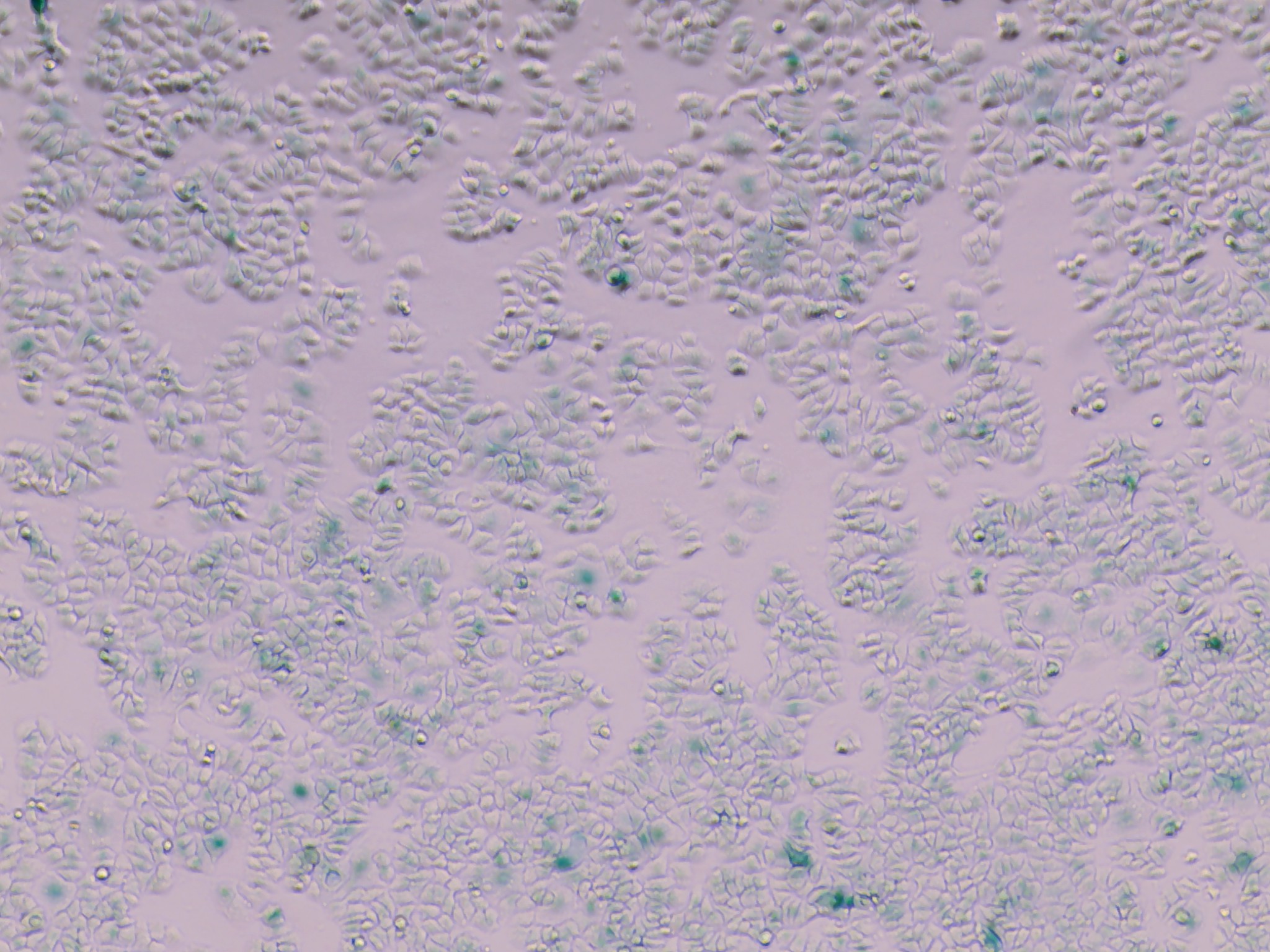

Supplement: Supplementary file 3 — Source data Fig. 2 [file 44318_2025_371_MOESM3_ESM.zip › SourceData_Figure 2/2J/t47d/n=1/t47d ctrl 10205.jpg]

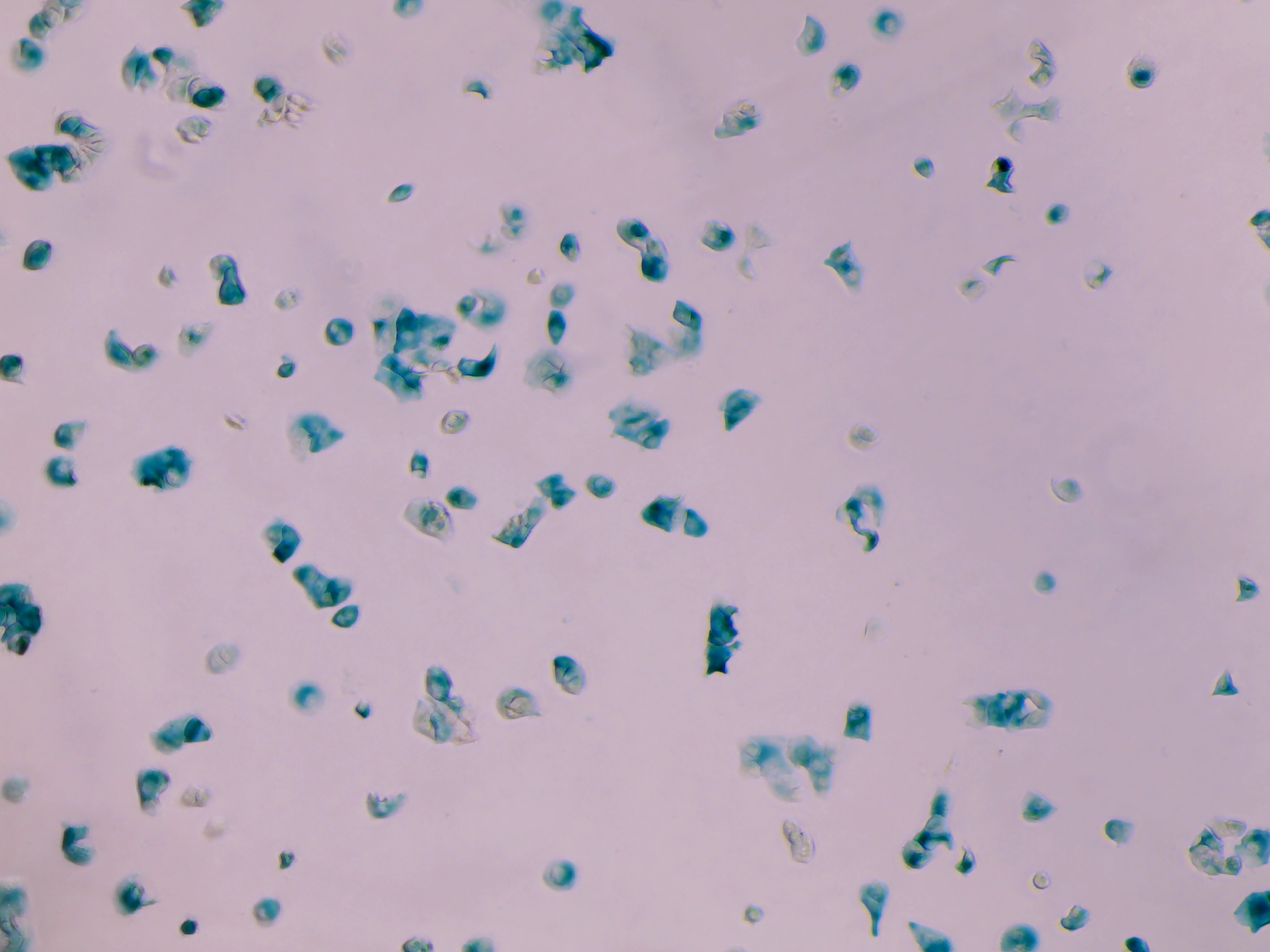

Supplement: Supplementary file 3 — Source data Fig. 2 [file 44318_2025_371_MOESM3_ESM.zip › SourceData_Figure 2/2J/t47d/n=1/t47d abema 10209.jpg]

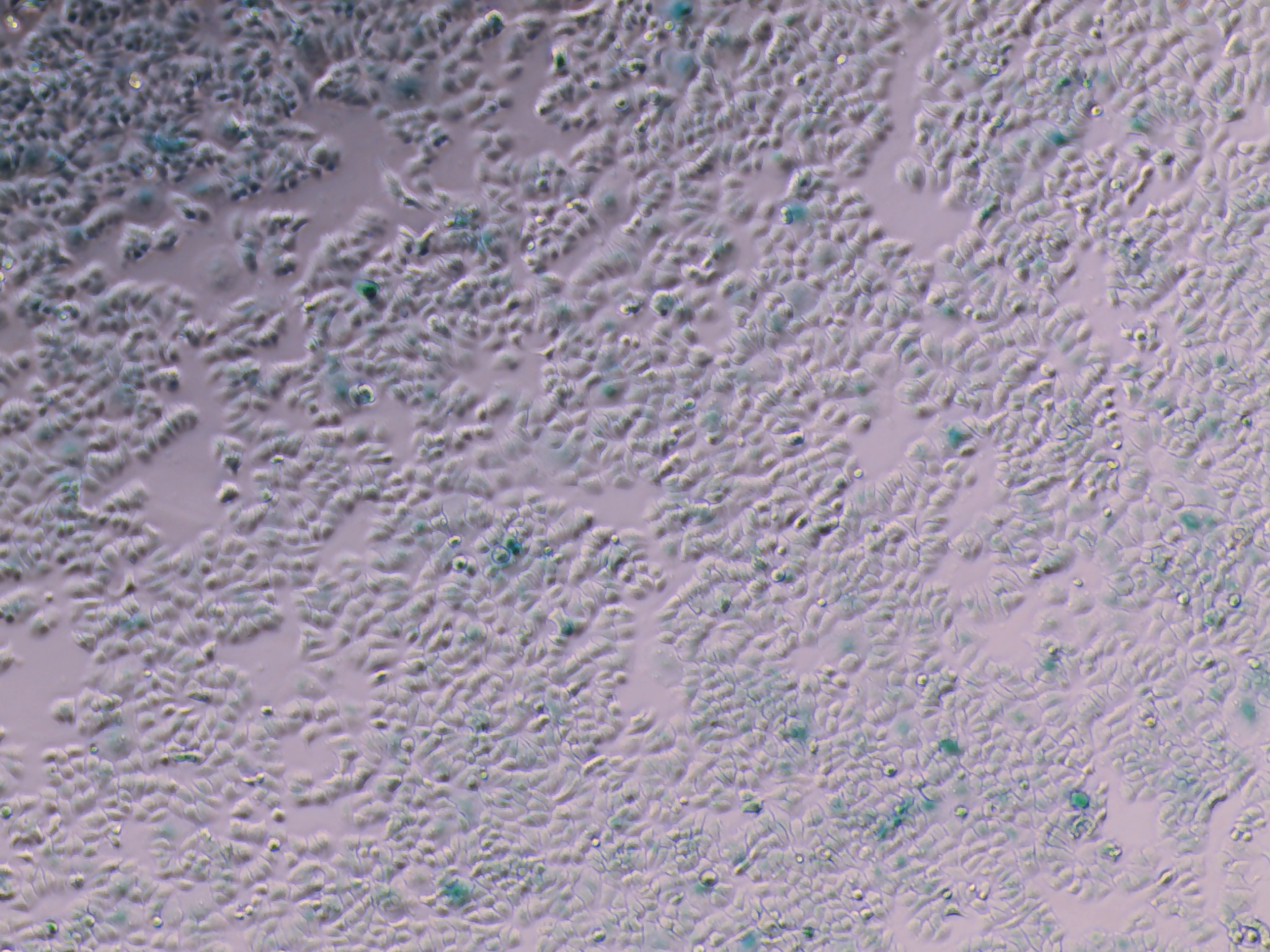

Supplement: Supplementary file 3 — Source data Fig. 2 [file 44318_2025_371_MOESM3_ESM.zip › SourceData_Figure 2/2J/t47d/n=1/t47d ctrl 40208.jpg]

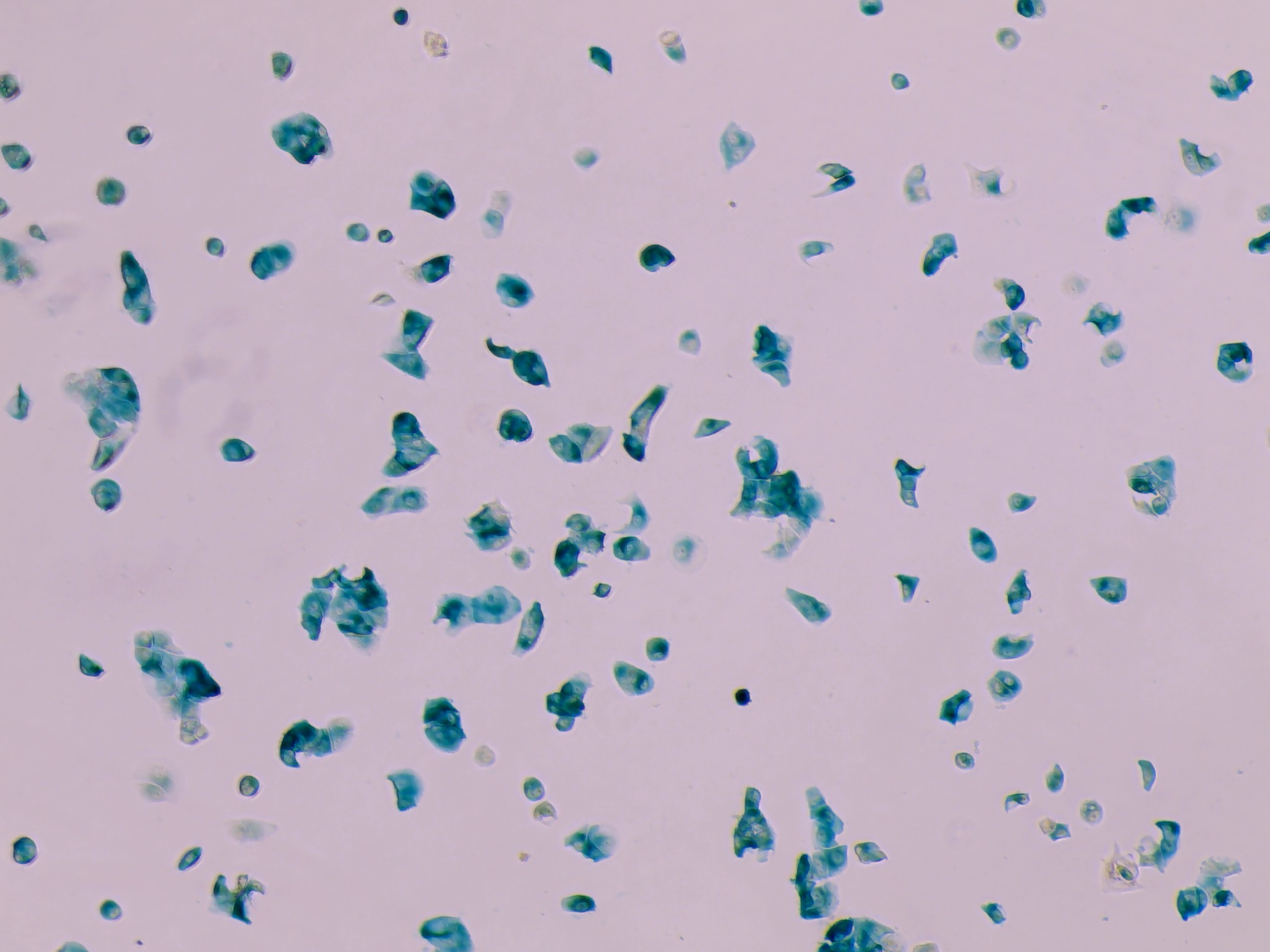

Supplement: Supplementary file 3 — Source data Fig. 2 [file 44318_2025_371_MOESM3_ESM.zip › SourceData_Figure 2/2J/t47d/n=1/t47d abema 50213.jpg]

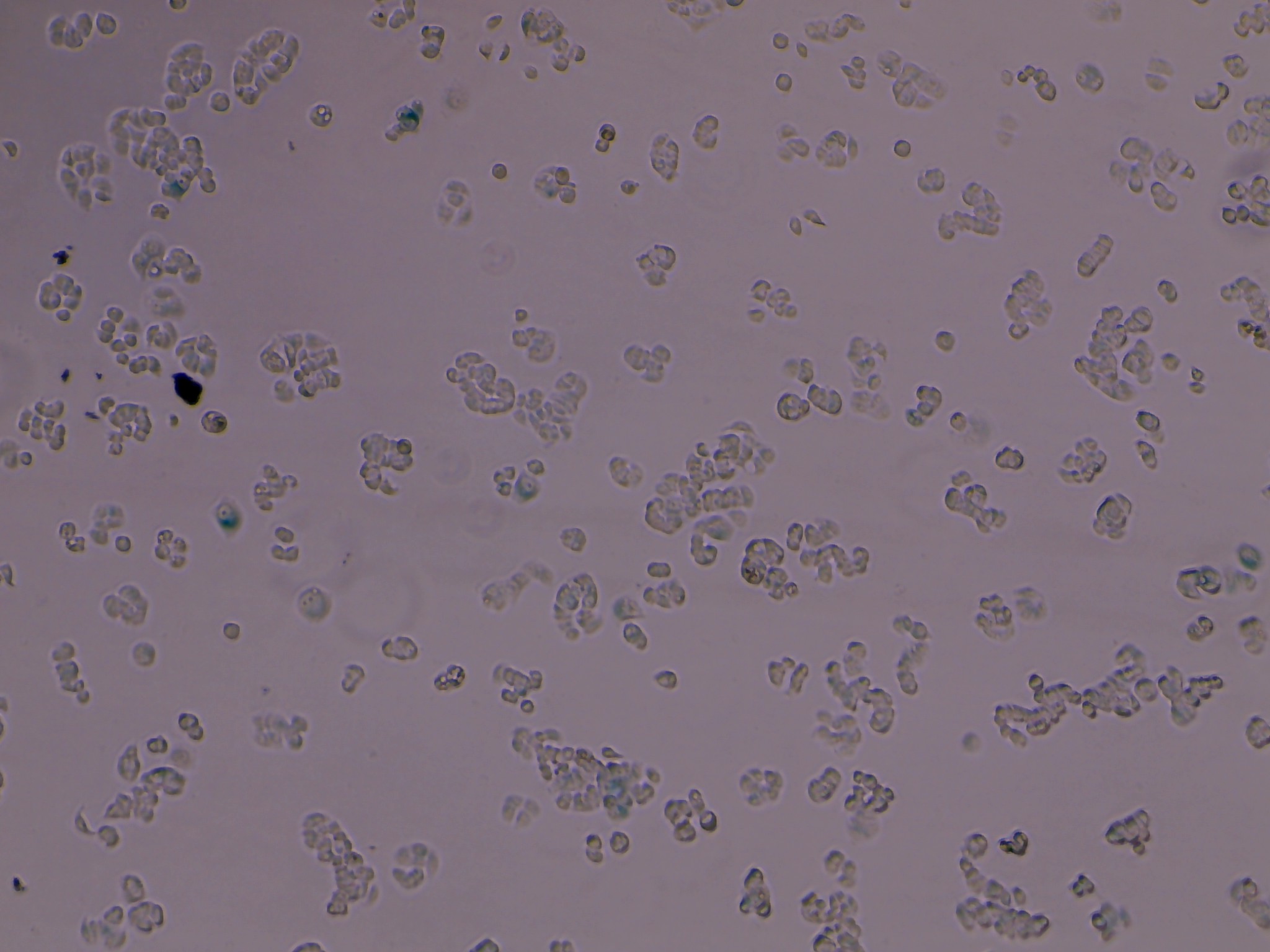

Supplement: Supplementary file 3 — Source data Fig. 2 [file 44318_2025_371_MOESM3_ESM.zip › SourceData_Figure 2/2J/t47d/n=3/t47d ctrl 50366.jpg]

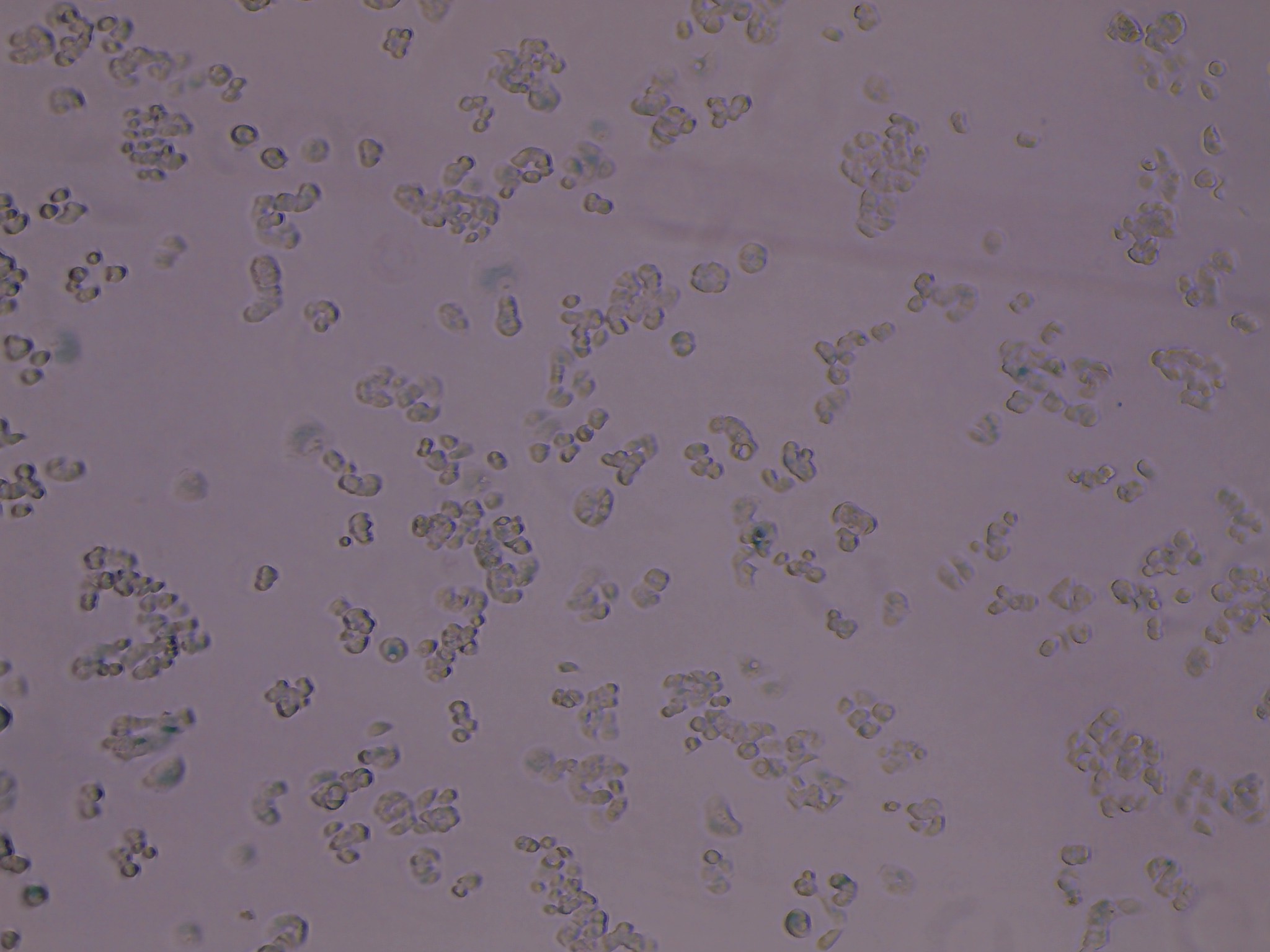

Supplement: Supplementary file 3 — Source data Fig. 2 [file 44318_2025_371_MOESM3_ESM.zip › SourceData_Figure 2/2J/t47d/n=3/t47d ctrl 40365.jpg]

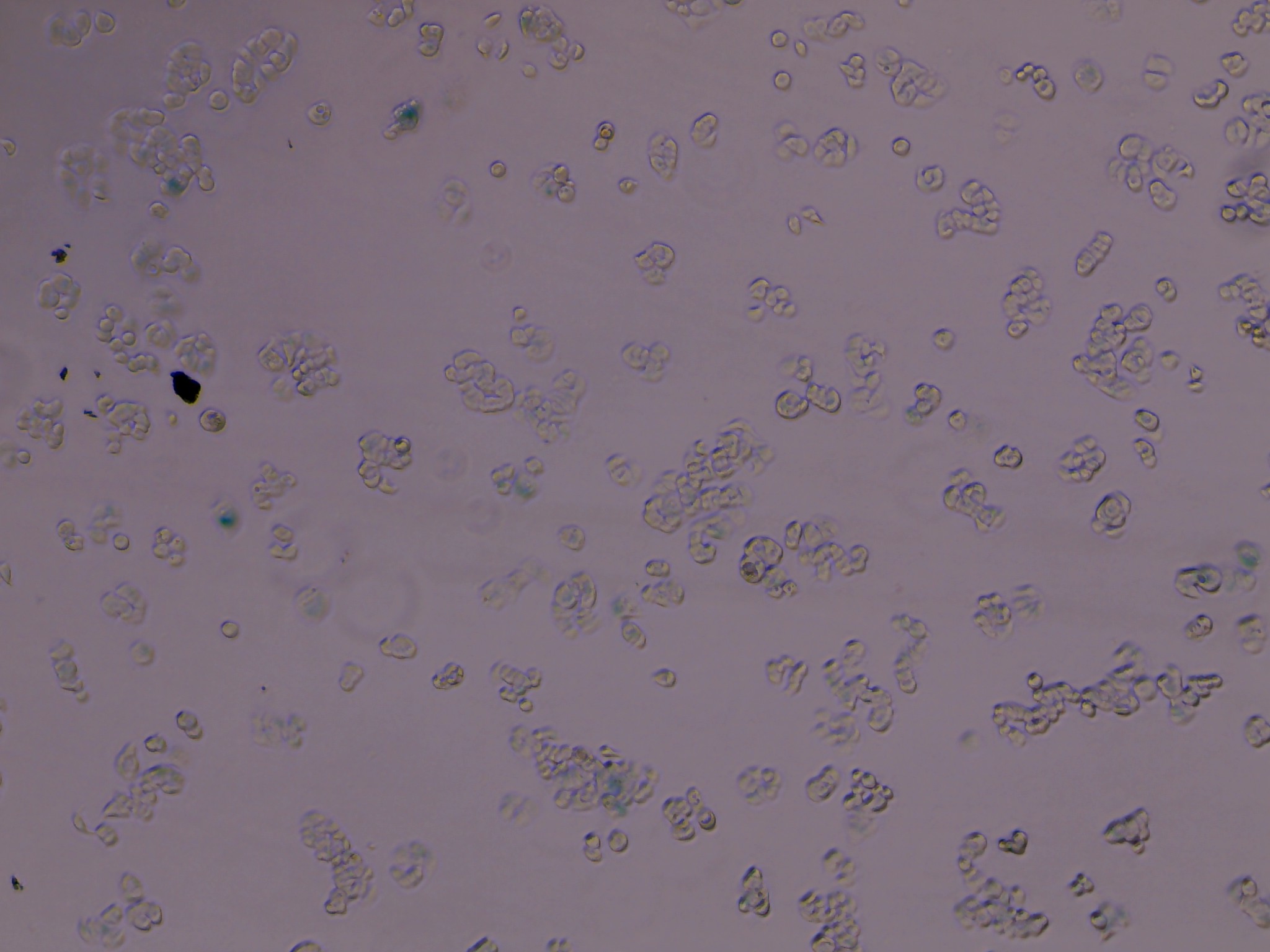

Supplement: Supplementary file 3 — Source data Fig. 2 [file 44318_2025_371_MOESM3_ESM.zip › SourceData_Figure 2/2J/t47d/n=3/t47d ctrl 60367.jpg]

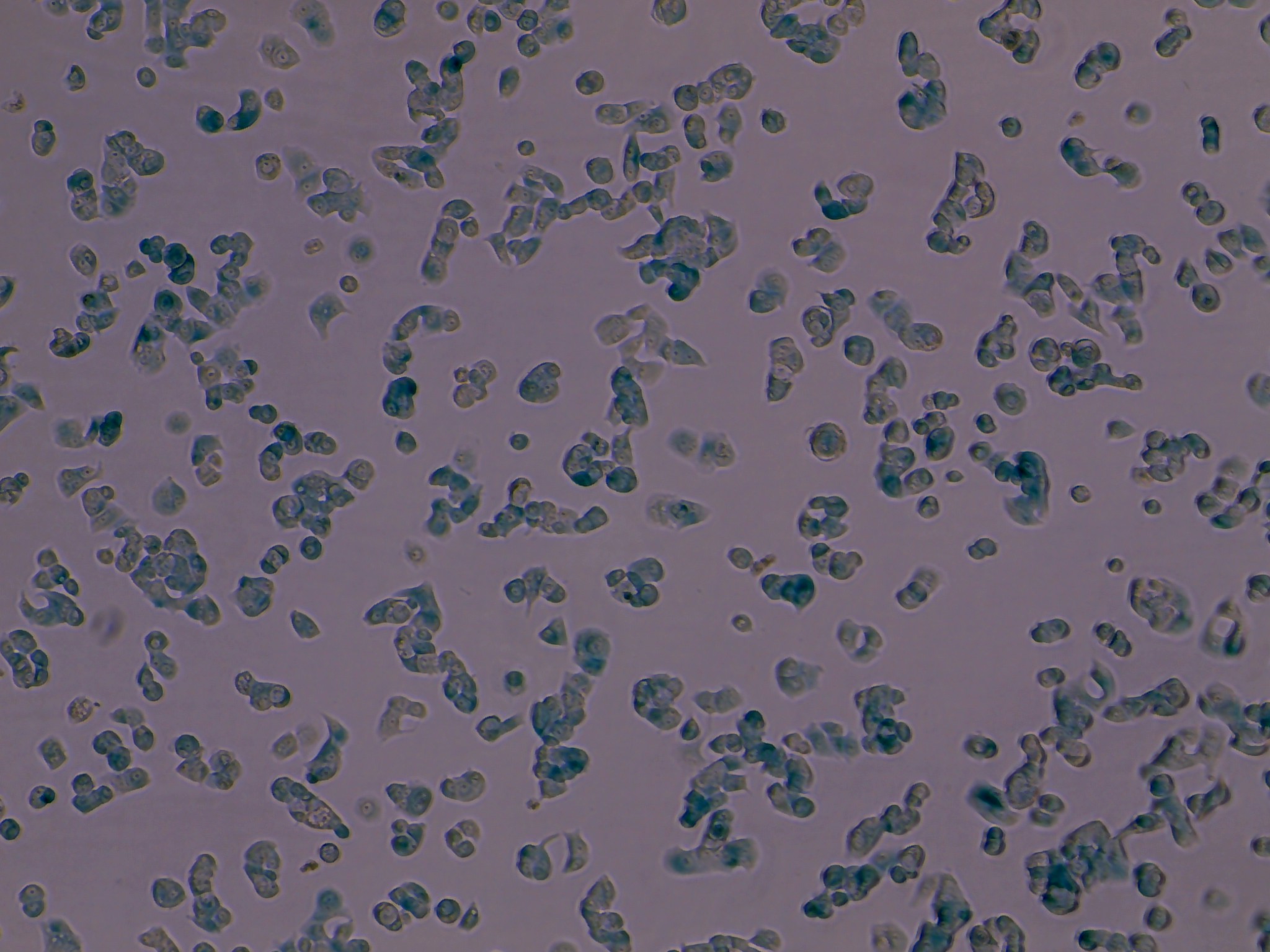

Supplement: Supplementary file 3 — Source data Fig. 2 [file 44318_2025_371_MOESM3_ESM.zip › SourceData_Figure 2/2J/t47d/n=3/t47d abema 60374.jpg]

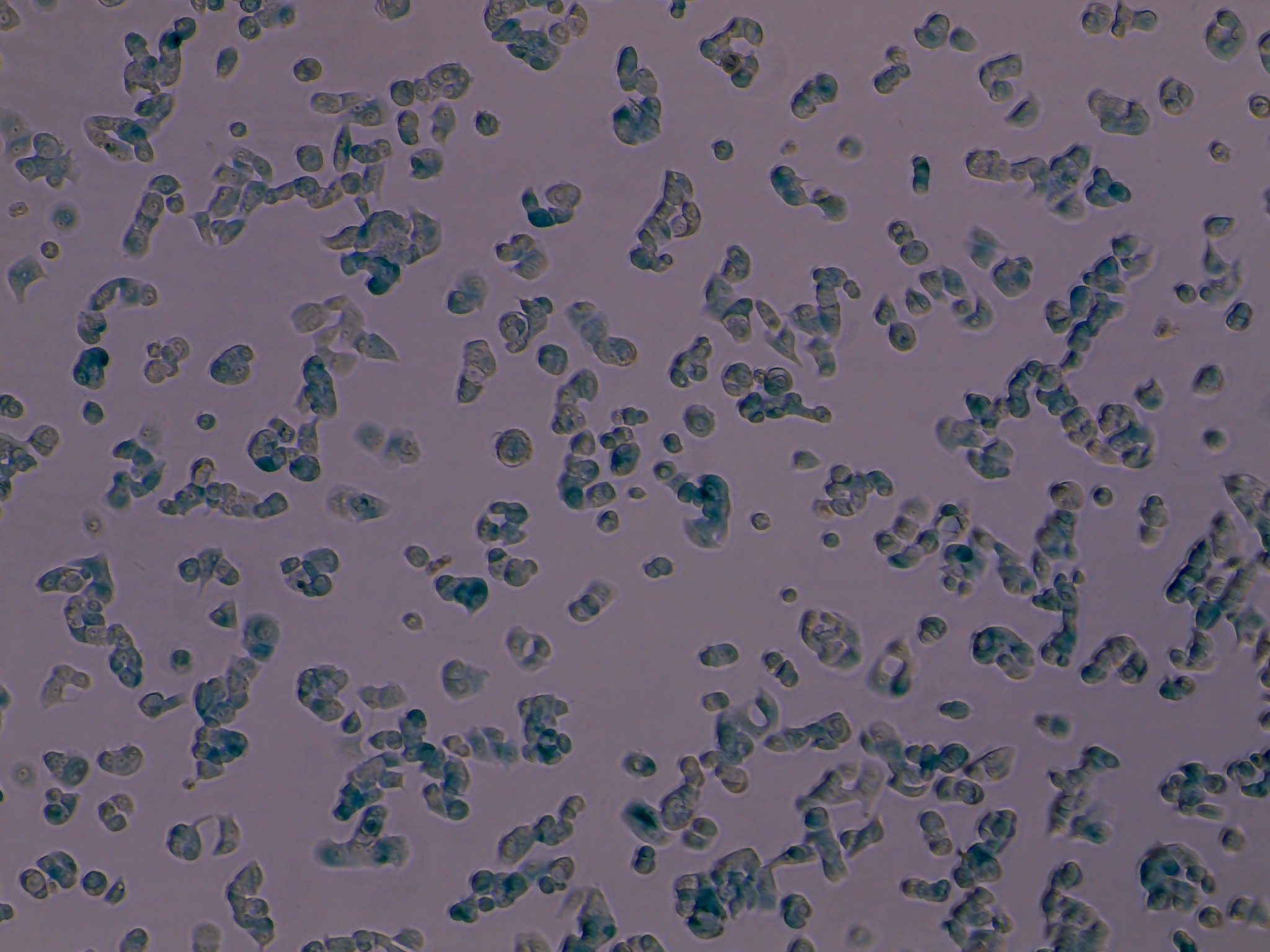

Supplement: Supplementary file 3 — Source data Fig. 2 [file 44318_2025_371_MOESM3_ESM.zip › SourceData_Figure 2/2J/t47d/n=3/t47d abema 40371.jpg]

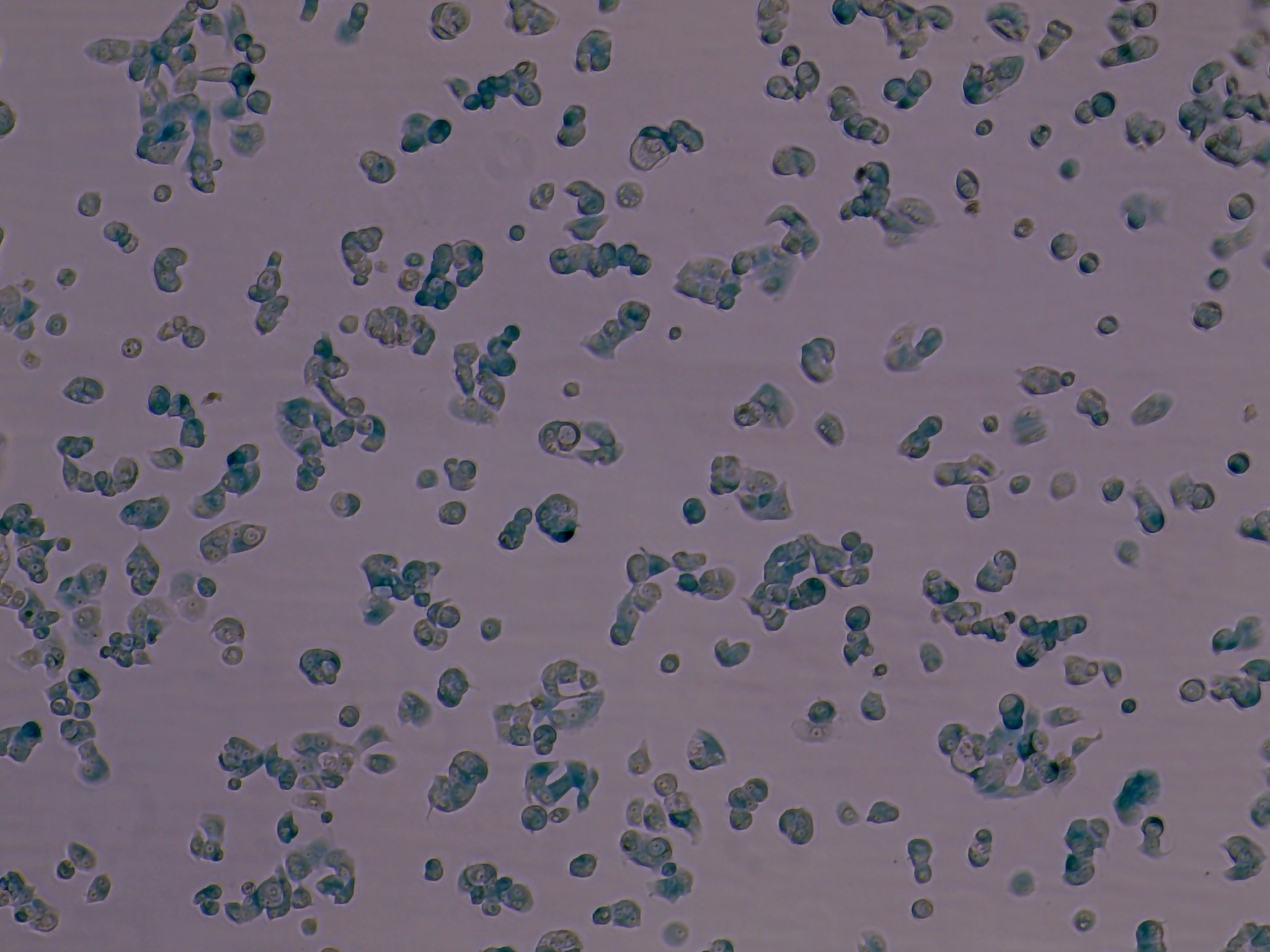

Supplement: Supplementary file 3 — Source data Fig. 2 [file 44318_2025_371_MOESM3_ESM.zip › SourceData_Figure 2/2J/t47d/n=3/t47d abema 50373.jpg]

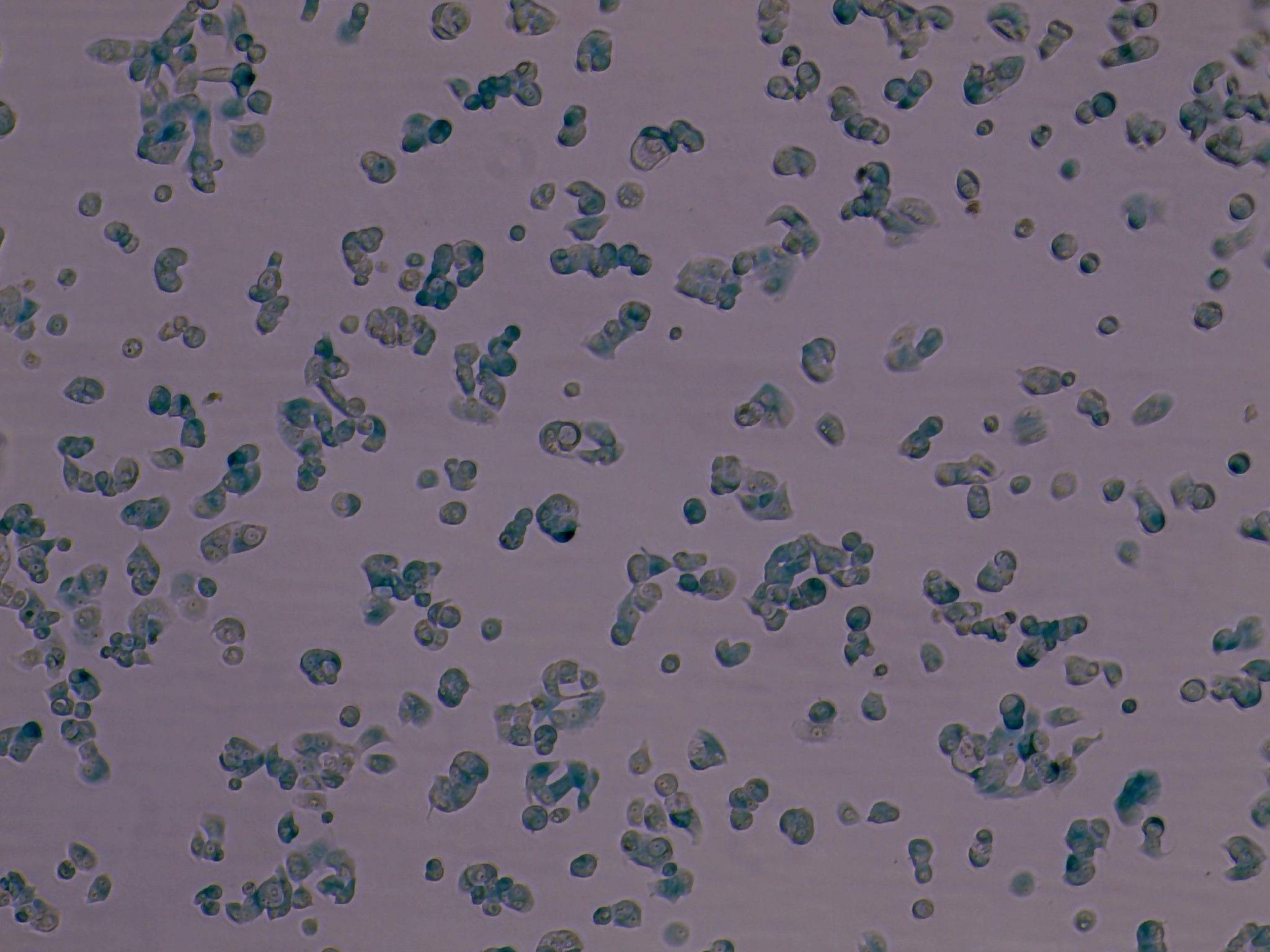

Supplement: Supplementary file 3 — Source data Fig. 2 [file 44318_2025_371_MOESM3_ESM.zip › SourceData_Figure 2/2J/t47d/n=3/t47d abema 50372.jpg]

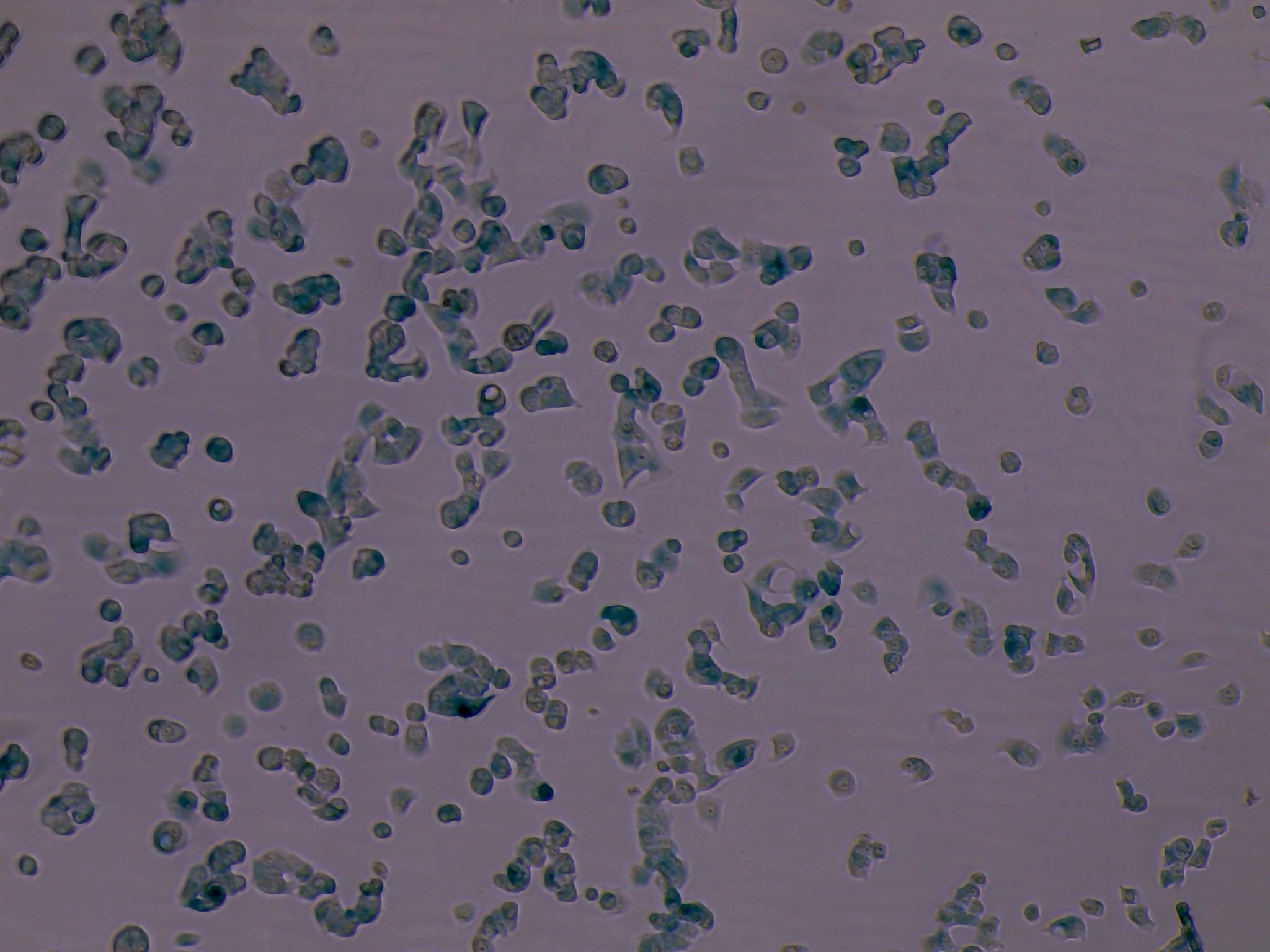

Supplement: Supplementary file 3 — Source data Fig. 2 [file 44318_2025_371_MOESM3_ESM.zip › SourceData_Figure 2/2J/t47d/n=2/t47d abema 30370.jpg]

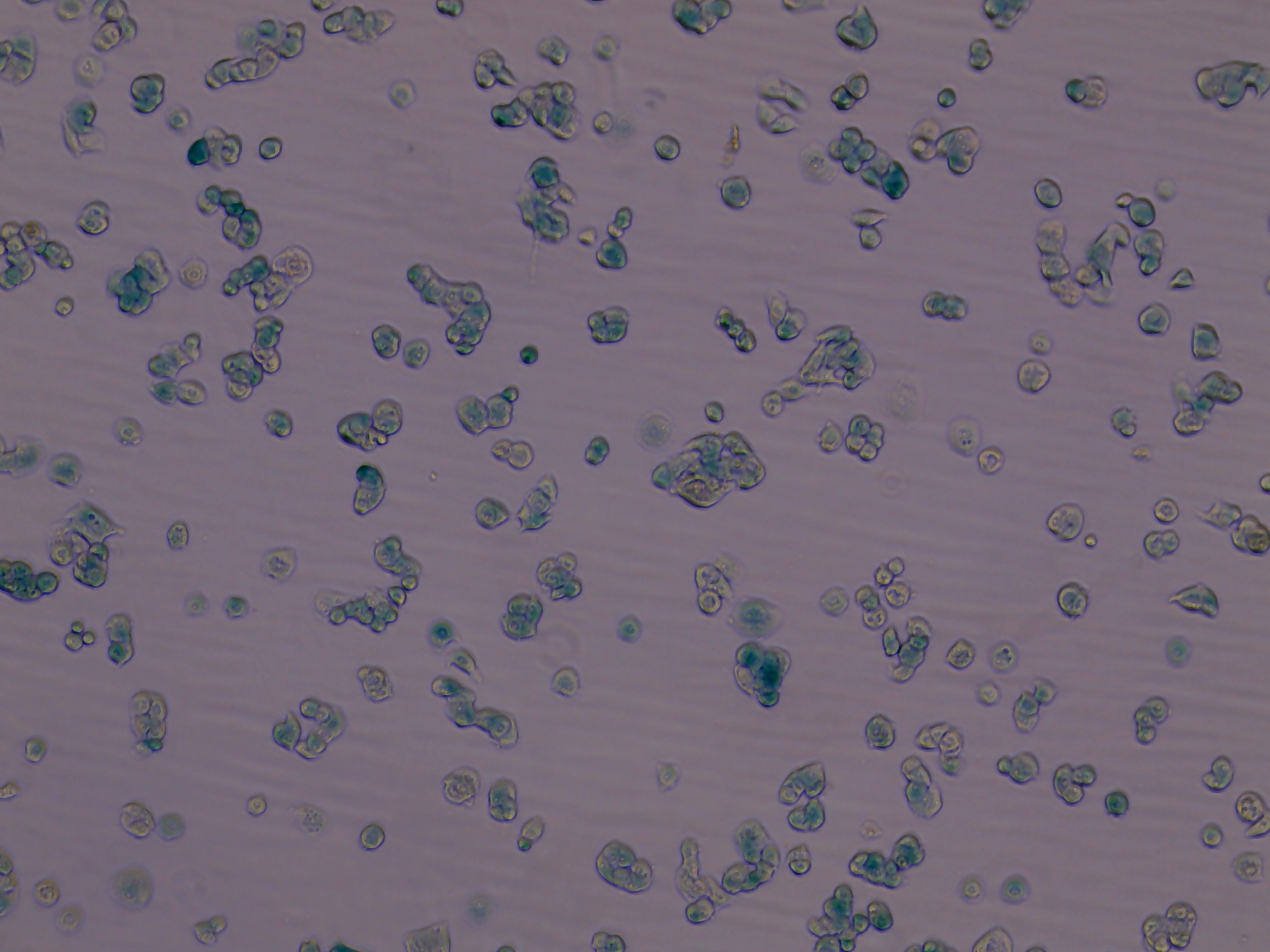

Supplement: Supplementary file 3 — Source data Fig. 2 [file 44318_2025_371_MOESM3_ESM.zip › SourceData_Figure 2/2J/t47d/n=2/t47d abema 20369.jpg]

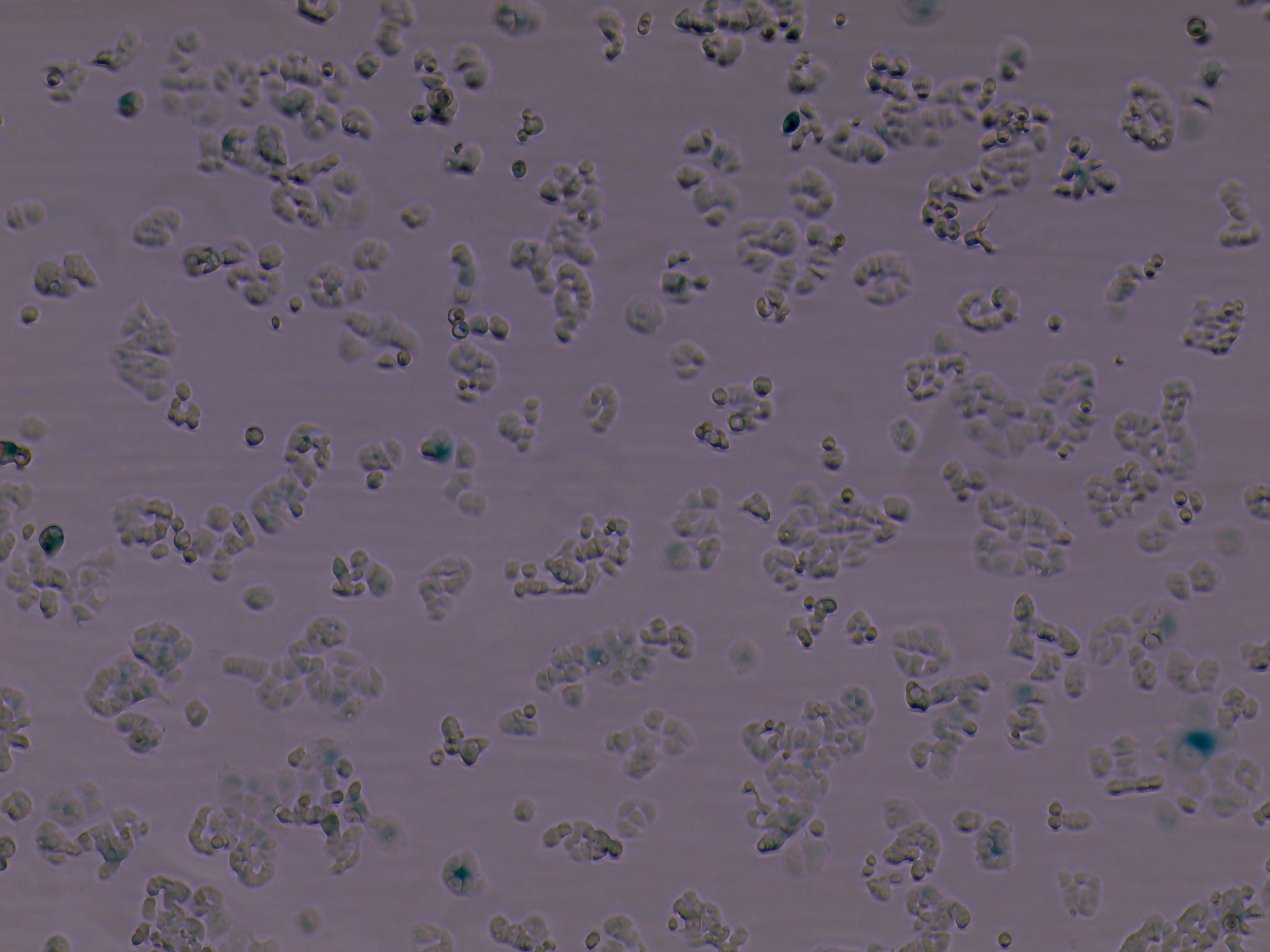

Supplement: Supplementary file 3 — Source data Fig. 2 [file 44318_2025_371_MOESM3_ESM.zip › SourceData_Figure 2/2J/t47d/n=2/t47d ctrl 20363.jpg]

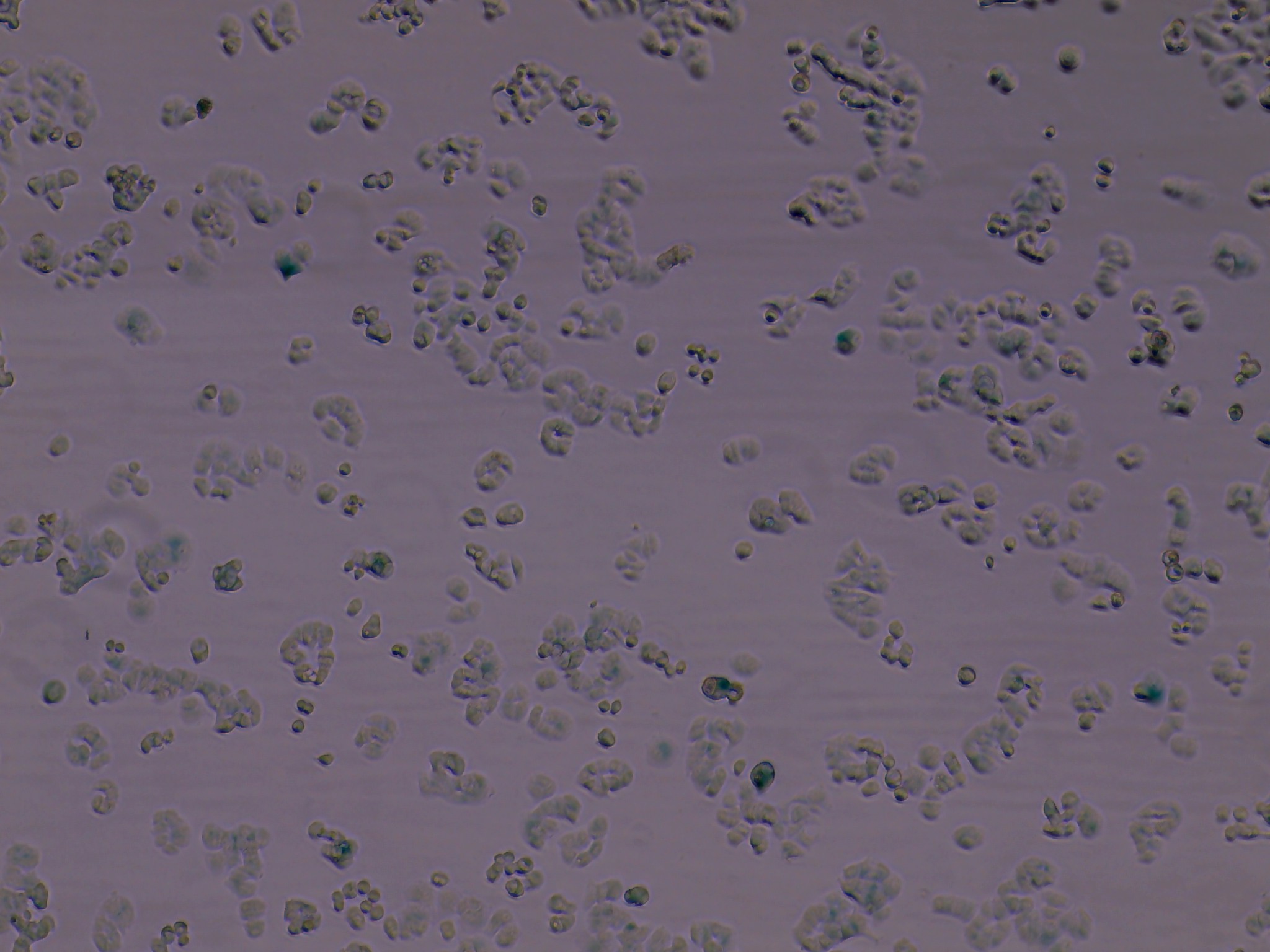

Supplement: Supplementary file 3 — Source data Fig. 2 [file 44318_2025_371_MOESM3_ESM.zip › SourceData_Figure 2/2J/t47d/n=2/t47d ctrl 30364.jpg]

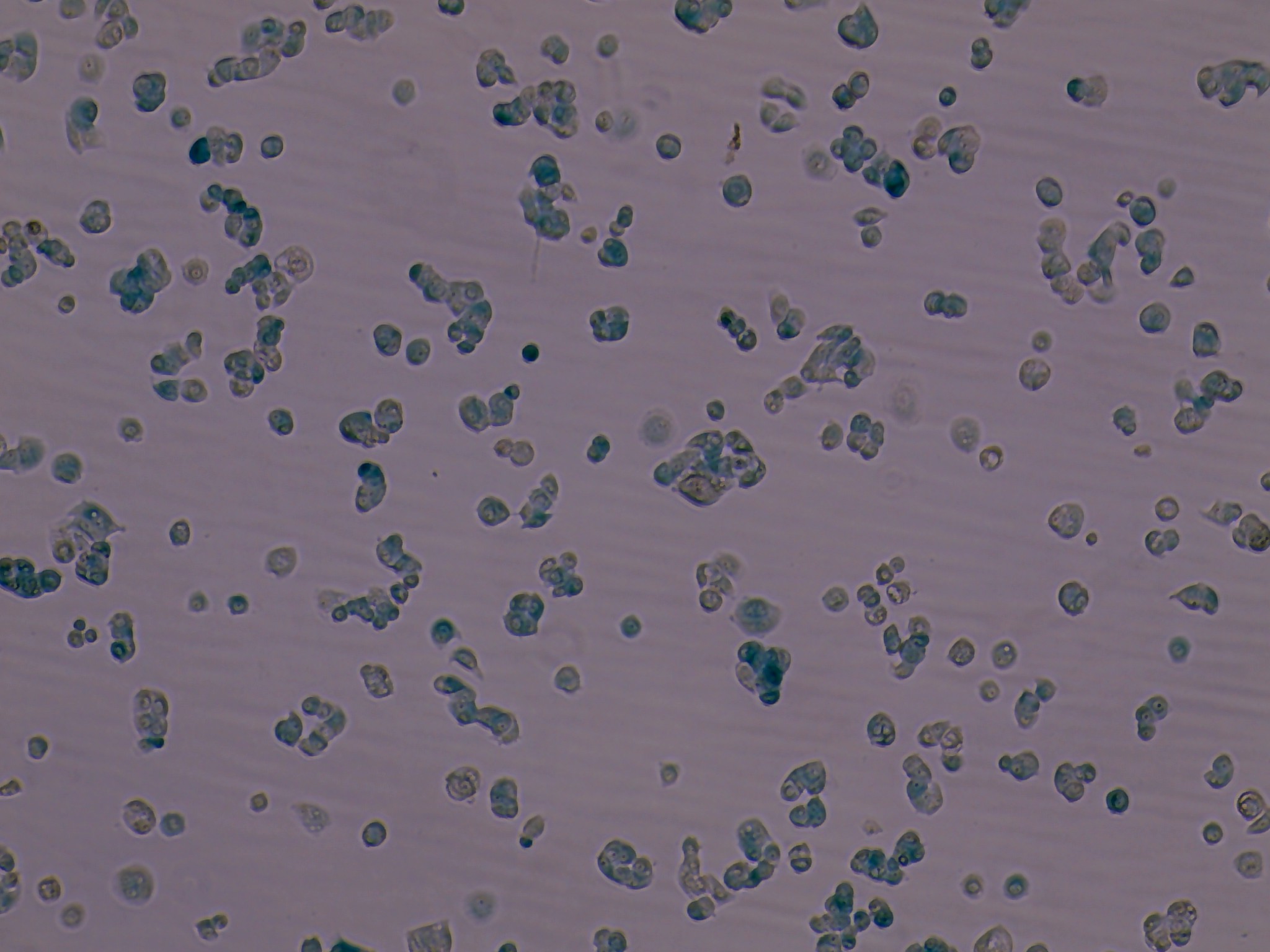

Supplement: Supplementary file 3 — Source data Fig. 2 [file 44318_2025_371_MOESM3_ESM.zip › SourceData_Figure 2/2J/t47d/n=2/t47d abema 10368.jpg]

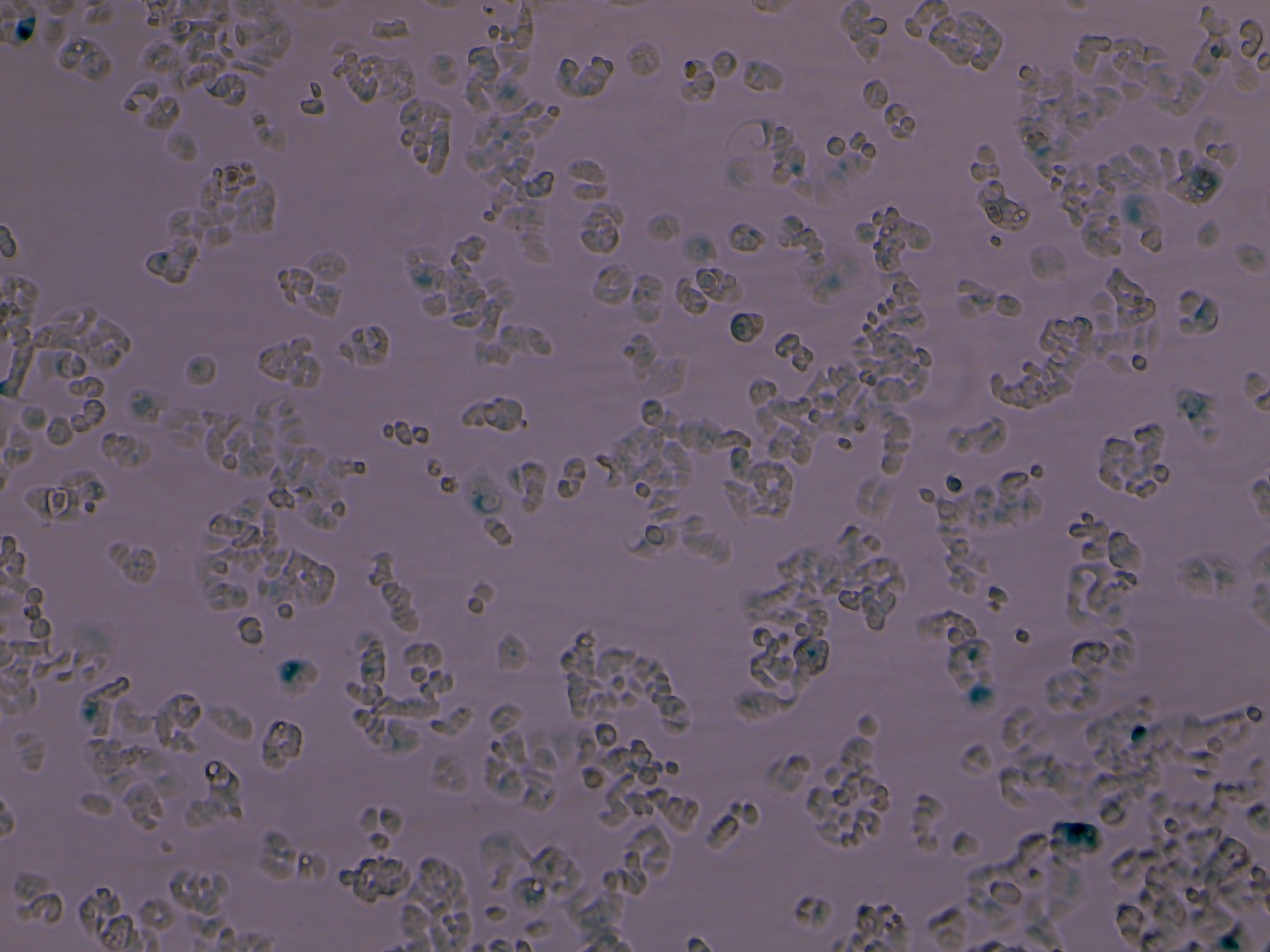

Supplement: Supplementary file 3 — Source data Fig. 2 [file 44318_2025_371_MOESM3_ESM.zip › SourceData_Figure 2/2J/t47d/n=2/t47d ctrl 10362.jpg]

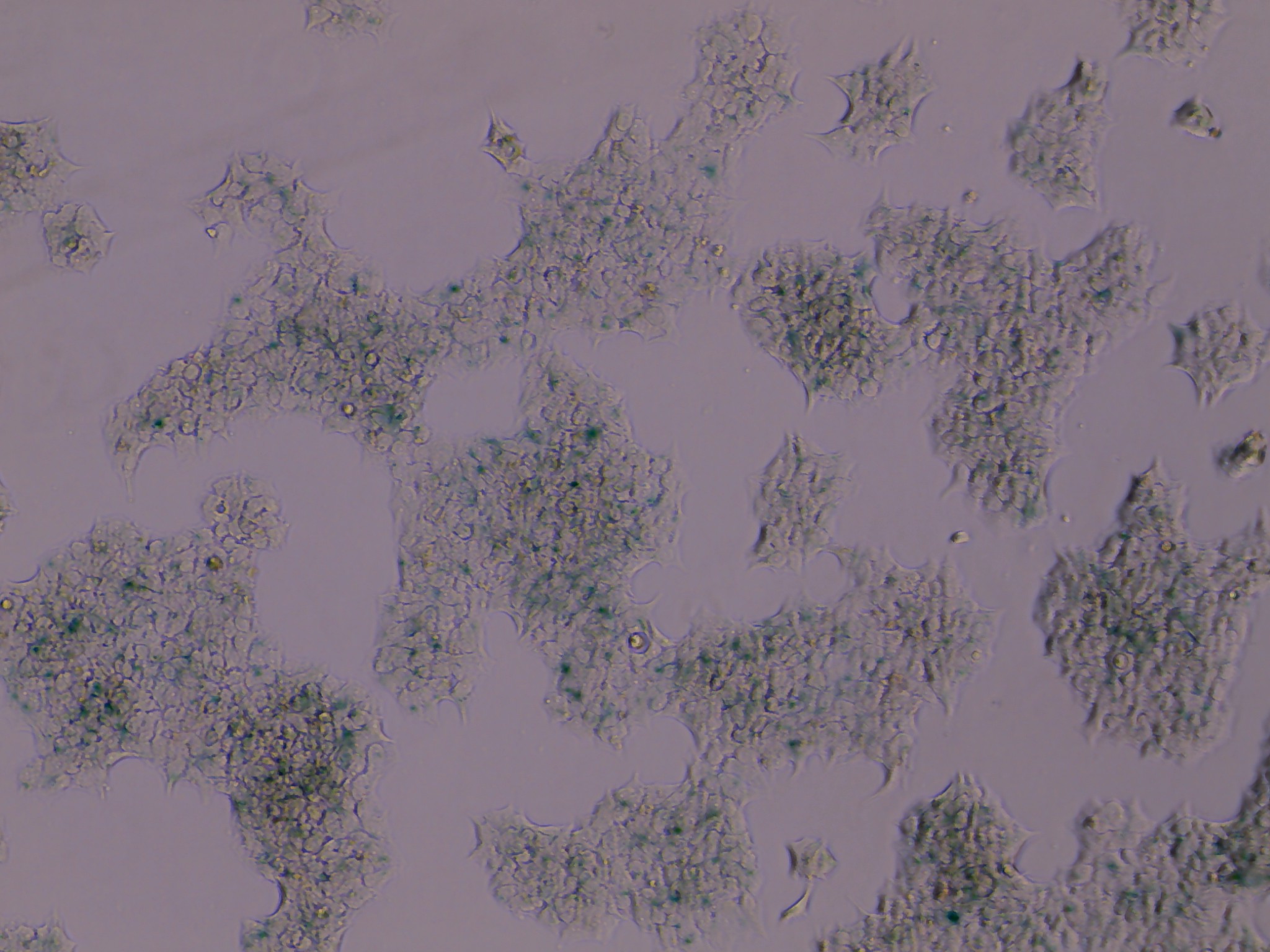

Supplement: Supplementary file 3 — Source data Fig. 2 [file 44318_2025_371_MOESM3_ESM.zip › SourceData_Figure 2/2J/bt747/n=1/bt747 abema 10375.jpg]

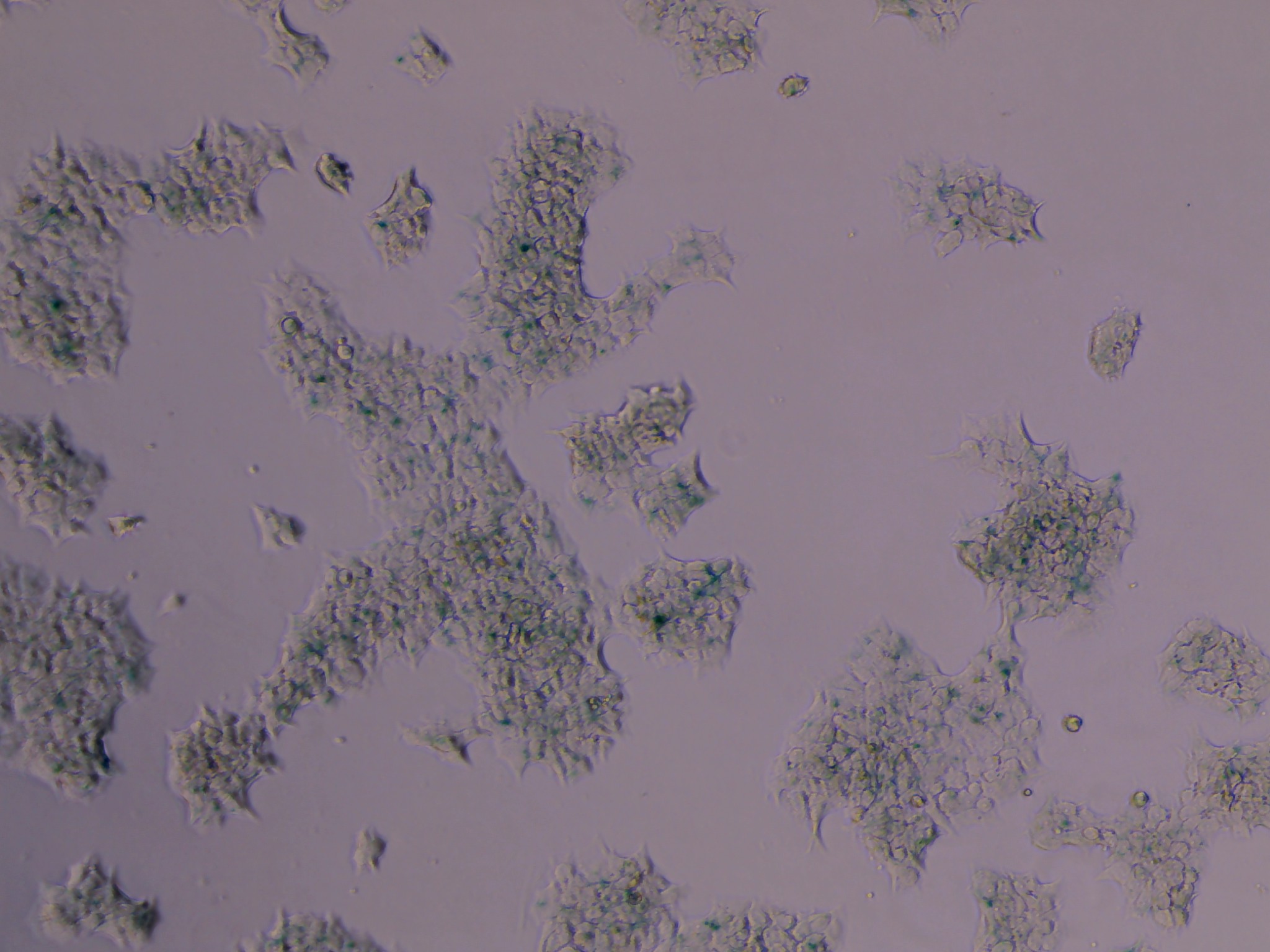

Supplement: Supplementary file 3 — Source data Fig. 2 [file 44318_2025_371_MOESM3_ESM.zip › SourceData_Figure 2/2J/bt747/n=1/bt747 abema 30377.jpg]

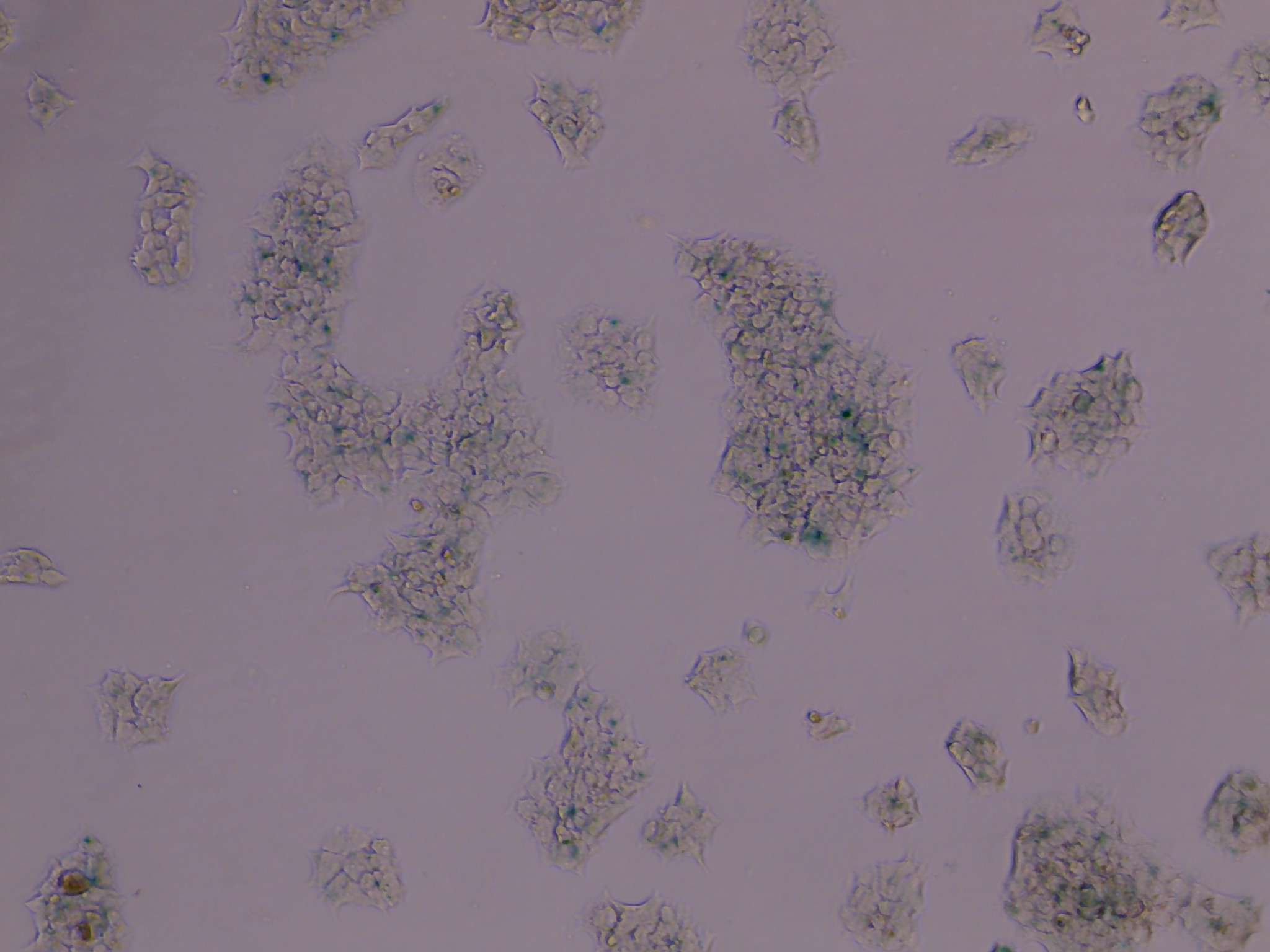

Supplement: Supplementary file 3 — Source data Fig. 2 [file 44318_2025_371_MOESM3_ESM.zip › SourceData_Figure 2/2J/bt747/n=1/bt747 abema 20376.jpg]

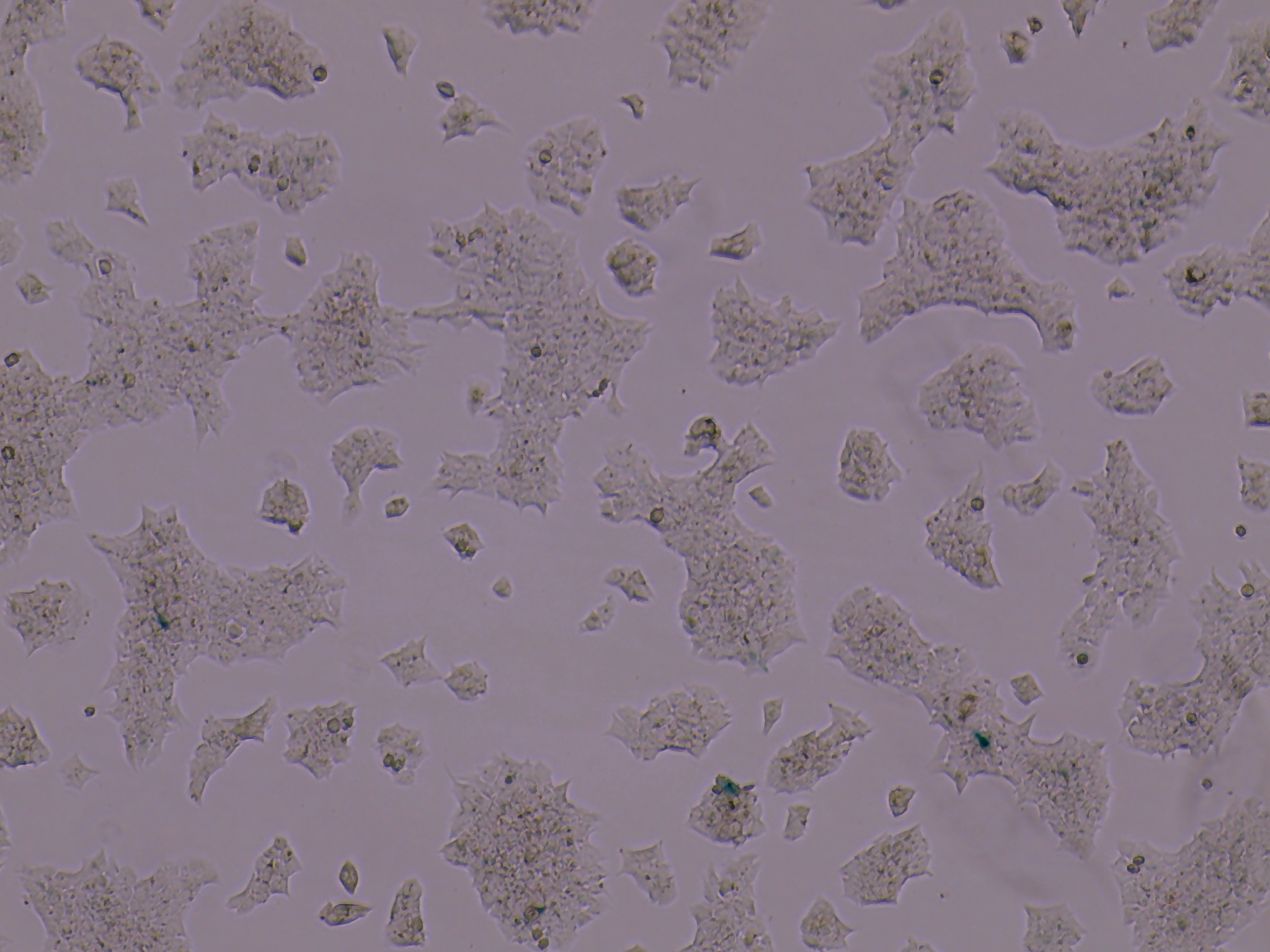

Supplement: Supplementary file 3 — Source data Fig. 2 [file 44318_2025_371_MOESM3_ESM.zip › SourceData_Figure 2/2J/bt747/n=1/bt747 ctrl 20383.jpg]

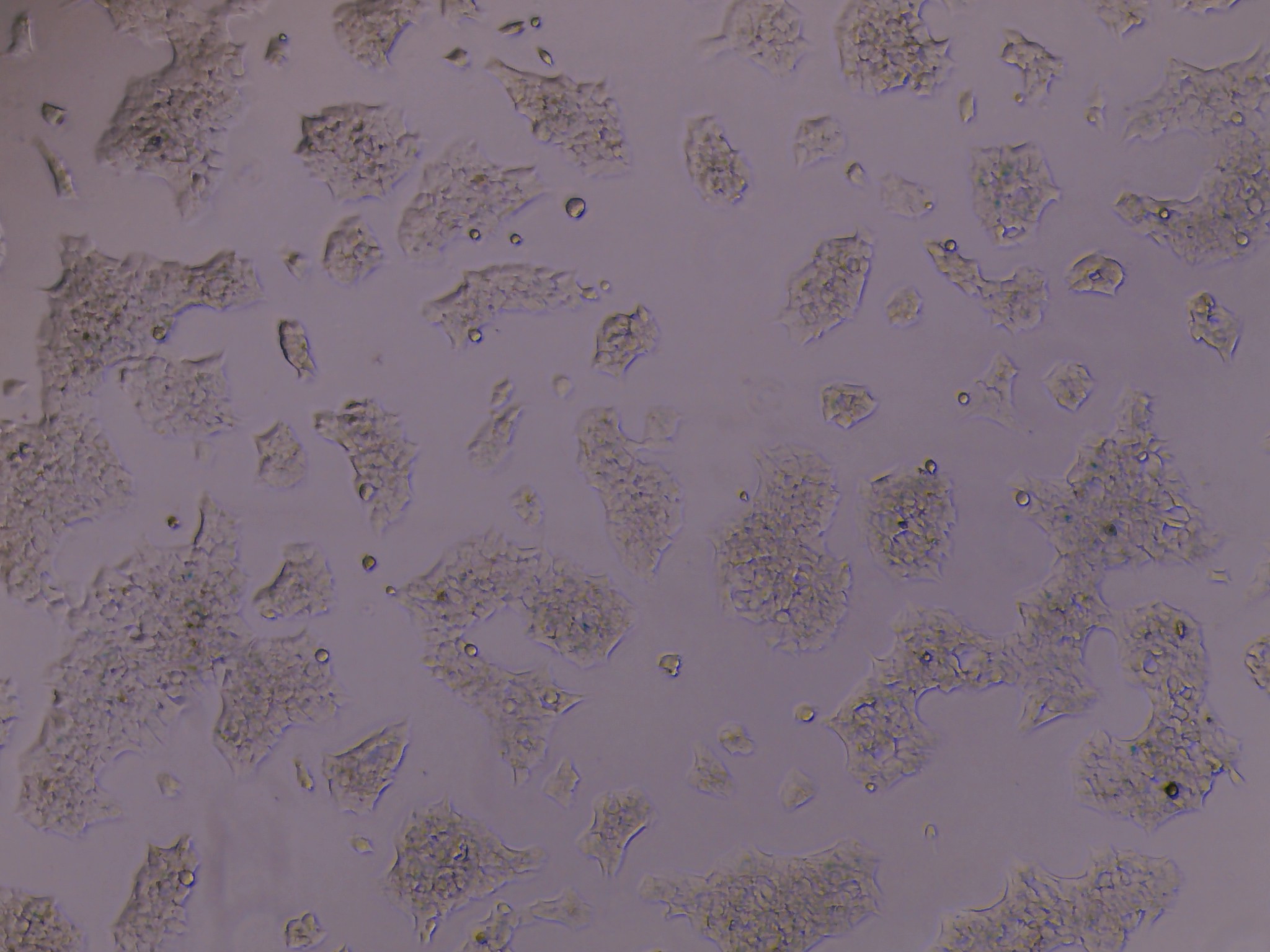

Supplement: Supplementary file 3 — Source data Fig. 2 [file 44318_2025_371_MOESM3_ESM.zip › SourceData_Figure 2/2J/bt747/n=1/bt747 ctrl 10382.jpg]

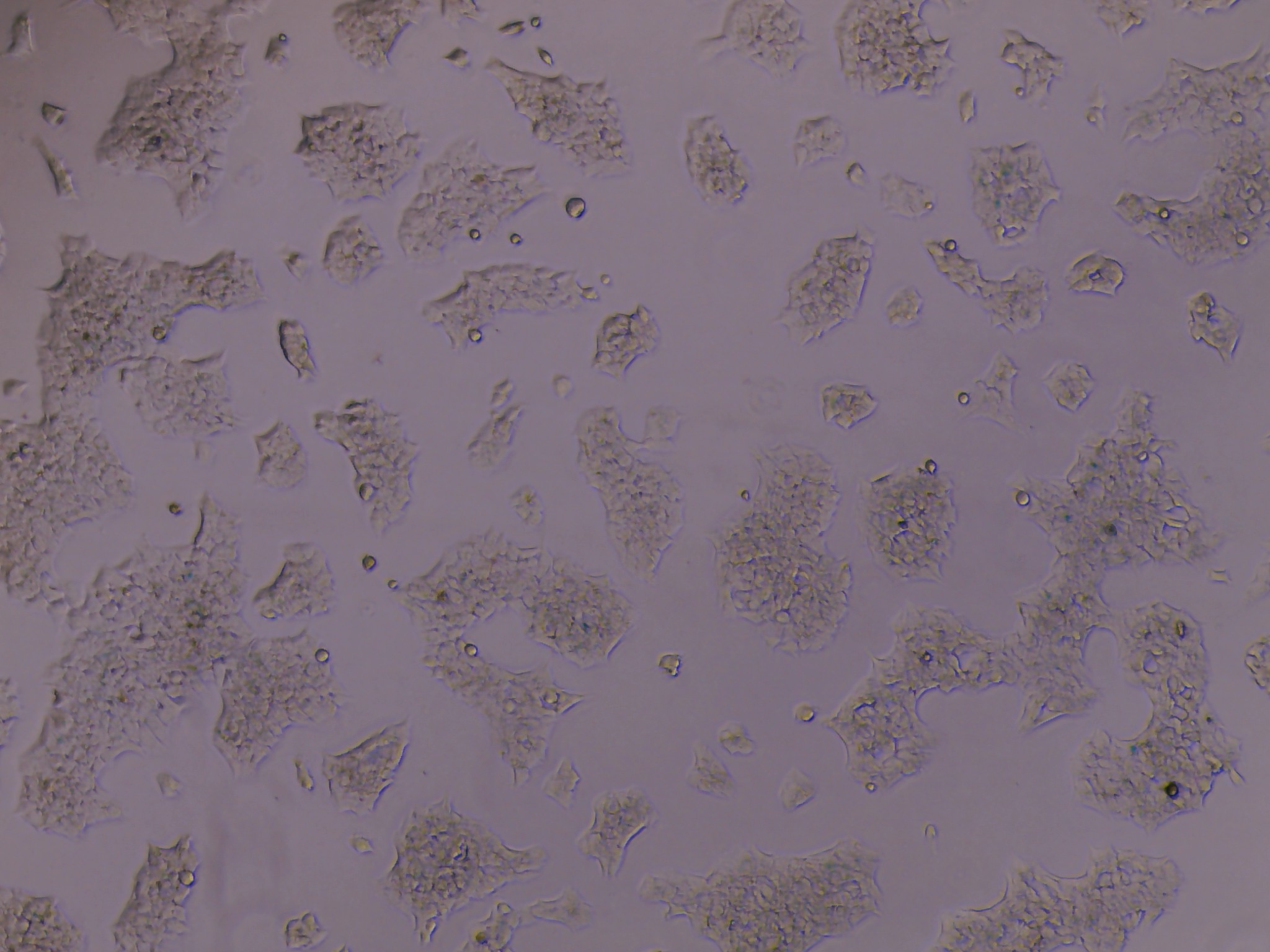

Supplement: Supplementary file 3 — Source data Fig. 2 [file 44318_2025_371_MOESM3_ESM.zip › SourceData_Figure 2/2J/bt747/n=1/bt747 ctrl 10381.jpg]

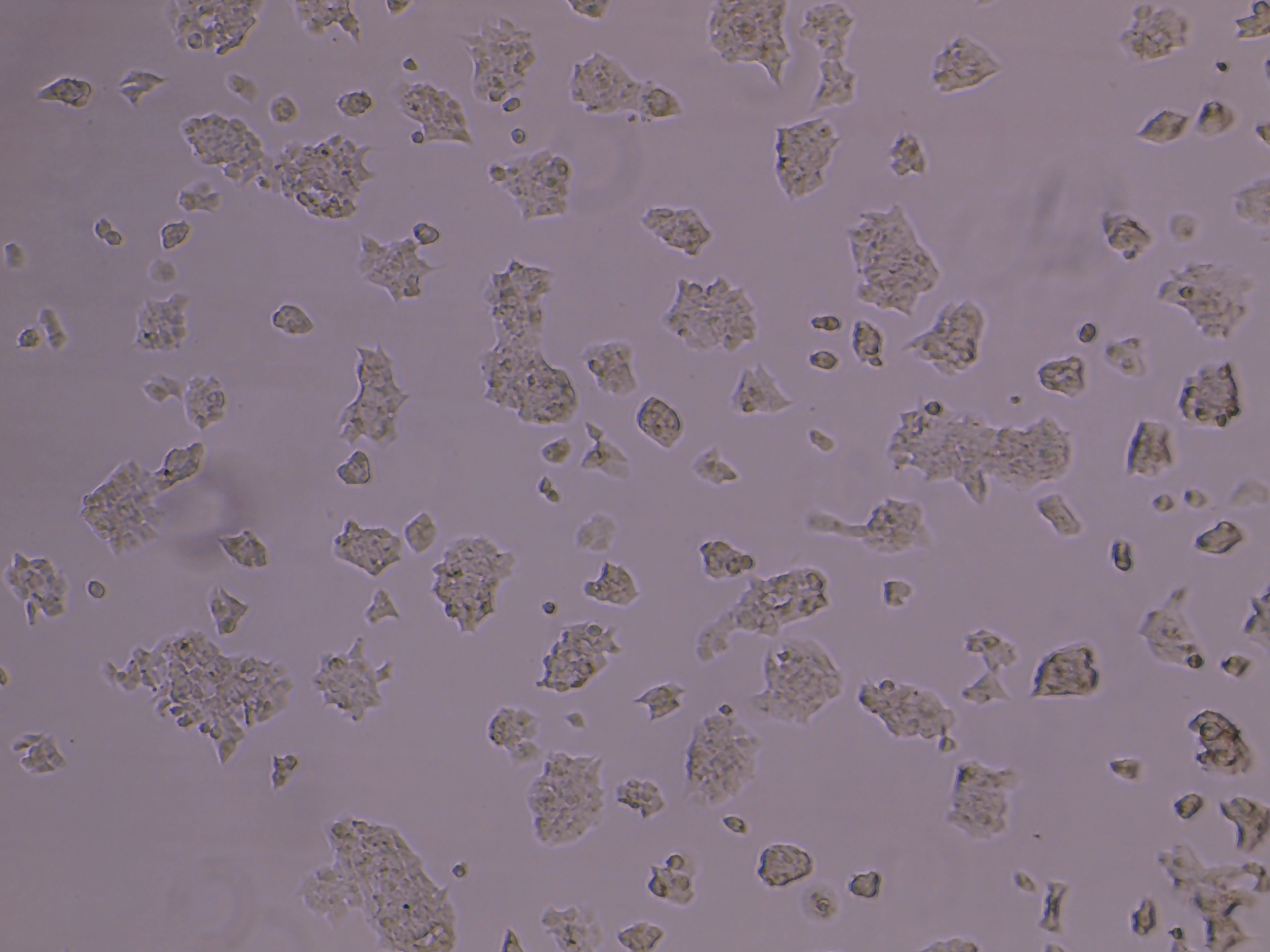

Supplement: Supplementary file 3 — Source data Fig. 2 [file 44318_2025_371_MOESM3_ESM.zip › SourceData_Figure 2/2J/bt747/n=2/bt747 ctrl 50386.jpg]

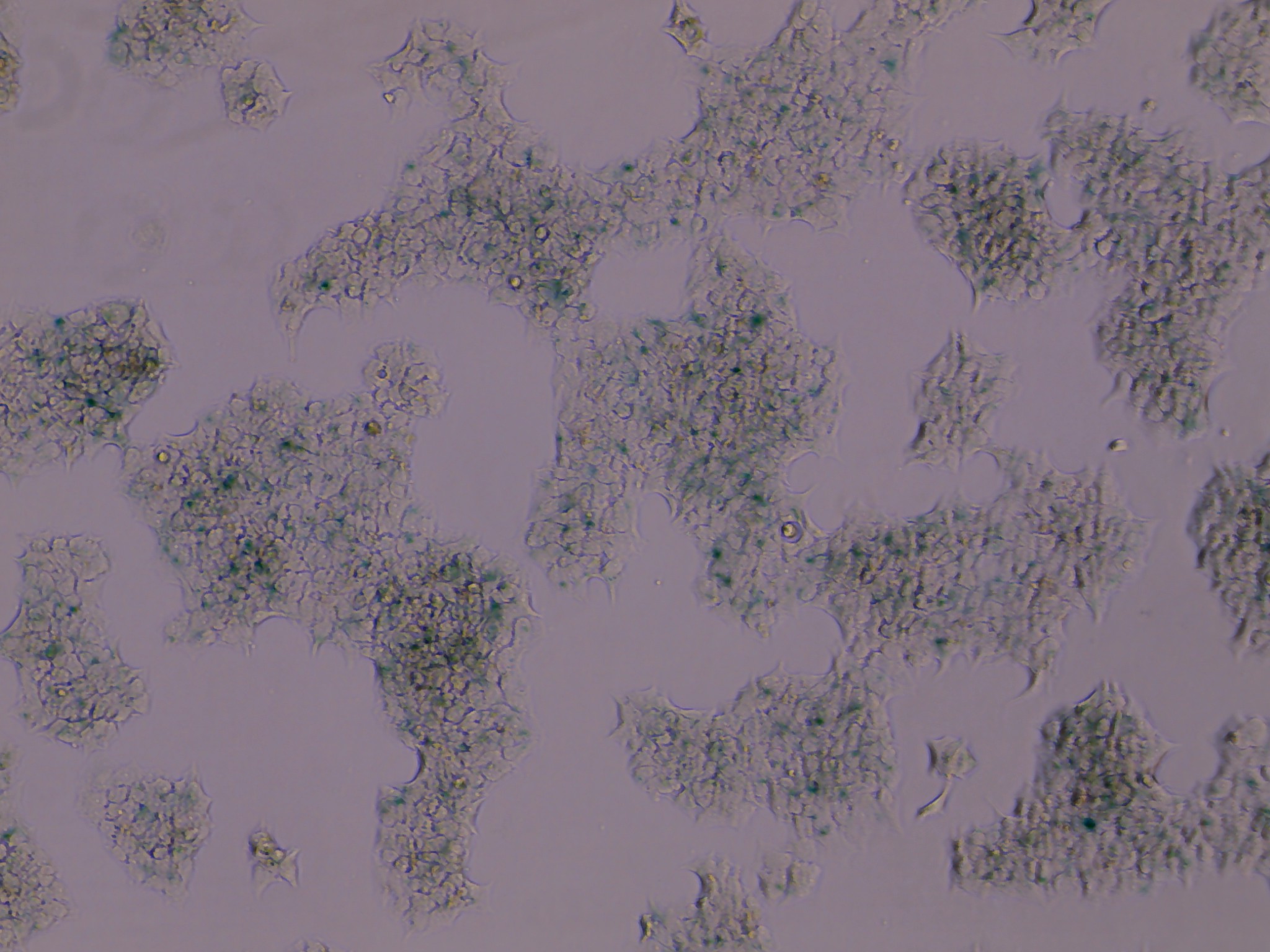

Supplement: Supplementary file 3 — Source data Fig. 2 [file 44318_2025_371_MOESM3_ESM.zip › SourceData_Figure 2/2J/bt747/n=2/bt747 abema 50379.jpg]

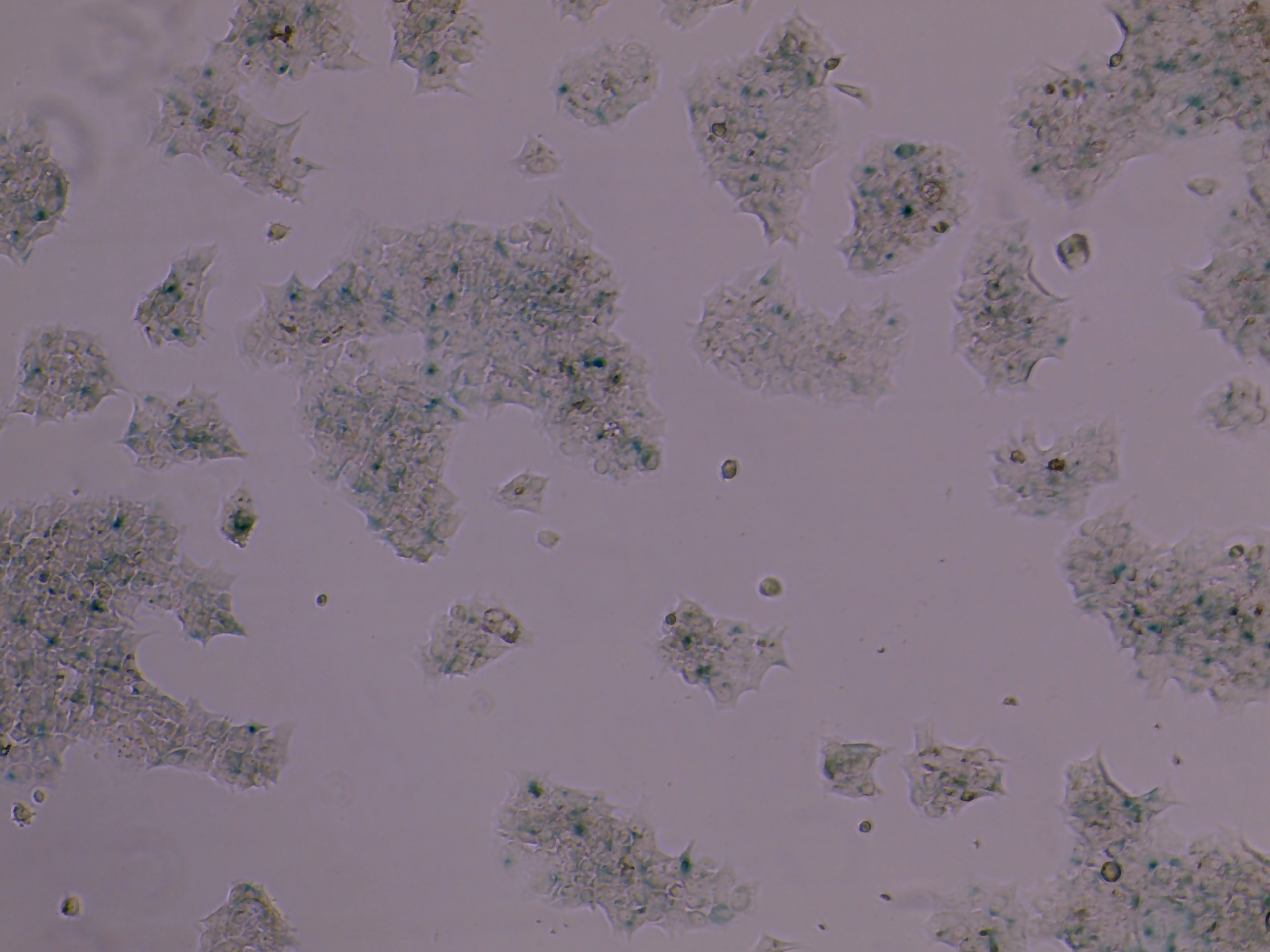

Supplement: Supplementary file 3 — Source data Fig. 2 [file 44318_2025_371_MOESM3_ESM.zip › SourceData_Figure 2/2J/bt747/n=2/bt747 abema 40378.jpg]

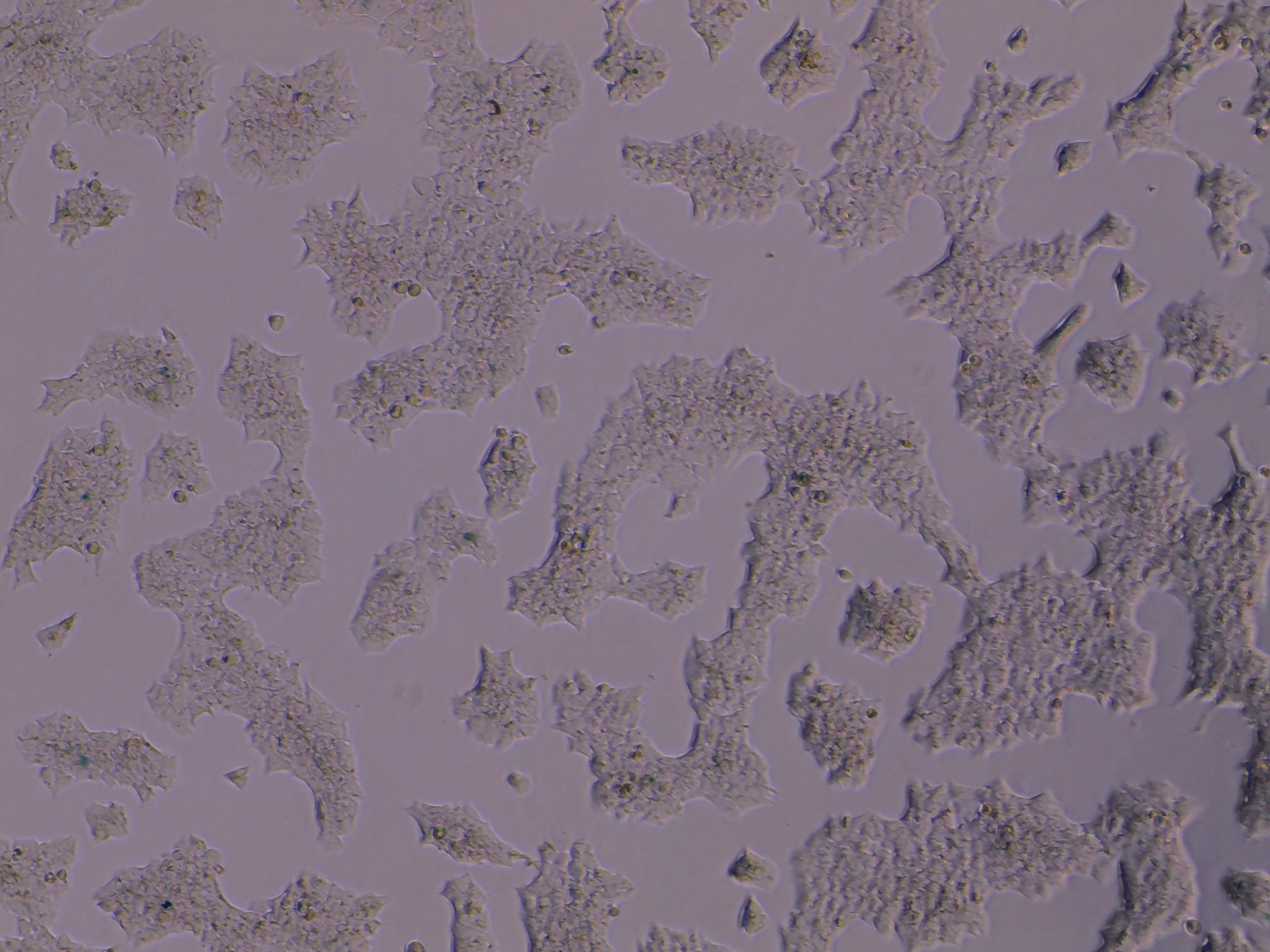

Supplement: Supplementary file 3 — Source data Fig. 2 [file 44318_2025_371_MOESM3_ESM.zip › SourceData_Figure 2/2J/bt747/n=2/bt747 ctrl 40385.jpg]

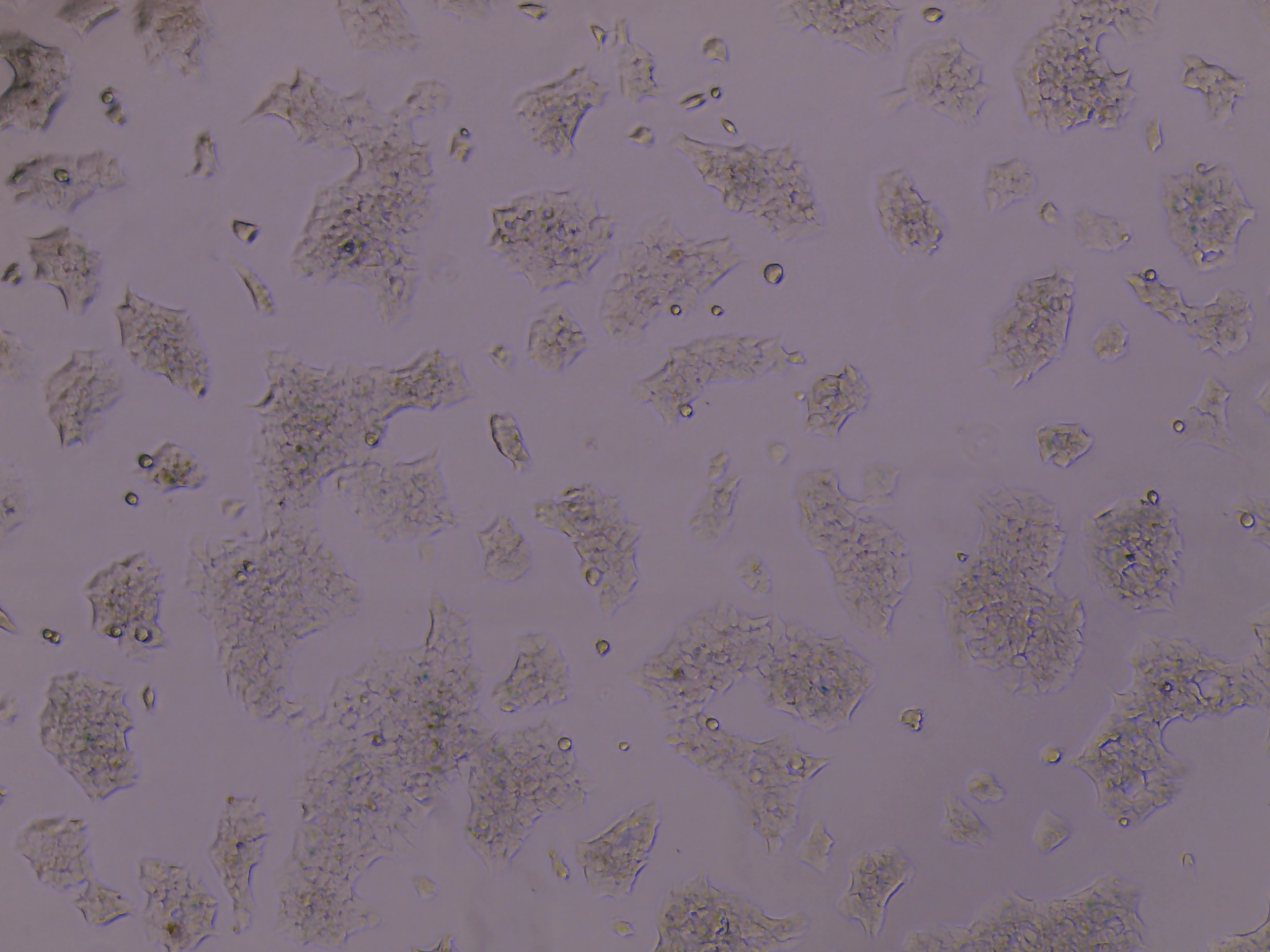

Supplement: Supplementary file 3 — Source data Fig. 2 [file 44318_2025_371_MOESM3_ESM.zip › SourceData_Figure 2/2J/bt747/n=2/bt747 ctrl 60387.jpg]

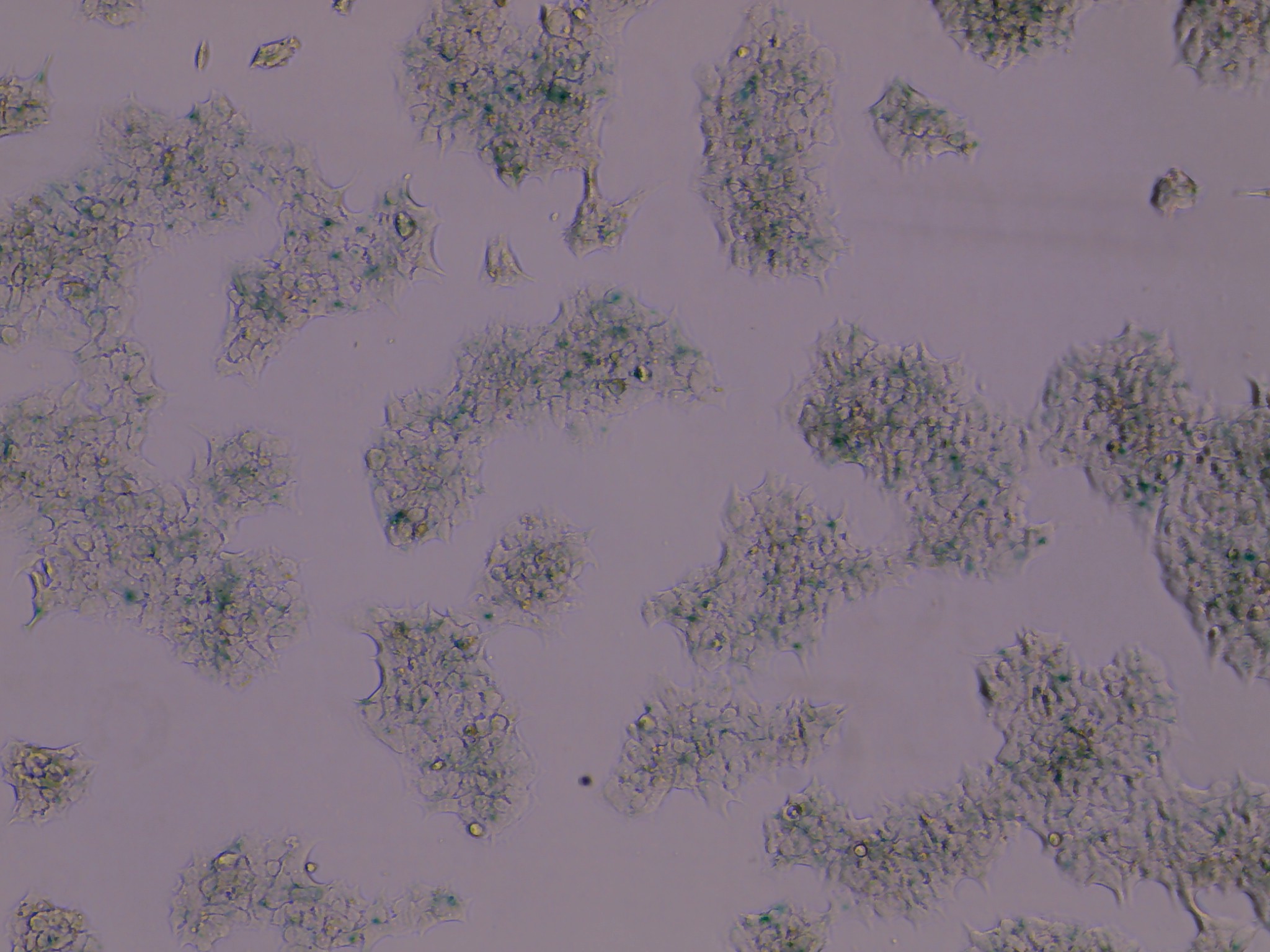

Supplement: Supplementary file 3 — Source data Fig. 2 [file 44318_2025_371_MOESM3_ESM.zip › SourceData_Figure 2/2J/bt747/n=2/bt747 abema 60380.jpg]

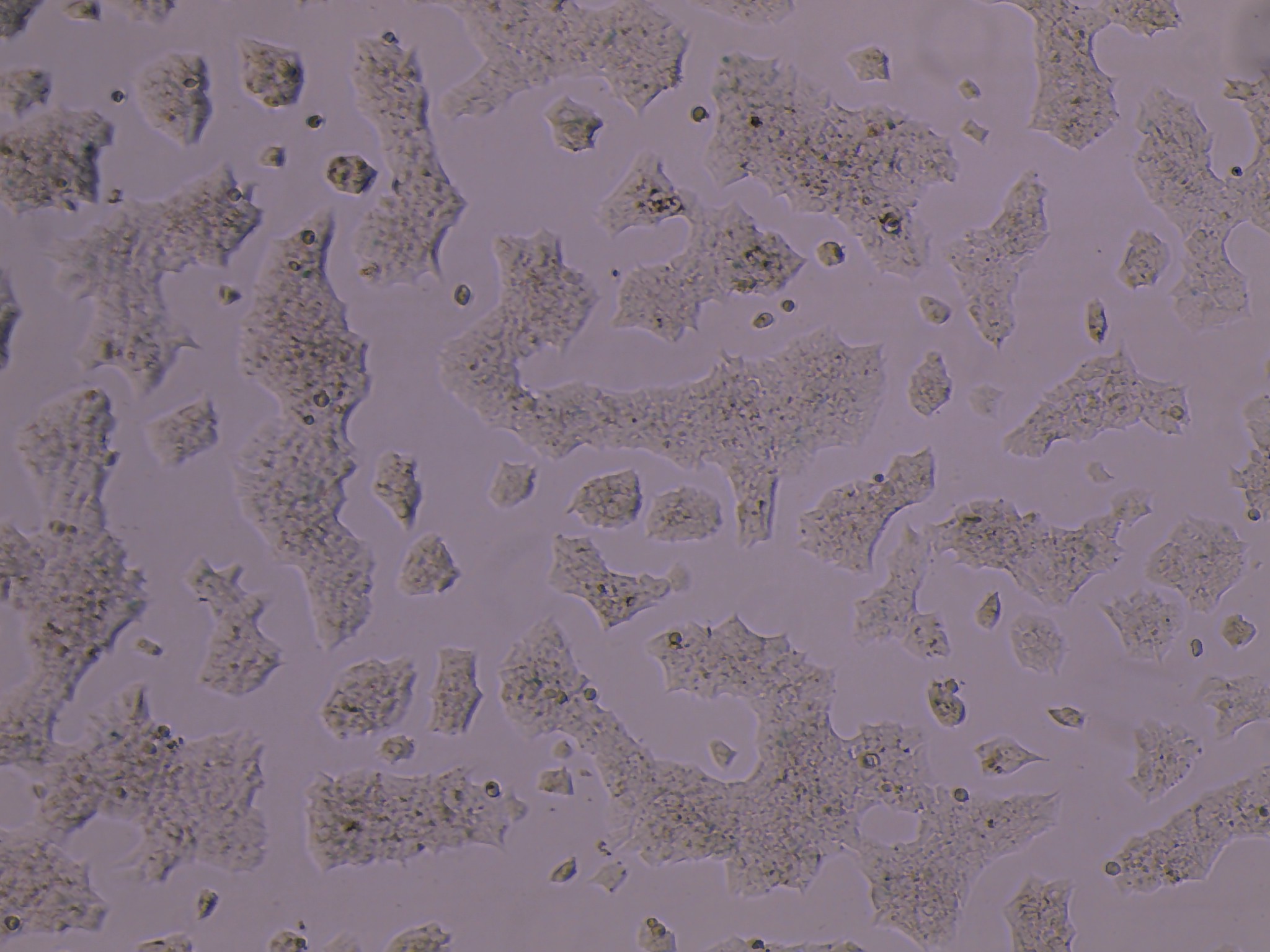

Supplement: Supplementary file 3 — Source data Fig. 2 [file 44318_2025_371_MOESM3_ESM.zip › SourceData_Figure 2/2J/bt747/n=2/bt747 ctrl 70388.jpg]

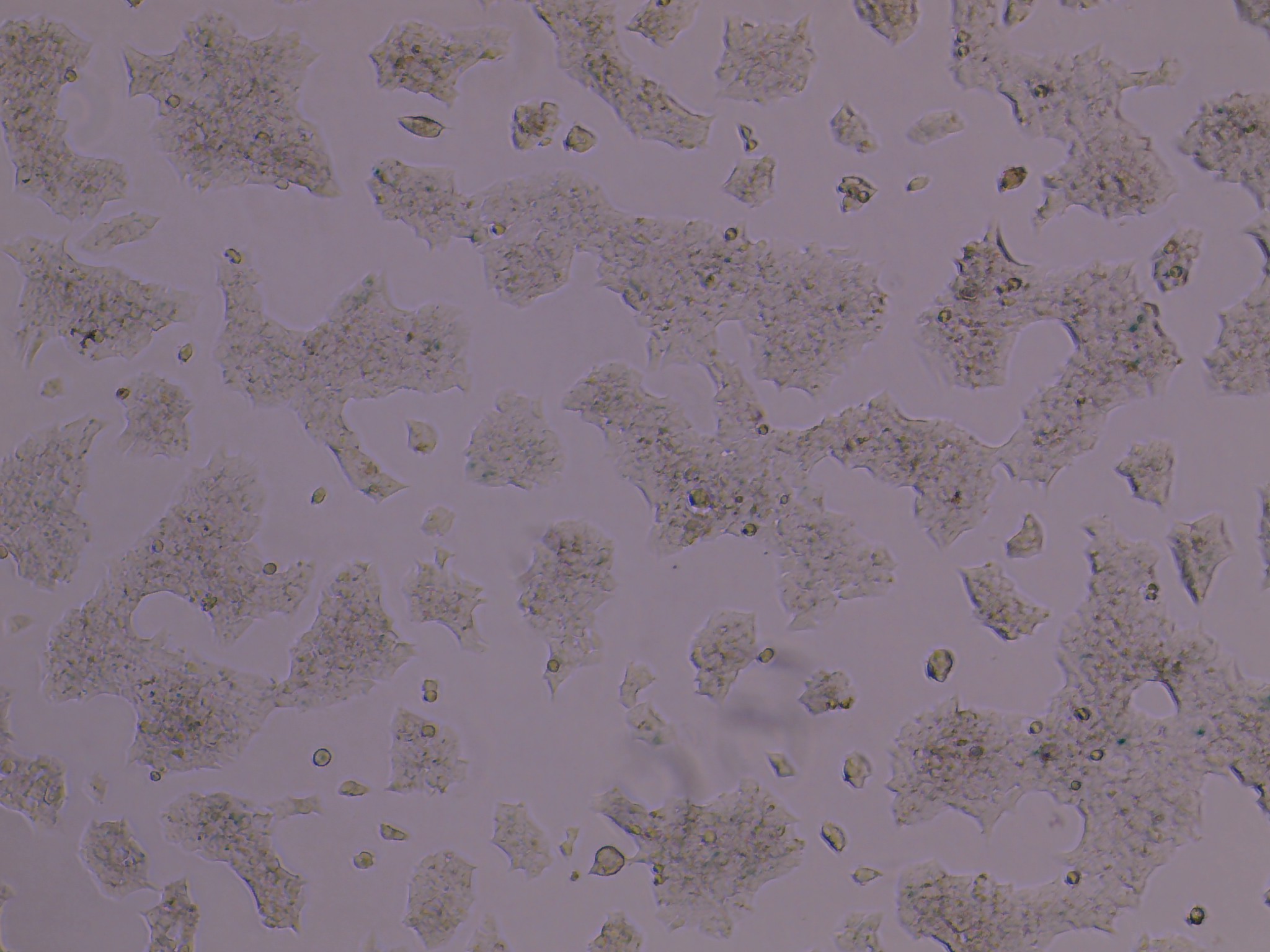

Supplement: Supplementary file 3 — Source data Fig. 2 [file 44318_2025_371_MOESM3_ESM.zip › SourceData_Figure 2/2J/bt747/n=2/bt747 ctrl 30384.jpg]

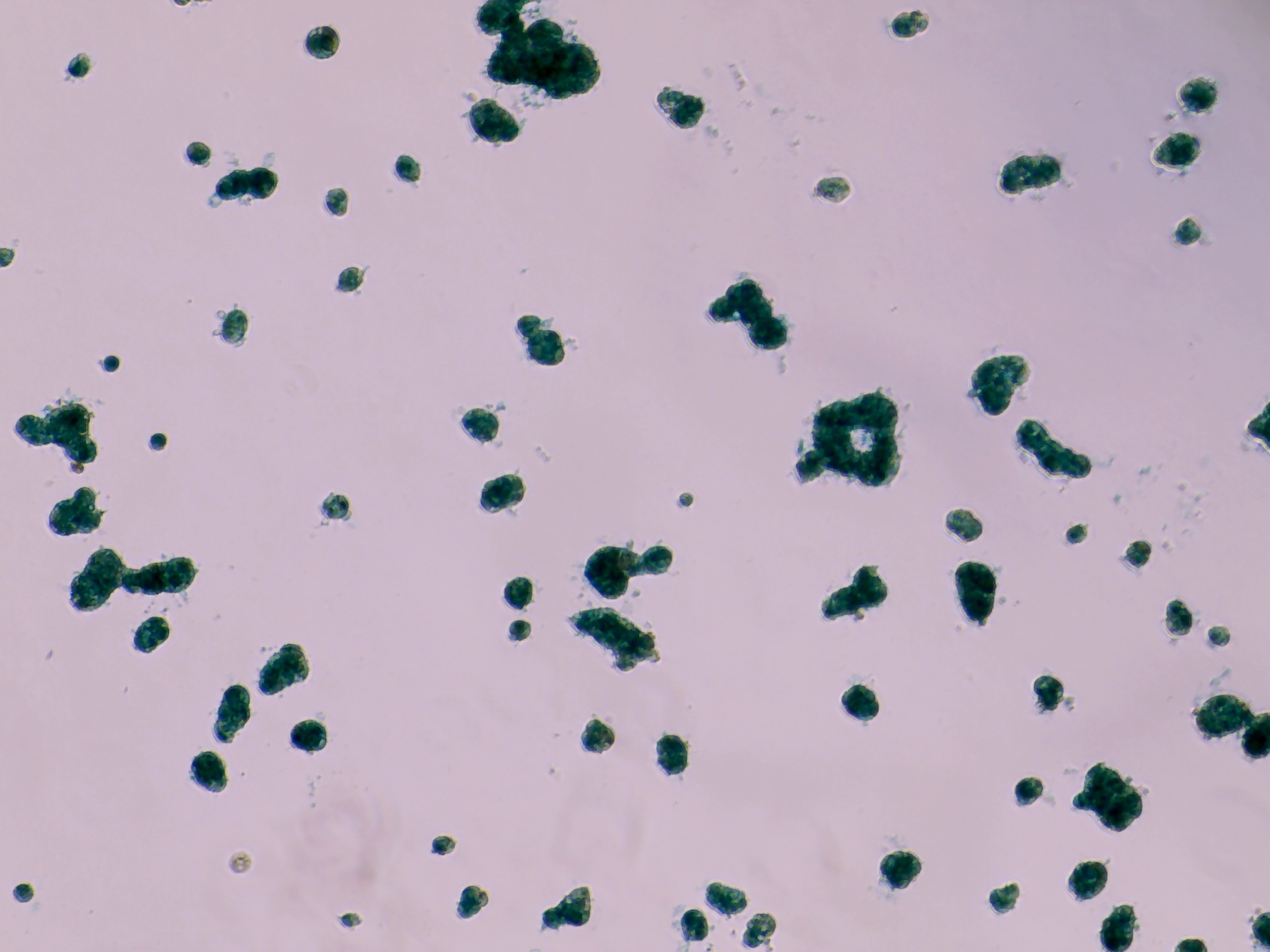

Supplement: Supplementary file 3 — Source data Fig. 2 [file 44318_2025_371_MOESM3_ESM.zip › SourceData_Figure 2/2J/ZR-75-30/n=1/zr75-1 abema 20231.jpg]

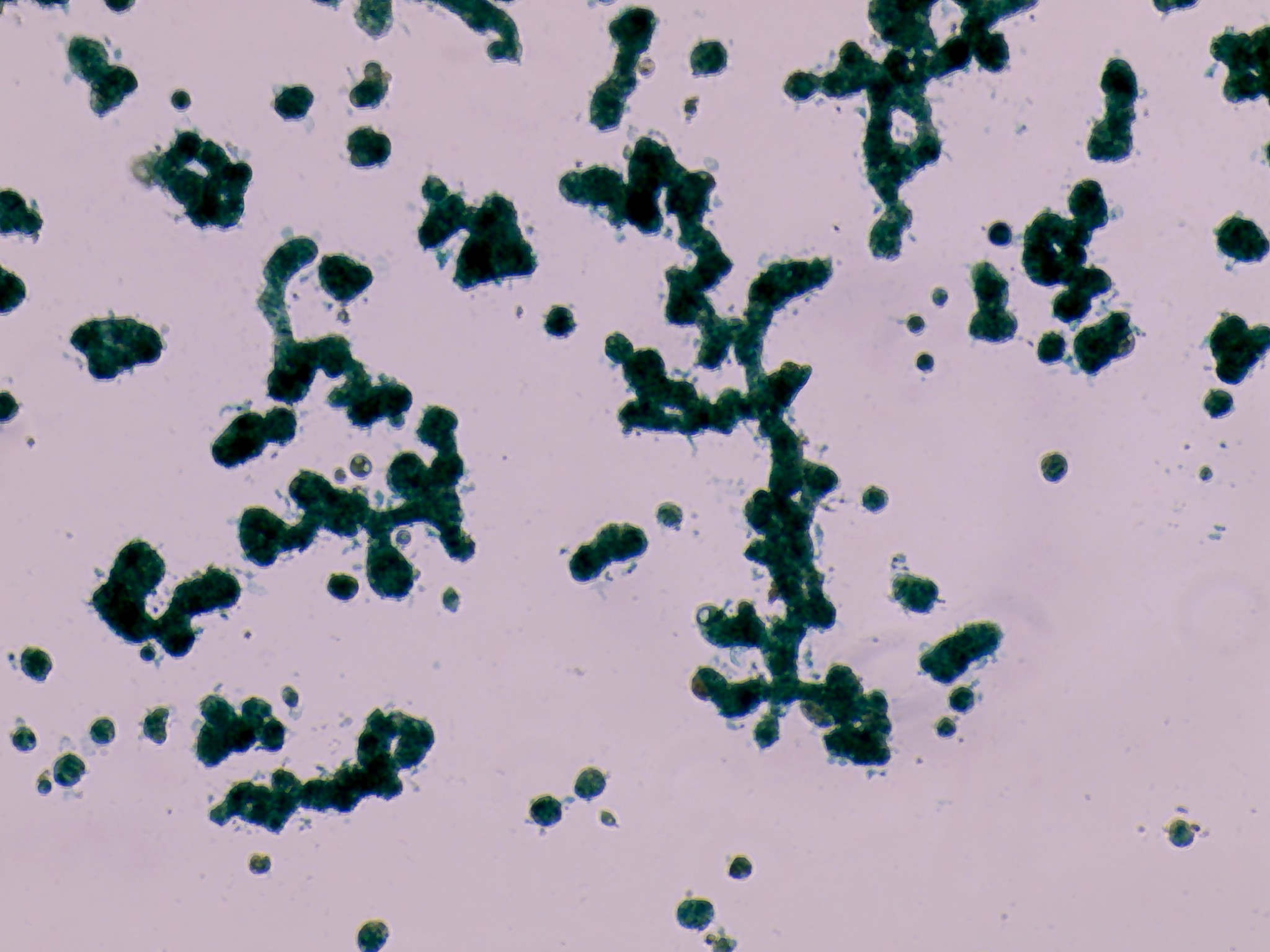

Supplement: Supplementary file 3 — Source data Fig. 2 [file 44318_2025_371_MOESM3_ESM.zip › SourceData_Figure 2/2J/ZR-75-30/n=1/zr75-1 abema 30232.jpg]

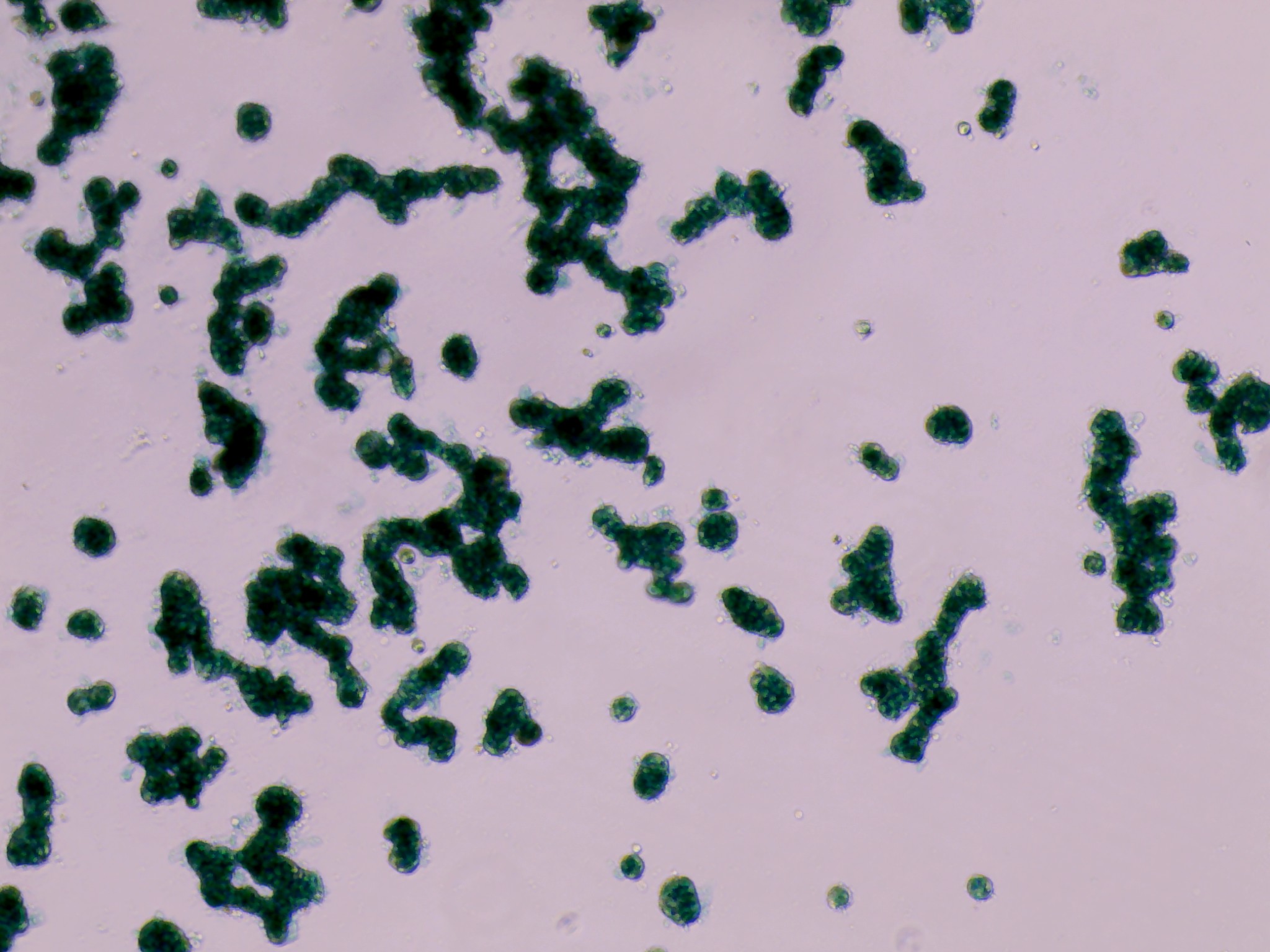

Supplement: Supplementary file 3 — Source data Fig. 2 [file 44318_2025_371_MOESM3_ESM.zip › SourceData_Figure 2/2J/ZR-75-30/n=1/zr75-1 abema 10230.jpg]

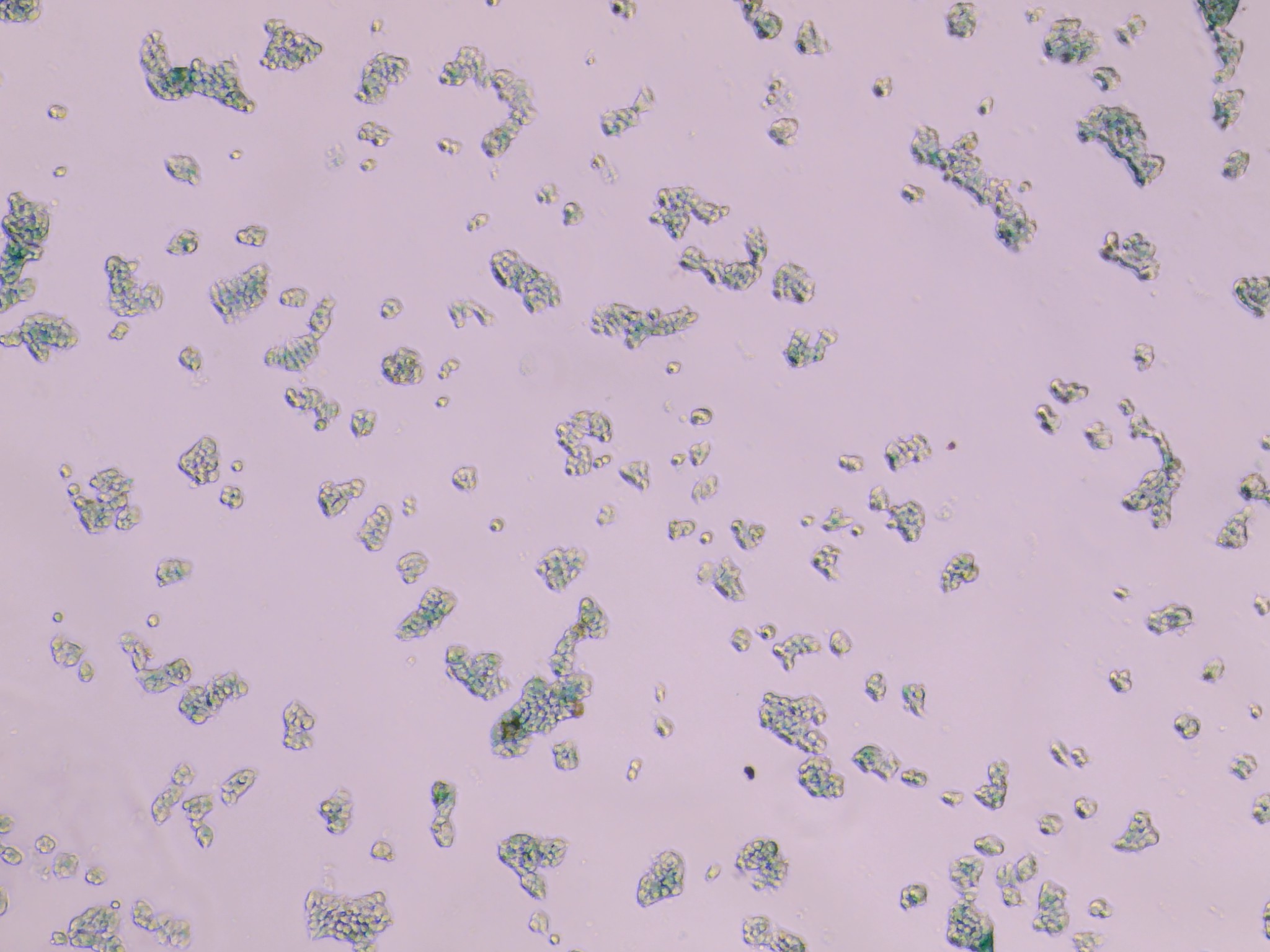

Supplement: Supplementary file 3 — Source data Fig. 2 [file 44318_2025_371_MOESM3_ESM.zip › SourceData_Figure 2/2J/ZR-75-30/n=1/zr75-1 ctrl 30228.jpg]

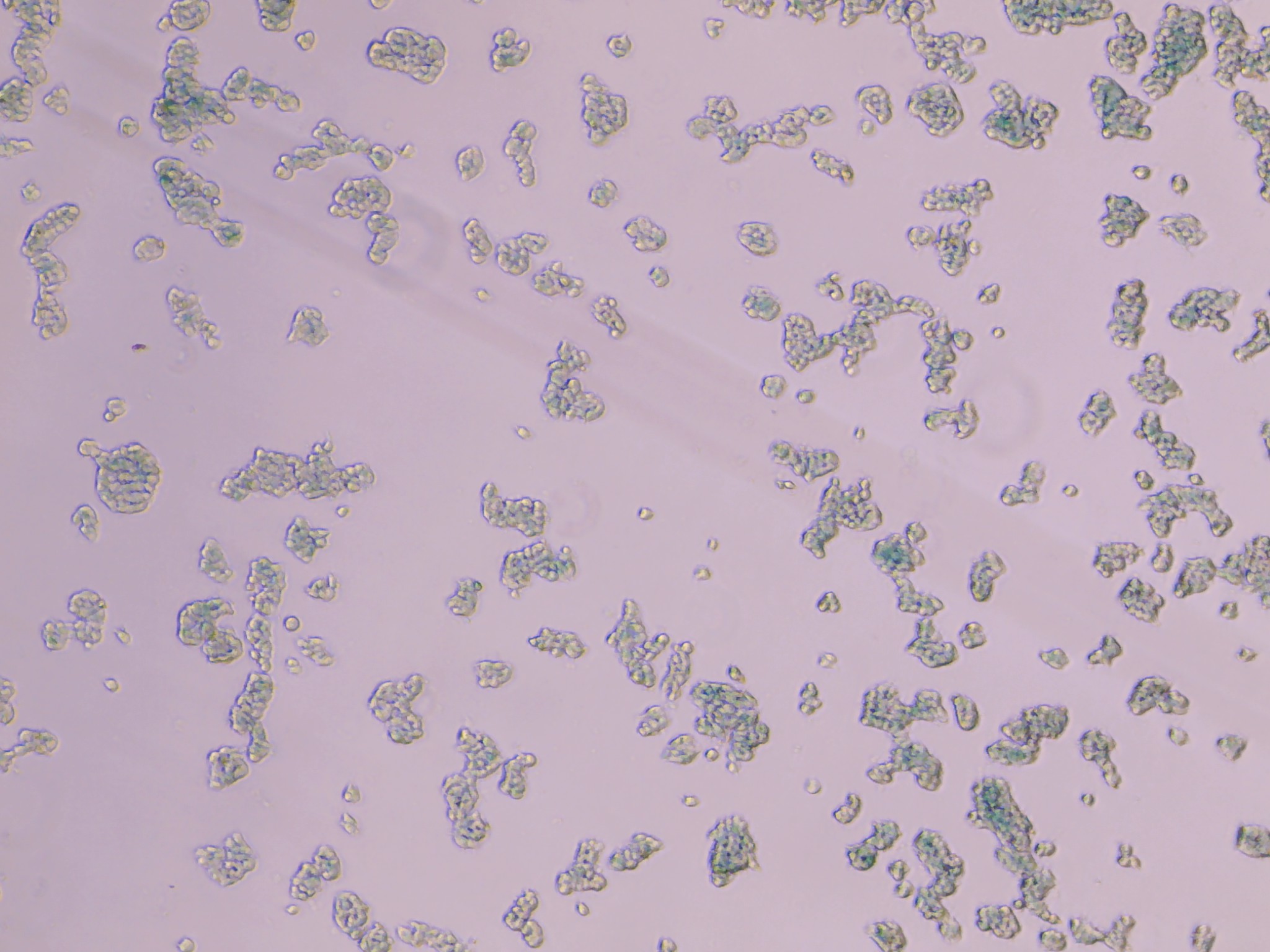

Supplement: Supplementary file 3 — Source data Fig. 2 [file 44318_2025_371_MOESM3_ESM.zip › SourceData_Figure 2/2J/ZR-75-30/n=1/zr75-1 ctrl 20227.jpg]

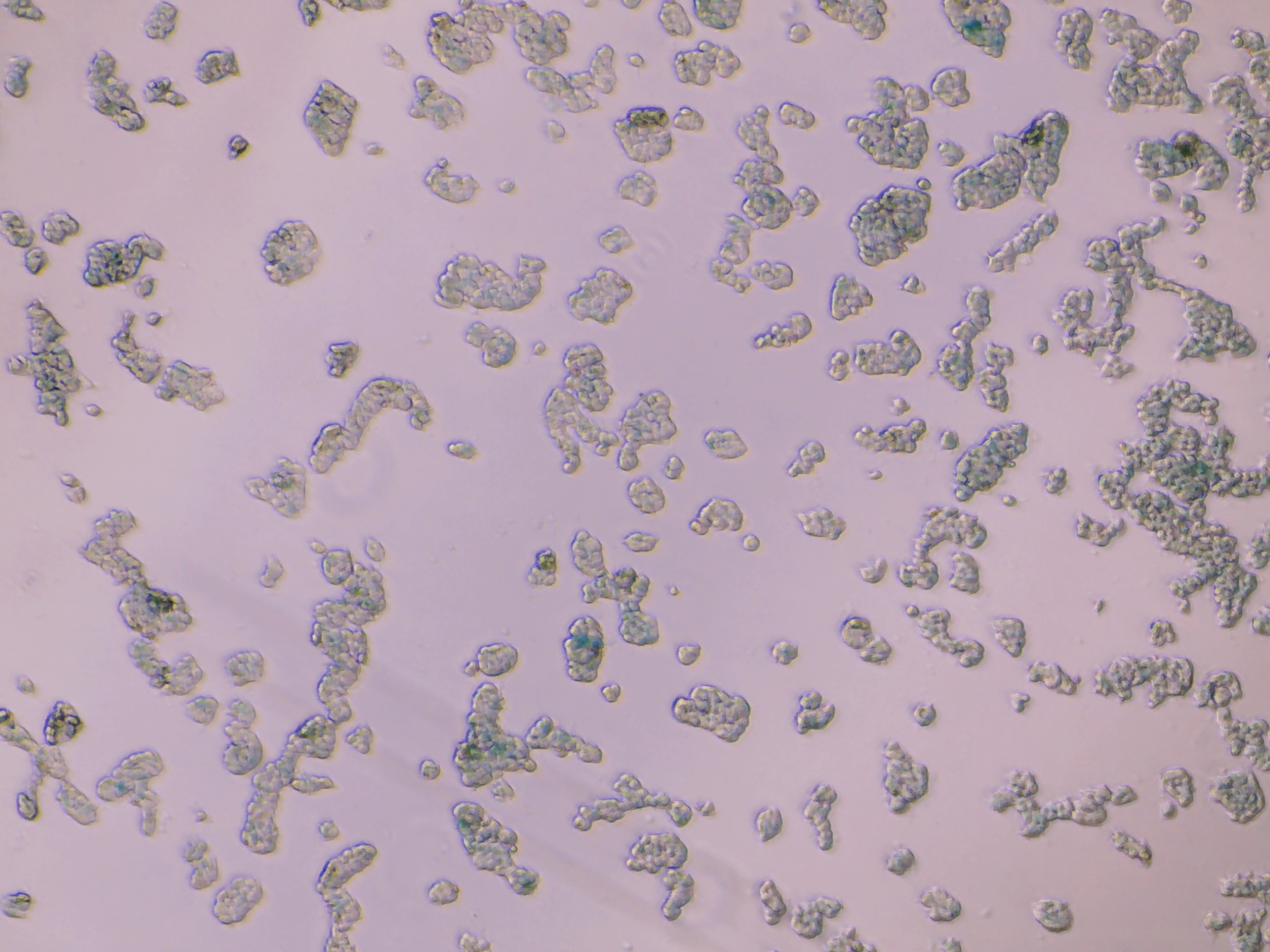

Supplement: Supplementary file 3 — Source data Fig. 2 [file 44318_2025_371_MOESM3_ESM.zip › SourceData_Figure 2/2J/ZR-75-30/n=1/zr75-1 ctrl 40229.jpg]

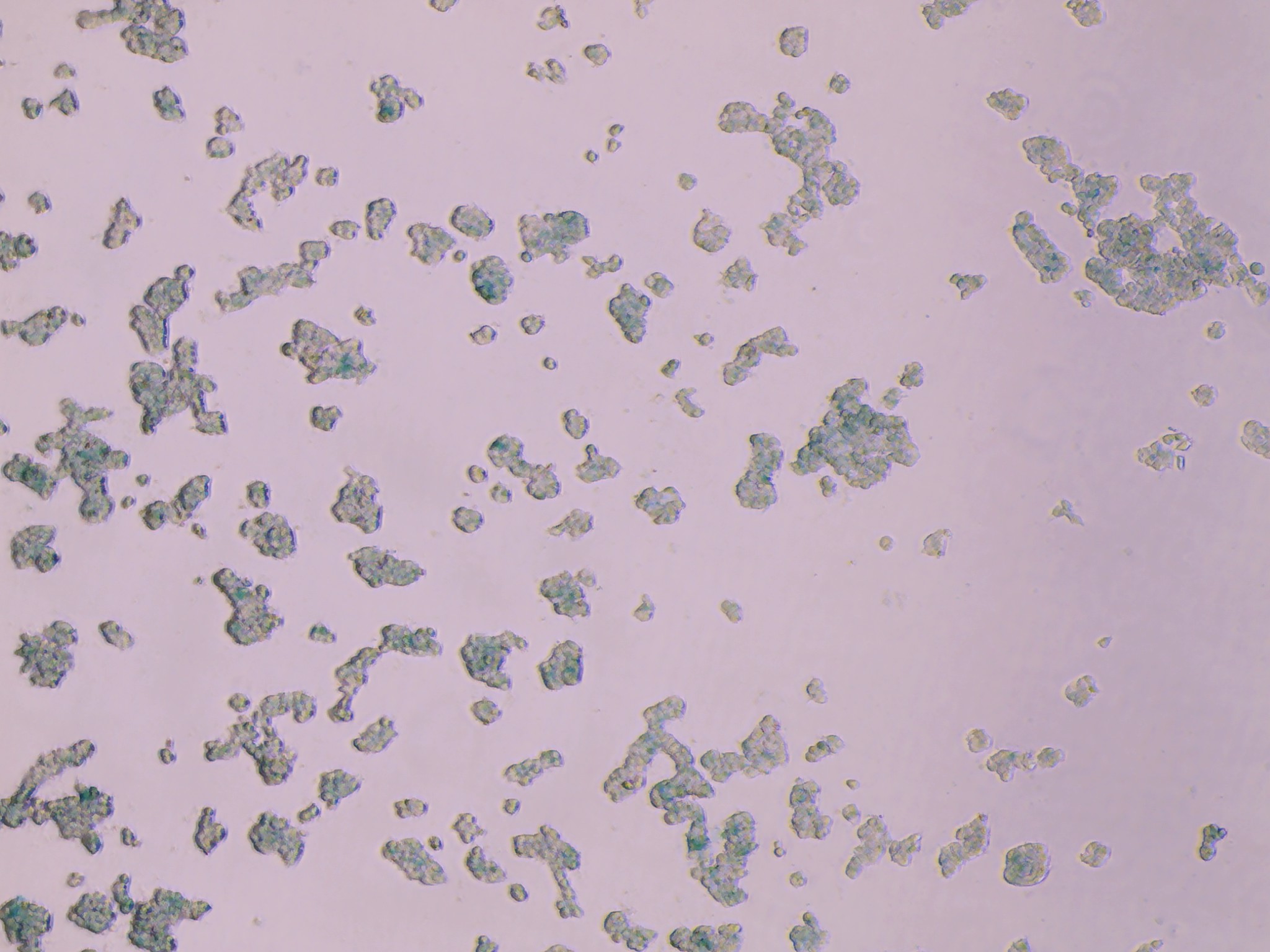

Supplement: Supplementary file 3 — Source data Fig. 2 [file 44318_2025_371_MOESM3_ESM.zip › SourceData_Figure 2/2J/ZR-75-30/n=1/zr75-1 ctrl 10226.jpg]

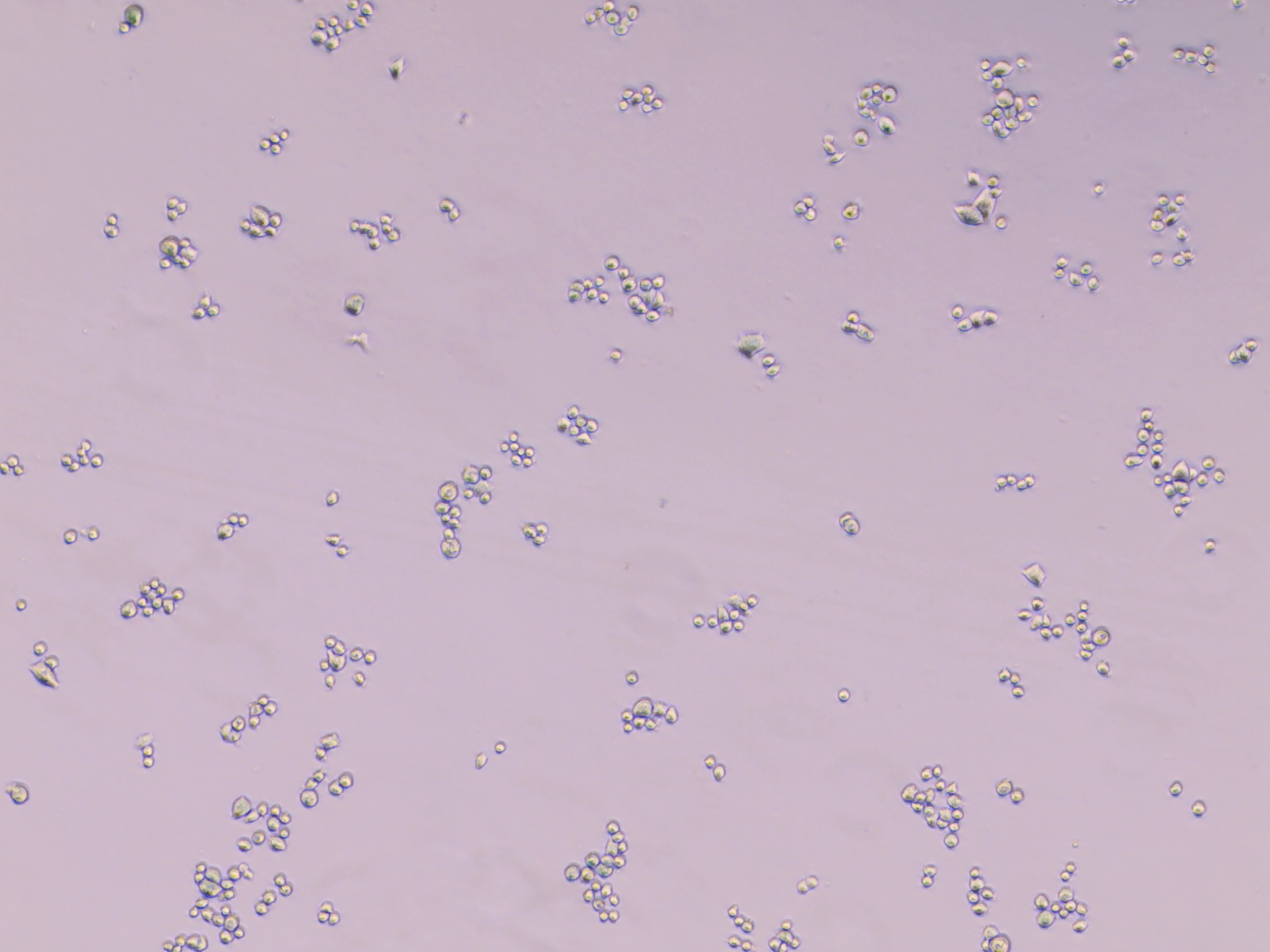

Supplement: Supplementary file 3 — Source data Fig. 2 [file 44318_2025_371_MOESM3_ESM.zip › SourceData_Figure 2/2J/ZR-75-30/n=2/zr75-30 ctrl 40225.jpg]
