# Supplementary figures and images for: Pharmacological CDK4/6 inhibition promotes vulnerability to lysosomotropic agents in breast cancer (part 2 of 2)
Source: EMBO J. 2025 Feb 10;44(7):1921–42. doi: 10.1038/s44318-025-00371-x (PMC11961731; doi:10.1038/s44318-025-00371-x)

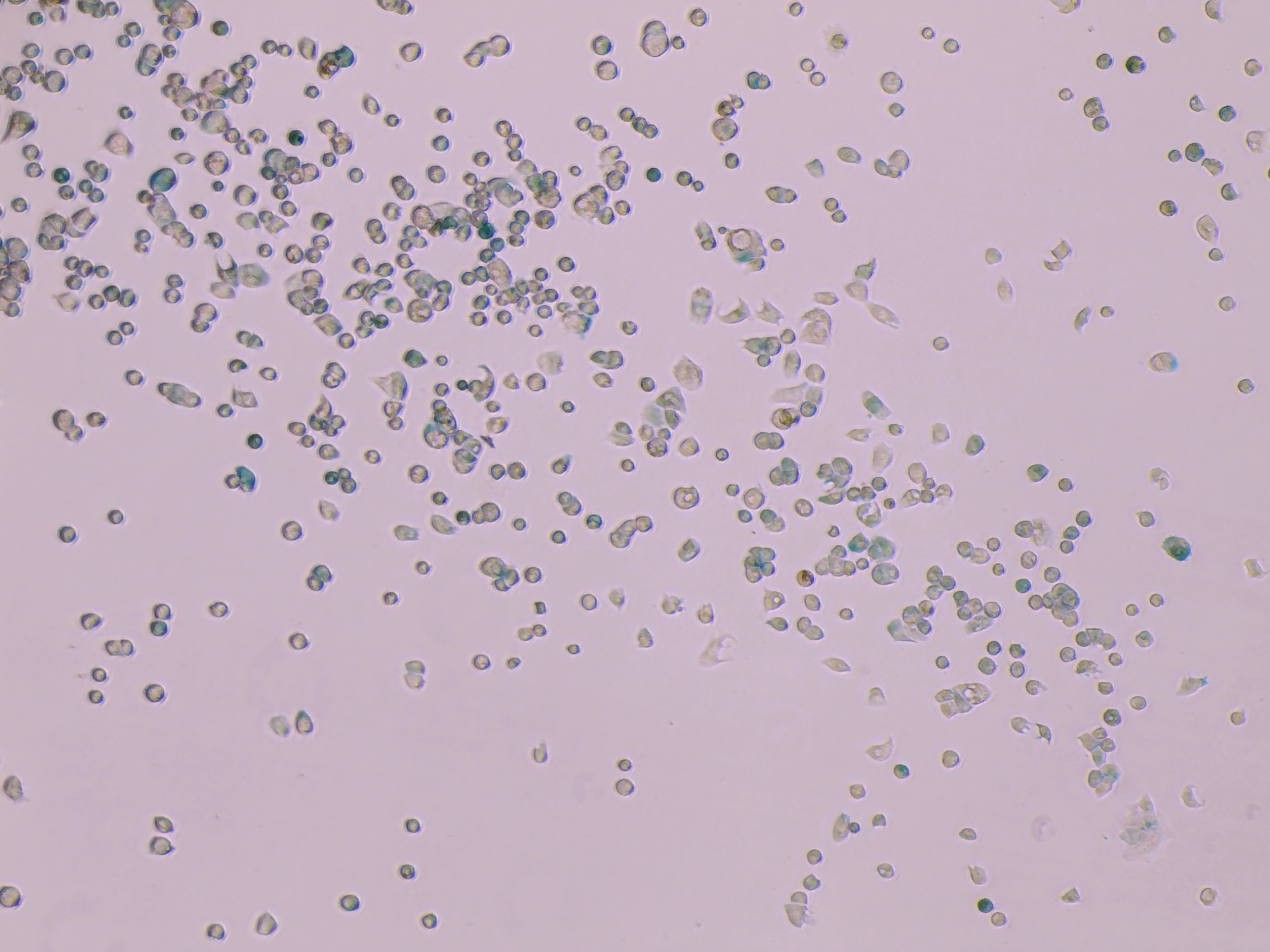

Supplement: Supplementary file 3 — Source data Fig. 2 [file 44318_2025_371_MOESM3_ESM.zip › SourceData_Figure 2/2J/ZR-75-30/n=2/zr75-30 abema 30219.jpg]

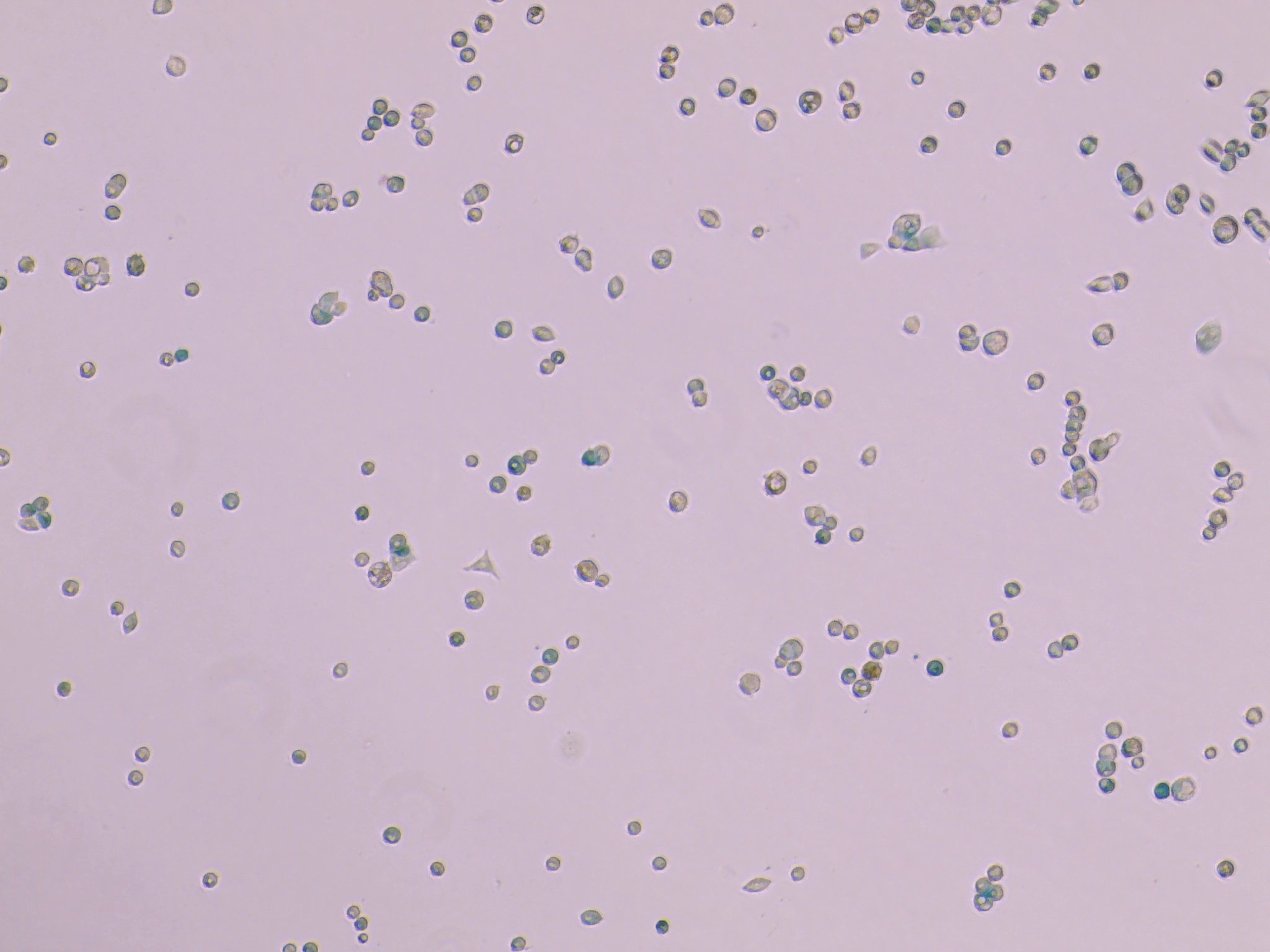

Supplement: Supplementary file 3 — Source data Fig. 2 [file 44318_2025_371_MOESM3_ESM.zip › SourceData_Figure 2/2J/ZR-75-30/n=2/zr75-30 abema 20218.jpg]

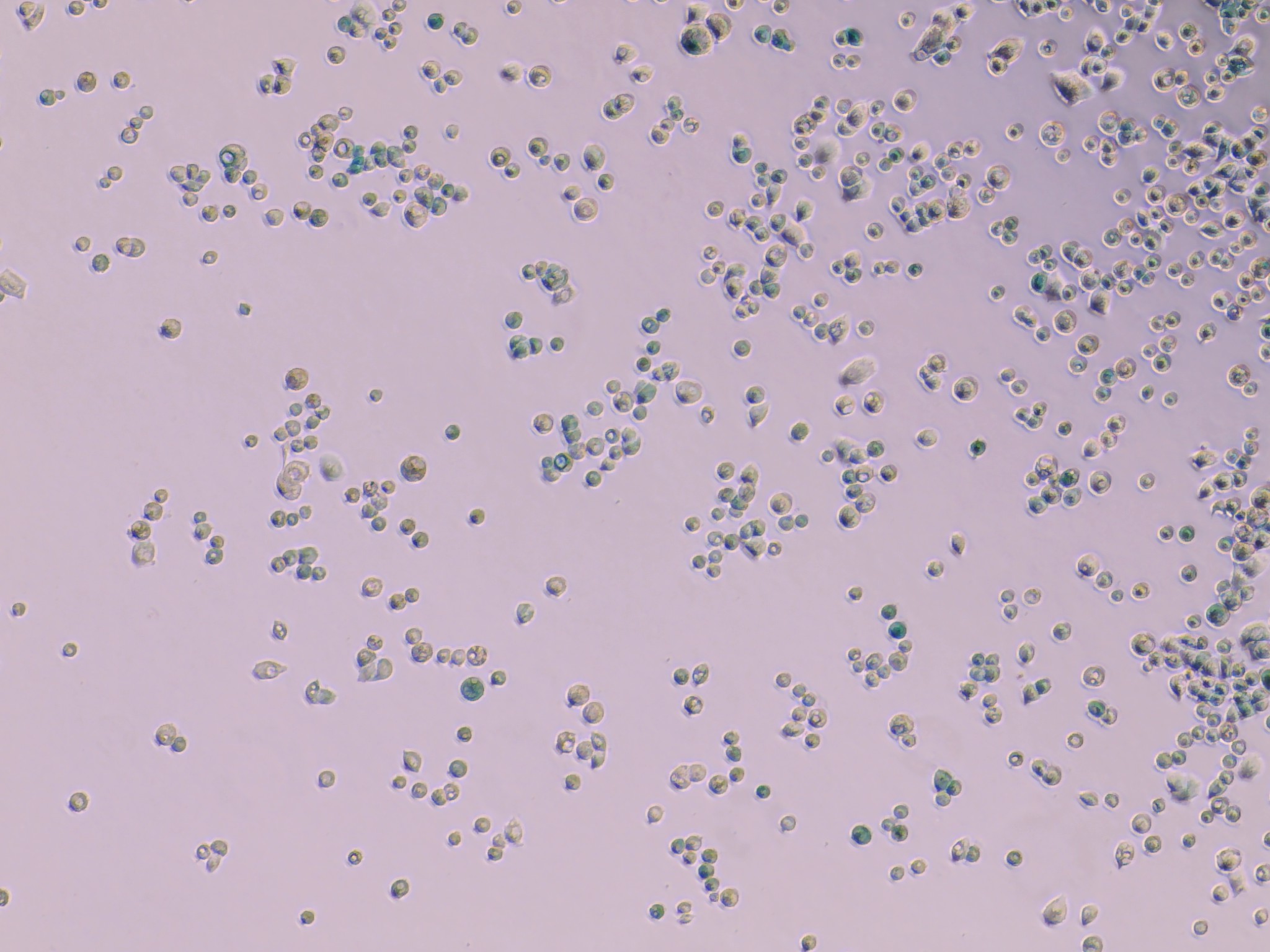

Supplement: Supplementary file 3 — Source data Fig. 2 [file 44318_2025_371_MOESM3_ESM.zip › SourceData_Figure 2/2J/ZR-75-30/n=2/zr75-30 abema 50221.jpg]

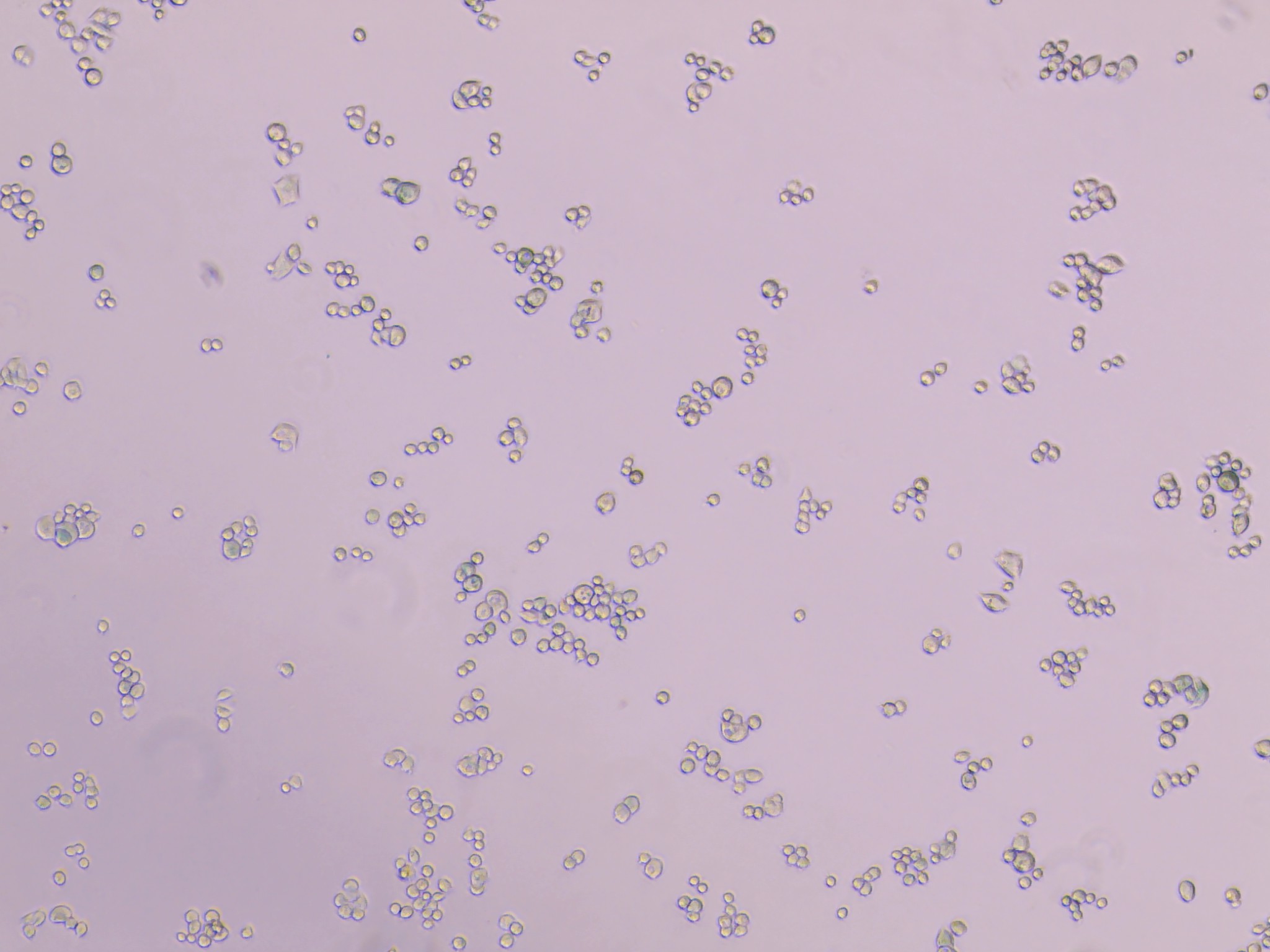

Supplement: Supplementary file 3 — Source data Fig. 2 [file 44318_2025_371_MOESM3_ESM.zip › SourceData_Figure 2/2J/ZR-75-30/n=2/zr75-30 ctrl 20223.jpg]

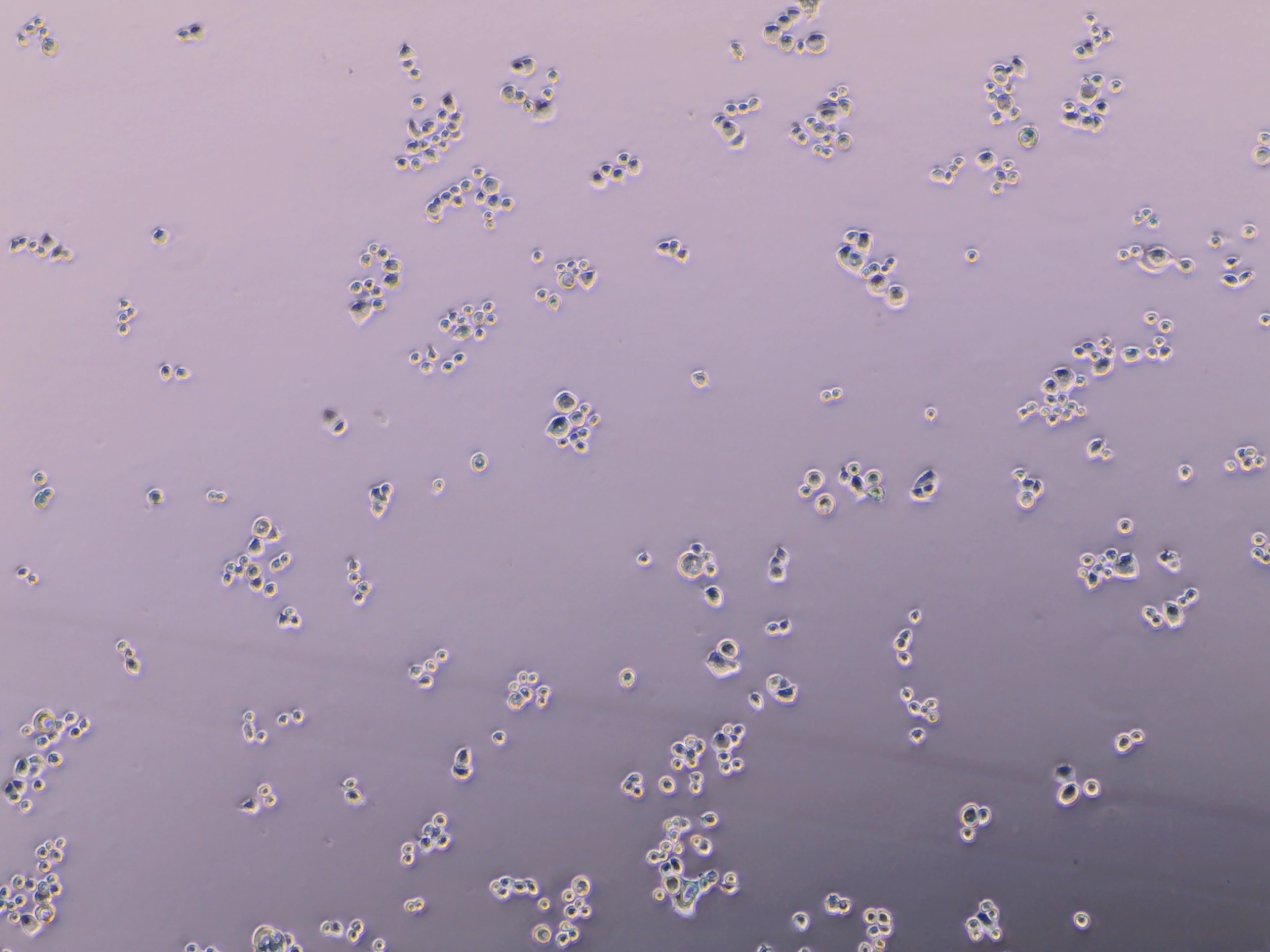

Supplement: Supplementary file 3 — Source data Fig. 2 [file 44318_2025_371_MOESM3_ESM.zip › SourceData_Figure 2/2J/ZR-75-30/n=2/zr75-30 ctrl 30224.jpg]

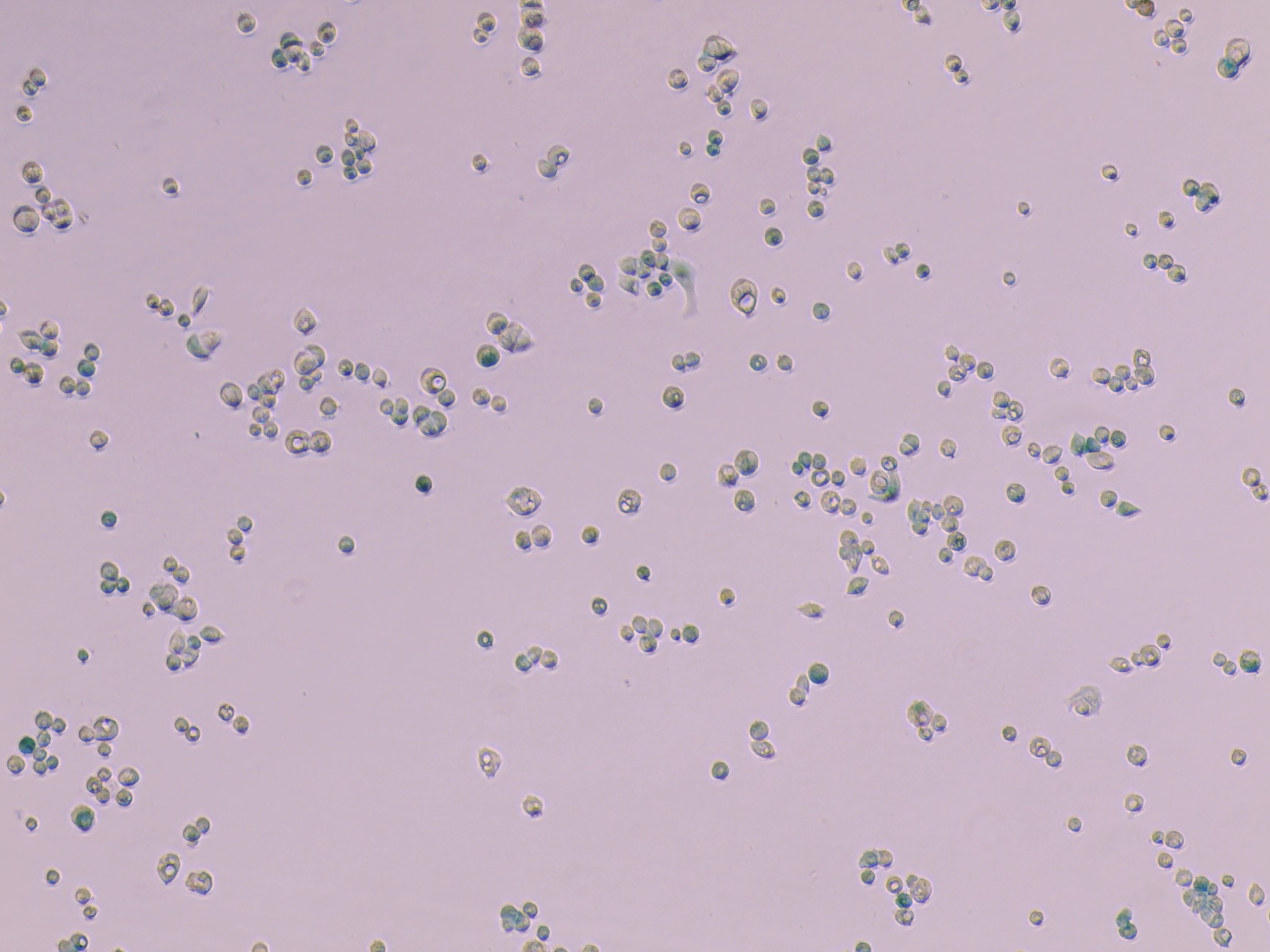

Supplement: Supplementary file 3 — Source data Fig. 2 [file 44318_2025_371_MOESM3_ESM.zip › SourceData_Figure 2/2J/ZR-75-30/n=2/zr75-30 abema 10217.jpg]

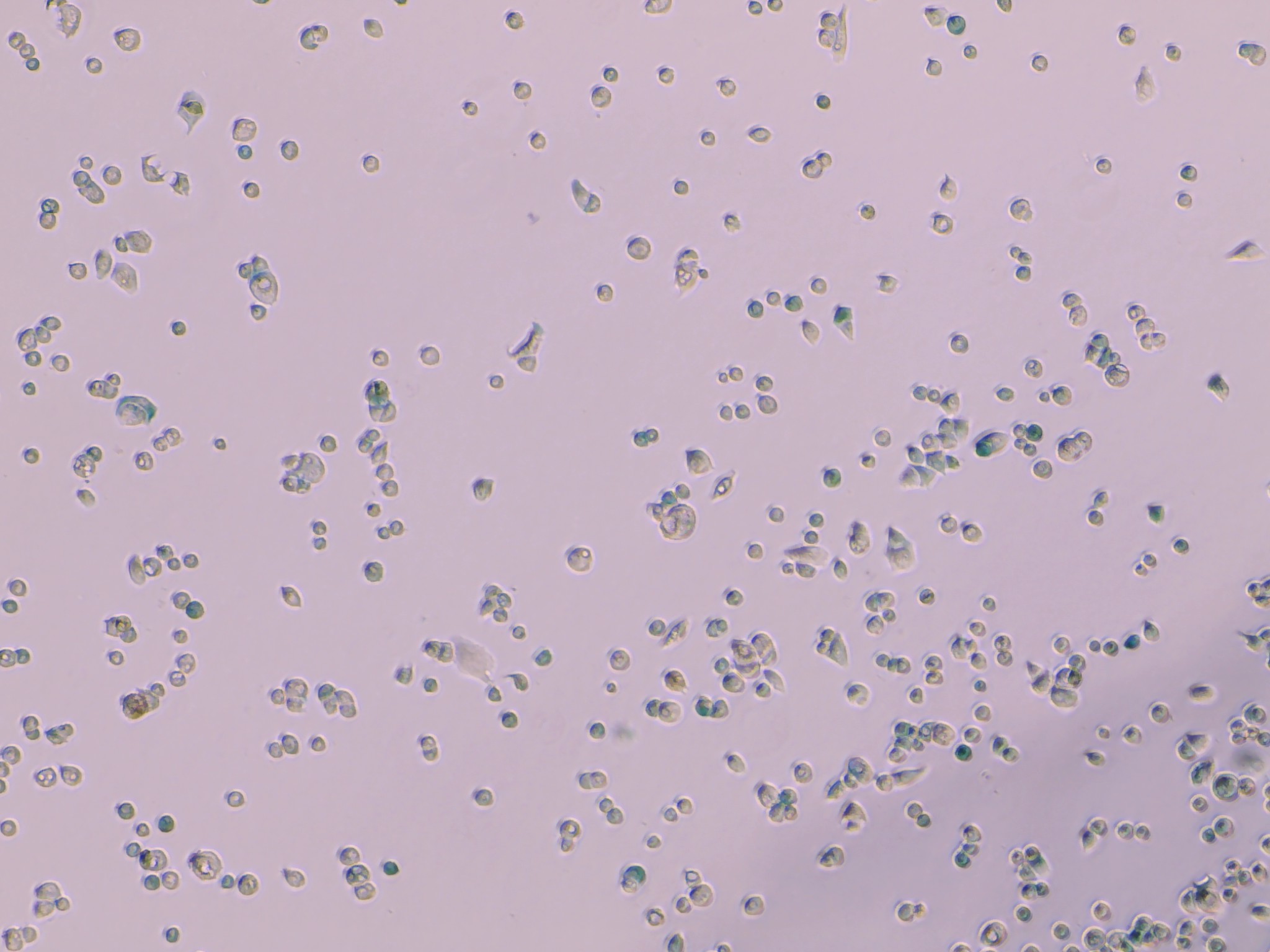

Supplement: Supplementary file 3 — Source data Fig. 2 [file 44318_2025_371_MOESM3_ESM.zip › SourceData_Figure 2/2J/ZR-75-30/n=2/zr75-30 abema 40220.jpg]

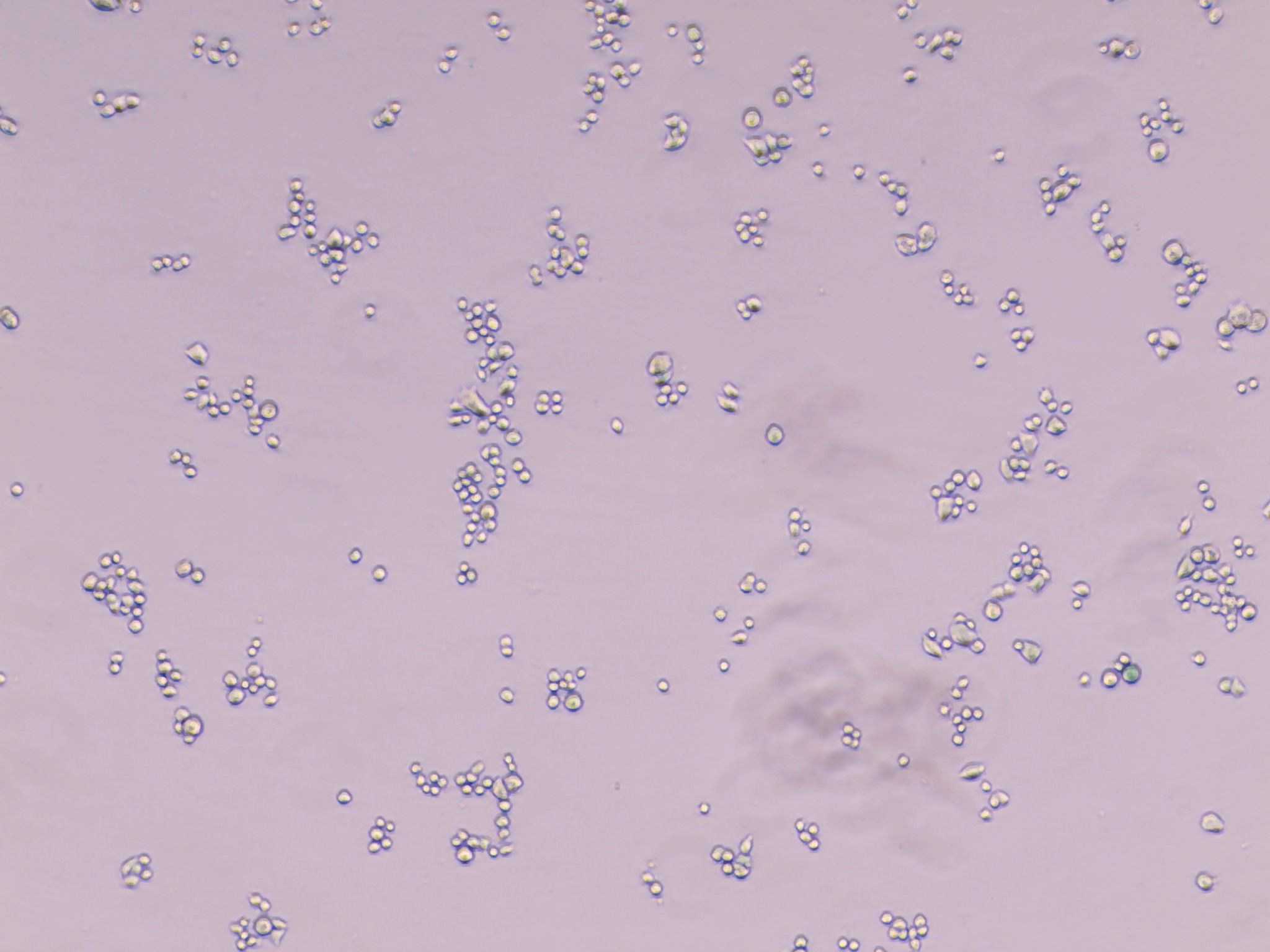

Supplement: Supplementary file 3 — Source data Fig. 2 [file 44318_2025_371_MOESM3_ESM.zip › SourceData_Figure 2/2J/ZR-75-30/n=2/zr75-30 ctrl 10222.jpg]

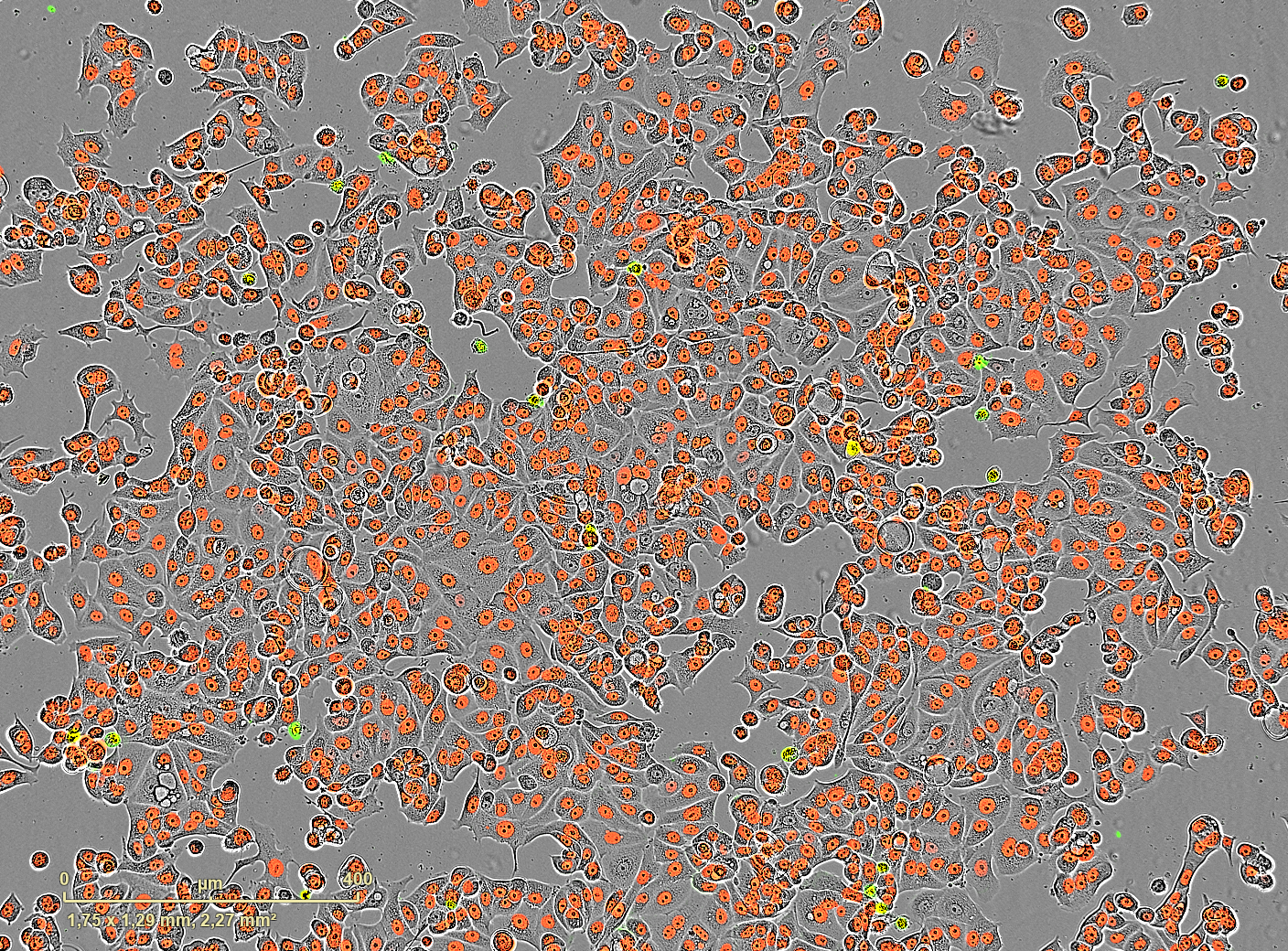

Supplement: Supplementary file 4 — Source data Fig. 3 [file 44318_2025_371_MOESM4_ESM.zip › SourceData_Figure 3/3B/mcf7 abema LLOme 1mM time 0_H1_2_00d00h00m.tif]

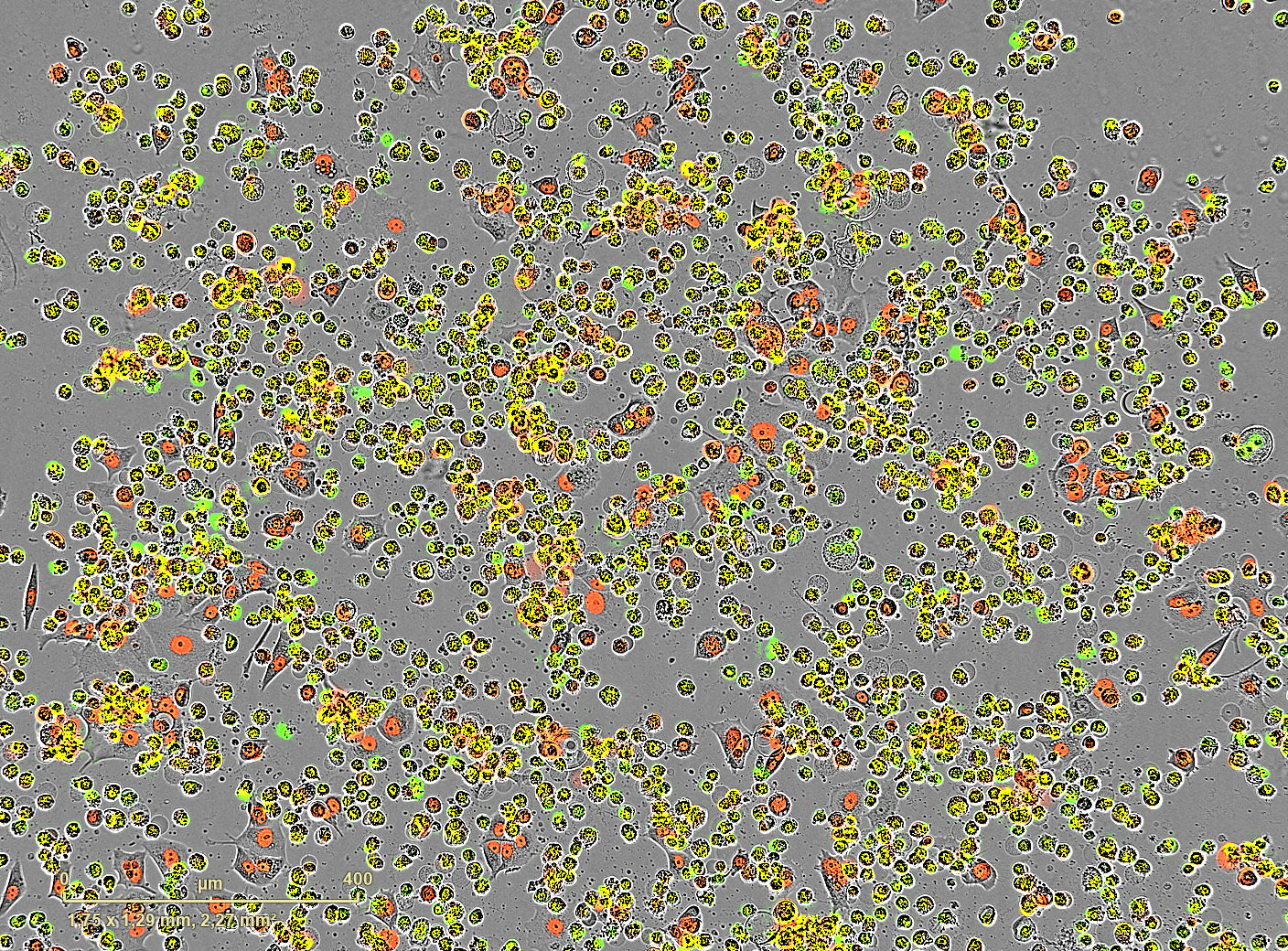

Supplement: Supplementary file 4 — Source data Fig. 3 [file 44318_2025_371_MOESM4_ESM.zip › SourceData_Figure 3/3B/mcg7 abema llome 1mM 3 days_H1_2_00d00h00m.tif]

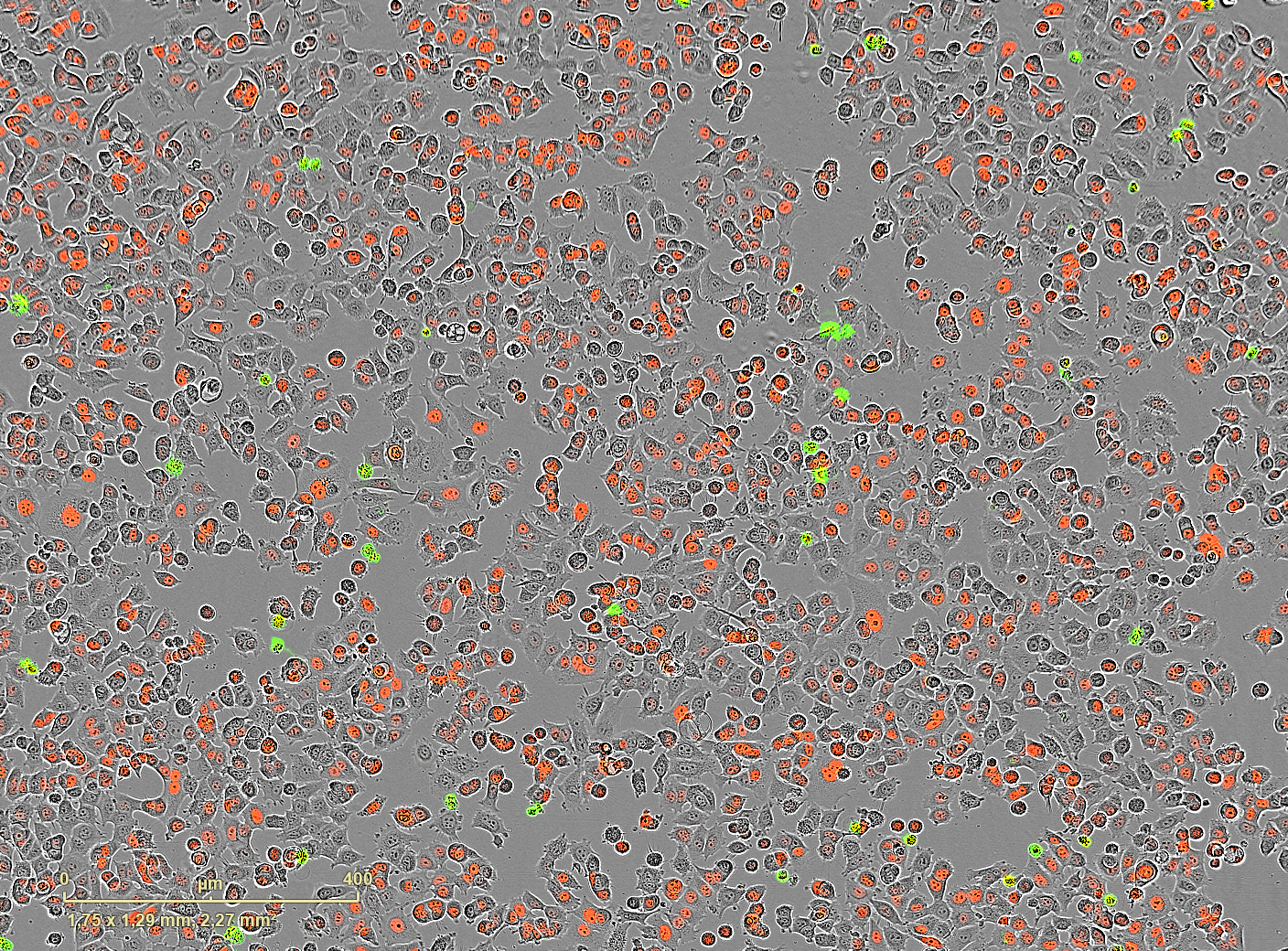

Supplement: Supplementary file 4 — Source data Fig. 3 [file 44318_2025_371_MOESM4_ESM.zip › SourceData_Figure 3/3B/mcf7 dmso llome 1mM time 0_H9_1_00d00h00m.tif]

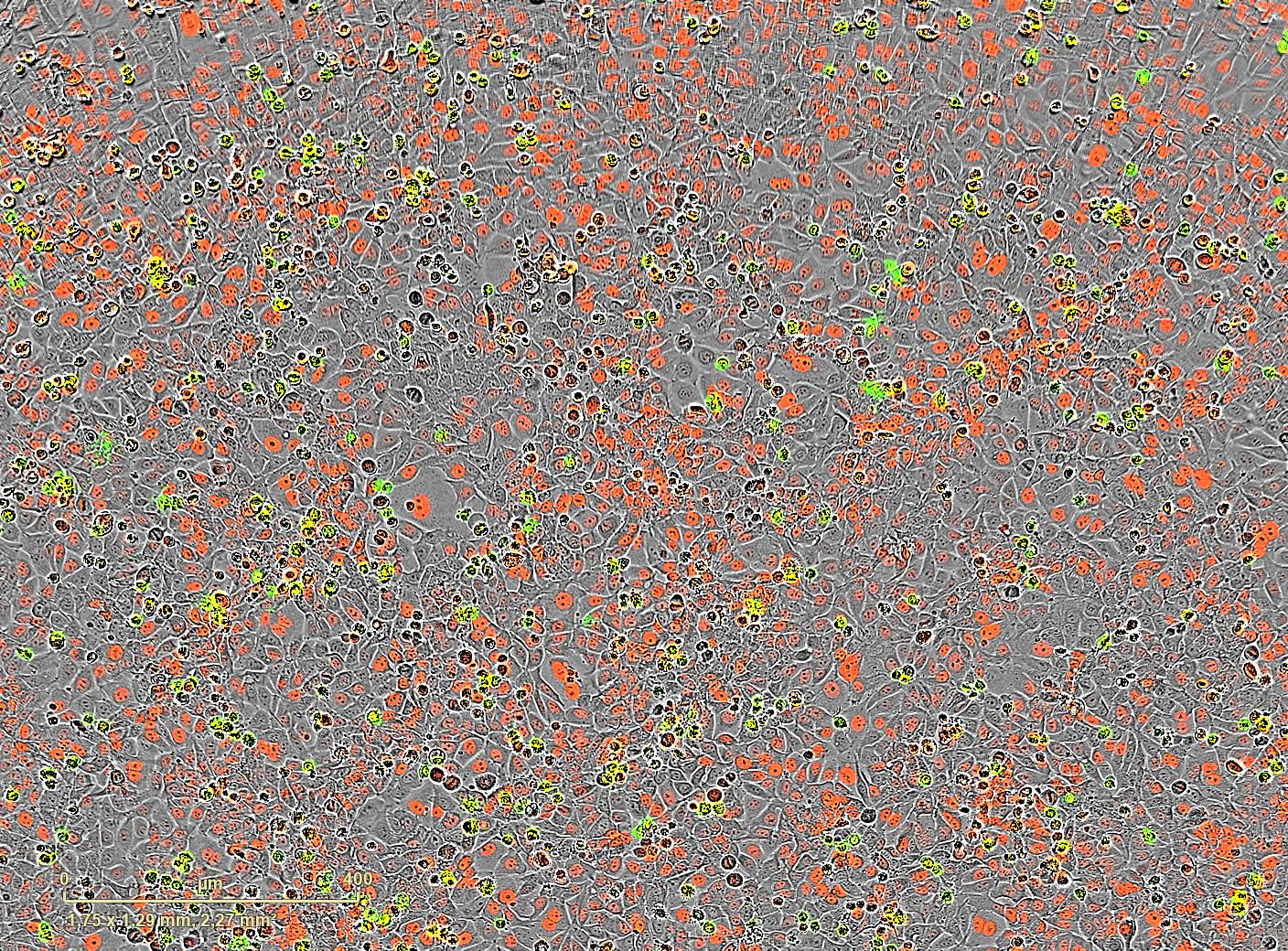

Supplement: Supplementary file 4 — Source data Fig. 3 [file 44318_2025_371_MOESM4_ESM.zip › SourceData_Figure 3/3B/mcf7 dmso llome 1mM 3 days_H9_1_00d00h00m.tif]

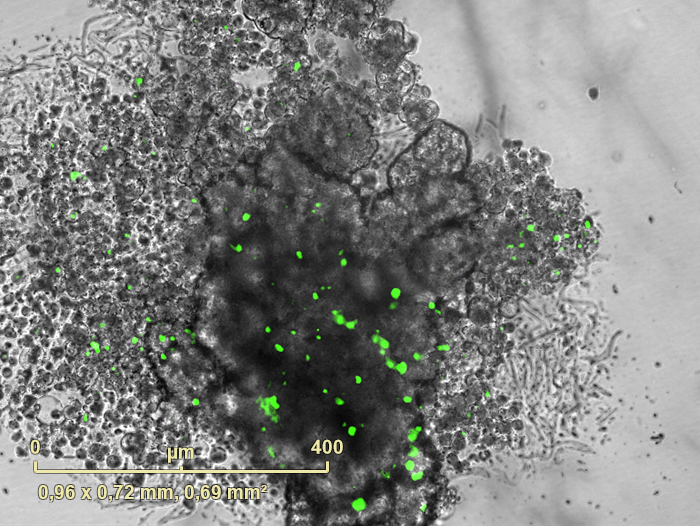

Supplement: Supplementary file 4 — Source data Fig. 3 [file 44318_2025_371_MOESM4_ESM.zip › SourceData_Figure 3/3J/MCF7_Abema_H3_1_2024y04m07d_13h13m.tif]

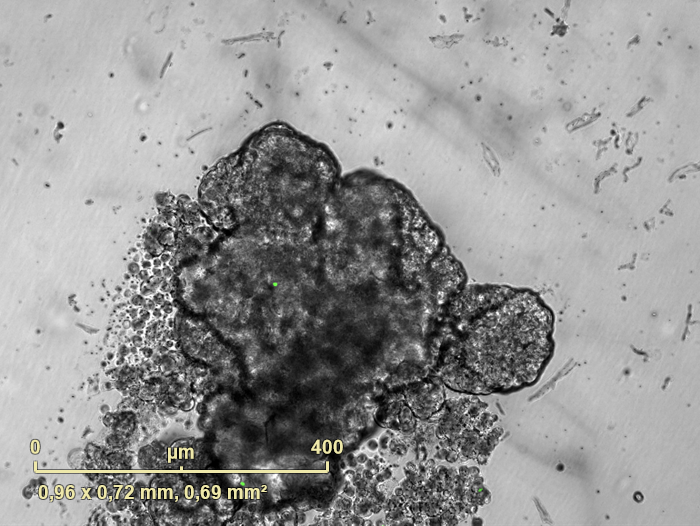

Supplement: Supplementary file 4 — Source data Fig. 3 [file 44318_2025_371_MOESM4_ESM.zip › SourceData_Figure 3/3J/MCF7_Abema_H4_1_2024y04m06d_13h13m.tif]

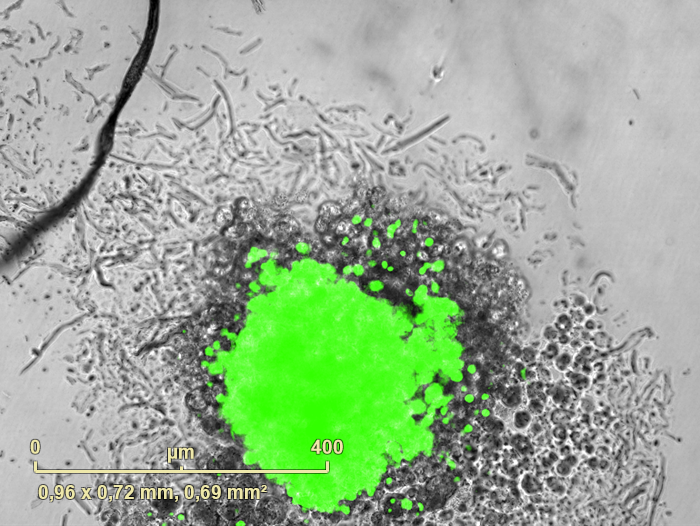

Supplement: Supplementary file 4 — Source data Fig. 3 [file 44318_2025_371_MOESM4_ESM.zip › SourceData_Figure 3/3J/MCF7_Abema_H6_1_2024y04m08d_13h13m.tif]

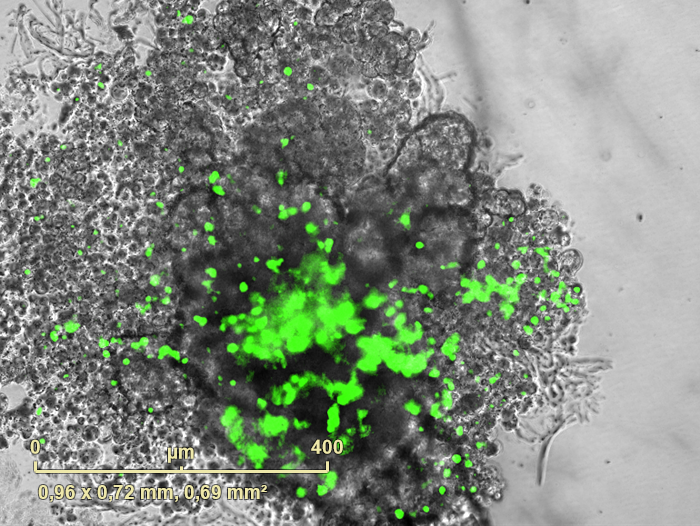

Supplement: Supplementary file 4 — Source data Fig. 3 [file 44318_2025_371_MOESM4_ESM.zip › SourceData_Figure 3/3J/MCF7_Abema_H3_1_2024y04m08d_13h13m.tif]

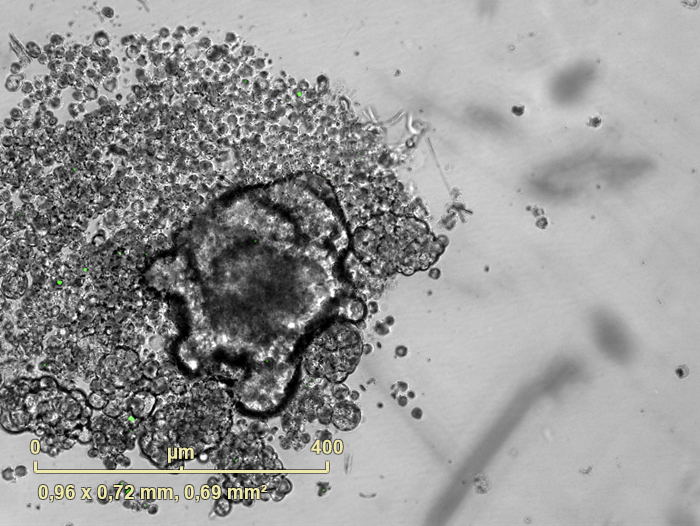

Supplement: Supplementary file 4 — Source data Fig. 3 [file 44318_2025_371_MOESM4_ESM.zip › SourceData_Figure 3/3J/MCF7_Abema_H1_1_2024y04m06d_13h13m.tif]

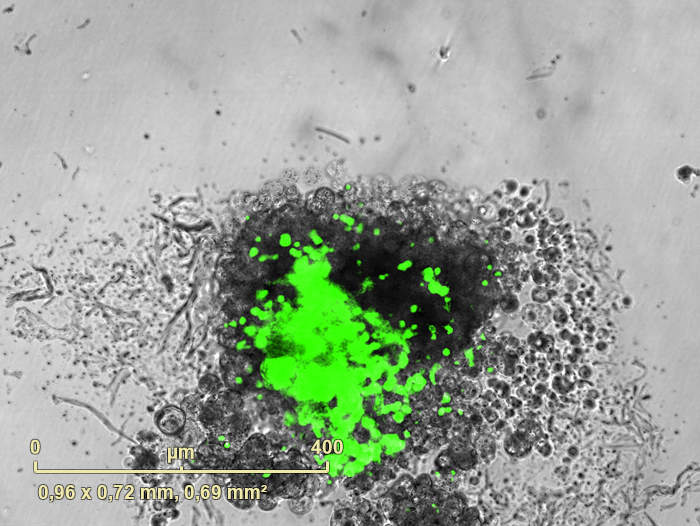

Supplement: Supplementary file 4 — Source data Fig. 3 [file 44318_2025_371_MOESM4_ESM.zip › SourceData_Figure 3/3J/MCF7_Abema_H6_1_2024y04m07d_13h13m.tif]

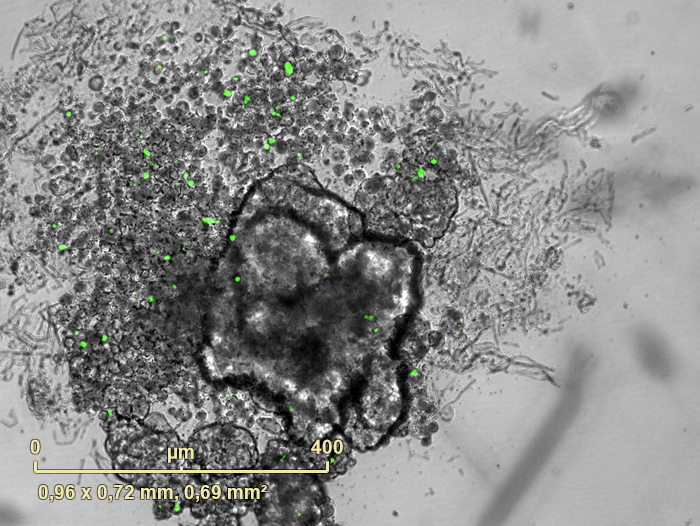

Supplement: Supplementary file 4 — Source data Fig. 3 [file 44318_2025_371_MOESM4_ESM.zip › SourceData_Figure 3/3J/MCF7_Abema_H1_1_2024y04m08d_13h13m.tif]

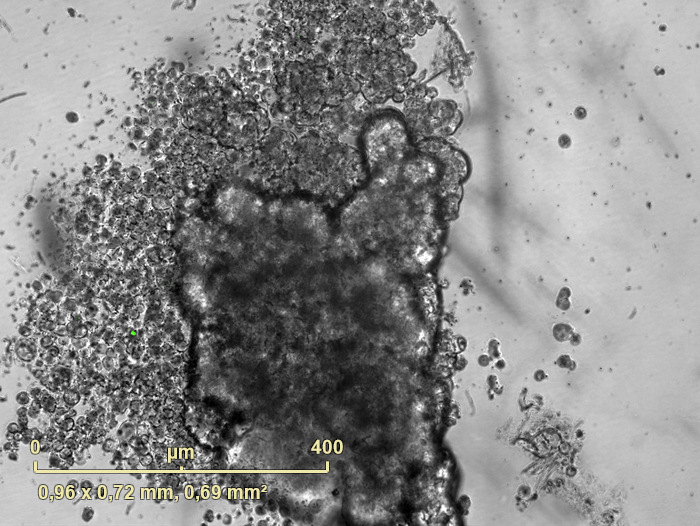

Supplement: Supplementary file 4 — Source data Fig. 3 [file 44318_2025_371_MOESM4_ESM.zip › SourceData_Figure 3/3J/MCF7_Abema_H3_1_2024y04m06d_13h13m.tif]

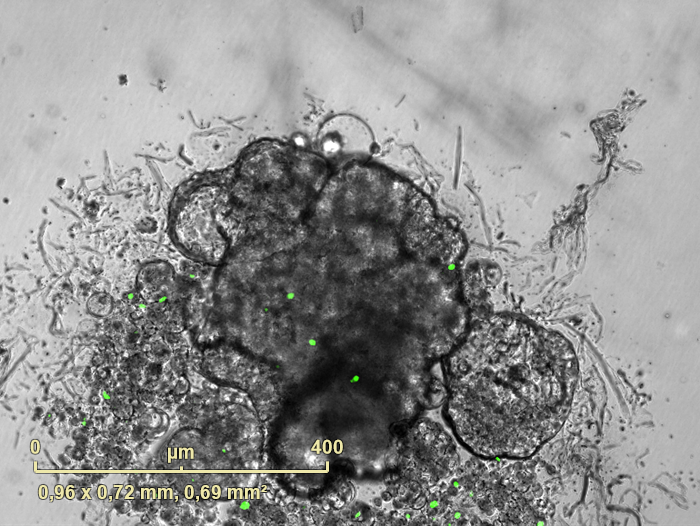

Supplement: Supplementary file 4 — Source data Fig. 3 [file 44318_2025_371_MOESM4_ESM.zip › SourceData_Figure 3/3J/MCF7_Abema_H4_1_2024y04m07d_13h13m.tif]

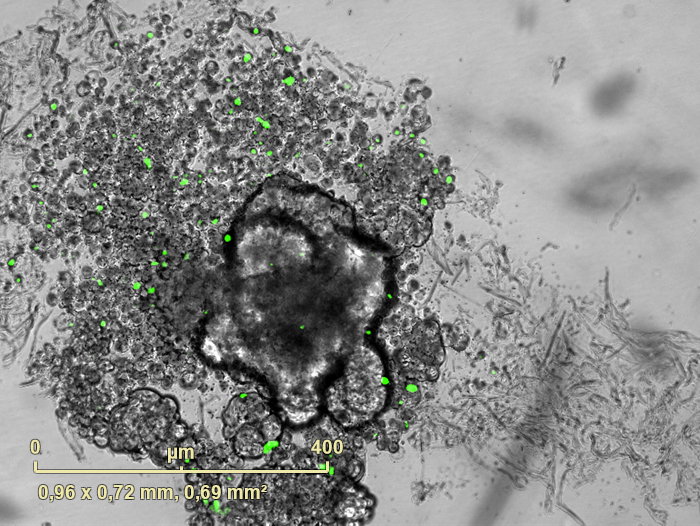

Supplement: Supplementary file 4 — Source data Fig. 3 [file 44318_2025_371_MOESM4_ESM.zip › SourceData_Figure 3/3J/MCF7_Abema_H1_1_2024y04m07d_13h13m.tif]

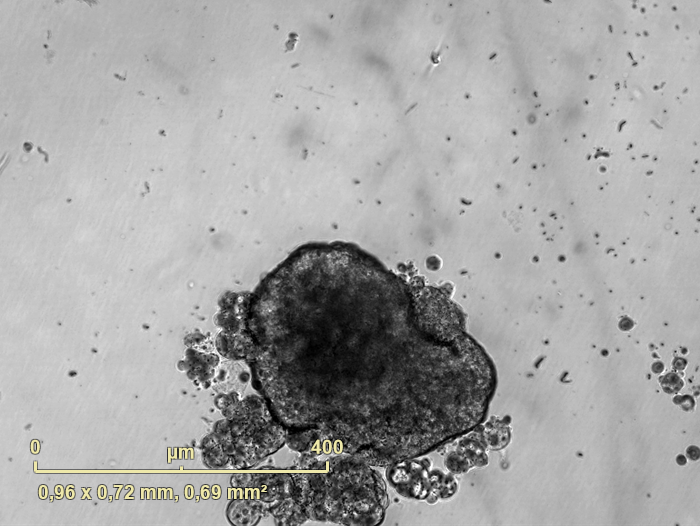

Supplement: Supplementary file 4 — Source data Fig. 3 [file 44318_2025_371_MOESM4_ESM.zip › SourceData_Figure 3/3J/MCF7_Abema_H6_1_2024y04m06d_13h13m.tif]

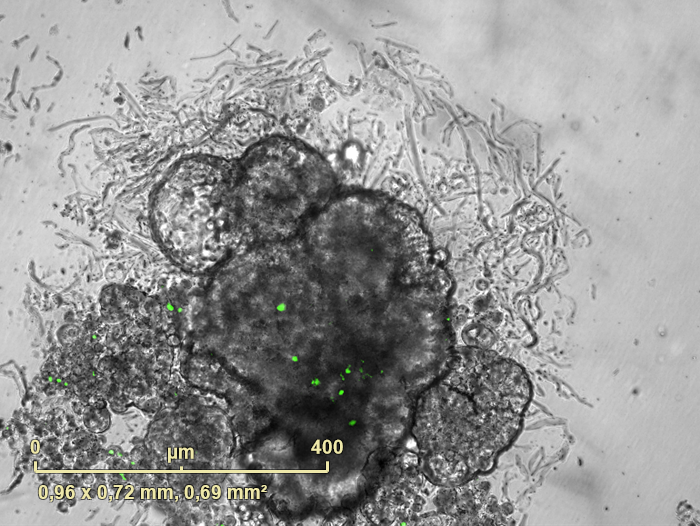

Supplement: Supplementary file 4 — Source data Fig. 3 [file 44318_2025_371_MOESM4_ESM.zip › SourceData_Figure 3/3J/MCF7_Abema_H4_1_2024y04m08d_13h13m.tif]

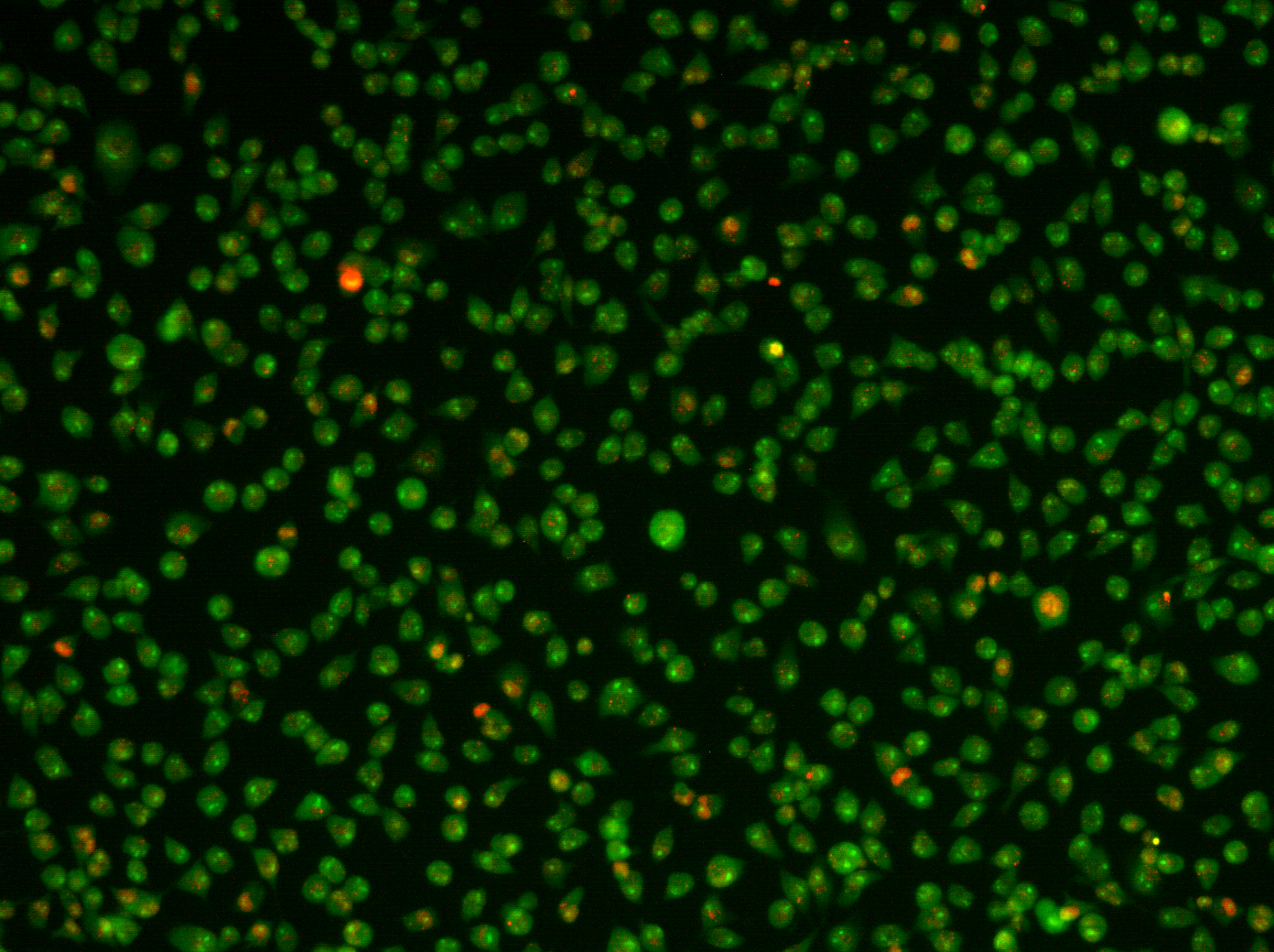

Supplement: Supplementary file 5 — Source data Fig. 4 [file 44318_2025_371_MOESM5_ESM.zip › SourceData_Figure 4/4C/MDA231 Experiment.lif_Overlay007.tif]

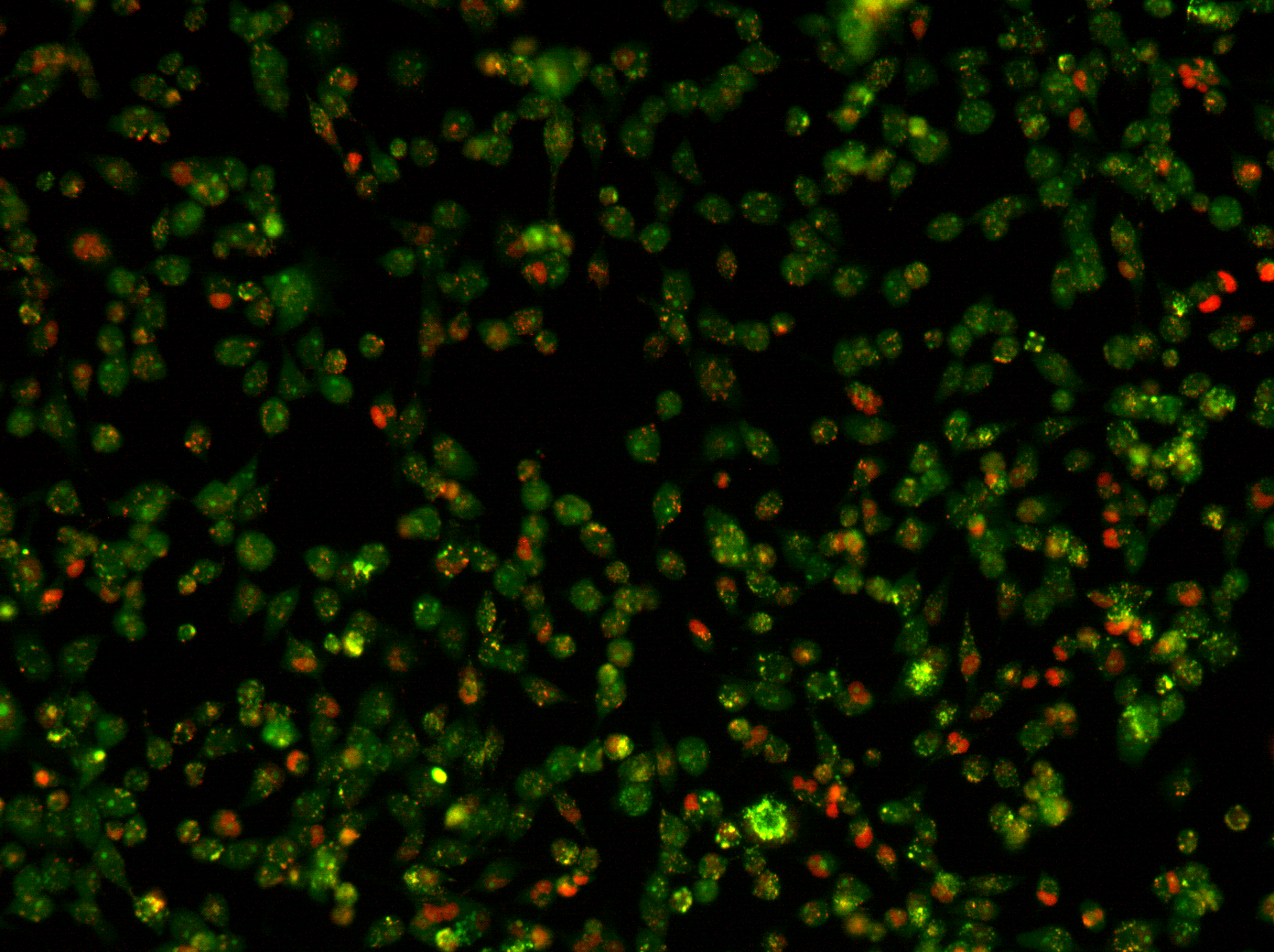

Supplement: Supplementary file 5 — Source data Fig. 4 [file 44318_2025_371_MOESM5_ESM.zip › SourceData_Figure 4/4C/abema mda231_Overlay006.tif]

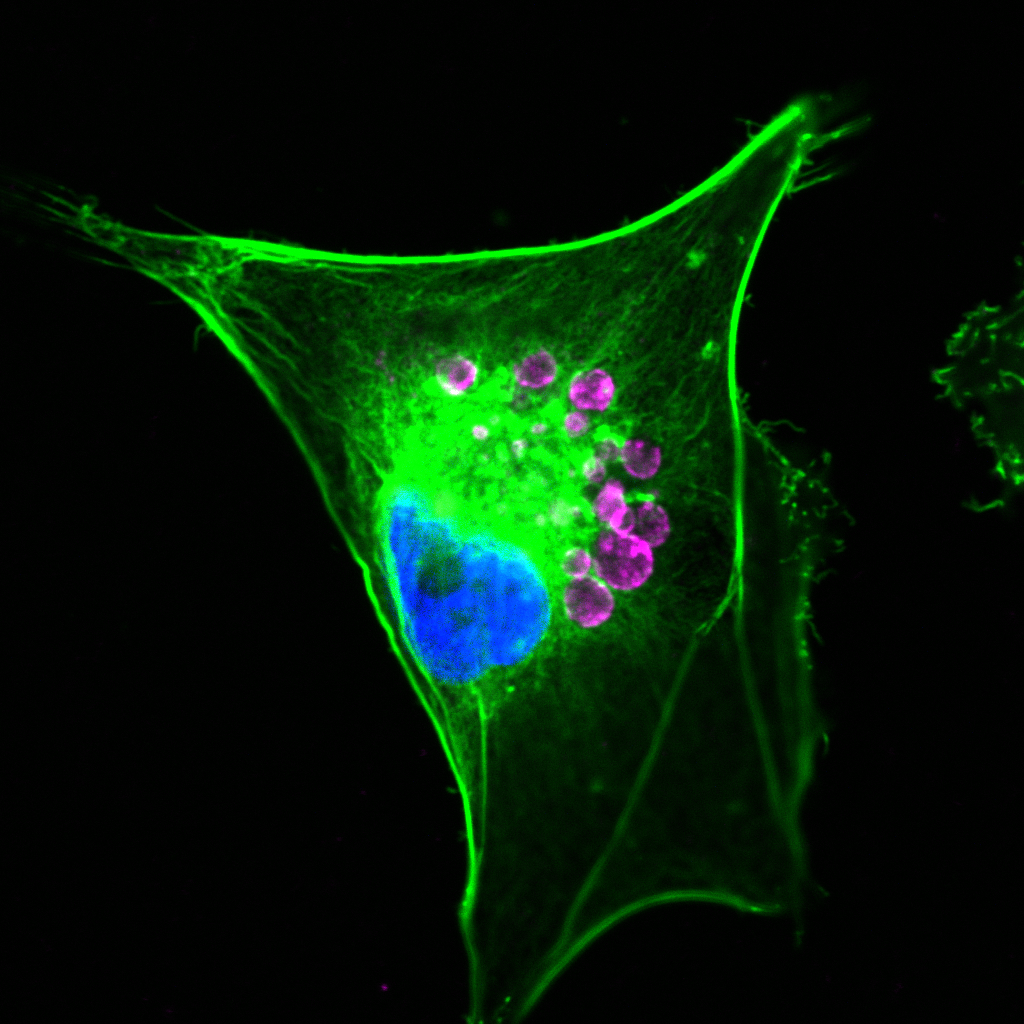

Supplement: Supplementary file 5 — Source data Fig. 4 [file 44318_2025_371_MOESM5_ESM.zip › SourceData_Figure 4/4D/MDA-231 Abema.tif]

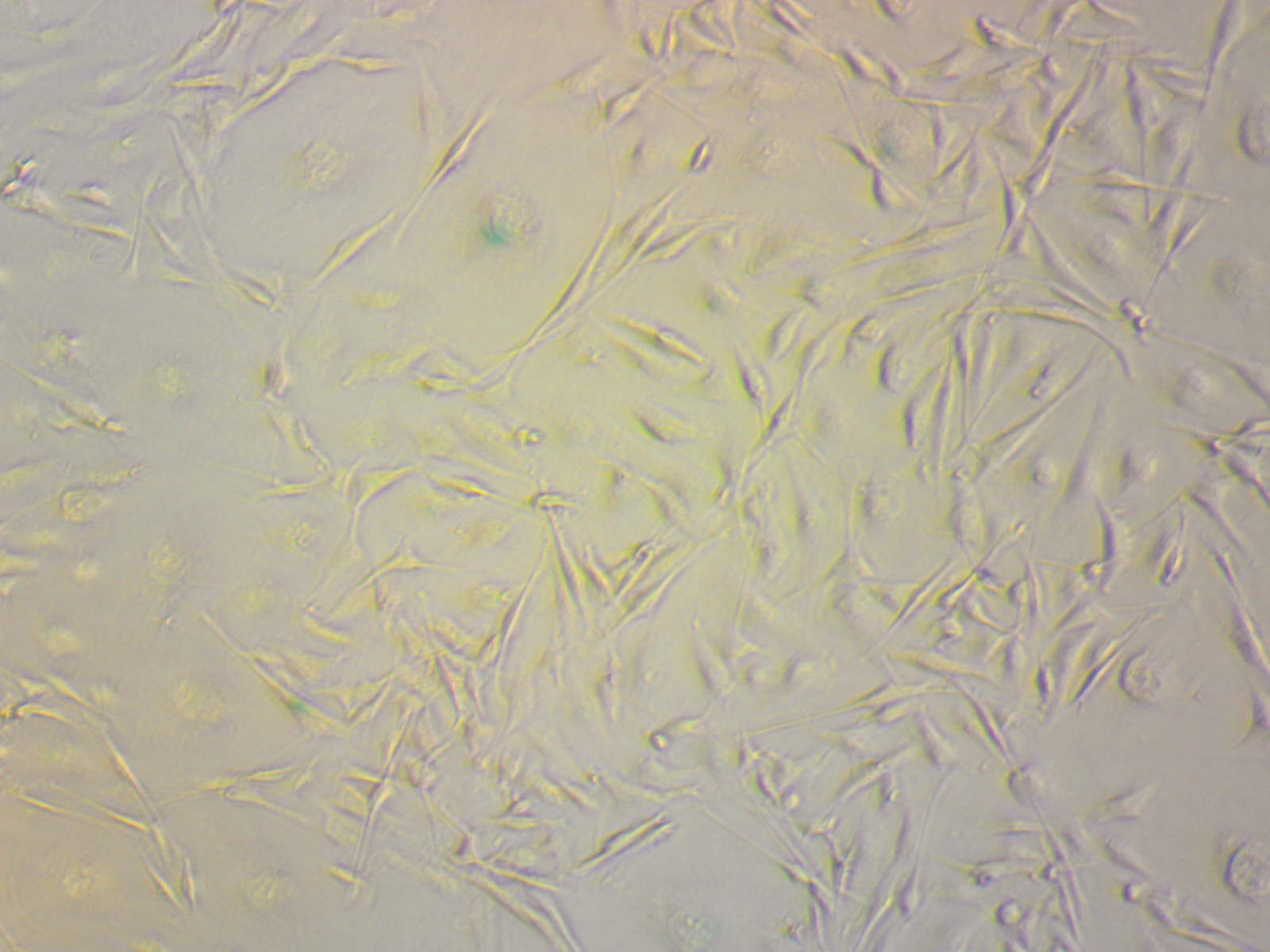

Supplement: Supplementary file 5 — Source data Fig. 4 [file 44318_2025_371_MOESM5_ESM.zip › SourceData_Figure 4/4A/MDA-231 Veh-1.jpg]

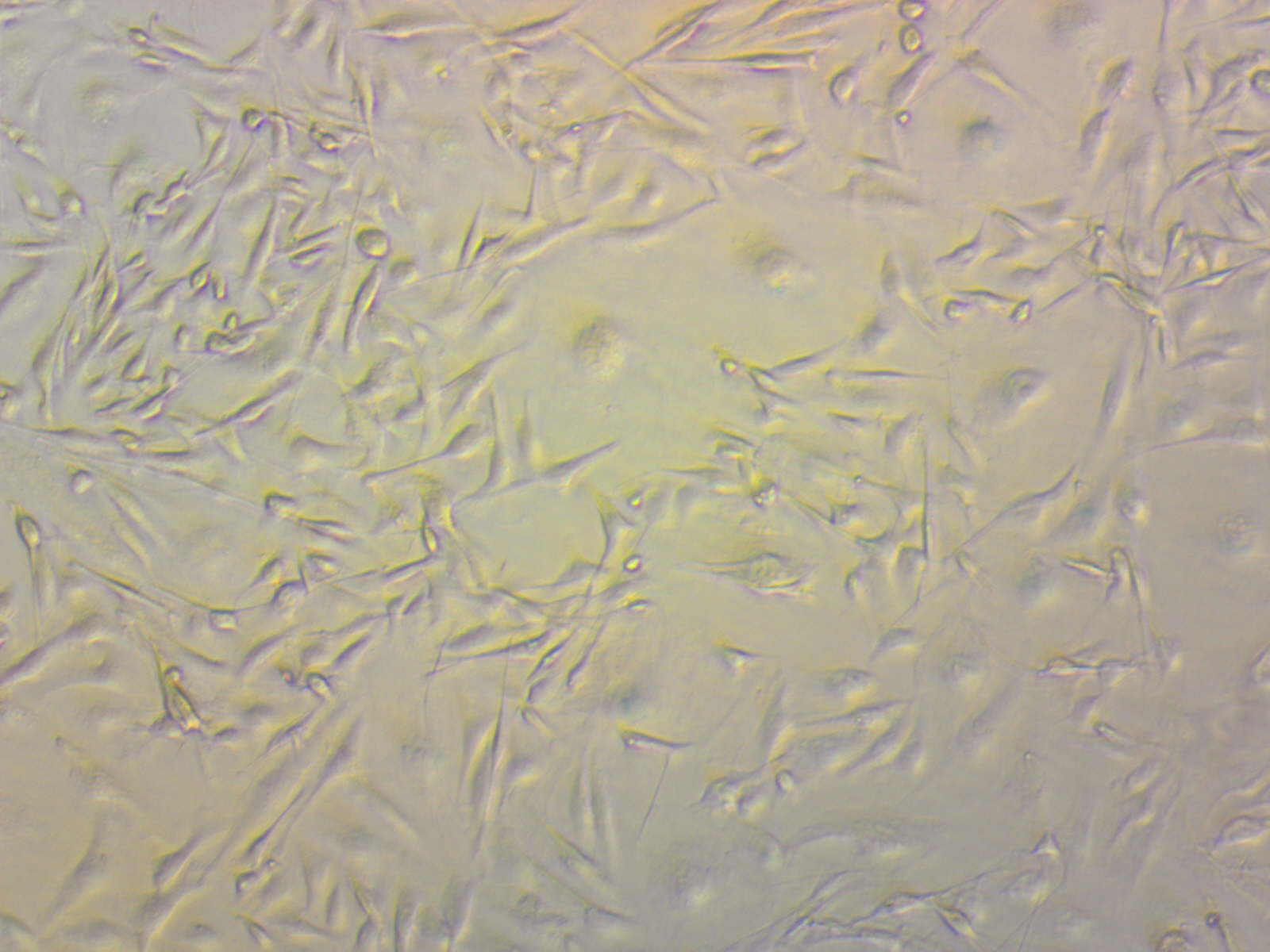

Supplement: Supplementary file 5 — Source data Fig. 4 [file 44318_2025_371_MOESM5_ESM.zip › SourceData_Figure 4/4A/MDA-231 Veh-2.jpg]

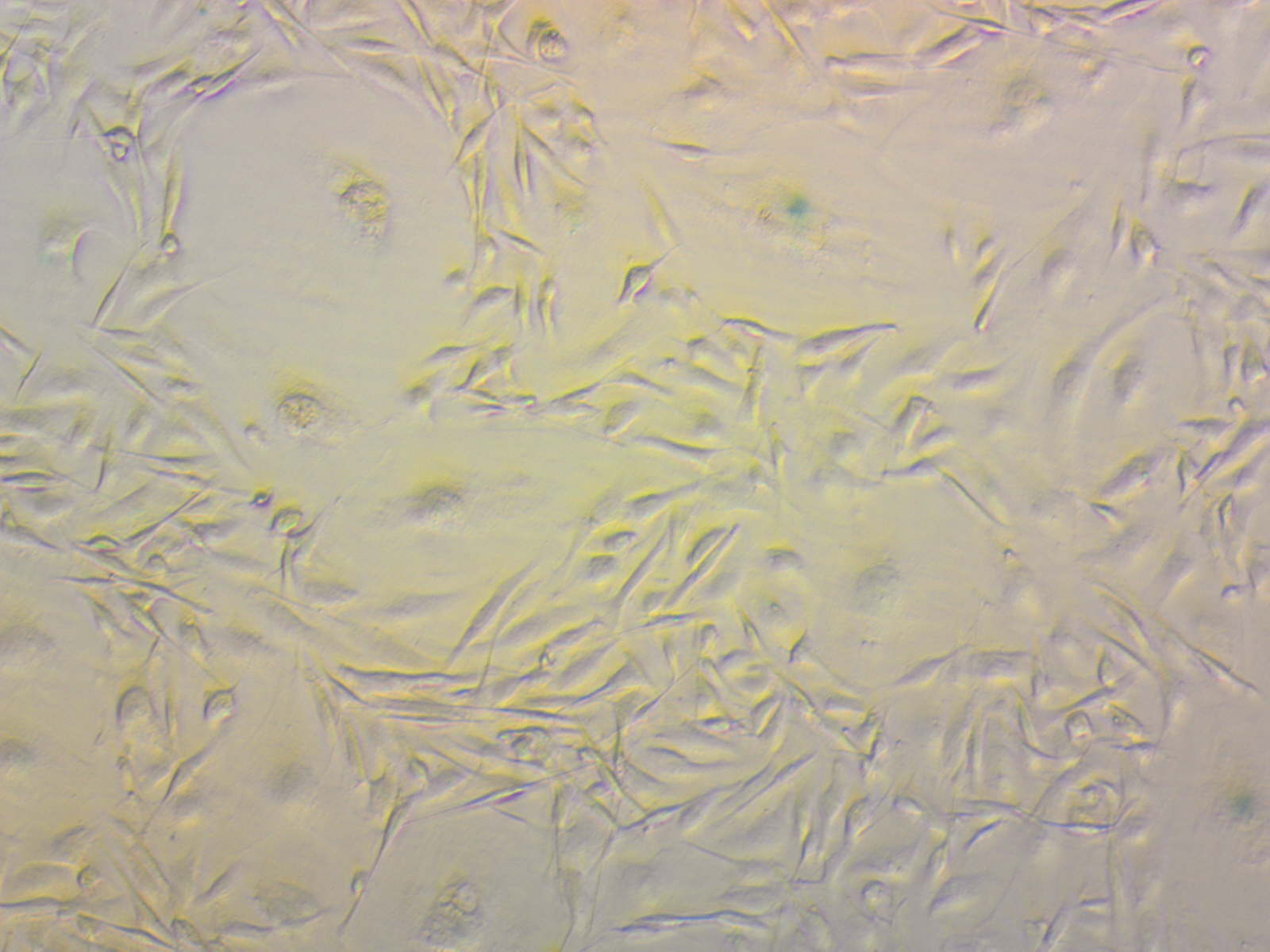

Supplement: Supplementary file 5 — Source data Fig. 4 [file 44318_2025_371_MOESM5_ESM.zip › SourceData_Figure 4/4A/MDA-231 Veh-3.jpg]

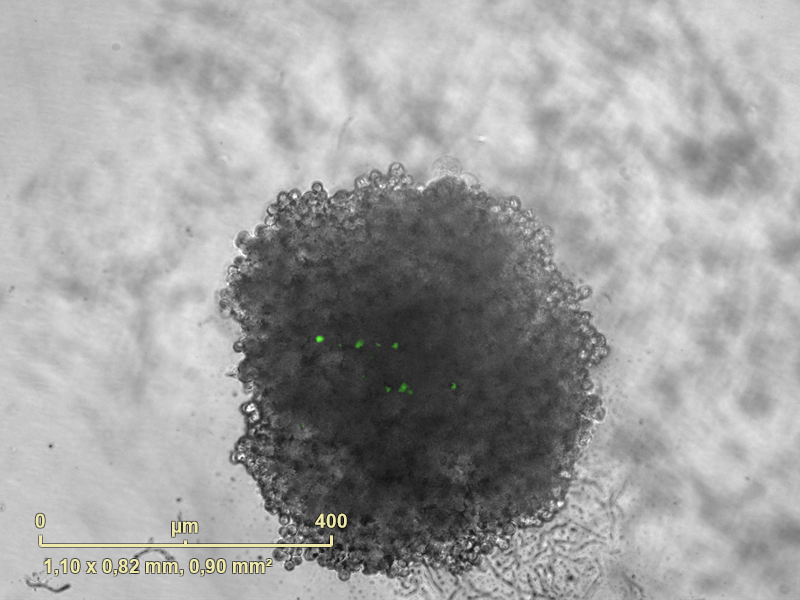

Supplement: Supplementary file 5 — Source data Fig. 4 [file 44318_2025_371_MOESM5_ESM.zip › SourceData_Figure 4/4I/MDA-231_Abema_A3_1_2024y04m07d_13h13m.tif]

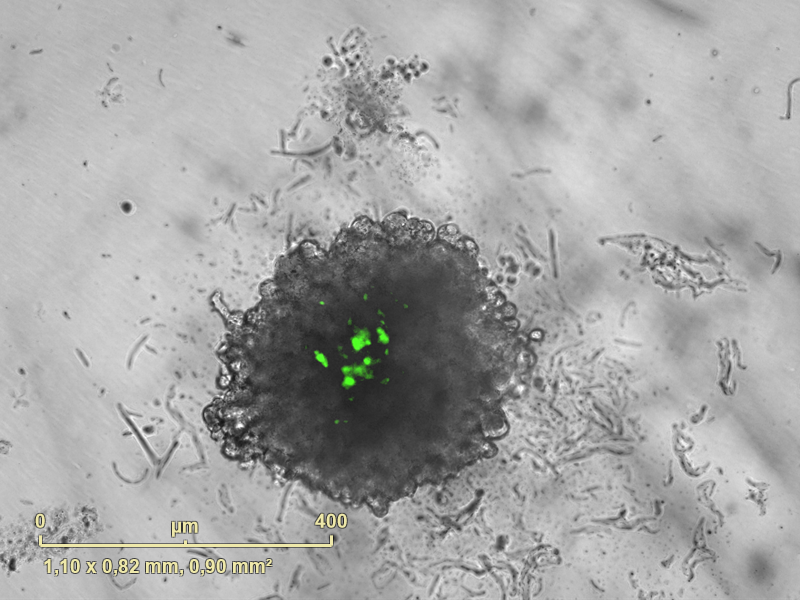

Supplement: Supplementary file 5 — Source data Fig. 4 [file 44318_2025_371_MOESM5_ESM.zip › SourceData_Figure 4/4I/MDA-231_Abema_B4_1_2024y04m07d_13h13m.tif]

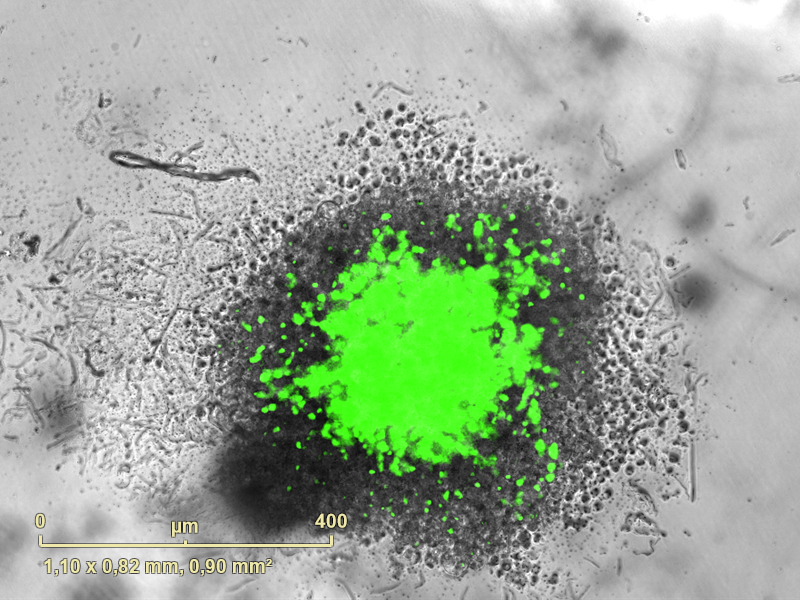

Supplement: Supplementary file 5 — Source data Fig. 4 [file 44318_2025_371_MOESM5_ESM.zip › SourceData_Figure 4/4I/MDA-231_Abema_A6_1_2024y04m08d_13h13m.tif]

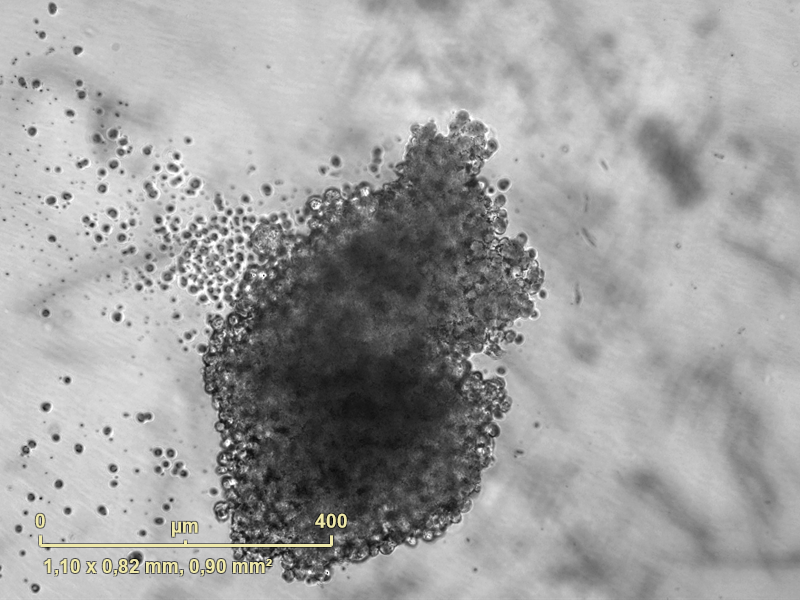

Supplement: Supplementary file 5 — Source data Fig. 4 [file 44318_2025_371_MOESM5_ESM.zip › SourceData_Figure 4/4I/MDA-231_Abema_C1_1_2024y04m06d_13h13m.tif]

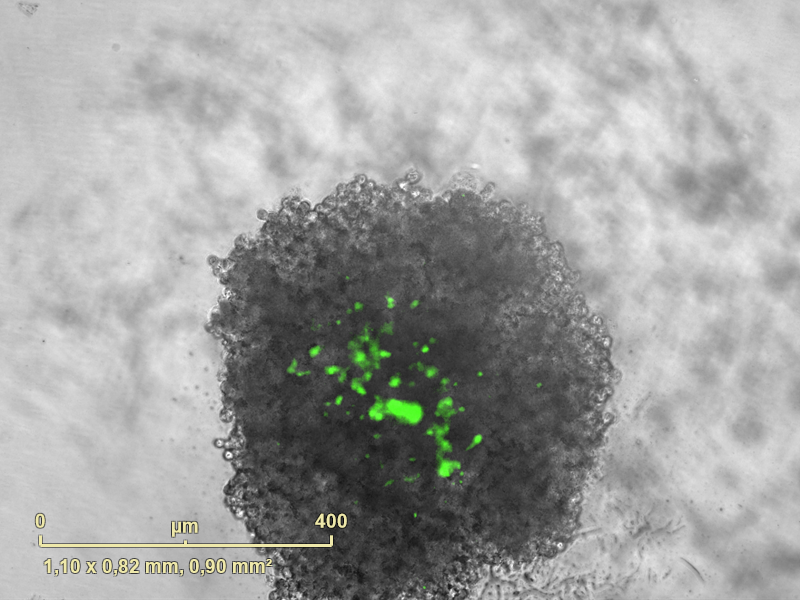

Supplement: Supplementary file 5 — Source data Fig. 4 [file 44318_2025_371_MOESM5_ESM.zip › SourceData_Figure 4/4I/MDA-231_Abema_A3_1_2024y04m08d_13h13m.tif]

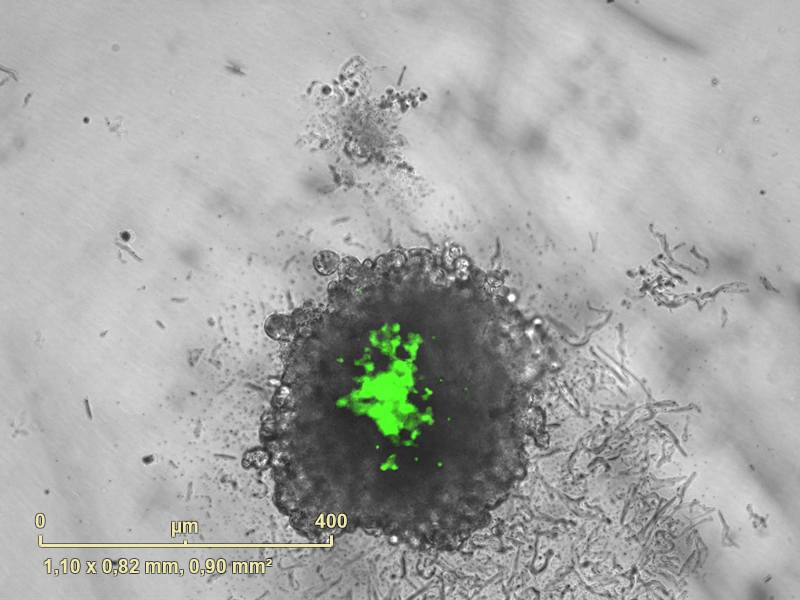

Supplement: Supplementary file 5 — Source data Fig. 4 [file 44318_2025_371_MOESM5_ESM.zip › SourceData_Figure 4/4I/MDA-231_Abema_B4_1_2024y04m08d_13h13m.tif]

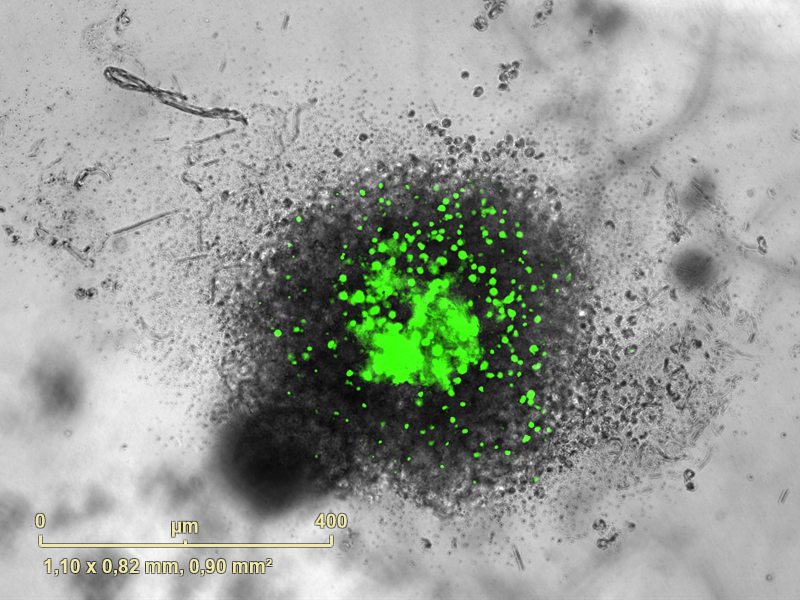

Supplement: Supplementary file 5 — Source data Fig. 4 [file 44318_2025_371_MOESM5_ESM.zip › SourceData_Figure 4/4I/MDA-231_Abema_A6_1_2024y04m07d_13h13m.tif]

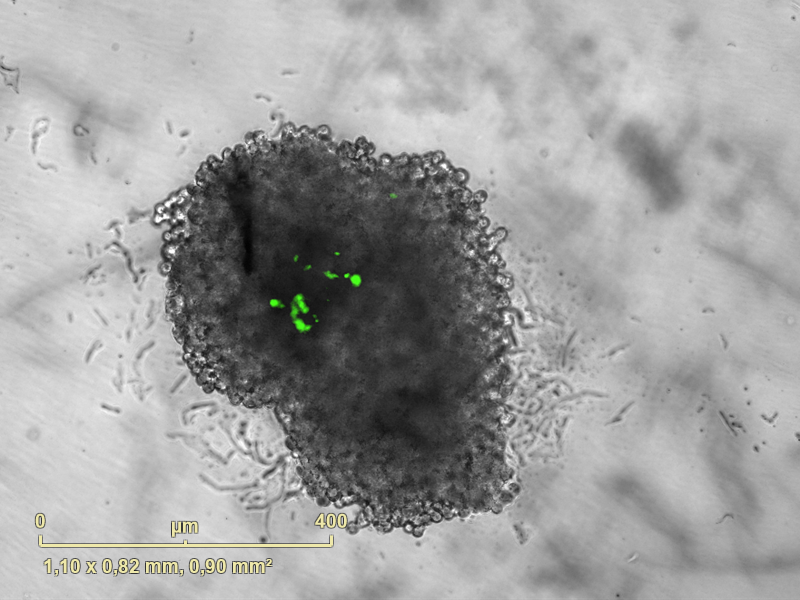

Supplement: Supplementary file 5 — Source data Fig. 4 [file 44318_2025_371_MOESM5_ESM.zip › SourceData_Figure 4/4I/MDA-231_Abema_C1_1_2024y04m07d_13h13m.tif]

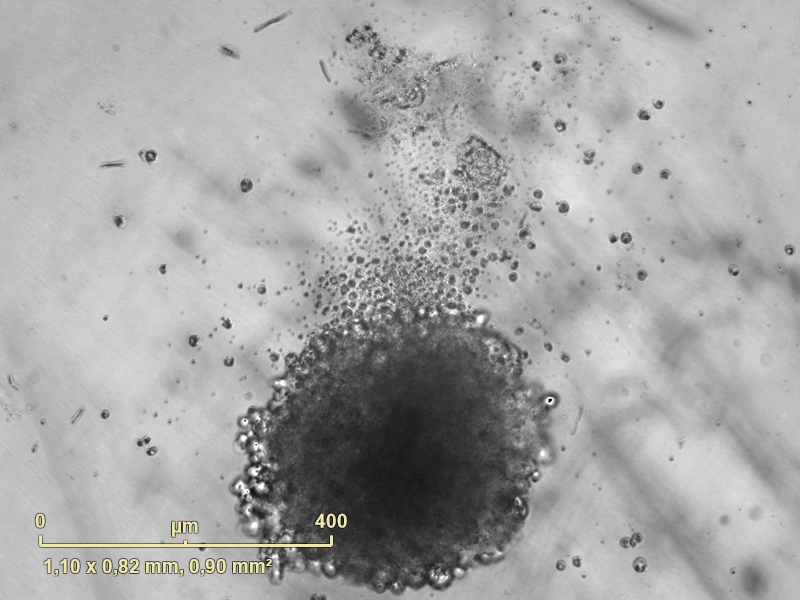

Supplement: Supplementary file 5 — Source data Fig. 4 [file 44318_2025_371_MOESM5_ESM.zip › SourceData_Figure 4/4I/MDA-231_Abema_B4_1_2024y04m06d_13h13m.tif]

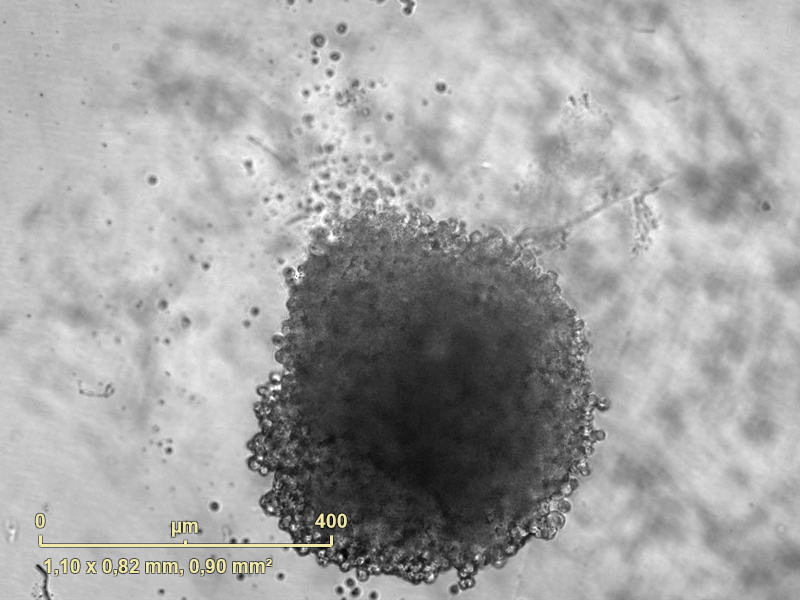

Supplement: Supplementary file 5 — Source data Fig. 4 [file 44318_2025_371_MOESM5_ESM.zip › SourceData_Figure 4/4I/MDA-231_Abema_A3_1_2024y04m06d_13h13m.tif]

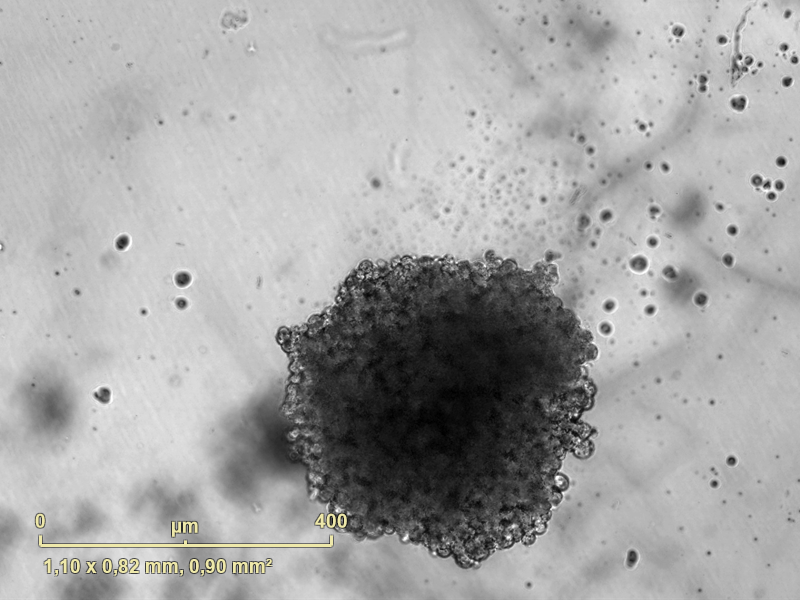

Supplement: Supplementary file 5 — Source data Fig. 4 [file 44318_2025_371_MOESM5_ESM.zip › SourceData_Figure 4/4I/MDA-231_Abema_A6_1_2024y04m06d_13h13m.tif]

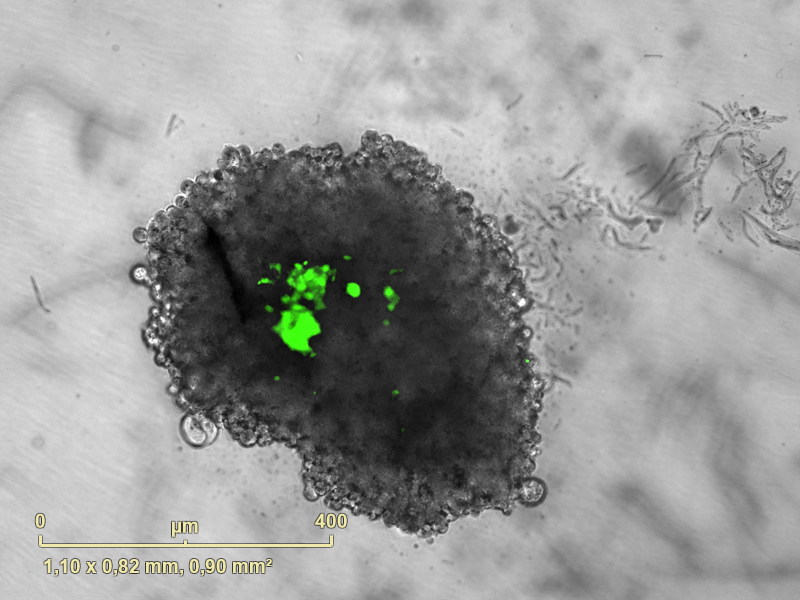

Supplement: Supplementary file 5 — Source data Fig. 4 [file 44318_2025_371_MOESM5_ESM.zip › SourceData_Figure 4/4I/MDA-231_Abema_C1_1_2024y04m08d_13h13m.tif]

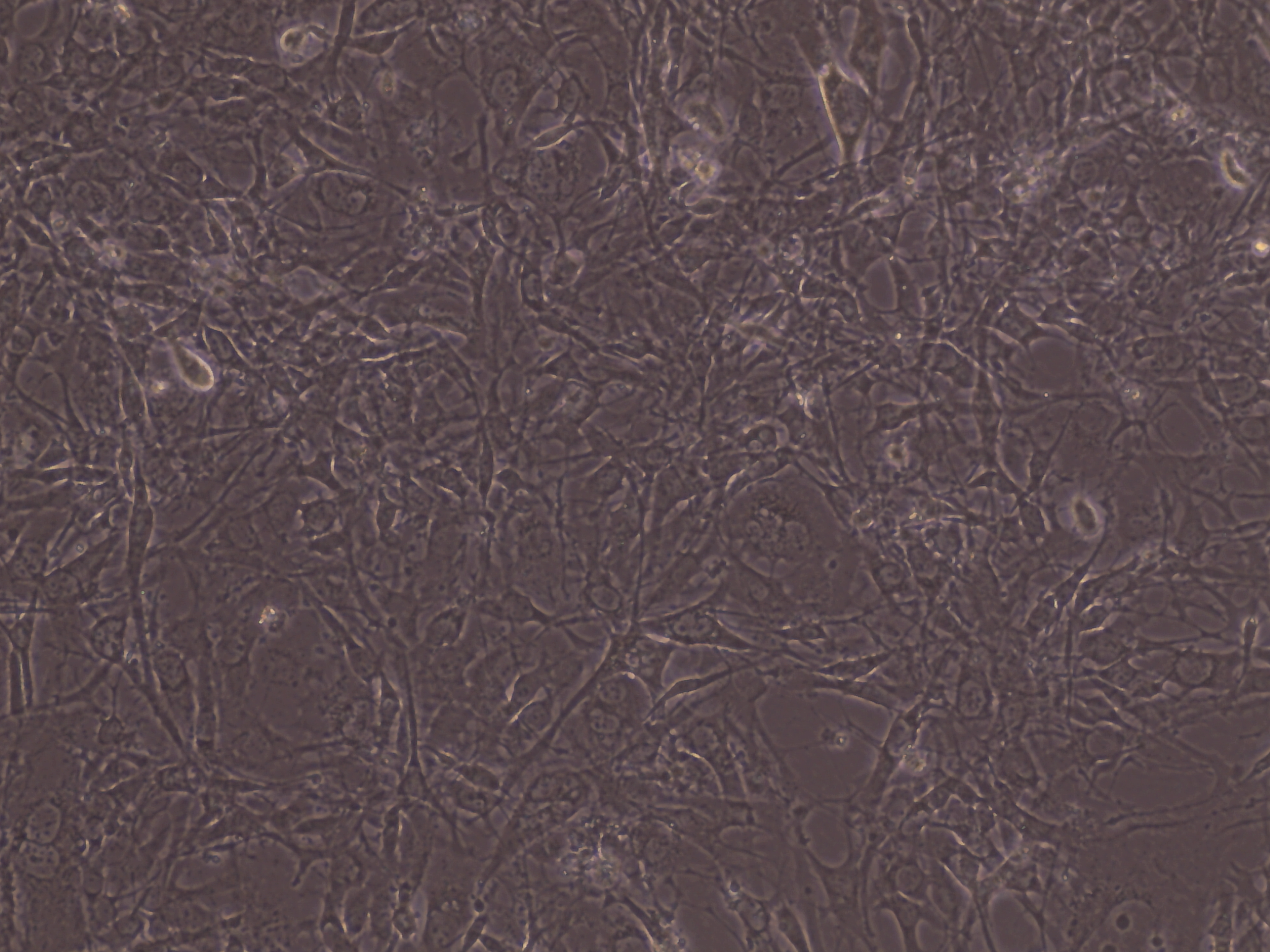

Supplement: Supplementary file 6 — Source data Fig. 5 [file 44318_2025_371_MOESM6_ESM.zip › SourceData_Figure 5/5H/bt549 ctrl 2.tif]

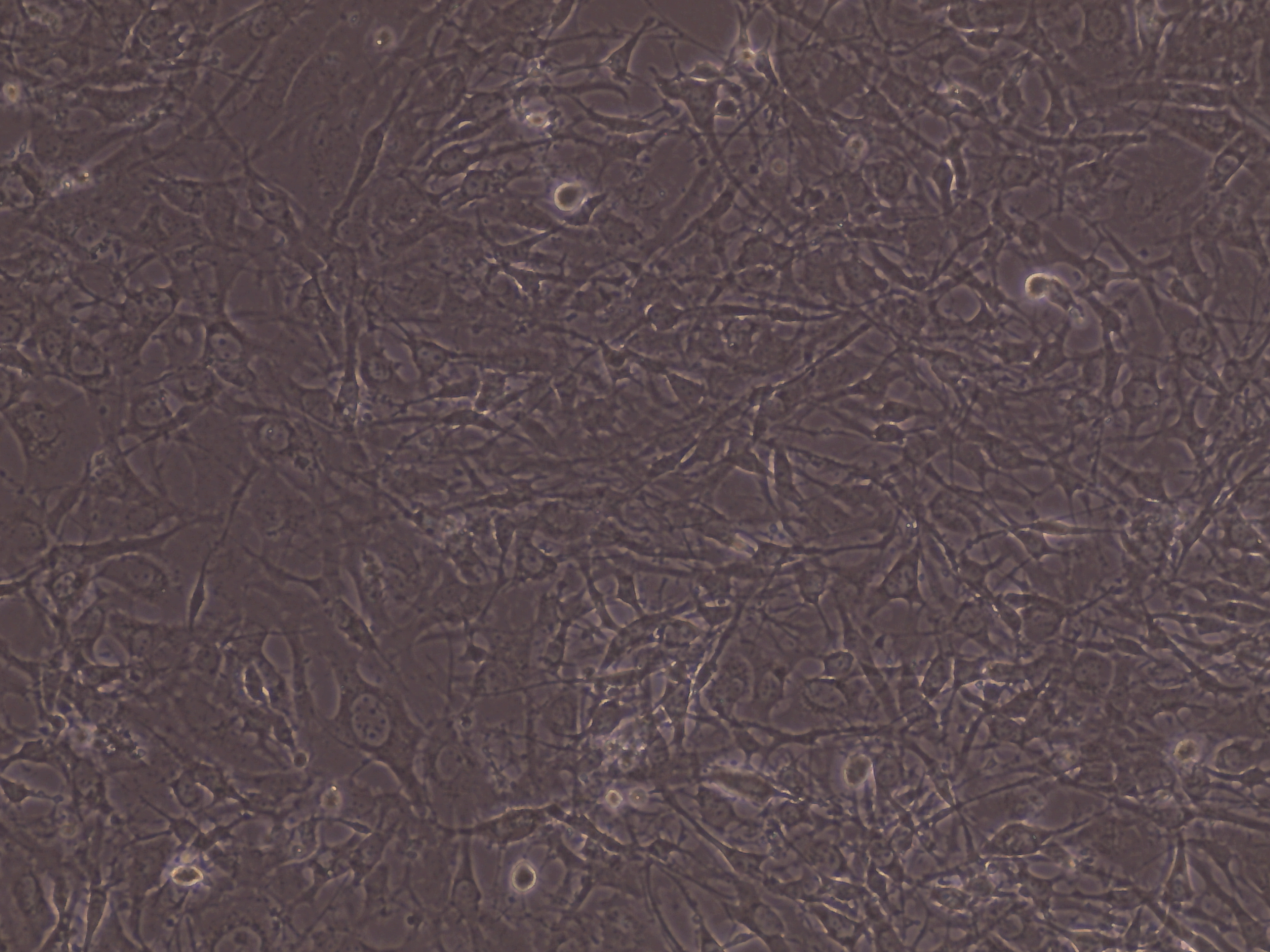

Supplement: Supplementary file 6 — Source data Fig. 5 [file 44318_2025_371_MOESM6_ESM.zip › SourceData_Figure 5/5H/bt549 ctrl 1.tif]

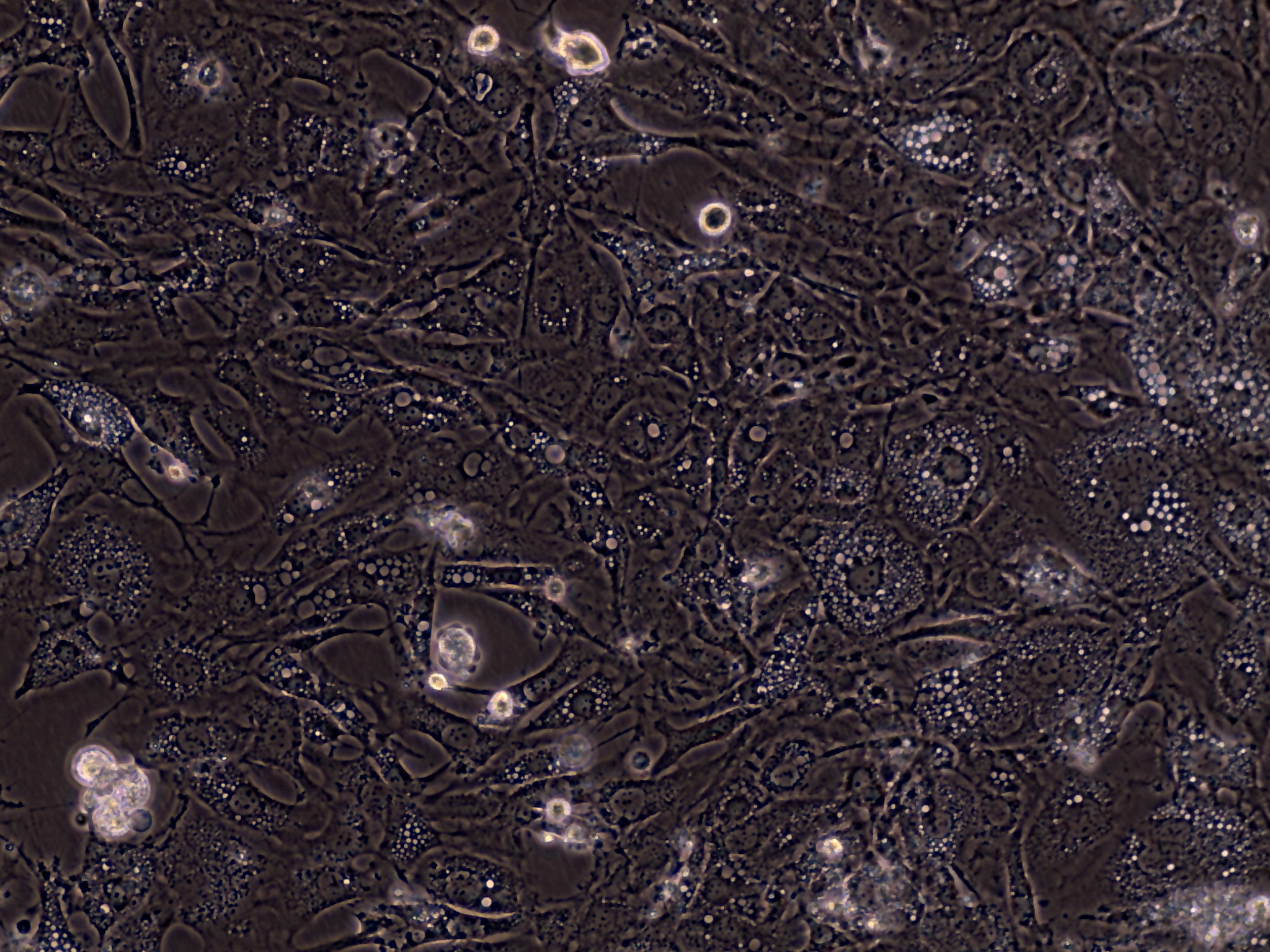

Supplement: Supplementary file 6 — Source data Fig. 5 [file 44318_2025_371_MOESM6_ESM.zip › SourceData_Figure 5/5H/bt549 vac 1.tif]

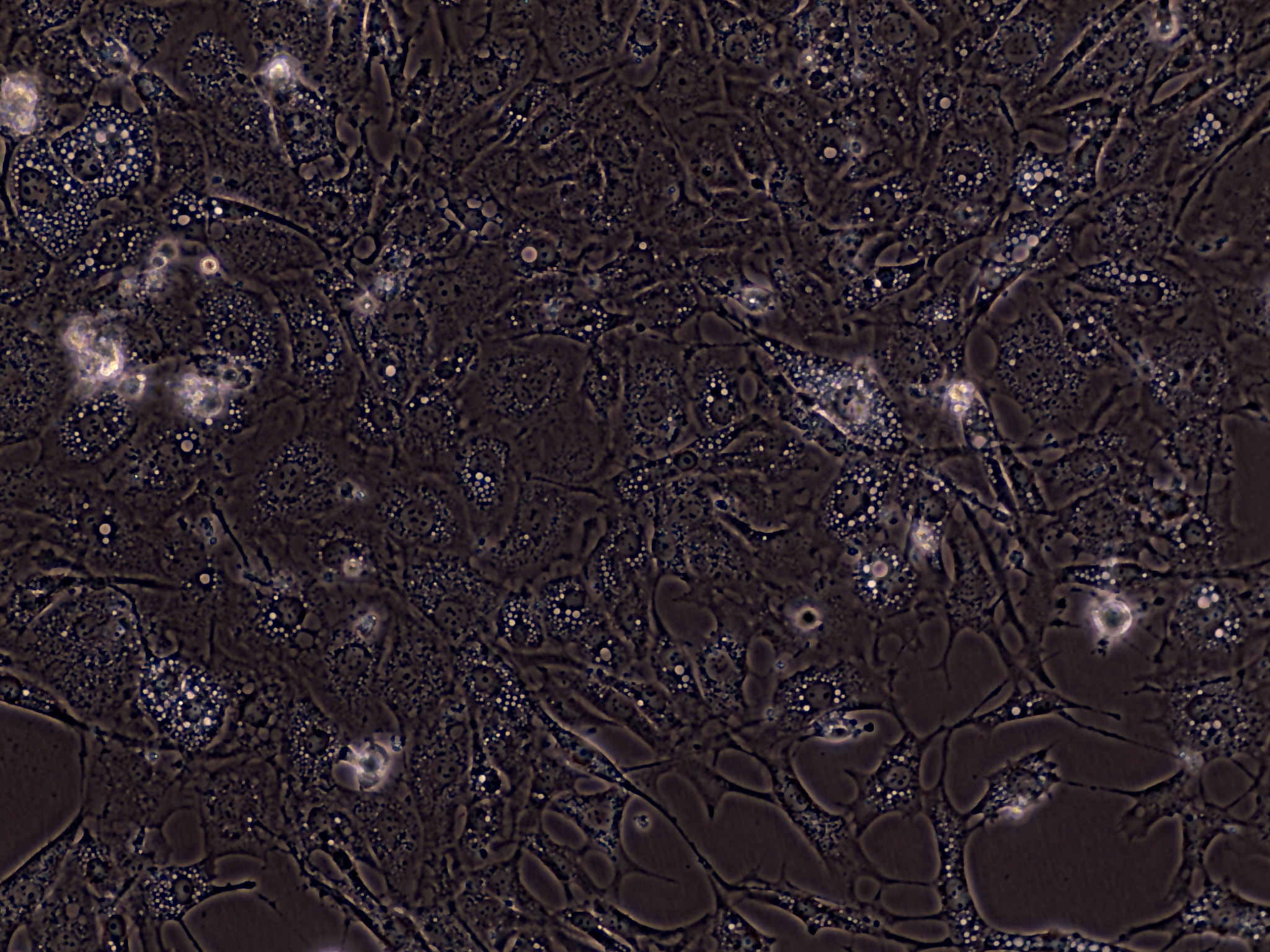

Supplement: Supplementary file 6 — Source data Fig. 5 [file 44318_2025_371_MOESM6_ESM.zip › SourceData_Figure 5/5H/bt549 vac 4.tif]

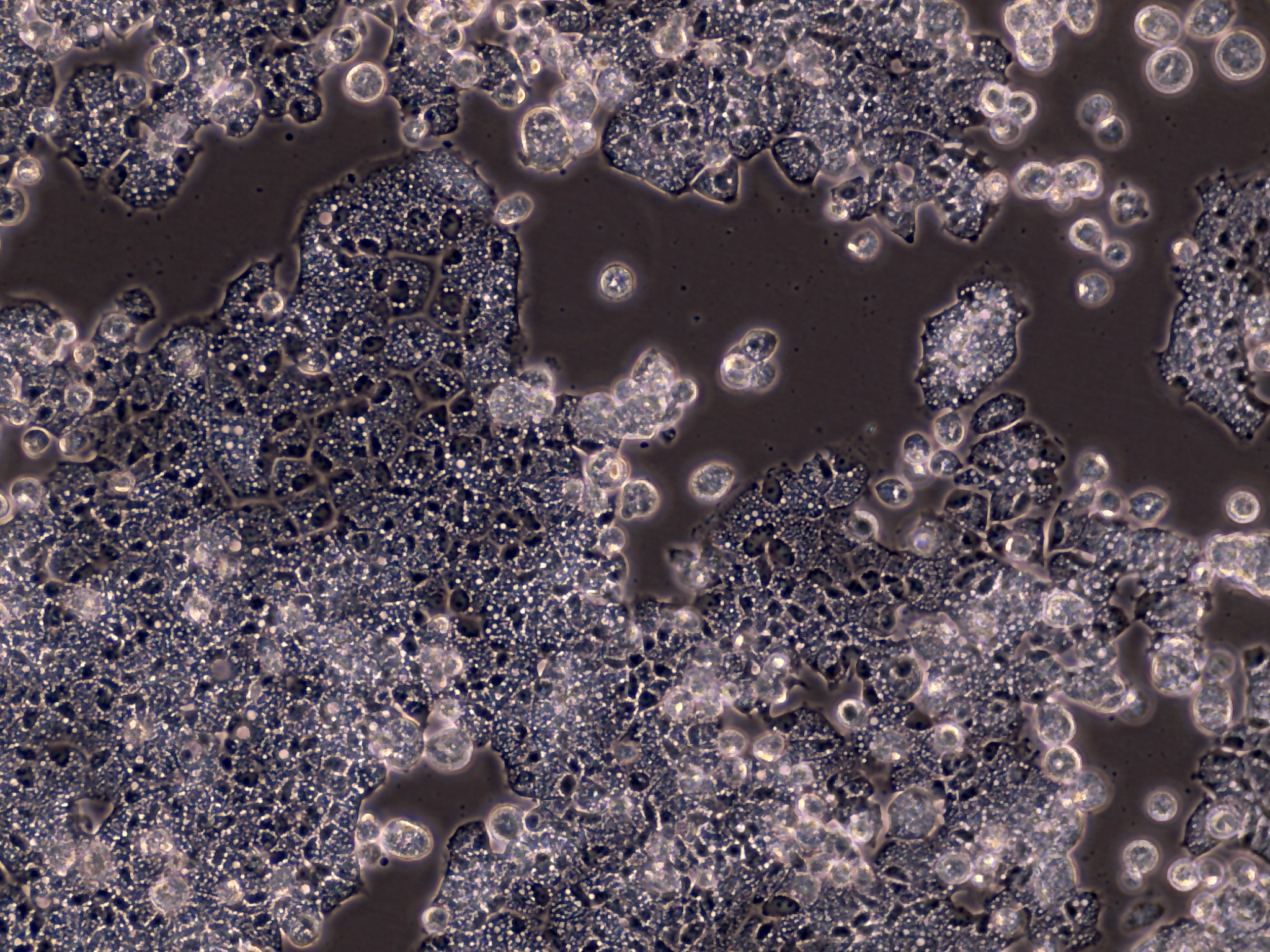

Supplement: Supplementary file 6 — Source data Fig. 5 [file 44318_2025_371_MOESM6_ESM.zip › SourceData_Figure 5/5D/mcf7 vac 7.tif]

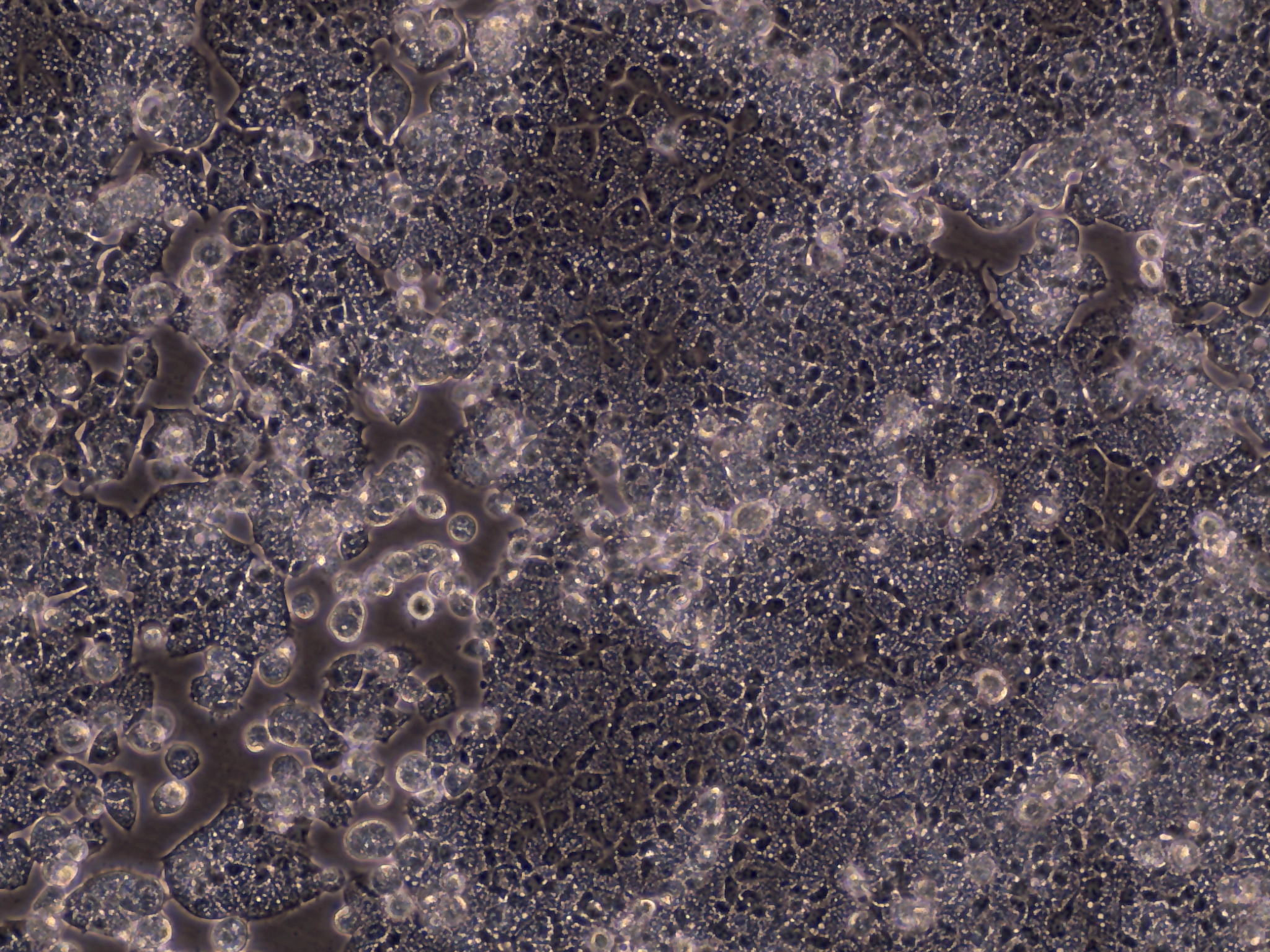

Supplement: Supplementary file 6 — Source data Fig. 5 [file 44318_2025_371_MOESM6_ESM.zip › SourceData_Figure 5/5D/mcf7 vac 2.tif]

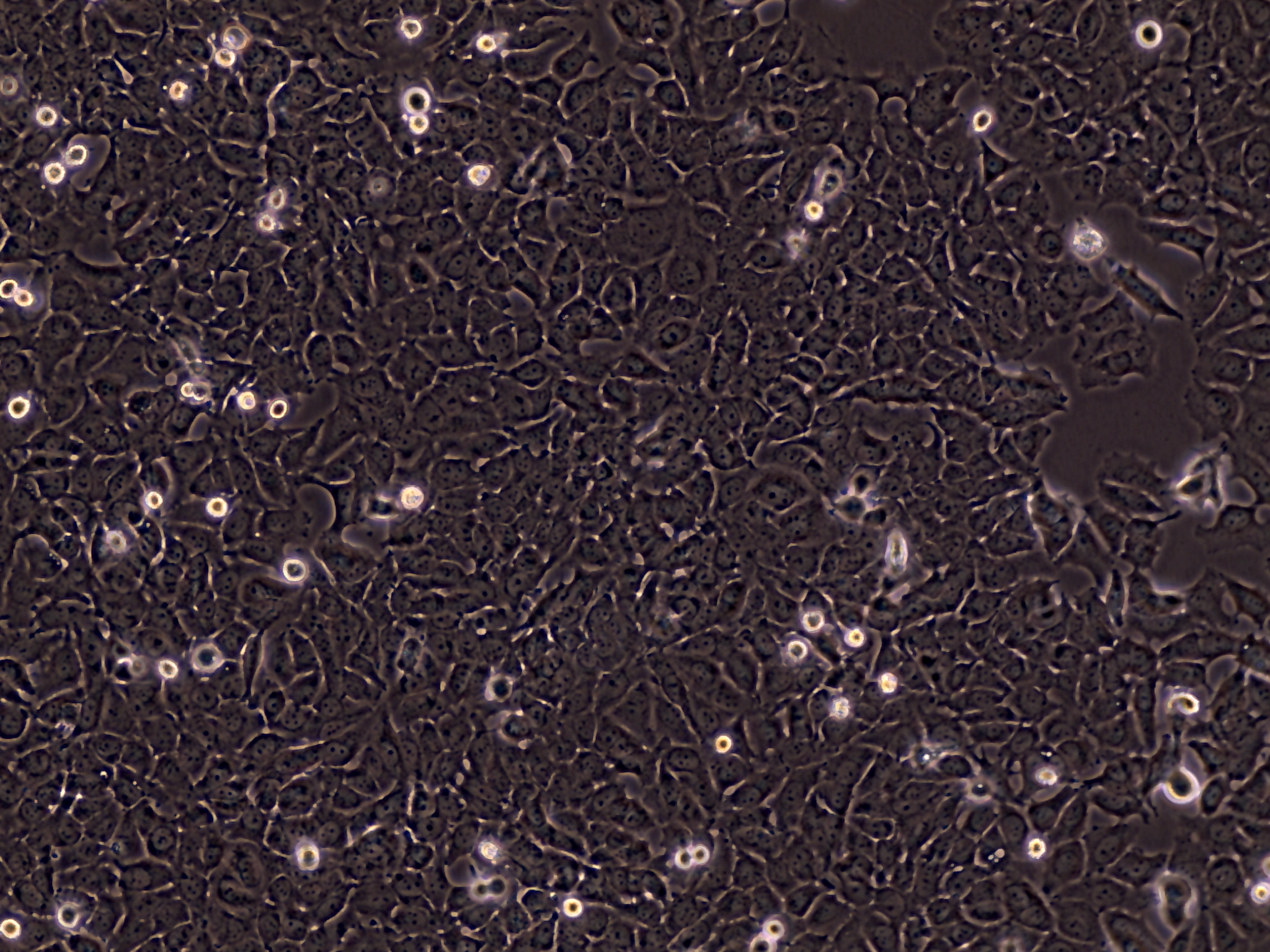

Supplement: Supplementary file 6 — Source data Fig. 5 [file 44318_2025_371_MOESM6_ESM.zip › SourceData_Figure 5/5D/mcf7 ctrl5.tif]

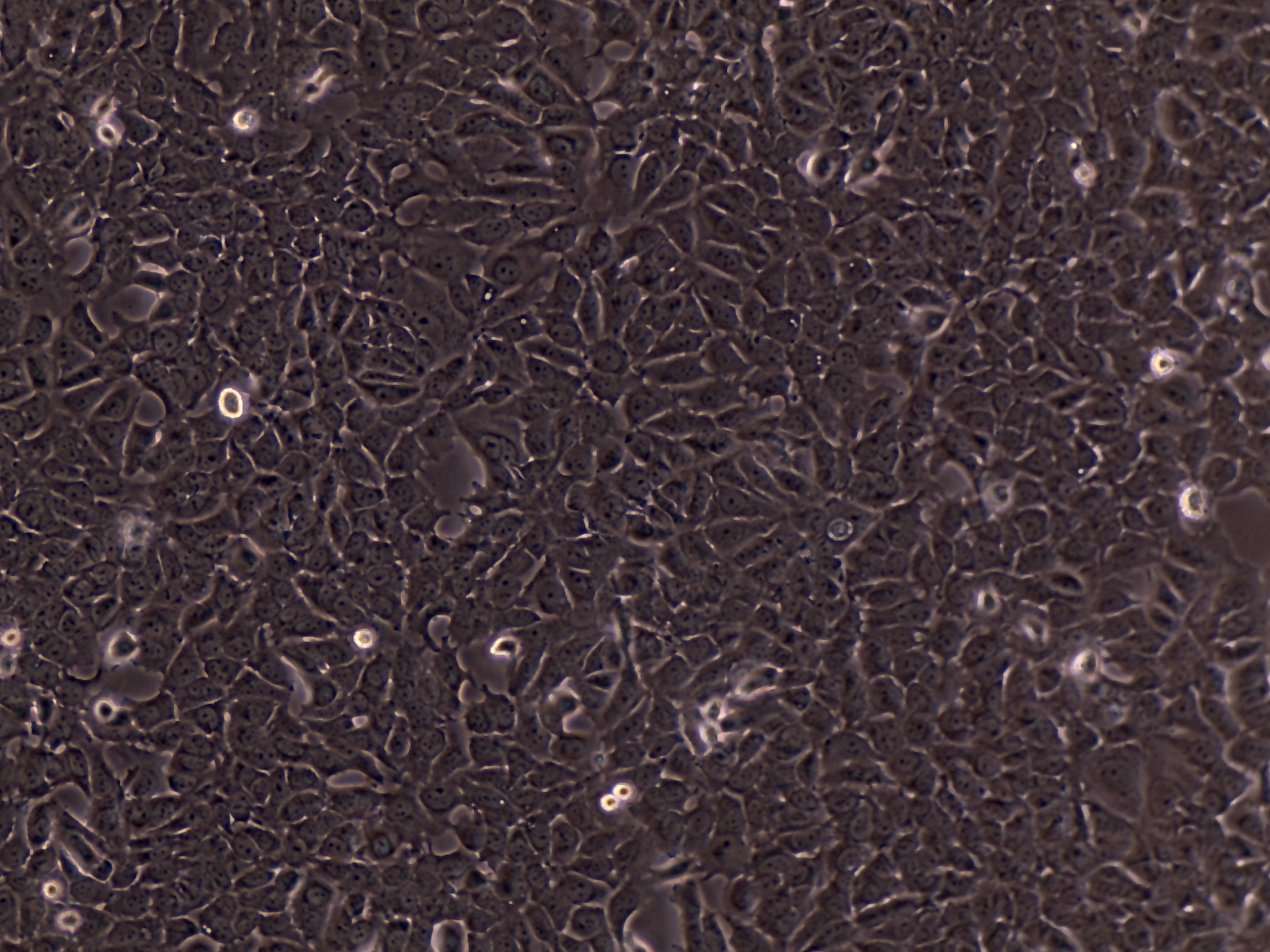

Supplement: Supplementary file 6 — Source data Fig. 5 [file 44318_2025_371_MOESM6_ESM.zip › SourceData_Figure 5/5D/mcf7 ctrl6.tif]

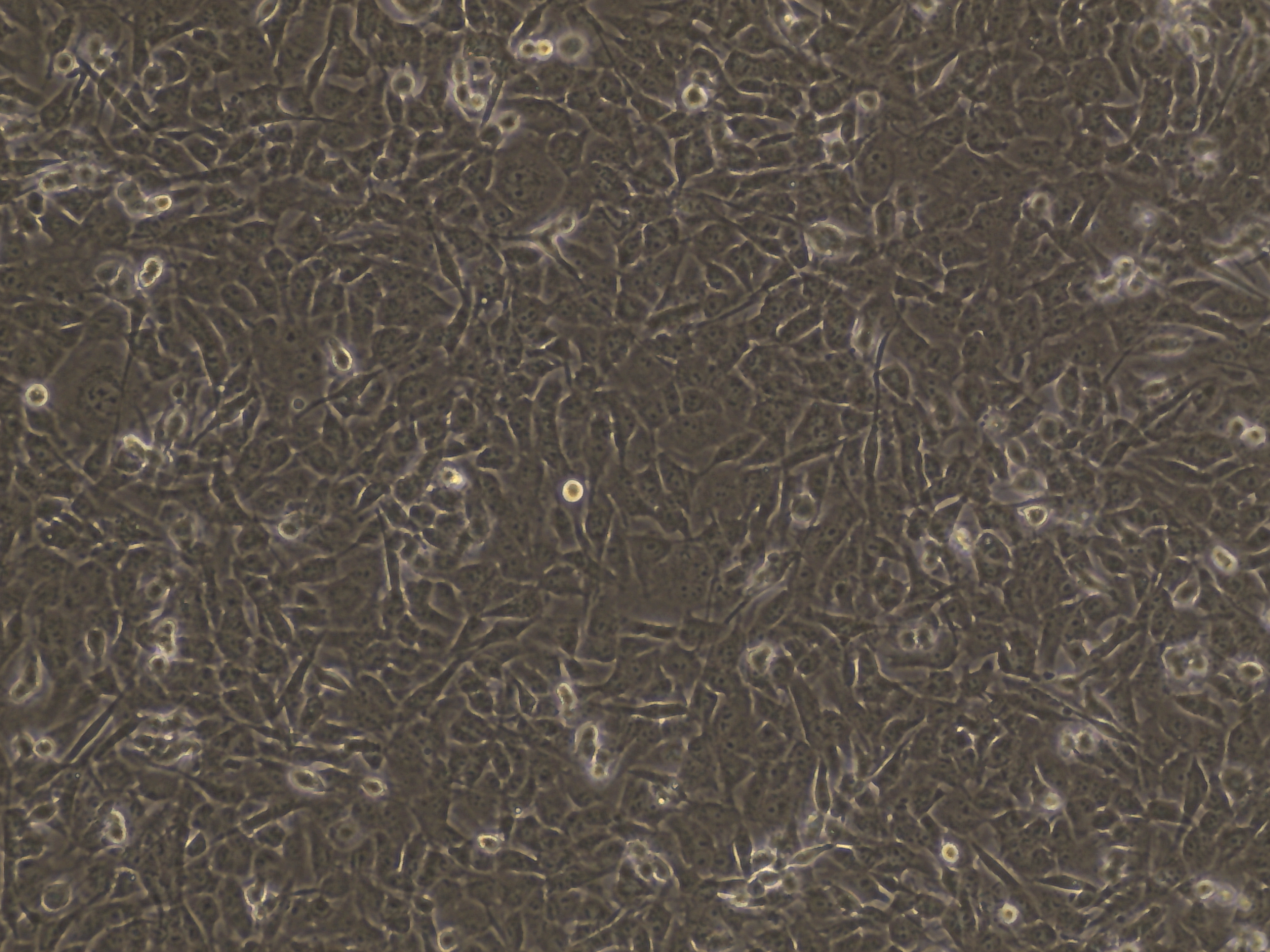

Supplement: Supplementary file 6 — Source data Fig. 5 [file 44318_2025_371_MOESM6_ESM.zip › SourceData_Figure 5/5E/mda231 ctrl 5.tif]

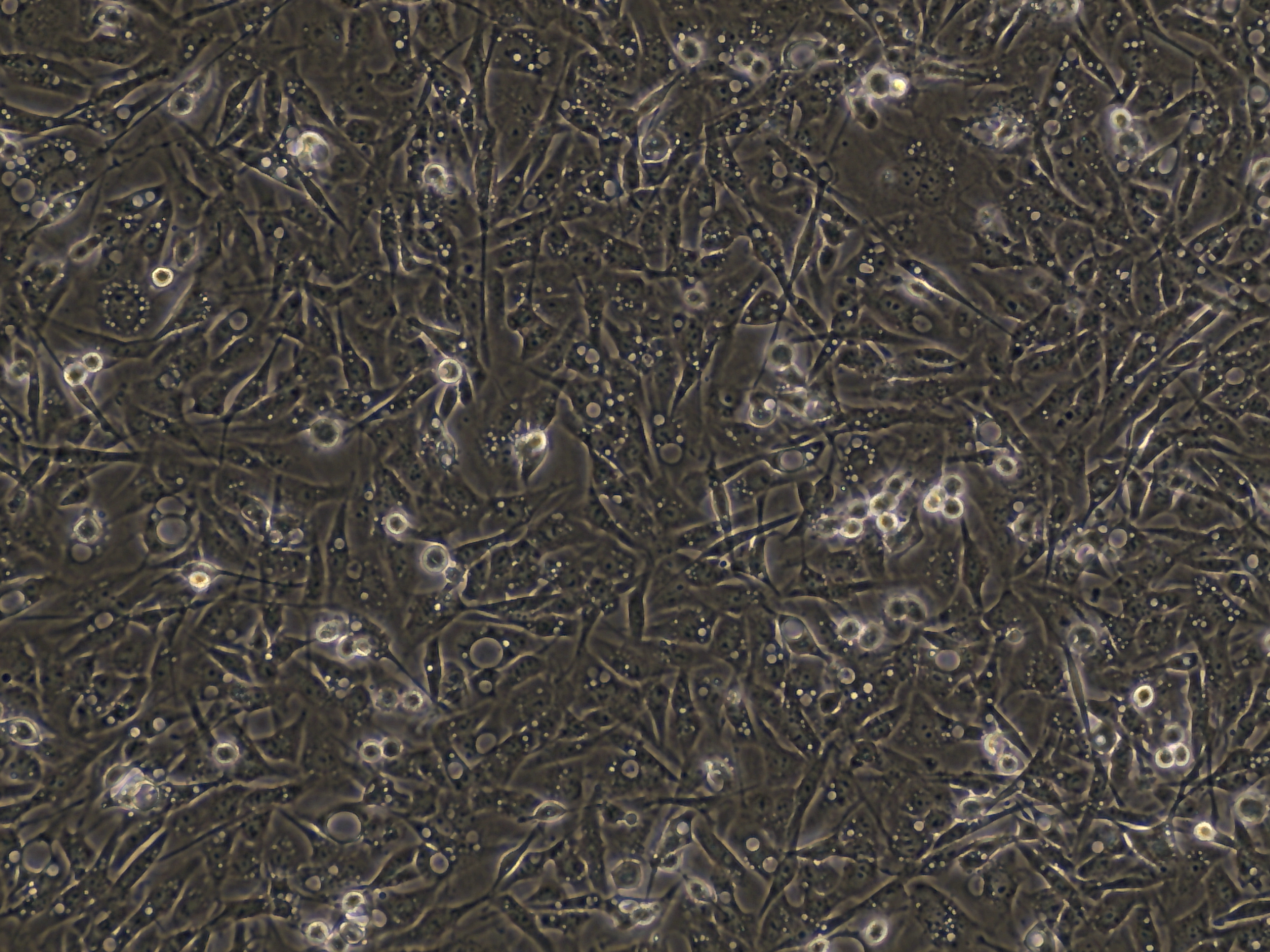

Supplement: Supplementary file 6 — Source data Fig. 5 [file 44318_2025_371_MOESM6_ESM.zip › SourceData_Figure 5/5E/mda231 vac 1.tif]

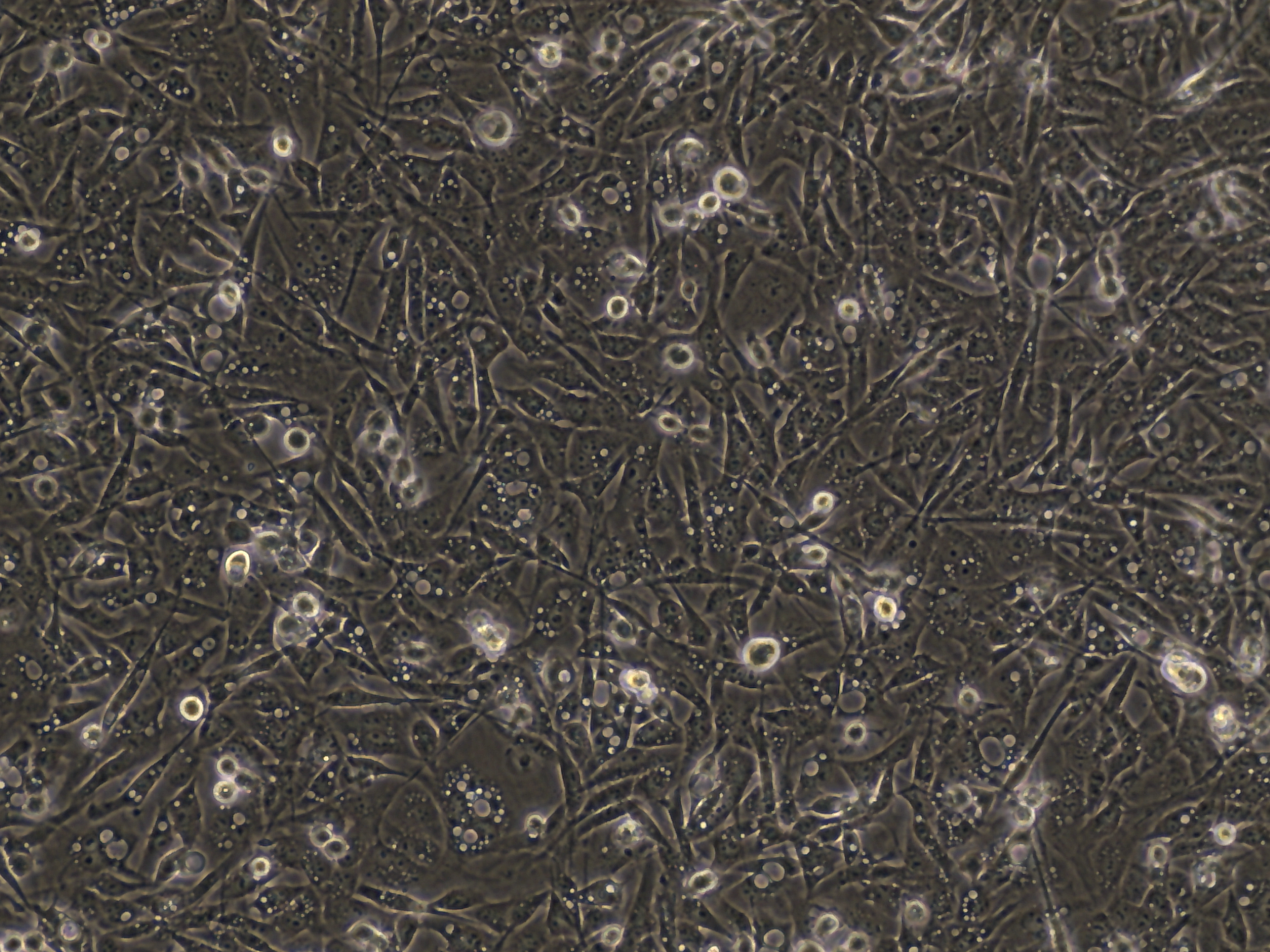

Supplement: Supplementary file 6 — Source data Fig. 5 [file 44318_2025_371_MOESM6_ESM.zip › SourceData_Figure 5/5E/mda231 vac 2.tif]

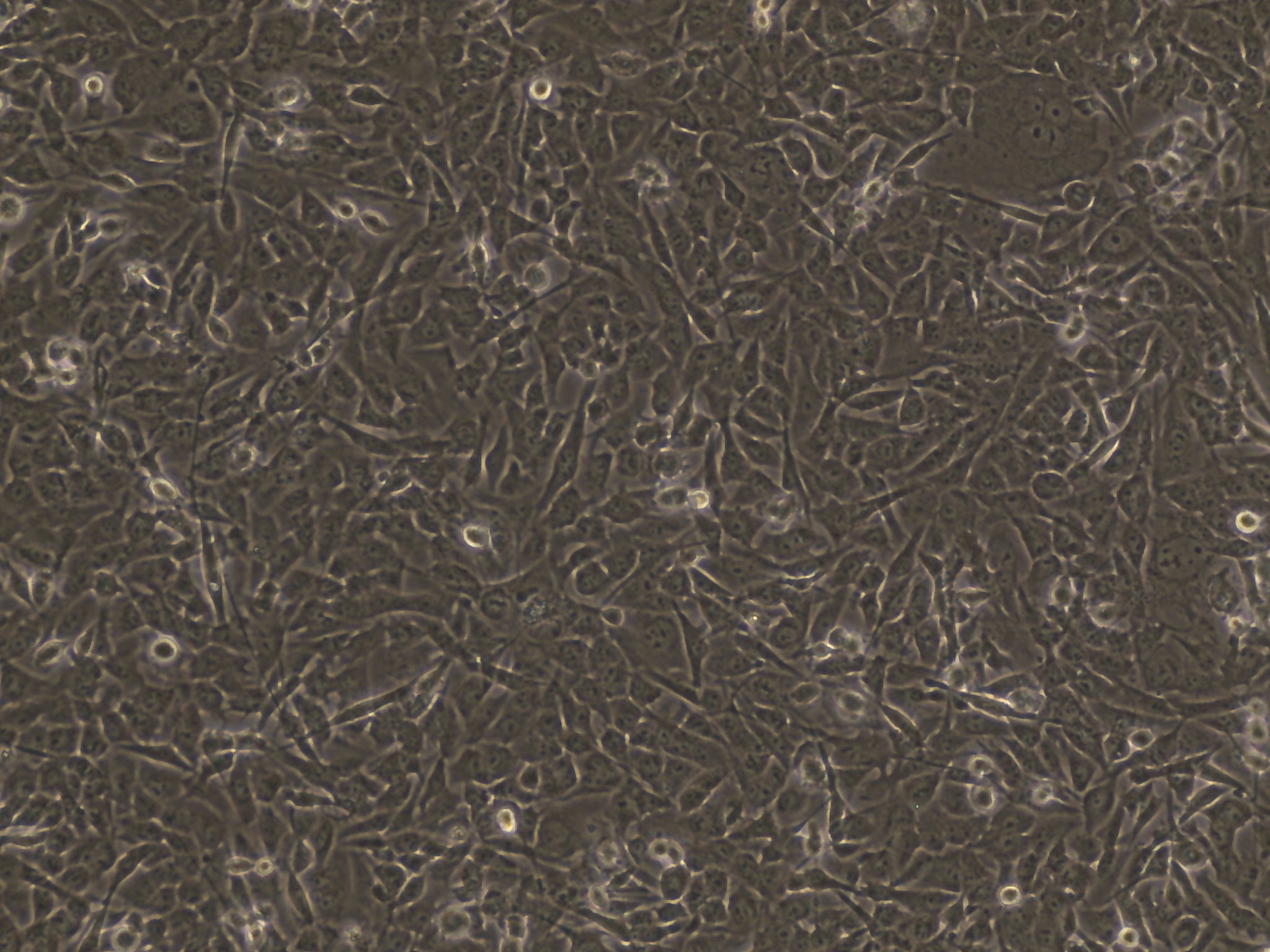

Supplement: Supplementary file 6 — Source data Fig. 5 [file 44318_2025_371_MOESM6_ESM.zip › SourceData_Figure 5/5E/mda231 ctrl 3.tif]

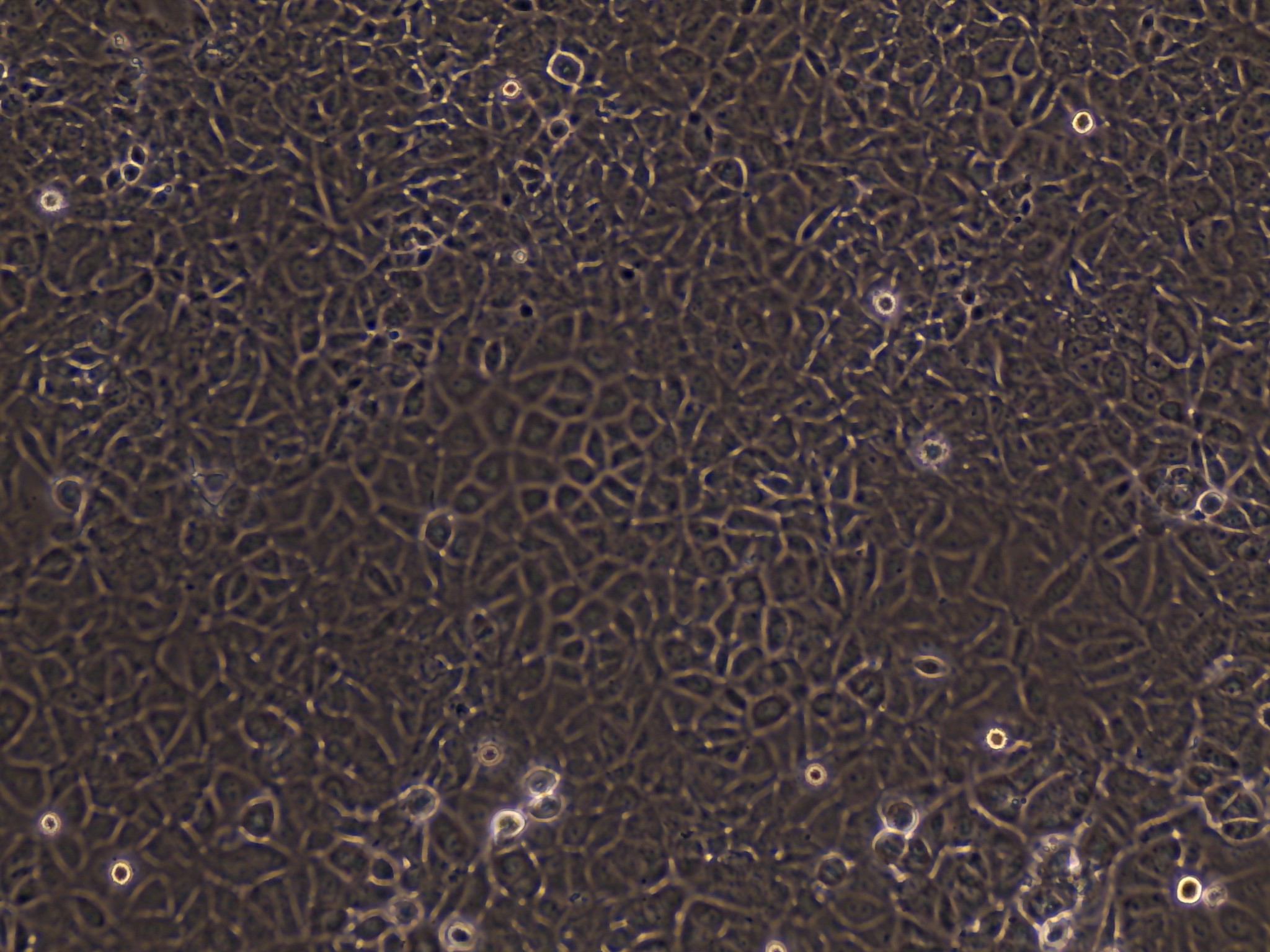

Supplement: Supplementary file 6 — Source data Fig. 5 [file 44318_2025_371_MOESM6_ESM.zip › SourceData_Figure 5/5B/mcf7 ctrl 1.jpg]

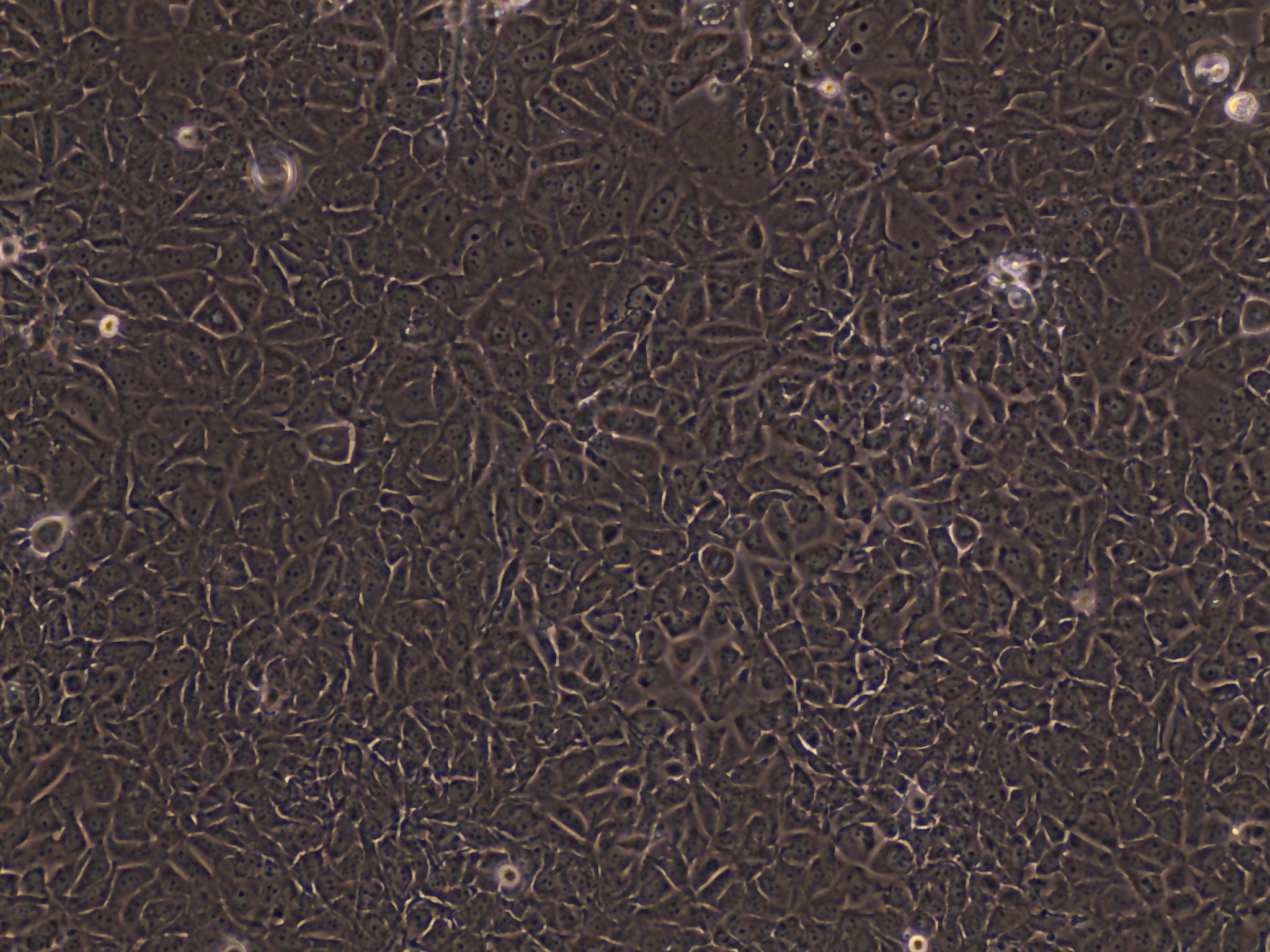

Supplement: Supplementary file 6 — Source data Fig. 5 [file 44318_2025_371_MOESM6_ESM.zip › SourceData_Figure 5/5B/mcf7 ctrl 3.jpg]

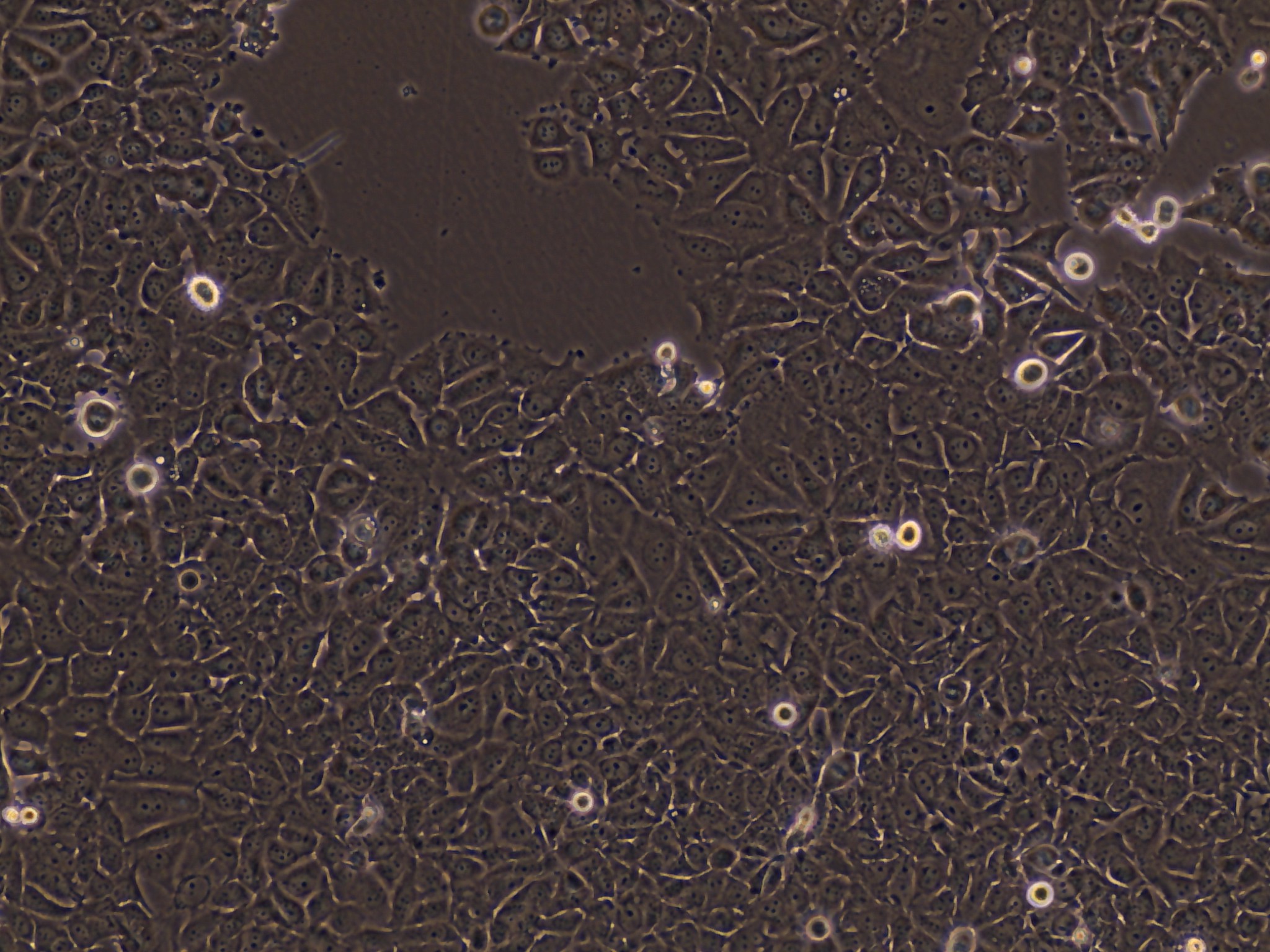

Supplement: Supplementary file 6 — Source data Fig. 5 [file 44318_2025_371_MOESM6_ESM.zip › SourceData_Figure 5/5B/mcf7 ctrl 2.jpg]

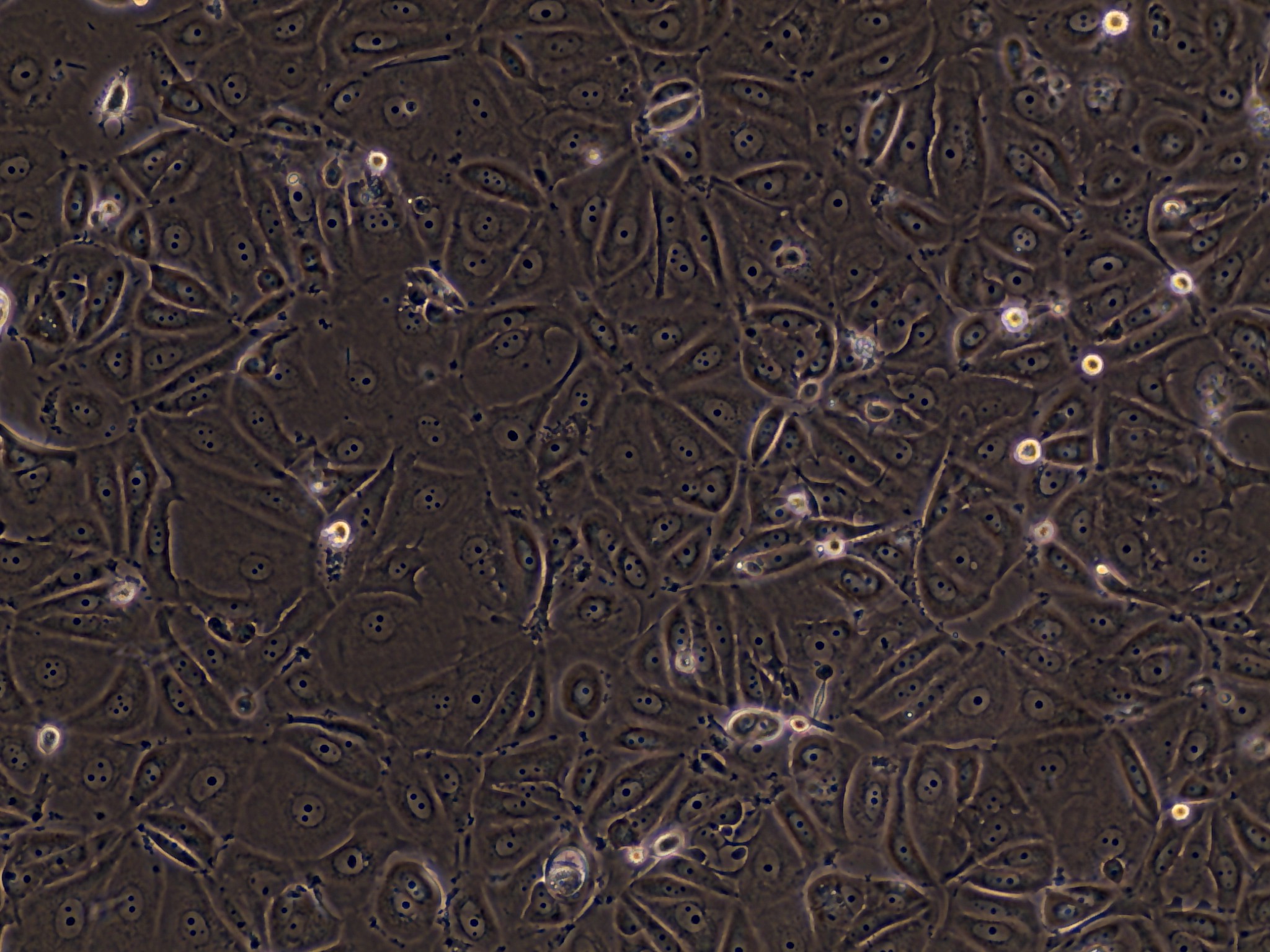

Supplement: Supplementary file 6 — Source data Fig. 5 [file 44318_2025_371_MOESM6_ESM.zip › SourceData_Figure 5/5B/mcf7 palbo1.jpg]

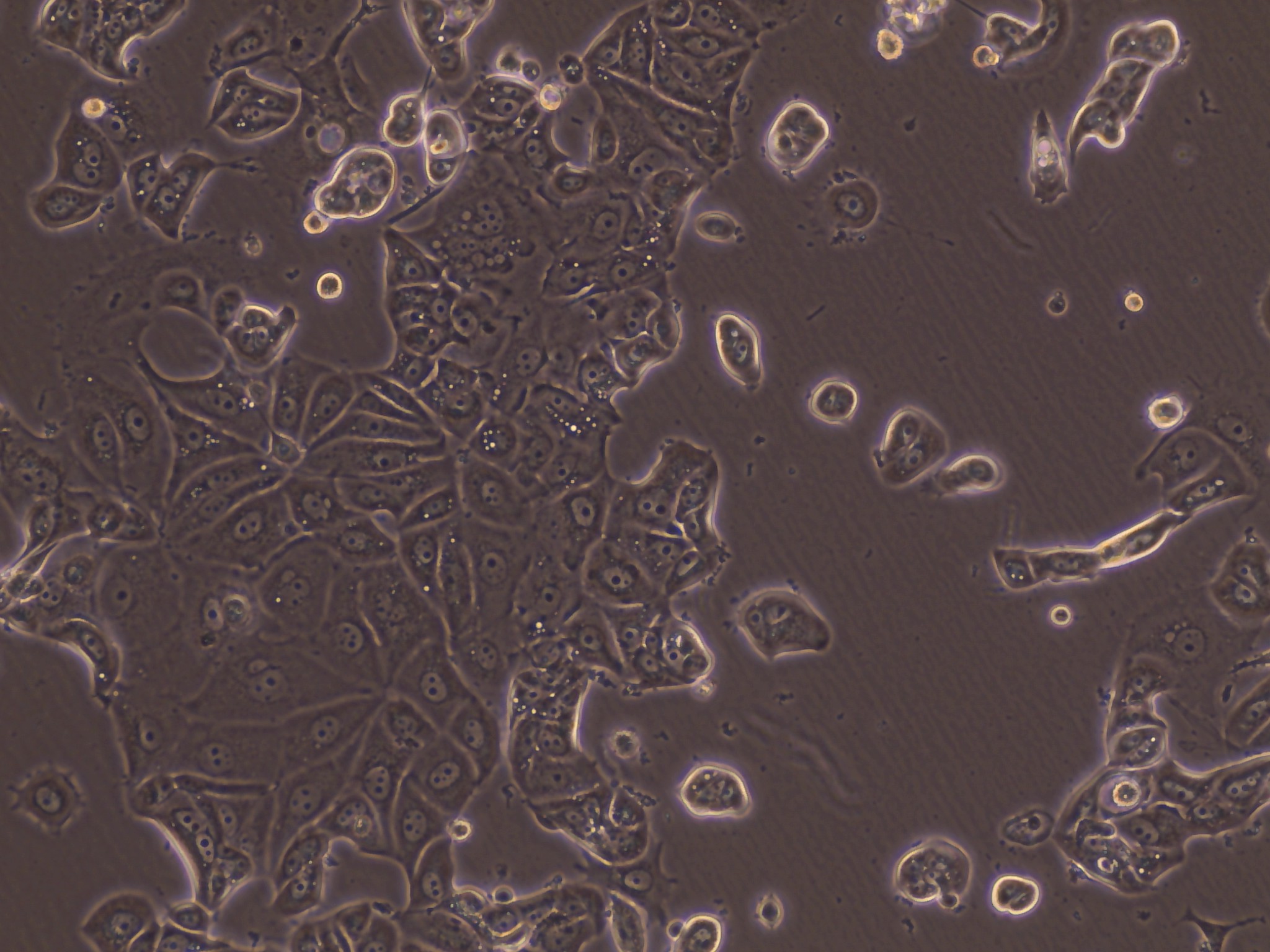

Supplement: Supplementary file 6 — Source data Fig. 5 [file 44318_2025_371_MOESM6_ESM.zip › SourceData_Figure 5/5B/mcf7 abema4.jpg]

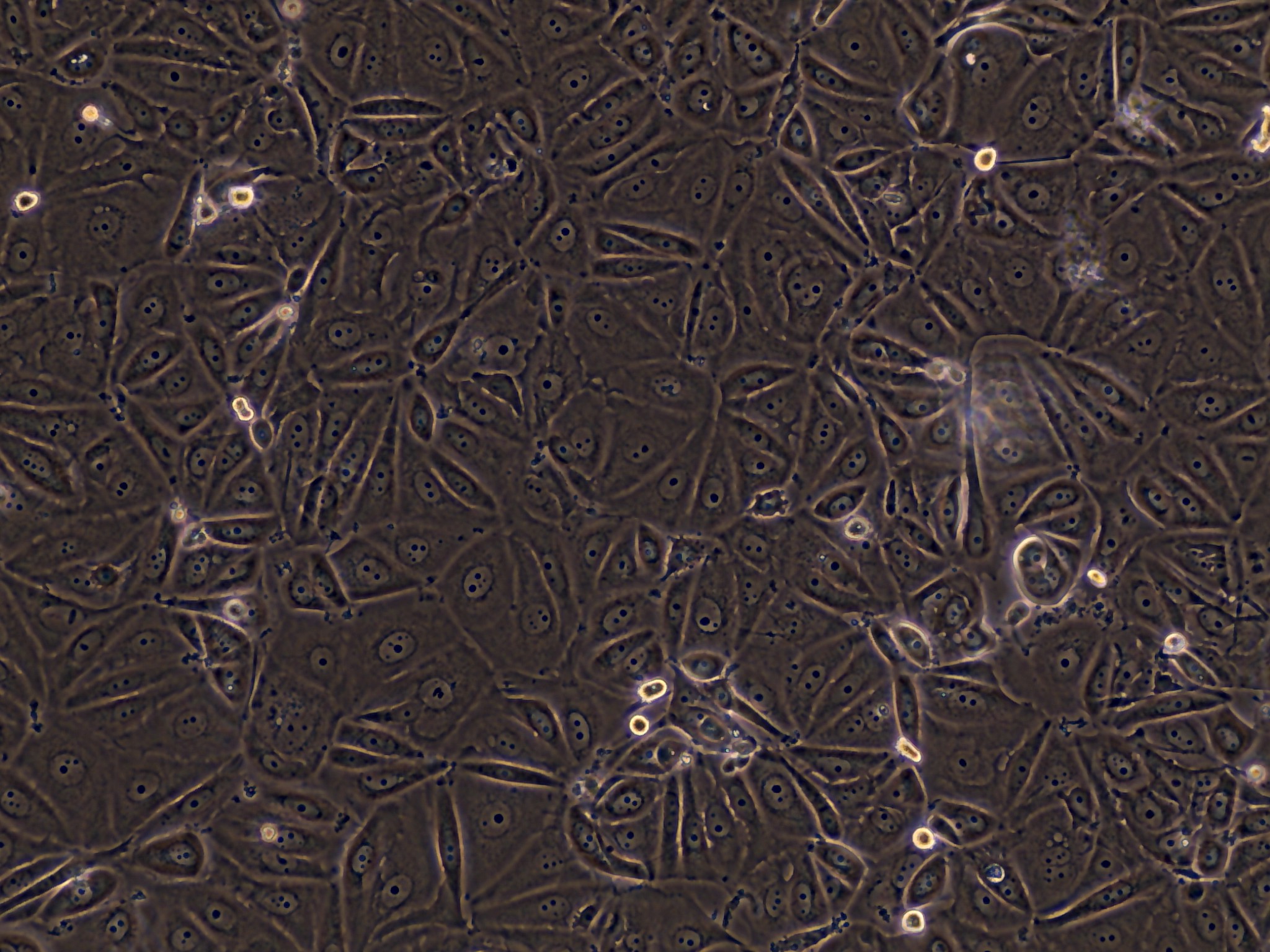

Supplement: Supplementary file 6 — Source data Fig. 5 [file 44318_2025_371_MOESM6_ESM.zip › SourceData_Figure 5/5B/mcf7 palbo2.jpg]

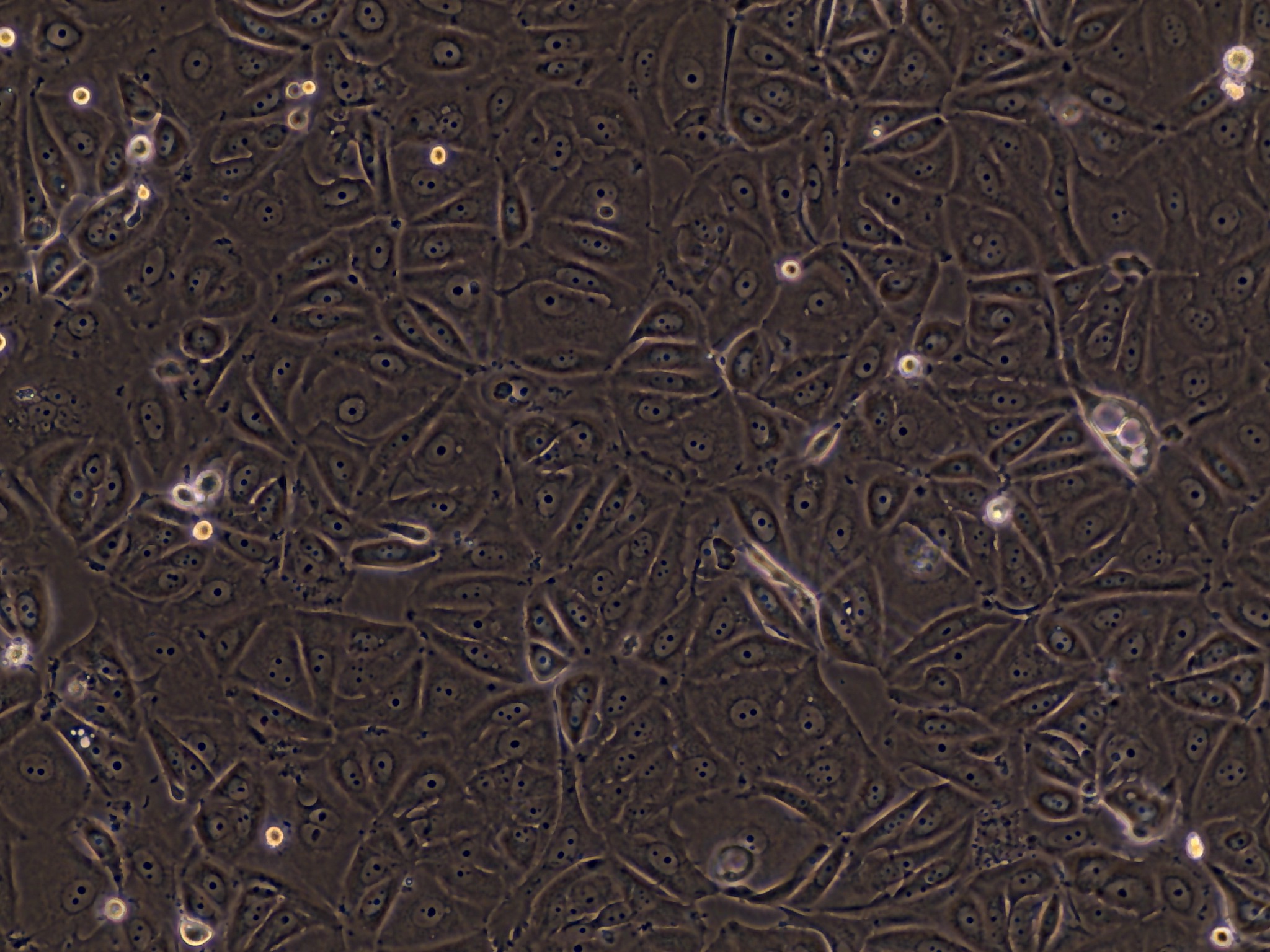

Supplement: Supplementary file 6 — Source data Fig. 5 [file 44318_2025_371_MOESM6_ESM.zip › SourceData_Figure 5/5B/mcf7 palbo3.jpg]

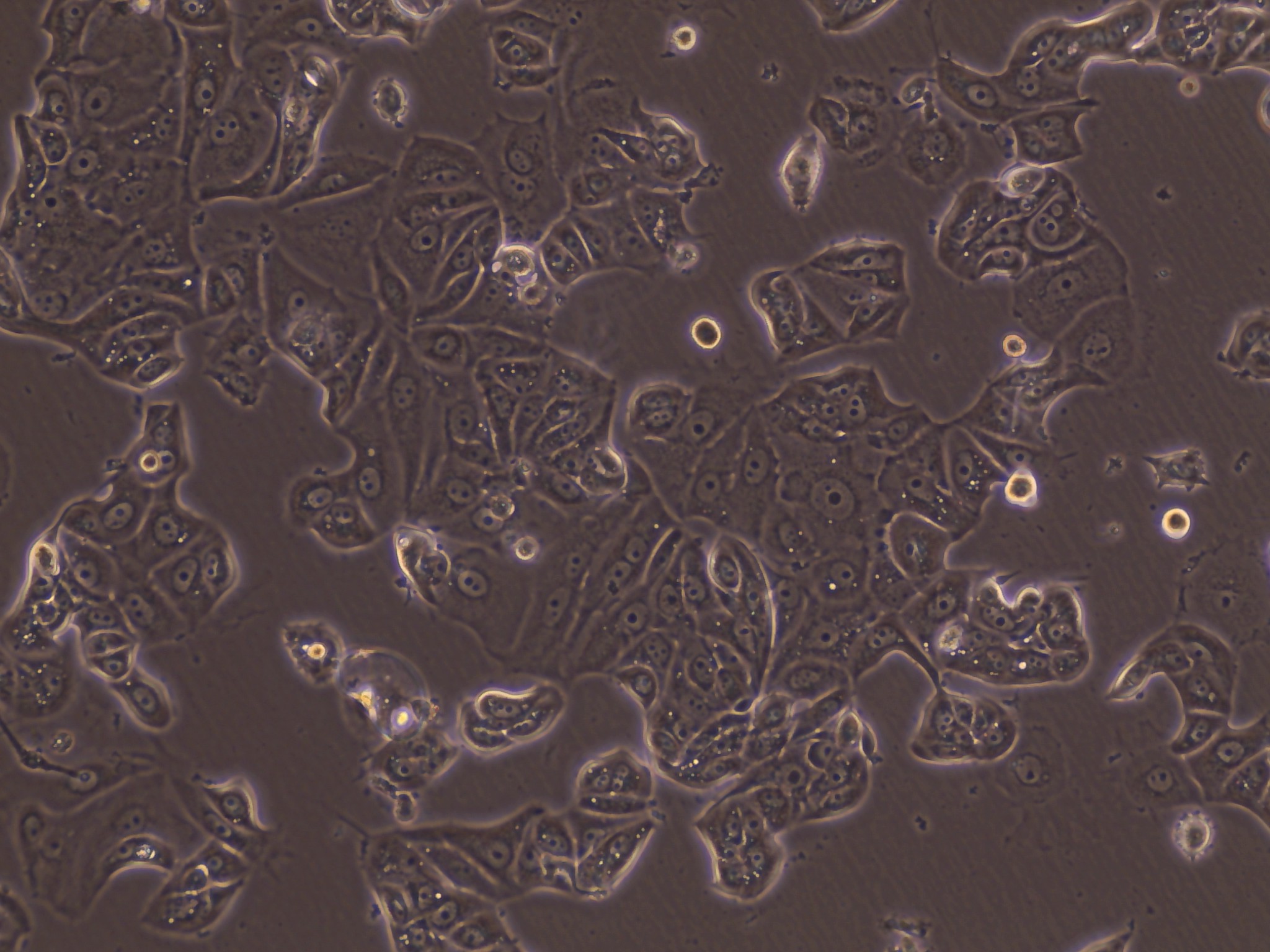

Supplement: Supplementary file 6 — Source data Fig. 5 [file 44318_2025_371_MOESM6_ESM.zip › SourceData_Figure 5/5B/mcf7 abema2.jpg]

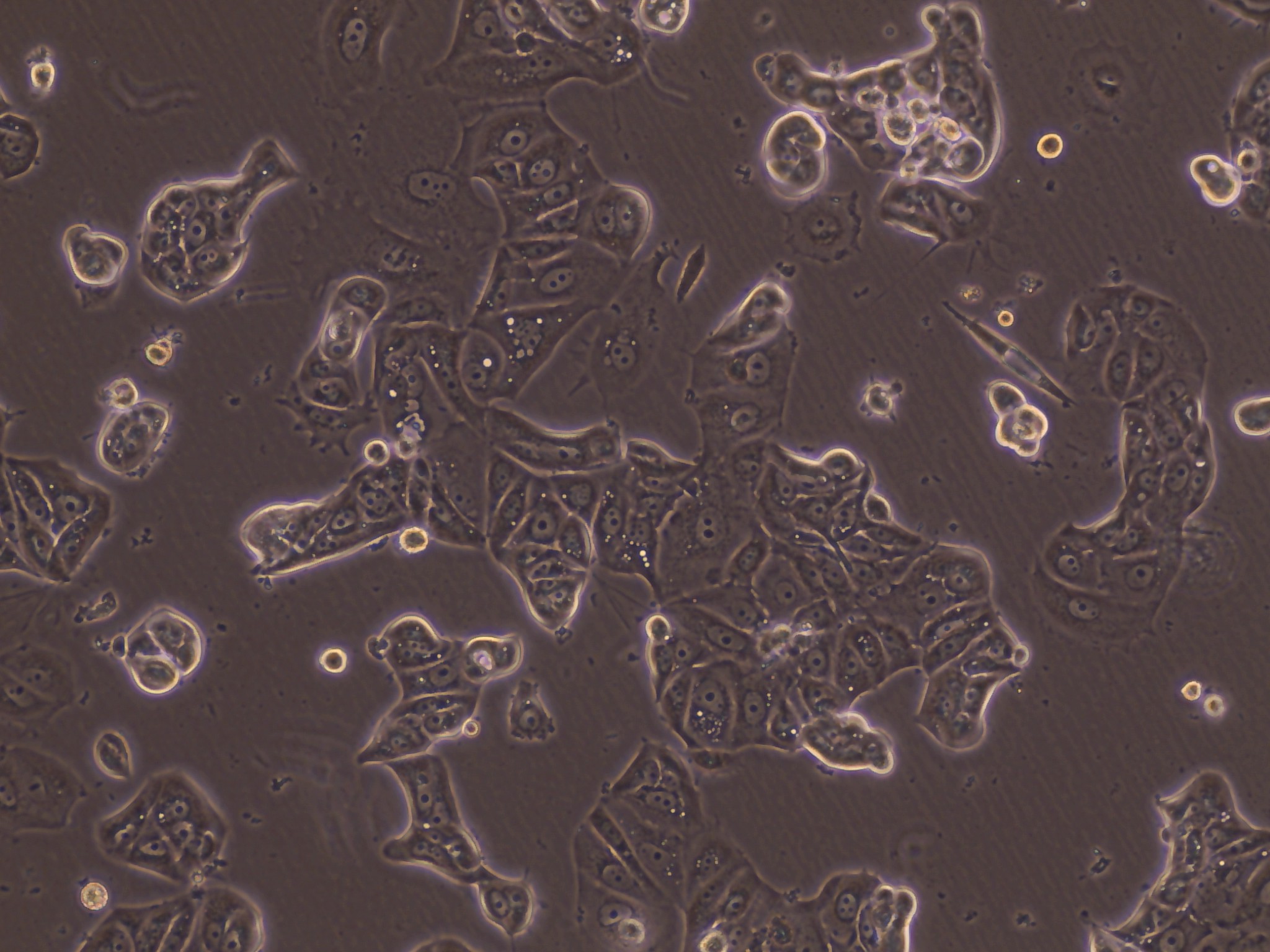

Supplement: Supplementary file 6 — Source data Fig. 5 [file 44318_2025_371_MOESM6_ESM.zip › SourceData_Figure 5/5B/mcf7 abema3.jpg]

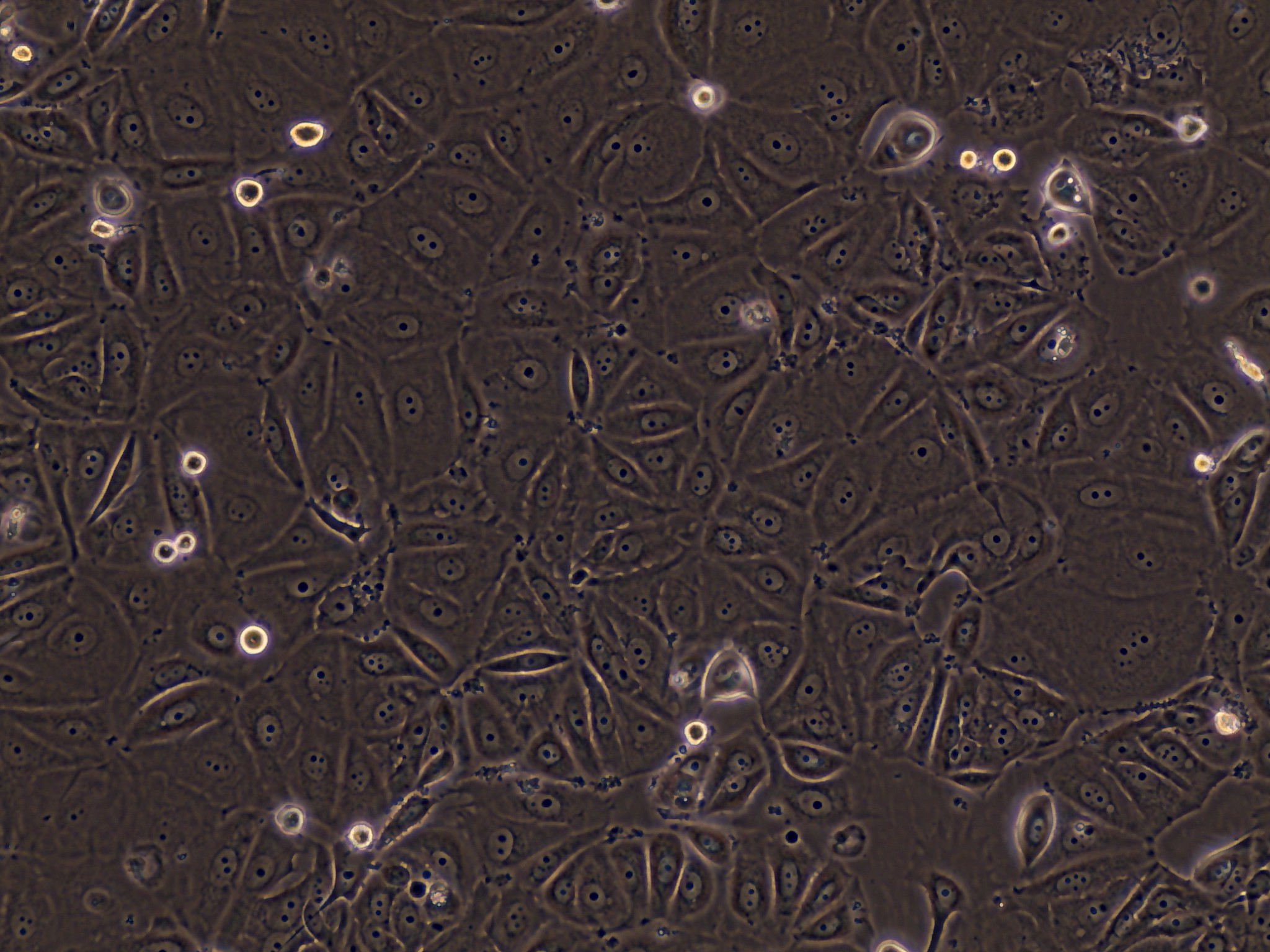

Supplement: Supplementary file 6 — Source data Fig. 5 [file 44318_2025_371_MOESM6_ESM.zip › SourceData_Figure 5/5B/mcf7 palbo4.jpg]

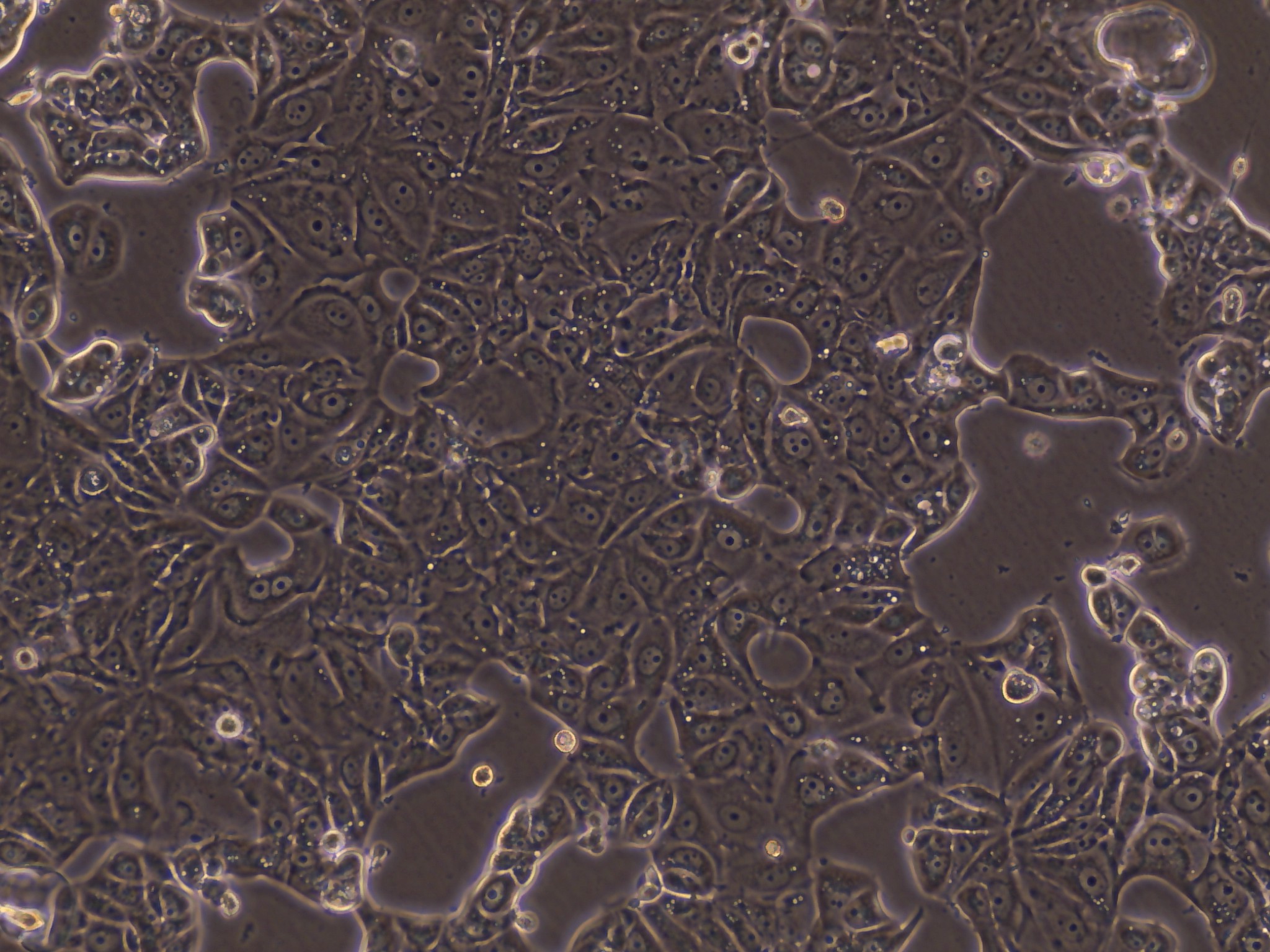

Supplement: Supplementary file 6 — Source data Fig. 5 [file 44318_2025_371_MOESM6_ESM.zip › SourceData_Figure 5/5B/mcf7 abema1.jpg]
