# Supplementary material for: Pharmacological CDK4/6 inhibition promotes vulnerability to lysosomotropic agents in breast cancer
Source: EMBO J. 2025 Feb 10;44(7):1921–42. doi: 10.1038/s44318-025-00371-x (PMC11961731; doi:10.1038/s44318-025-00371-x)
Supplement: Supplementary file 8 — Expanded View Figures [file 44318_2025_371_MOESM8_ESM.pdf]

Expanded View Figures

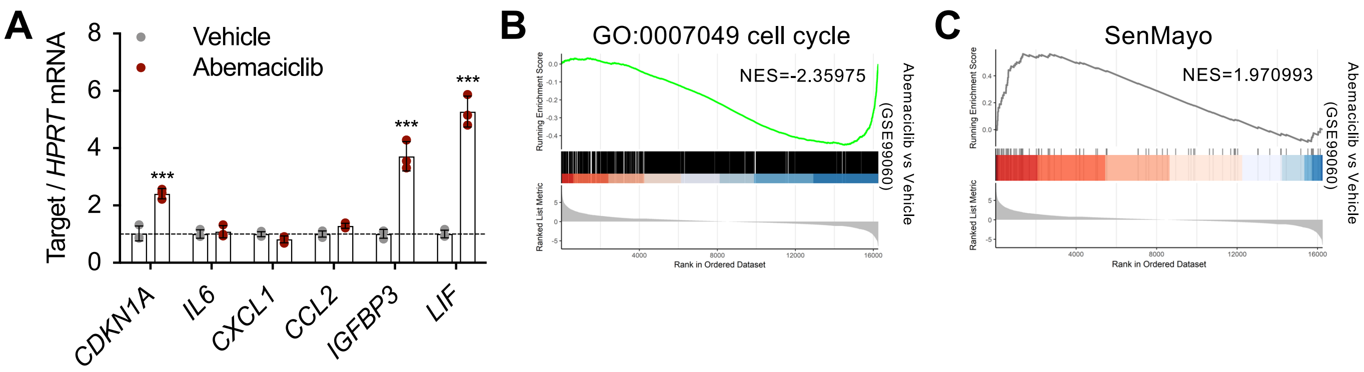

Figure EV1. Abemaciclib treatment induces senescence-like phenotypes in MCF-7 breast cancer cells.

(**A**) RNA was extracted from MCF-7 cells treated with either the vehicle (water) or abemaciclib (1 µM for 8 days), followed by qPCR analysis targeting the specified genes. $n = 3$ independent experiments, $p < 0.0001$. (**B**, **C**) Gene Set Enrichment Analysis (GSEA) plot showing the enrichment for the GO term "cell cycle" (**B**) and the SenMayo geneset (**C**) in MCF-7 cells treated with abemaciclib compared to vehicle-treated cells. The expression data were obtained from GSE99060. Data are mean ± SD. For (**A**): two-way ANOVA; ***$p < 0.001$.

 © The Author(s)

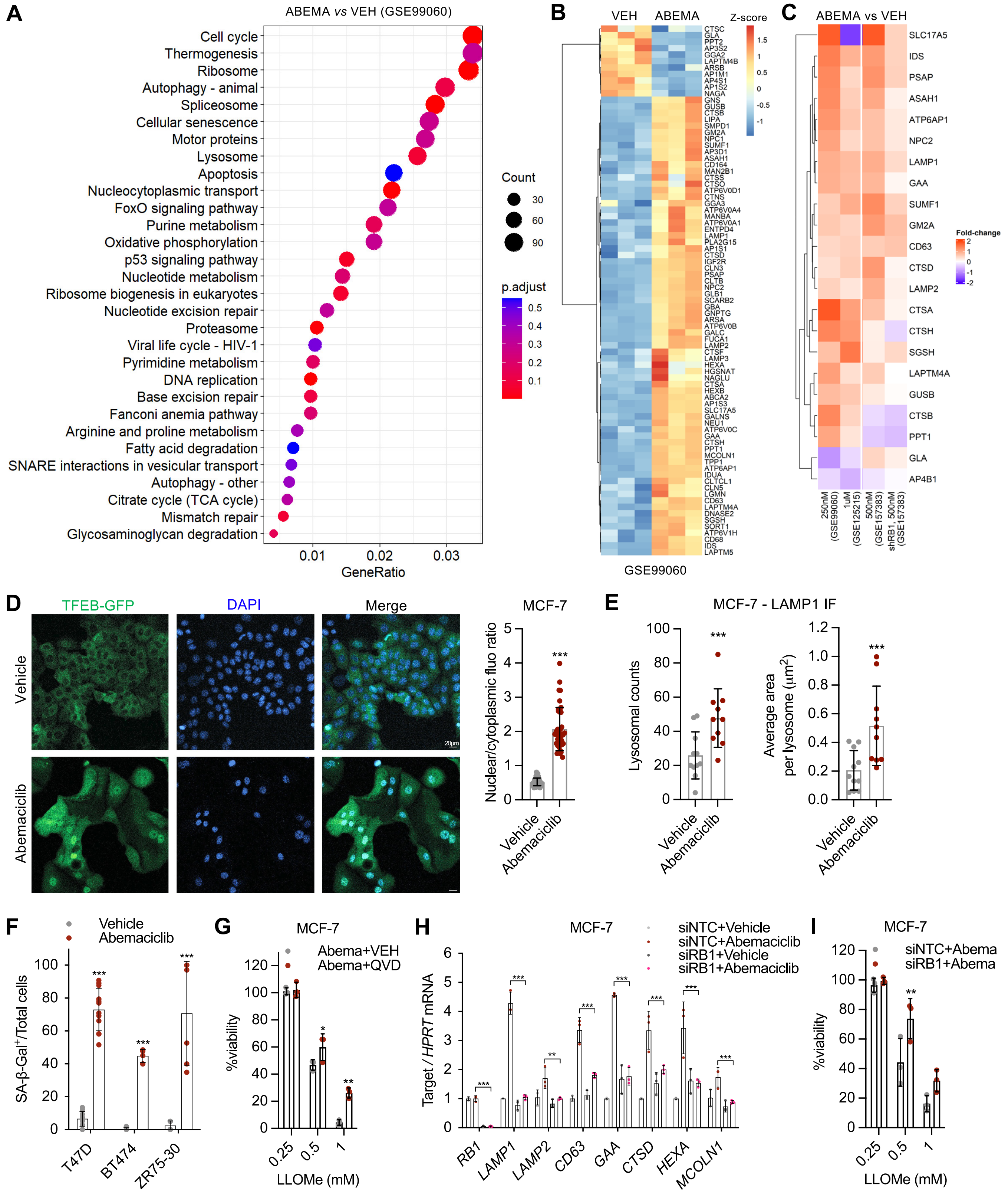
A
ABEMA vs VEH (GSE99060)
Cell cycle
Thermogenesis
Ribosome
Autophagy - animal
Spliceosome
Cellular senescence
Motor proteins
Lysosome
Apoptosis
Nucleocytoplasmic transport
FoxO signaling pathway
Purine metabolism
Oxidative phosphorylation
p53 signaling pathway
Nucleotide metabolism
Ribosome biogenesis in eukaryotes
Nucleotide excision repair
Proteasome
Viral life cycle - HIV-1
Pyrimidine metabolism
DNA replication
Base excision repair
Fanconi anemia pathway
Arginine and proline metabolism
Fatty acid degradation
SNARE interactions in vesicular transport
Autophagy - other
Citrate cycle (TCA cycle)
Mismatch repair
Glycosaminoglycan degradation
GeneRatio
Count
p.adjust
B
VEH
ABEMA
Z-score
GSE99060
C
ABEMA vs VEH
Fold-change
SLC17A5
IDS
PSAP
ASAH1
ATP6AP1
NPC2
LAMP1
GAA
SUMF1
GM2A
CD63
CTSD
LAMP2
CTSA
CTSH
SGSH
LAPTM4A
GUSB
CTSB
PPT1
GLA
AP4B1
250nM (GSE99060)
1uM (GSE125215)
500nM (GSE157383)
shRB1, 500nM (GSE157383)
D
TFEB-GFP
DAPI
Merge
Vehicle
Abemaciclib
MCF-7
Nuclear/cytoplasmic fluo ratio
E
MCF-7 - LAMP1 IF
Lysosomal counts
Average area per lysosome (μm²)
F
Vehicle
Abemaciclib
SA-β-Gal+/Total cells
T47D
BT474
ZR75-30
G
MCF-7
Abema+VEH
Abema+QVD
%viability
LLOMe (mM)
H
MCF-7
siNTC+Vehicle
siNTC+Abemaciclib
siRB1+Vehicle
siRB1+Abemaciclib
Target / HPRT mRNA
RB1
LAMP1
LAMP2
CD63
GAA
CTSD
HEXA
MCOLN1
I
MCF-7
siNTC+Abema
siRB1+Abema
%viability
LLOMe (mM)

Figure EV2. Abemaciclib treatment increases lysosomal mass in HR$^+$ breast cancer cells.

(**A**) Dot plot shows the significantly enriched GO terms when comparing the gene expression profiles of MCF-7 cells treated with abemaciclib to those of vehicle-treated cells. Hypergeometric test (clusterProfiler package) for dot plots. (**B**) Heatmap of differentially expressed lysosomal genes following abemaciclib treatment of MCF-7 cells. The expression data were obtained from GSE99060. (**C**) Heatmap of differentially expressed lysosomal genes following abemaciclib treatment (fold change *vs.* vehicle) in MCF-7 cells with the indicated doses and genetic backgrounds. Expression data were obtained from the GSE99060, GSE125215, and GSE157383 datasets. (**D**) MCF-7 cells labeled with TFEB-GFP via lentiviral particles were treated with either vehicle (water) or abemaciclib (1 µM for 48 h), followed by imaging to assess the subcellular localization of TFEB proteins (scale bar: 20 µm). The ratio of nuclear-to-cytoplasmic fluorescence signals was quantified and plotted. $n = 3$ independent experiments; $p < 0.0001$. (**E**) MCF-7 cells were treated with either vehicle (water) or abemaciclib (1 µM for 8 days), replated, and stained for LAMP1. The cells were then analyzed to quantify the lysosomal counts and average lysosomal area. The cells were obtained from three independent experiments ($p < 0.0001$). (**F**) Quantification of SA-β-Gal positive cells from Fig. 2J, $p < 0.0001$ for all. (**G**) MCF-7 cells were treated with abemaciclib, with or without QVD (5 µM), followed by treatment with LLOMe at the indicated concentrations. Cell viability was measured using the MTS assay. $n = 3$ independent experiments, $p_{0.5mM} = 0.0311$, $p_{1mM} = 0.009$. (**H**) MCF-7 cells were transfected with siRNA targeting *RB1* and then treated with vehicle or abemaciclib (1 µM for 5 days) 1 d after siRNA transfection. At the end of the treatment, RNA was extracted from both siNTC and siRB1 groups, and qPCR was performed to assess the expression of *RB1* and lysosomal genes. $n = 3$ independent experiments, $p_{LAMP2} = 0.005$, and other genes $p < 0.001$. (**I**) MCF-7 cells were transfected with siRNA targeting *RB1* and treated with either vehicle or abemaciclib (1 µM for 5 days), starting 1 day after siRNA transfection. Following this, the cells were replated and subjected to subsequent treatment with LLOMe for 48 h at the indicated concentrations. $n = 3$ independent experiments, $p_{0.5mM} = 0.0028$. Data are mean ± SD. For (**D**, **E**), unpaired Student's *t-tests* (two-tailed) were used. For (**F–I**), two-way ANOVA was used. *$p < 0.05$, **$p < 0.01$, ***$p < 0.001$.

 © The Author(s)

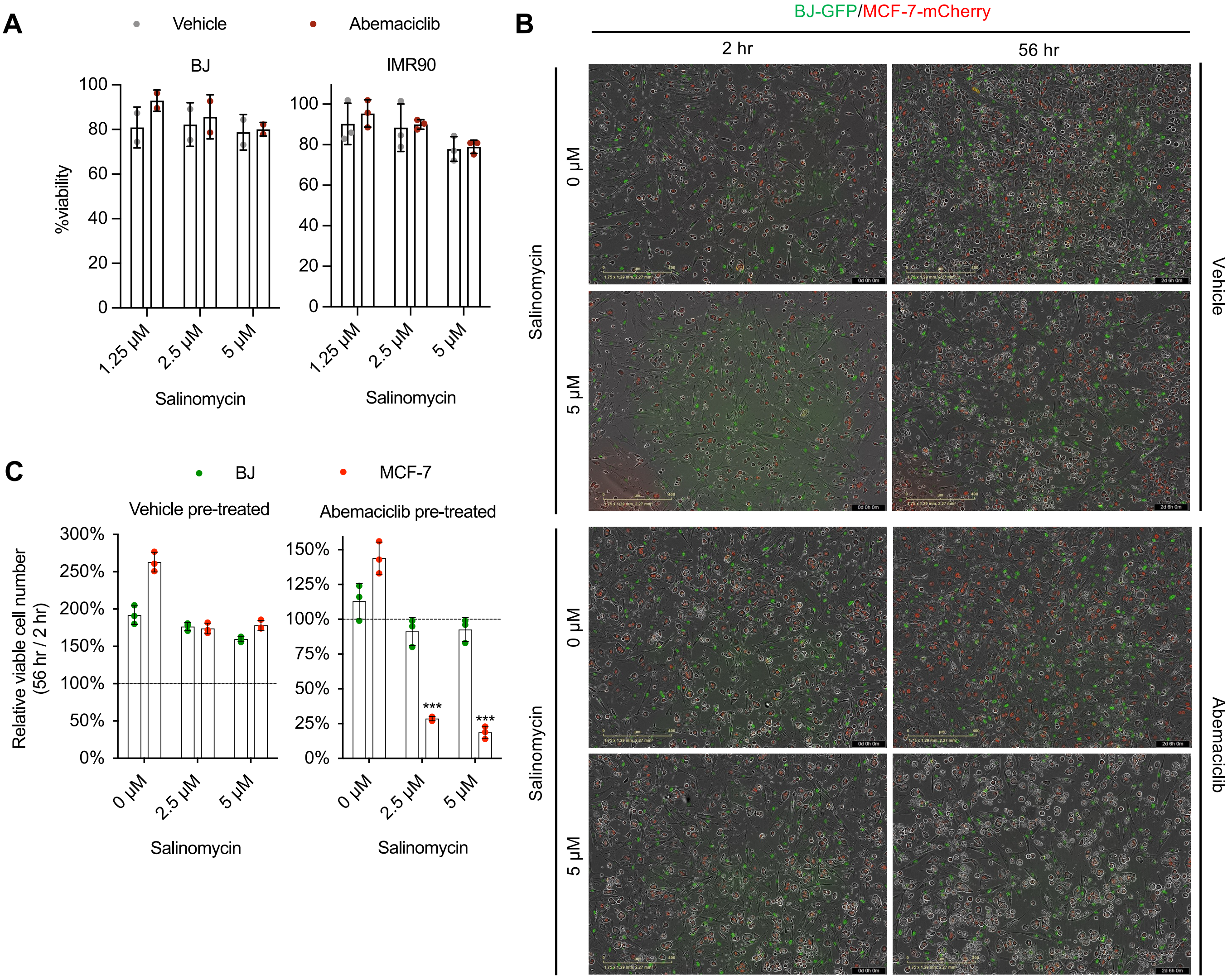

Figure EV3. Abemaciclib-treated normal fibroblasts are not sensitive to lysosomotropic agents.

(**A**) BJ and IMR90 cells were pretreated with either vehicle (water) or abemaciclib (1 µM for 7 days), followed by incubation with salinomycin at the indicated concentrations. Cell viability was assessed using an MTS assay. $n = 3$ independent experiments. (**B**) MCF-7 cells (mCherry) and BJ cells (GFP) were pretreated with either vehicle (water) or abemaciclib (1 µM for six days) and then co-cultured. These cells were subsequently incubated with salinomycin (0 or 5 µM for 56 h) and representative images were captured using IncuCyte at 2 and 56 h. (**C**) Ratio of viable MCF-7 and BJ cells (56-hr *vs* 2-hr) treated with different concentrations of salinomycin for both vehicle- and abemaciclib-pretreated groups. $n = 3$ independent experiments. $p < 0.001$ for all experiments. Data are mean ± SD. Two-way ANOVA. ***$p < 0.001$.

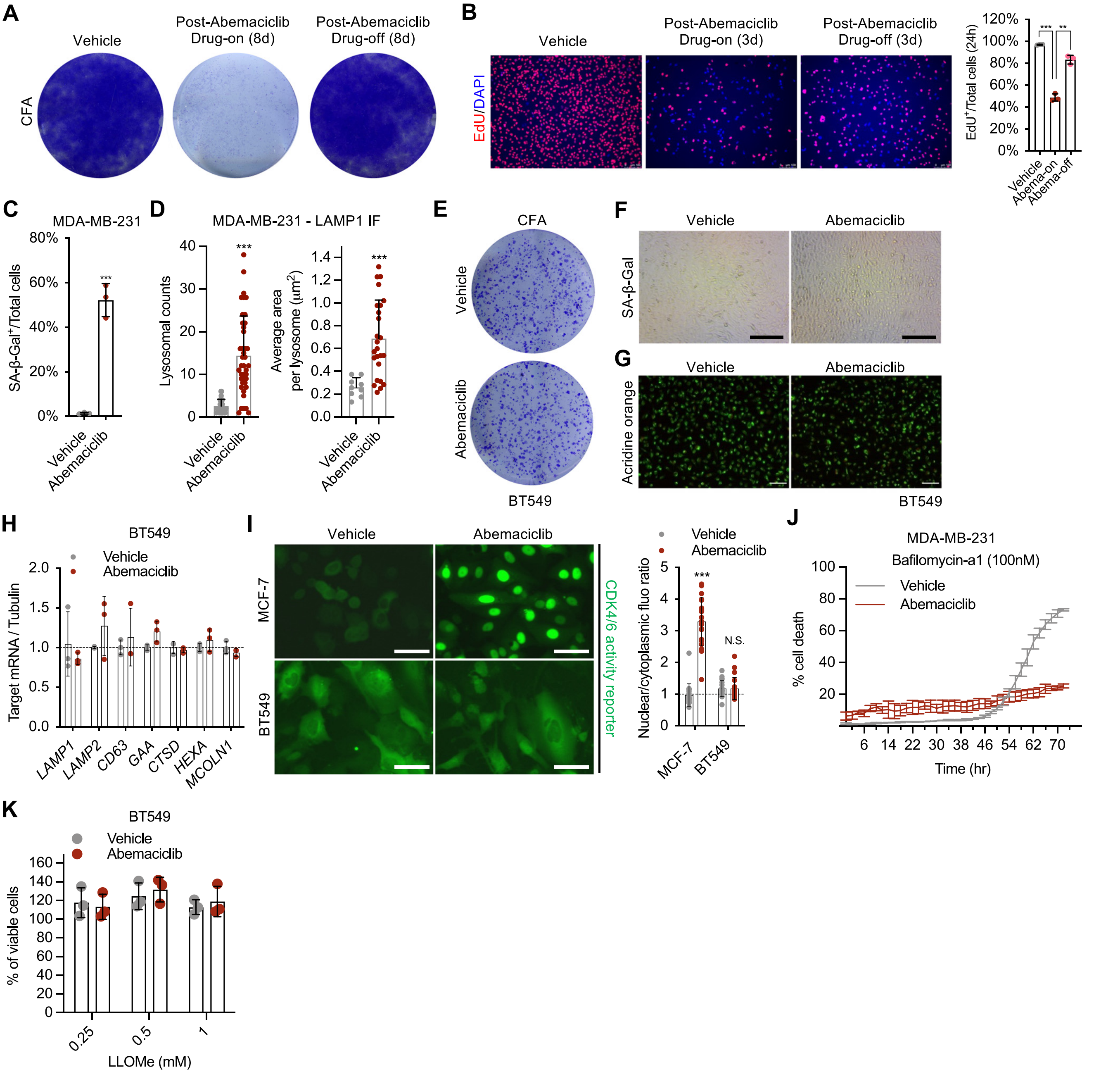
A
Vehicle
Post-Abemaciclib Drug-on (8d)
Post-Abemaciclib Drug-off (8d)
CFA
B
Vehicle
Post-Abemaciclib Drug-on (3d)
Post-Abemaciclib Drug-off (3d)
EdU/DAPI
EdU+/Total cells (24h)
Vehicle
Abema-on
Abema-off
C
MDA-MB-231
SA-β-Gal+/Total cells
D
MDA-MB-231 - LAMP1 IF
Lysosomal counts
Average area per lysosome (μm2)
E
CFA
BT549
F
SA-β-Gal
G
Acridine orange
H
BT549
Target mRNA / Tubulin
LAMP1
LAMP2
CD63
GAA
CTSD
HEXA
MCOLN1
I
MCF-7
BT549
CDK4/6 activity reporter
Nuclear/cytoplasmic fluo ratio
N.S.
J
MDA-MB-231
Bafilomycin-a1 (100nM)
% cell death
Time (hr)
K
BT549
% of viable cells
LLOMe (mM)
Vehicle
Abemaciclib

 © The Author(s)

Figure EV4. Abemaciclib selectively sensitizes triple-negative breast cancer cells to lysosomotropic agents-induced cell death.

(**A**, **B**) MDA-MB-231 cells were treated with either vehicle (water) or abemaciclib (1 µM for 8 days) and then replated for subsequent assays. For the colony formation assay (**A**), cells were cultured with or without abemaciclib for an additional eight days, followed by staining. For EdU staining (**B**), cells were replated and treated with or without abemaciclib for 3 days. EdU (10 µM) was added on day 4 and incubated for 20 h, followed by staining (scale bar, 100 µm). $n = 3$ independent experiments, $p_{on\text{-}vs\text{-}veh} < 0.0001$; $p_{on\text{-}vs\text{-}off} = 0.0027$. (**C**) Vehicle- or abemaciclib (1 µM for 8 days)-treated MDA-MB-231 cells were stained with LAMP1 and analyzed for lysosomal count and average area. $n = 3$ independent experiments; $p < 0.0001$. (**D**) MDA-MB-231 cells were treated with vehicle (water) or abemaciclib (1 µM for 8 days), replated and stained with LAMP1, and the cells were quantified for lysosomal counts or average area of lysosomes. The cells were obtained from three independent experiments ($p < 0.0001$). (**E–H**) BT549 cells were treated with vehicle (water) or abemaciclib (1 µM for 8 days), and then cells were replated for colony formation assay (8 days culture) (**E**), SA-β-Gal staining (scale bar, 1 mm) (**F**), acridine orange staining (scale bar, 1 mm) (**G**), or qRT-PCR for lysosomal genes (**H**), $n = 3$ independent experiments. (**I**) MCF-7 and BT549 cells were transfected with lentiviral particles encoding a CDK4/6 kinase activity reporter (CDK4KTR), followed by treatment with either vehicle or abemaciclib (1 µM for 48 h). Cells were imaged to analyze the localization of the mCherry protein (scale bar: 60 µm). Cytoplasmic localization of mCherry indicates active CDK4/6 kinases, whereas nuclear localization indicates suppressed CDK4/6 kinase activity. The nuclear-to-cytoplasmic mCherry fluorescence mean intensity ratio was calculated and plotted for each cell. The data represent cells from three independent experiments ($p < 0.001$). (**J**) Vehicle-or abemaciclib (1 µM for 8 days)-pretreated MDA-MB-231 cells were subsequently treated with bafilomycin A1, and cell death was measured using IncuCyte live cell imaging with Celltox™ Green. $n = 3$ independent experiments. (**K**) Vehicle- or abemaciclib-pretreated BT549 cells were subsequently treated with LLOMe and viability was measured using the MTS assay. $n = 3$ independent experiments. Data are mean ± SD. For (**B**), one-way ANOVA. For (**C**, **D**), unpaired Student's *t*-test (two-tailed) was used. For (**H–K**), two-way ANOVA was used. ***$p < 0.001$.

© The Author(s)

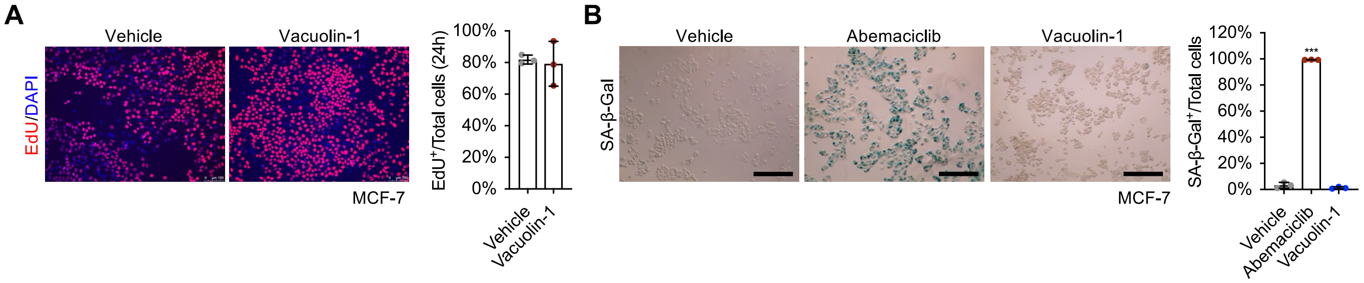

Figure EV5. Vacuolin-1 is unable to induce senescence-like phenotype.

(**A**, **B**) MCF-7 cells were treated with vehicle or vacuolin-1 (1 μM for 8 days) and re-plated for EdU staining (scale bar, 100 μm) (**A**) or SA-β-Gal staining (scale bar, 1 mm) (**B**). $n = 3$ independent experiments; $p < 0.0001$. Data are presented as the mean ± standard deviation (SD), one-way ANOVA. ***$p < 0.001$.

 © The Author(s)
